# Discovery of Novel Thiophene-arylamide Derivatives as DprE1 Inhibitors with Potent Antimycobacterial Activities

**DOI:** 10.1021/acs.jmedchem.1c00263

**Published:** 2021-04-14

**Authors:** Pengxu Wang, Sarah M. Batt, Bin Wang, Lei Fu, Rongfei Qin, Yu Lu, Gang Li, Gurdyal S. Besra, Haihong Huang

**Affiliations:** † Beijing Key Laboratory of Active Substance Discovery and Druggability Evaluation & Chinese Academy of Medical Sciences Key Laboratory of Anti-DR TB Innovative Drug Research, Institute of Materia Medica, Peking Union Medical College and Chinese Academy of Medical Sciences, 1 Xian Nong Tan Street, Beijing 100050, P. R. China; ‡ School of Biosciences, University of Birmingham, Birmingham B15 2TT, United Kingdom; § Beijing Key Laboratory of Drug Resistance Tuberculosis Research, Department of Pharmacology, Beijing Tuberculosis and Thoracic Tumor Research Institute, Beijing Chest Hospital, Capital Medical University, 97 Ma Chang Street, Beijing 101149, P. R. China

## Abstract

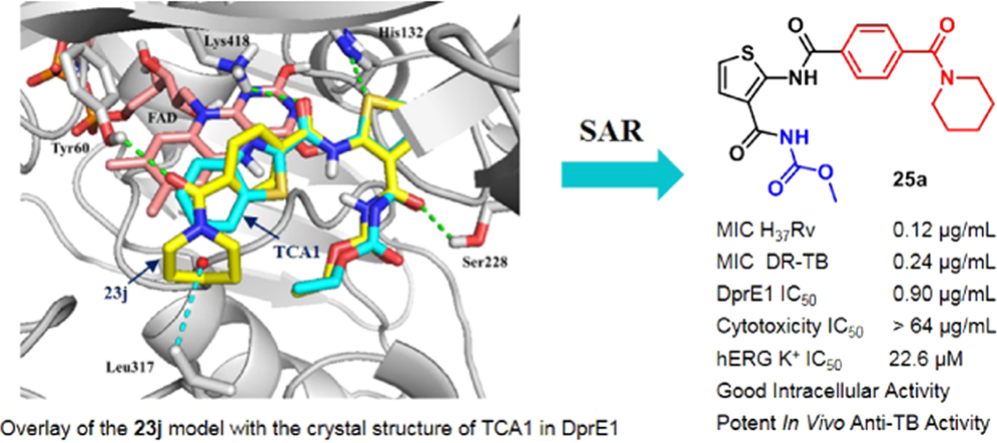

In this study, we report the design and synthesis of a series of novel thiophene-arylamide compounds derived from the noncovalent decaprenylphosphoryl-β-d-ribose 2′-epimerase (DprE1) inhibitor TCA1 through a structure-based scaffold hopping strategy. Systematic optimization of the two side chains flanking the thiophene core led to new lead compounds bearing a thiophene-arylamide scaffold with potent antimycobacterial activity and low cytotoxicity. Compounds **23j**, **24f**, **25a**, and **25b** exhibited potent *in vitro* activity against both drug-susceptible (minimum inhibitory concentration (MIC) = 0.02–0.12 μg/mL) and drug-resistant (MIC = 0.031–0.24 μg/mL) tuberculosis strains while retaining potent DprE1 inhibition (half maximal inhibitory concentration (IC_50_) = 0.2–0.9 μg/mL) and good intracellular antimycobacterial activity. In addition, these compounds showed good hepatocyte stability and low inhibition of the human ether-à-go-go related gene (hERG) channel. The representative compound **25a** with acceptable pharmacokinetic property demonstrated significant bactericidal activity in an acute mouse model of tuberculosis. Moreover, the molecular docking study of template compound **23j** provides new insight into the discovery of novel antitubercular agents targeting DprE1.

## Introduction

Tuberculosis (TB) is a chronic infectious disease caused primarily by pathogen *Mycobacterium tuberculosis* (*M. tuberculosis*). TB is one of the top 10 causes of death and the leading cause of mortality stemming from a single infectious agent. In 2020, the World Health Organization (WHO) reported that approximately 1.2 million human immunodeficiency virus (HIV)-negative people had died and 10 million new TB cases were identified. Globally, the TB incidence rate is falling but not fast enough to reach the 2020 milestone of a 20% reduction between 2015 and 2020.^1^ The COVID-19 pandemic threatens to reverse the recent progress in reducing the global burden of TB disease. The requirement for prolonged treatment with first-line drugs coupled with often difficult-to-manage side effects routinely leads to poor patient compliance and results in the accelerated emergence of drug-resistant strains of *M. tuberculosis*. Research focusing on the development of novel small molecules with activity against multidrug-resistant tuberculosis (MDR-TB) and extensively drug-resistant tuberculosis (XDR-TB) remains a significant challenge.^2,3^

The cell wall biosynthetic pathways have been identified as promising targets for the development of antitubercular agents.^4,5^ Decaprenylphosphoryl-β-d-ribose 2′-epimerase (DprE1) is crucial for mycobacterial cell wall biosynthesis.^6^ DprE1 catalyzes the flavin adenine dinucleotide (FAD)-dependent oxidation of decaprenylphosphoryl-β-d-ribose (DPR) to decaprenylphosphoryl-2′-keto-d-erythro-pentofuranose (DPX). DPX is then reduced by decaprenylphosphoryl-d-2-ketoerythropentose reductase (DprE2) to generate decaprenylphosphoryl-β-d-arabinofuranose (DPA), which is a unique precursor for the synthesis of cell-wall arabinans.^7,8^ Furthermore, DprE1 is specific to mycobacteria and actinomycetes, providing inherent biochemical selectivity over human cells and other bacterial species.^9^ Therefore, DprE1 has become a vulnerable target for treatment of drug-sensitive TB as well as MDR/XDR-TB.^10^

**Figure 1:**
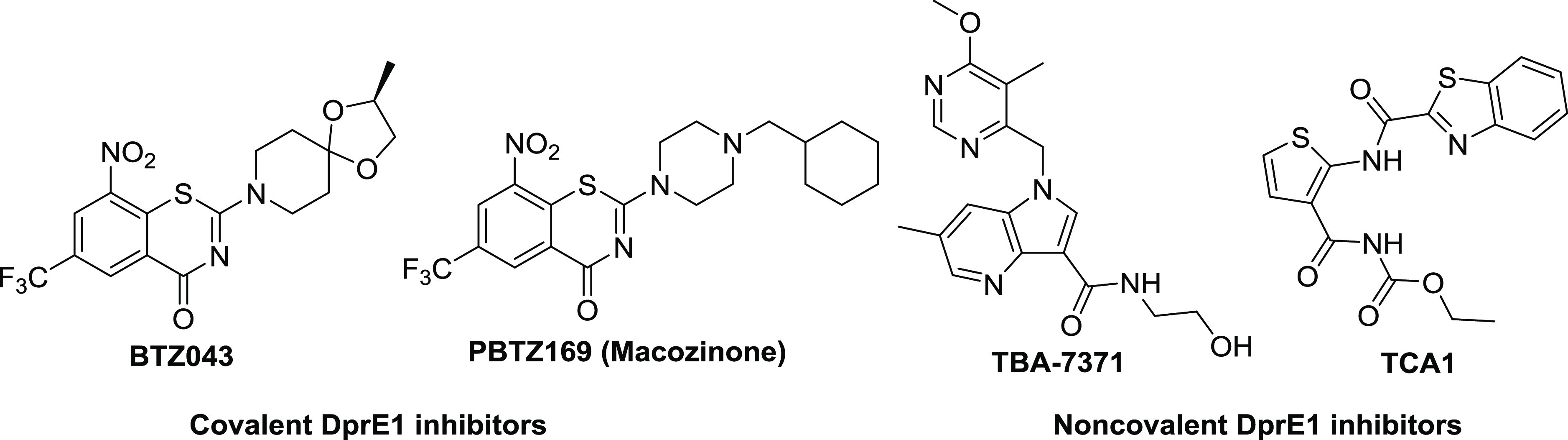
Structures of representative covalent and noncovalent DprE1 inhibitors.

A number of small molecules as anti-TB agents have so far been reported, with the benzothiazinones (BTZs) being the most well-developed and studied DprE1 inhibitors.^11,12^ Two compounds, BTZ043 and its next-generation analogue PBTZ169 (Macozinone), display potent antimycobacterial activity (minimum inhibitory concentrations (MICs) <0.016 μg/mL), and they are currently undergoing clinical development.^13,14^ These two small molecules likely impart their biological activity through formation of a covalent bond with Cys387 in the active site of DprE1. Recently, several structurally diverse noncovalent inhibitors of DprE1 have been described in the literature, most notably TBA-7371 and TCA1 (Figure 1).^6,15−19^ These compounds have the additional bonus of avoiding the nitro group used in BTZs, a well-established structural alert, and show the potential for further development. 1,4-Azaindole TBA-7371 has entered clinical development, and the structure–activity relationship (SAR) of this series has been fully explored and is well understood.^20,21^ TCA1 was identified *via* a cell-based phenotypic screen for inhibitors of biofilm formation in mycobacteria, which has bactericidal activity against replicating and nonreplicating *M. tuberculosis*.^22,23^ Inspired by the distinct thiophenamide moiety of TCA1, we have focused on the identification of a novel series of thiophene-arylamide compounds with improved activity and druggability derived from lead compound TCA1 through a scaffold hopping strategy.

**Figure 2:**
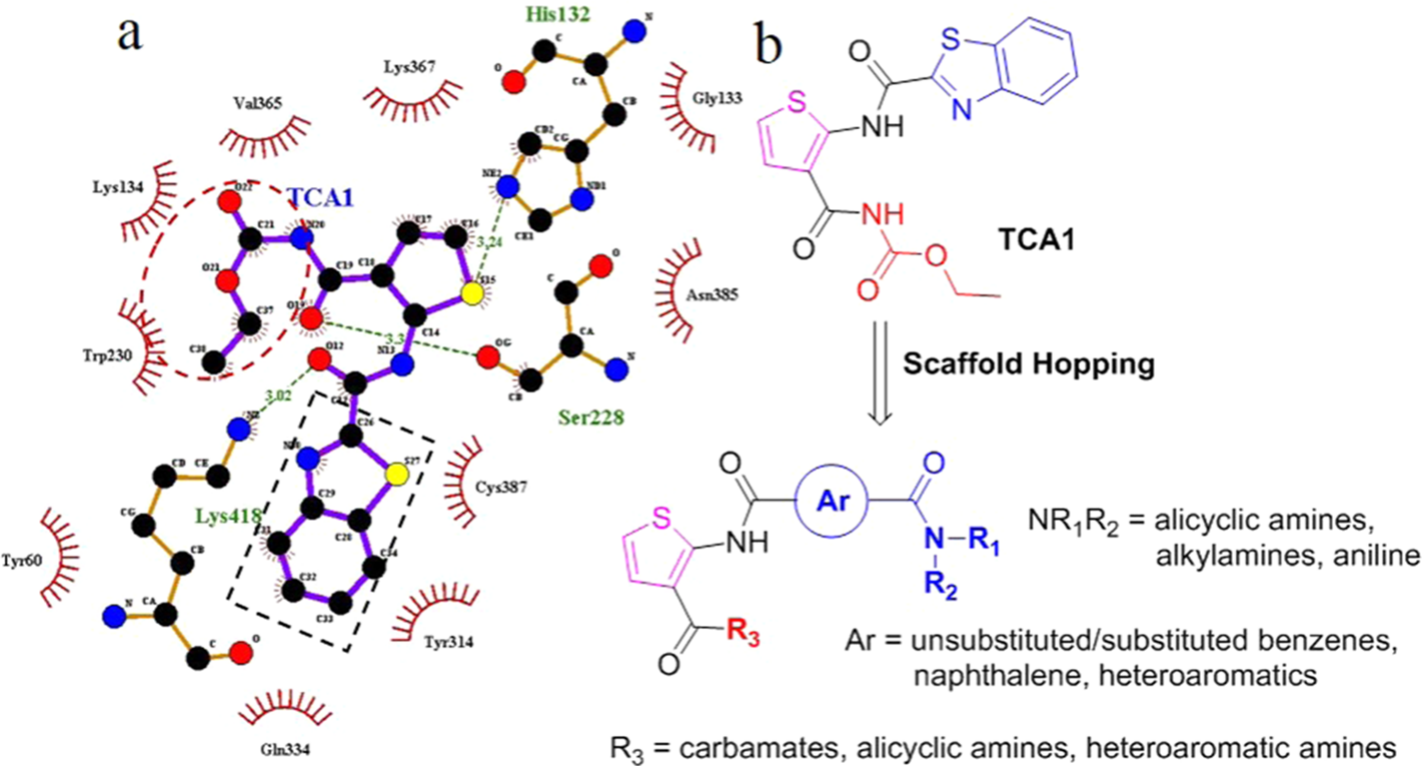
Optimization of TCA1 based on analysis of the cocrystal structure. (a) Noncovalent interactions of TCA1 with DprE1 and modification regions. (b) Design and optimization of thiophene-arylamide compounds as DprE1 inhibitors.

We began by analyzing the binding events displayed in TCA1 with the DprE1 cocrystal structure (Protein Data Bank (PDB): 4KW5) shown in Figure 2a.^22^ The thiophene moiety of TCA1 binds deeply within the bottom of the active site in DprE1 and shows that the noncovalent binding events between TCA1 and DprE1 are dominated by hydrophobic and van der Waals interactions. Further to these hydrophobic interactions, a clear hydrogen bond is formed between the thiophene moiety and His132 coupled with multiple hydrogen-bonding interactions between the carbonyl groups of TCA1 and residues Lys418 and Ser228 (Figure 2a). The binding mode indicated that the 2,3-disubstituted thiophene moiety plays an important role in maintaining key interactions of the cocrystal structure. The benzothiazole moiety is oriented parallel to the FAD isoalloxazine ring and forms additional hydrophobic interactions to keep critical pharmacodynamic conformation. We speculate that the addition of a hydrogen-bonding acceptor (HBA) in this region may enhance binding affinity with DprE1. Moreover, the terminal carbamate moiety at the 3-position on thiophene is considered to be metabolically labile and modification to this region may have an advantageous effect on overall metabolic stability.

Herein, we designed and synthesized a series of novel thiophene-arylamide derivatives to explore their structure–activity relationships (SARs) guided by the aforementioned crystal structure. These structural modifications were concentrated on the arylamide motif and carbamate moiety of TCA1 based on an evaluation of their antimycobacterial activities as well as preliminary druggability scoring (Figure 2b). A representative DprE1 inhibitor **25a** with acceptable pharmacokinetic (PK) properties demonstrated significant bactericidal activity in an acute mouse model of tuberculosis. Furthermore, molecular docking studies of the template compound **23j** with the benzamide moiety provide new insight into the discovery of novel anti-TB agents targeting DprE1.

## Results and Discussion

### Molecular Docking Study of Template Compound **23j** in DprE1

**Figure 3:**
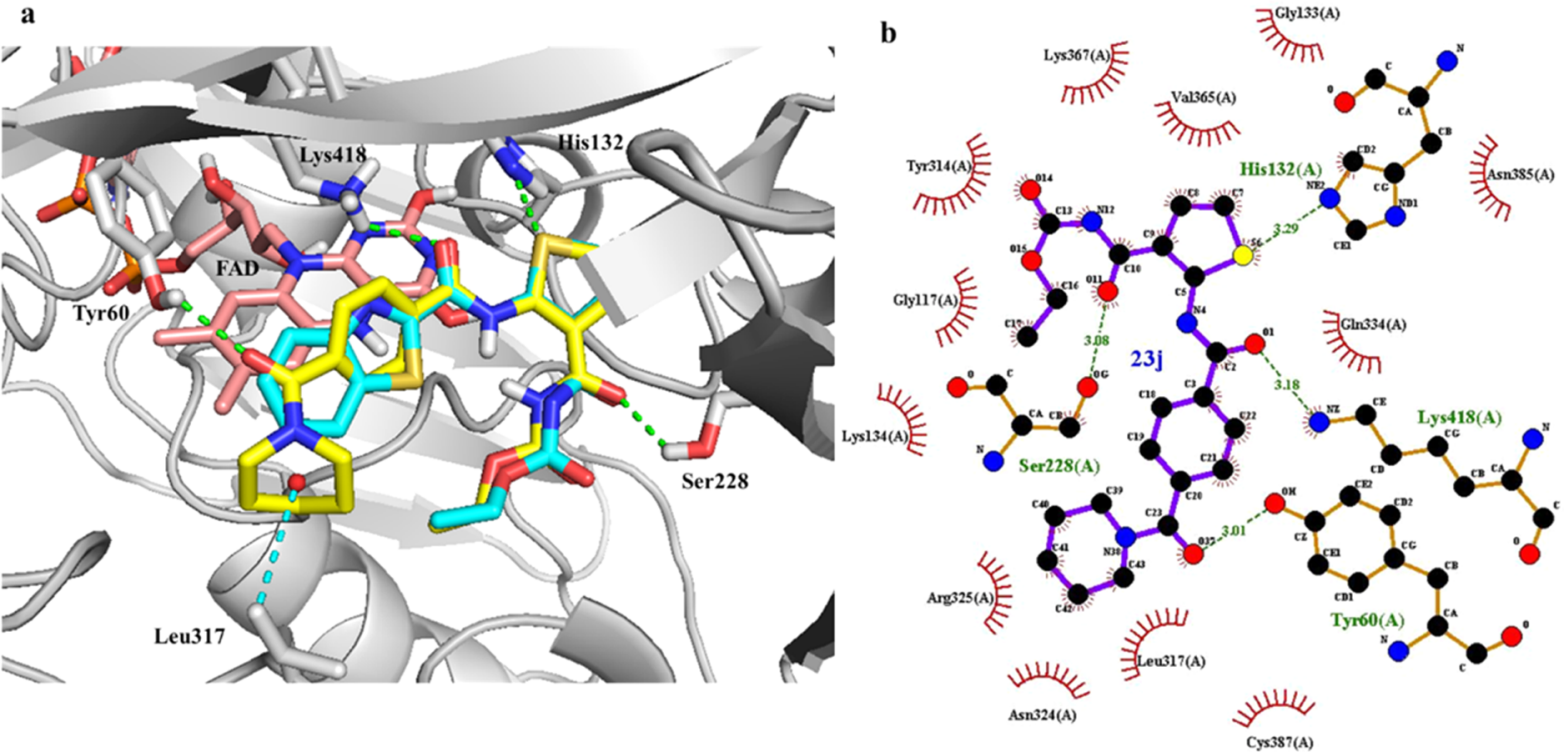
Molecular docking studies of compound **23j** in DprE1. (a) Overlay of the **23j** (yellow colored) model with the crystal structure of TCA1 (cyan) in complex with DprE1. (b) Docking model of **23j** in the binding site of *M. tuberculosis* DprE1 (PDB: 4KW5).

We began our study with molecular modeling to understand the binding mode of the template compound **23j** in the active site of DprE1 (Figure 3; PDB: 4KW5). The highest docking score conformation derived from the CDOCKER protocol was selected as the best binding pose. The overlay of the **23j** model with the crystal structure of TCA1 in complex with DprE1 showed similar interactions in the active site (Figure 3a). Hydrogen-bonding interactions were found between compound **23j** and residues Ser228, Lys418, and His132, which were consistent with the binding mode of TCA1. Furthermore, the phenyl ring was oriented roughly parallel to the isoalloxazine of FAD and formed hydrophobic interactions with residues Gln334 and Cys387. Interestingly, the acyl of benzamide formed a key hydrogen bond with Tyr60, and the terminal piperidine interacted with residues Leu317, Arg325, and Asn324 (Figure 3b). This additional reinforced interaction of acyl piperidine may enhance the binding affinity and therefore improve the antimycobacterial activity. This predicted model, which was in line with our design strategy, prompted us to more closely explore the SAR exhibited by the thiophene compounds containing this arylamide moiety.

### Chemistry

**Scheme 1:**
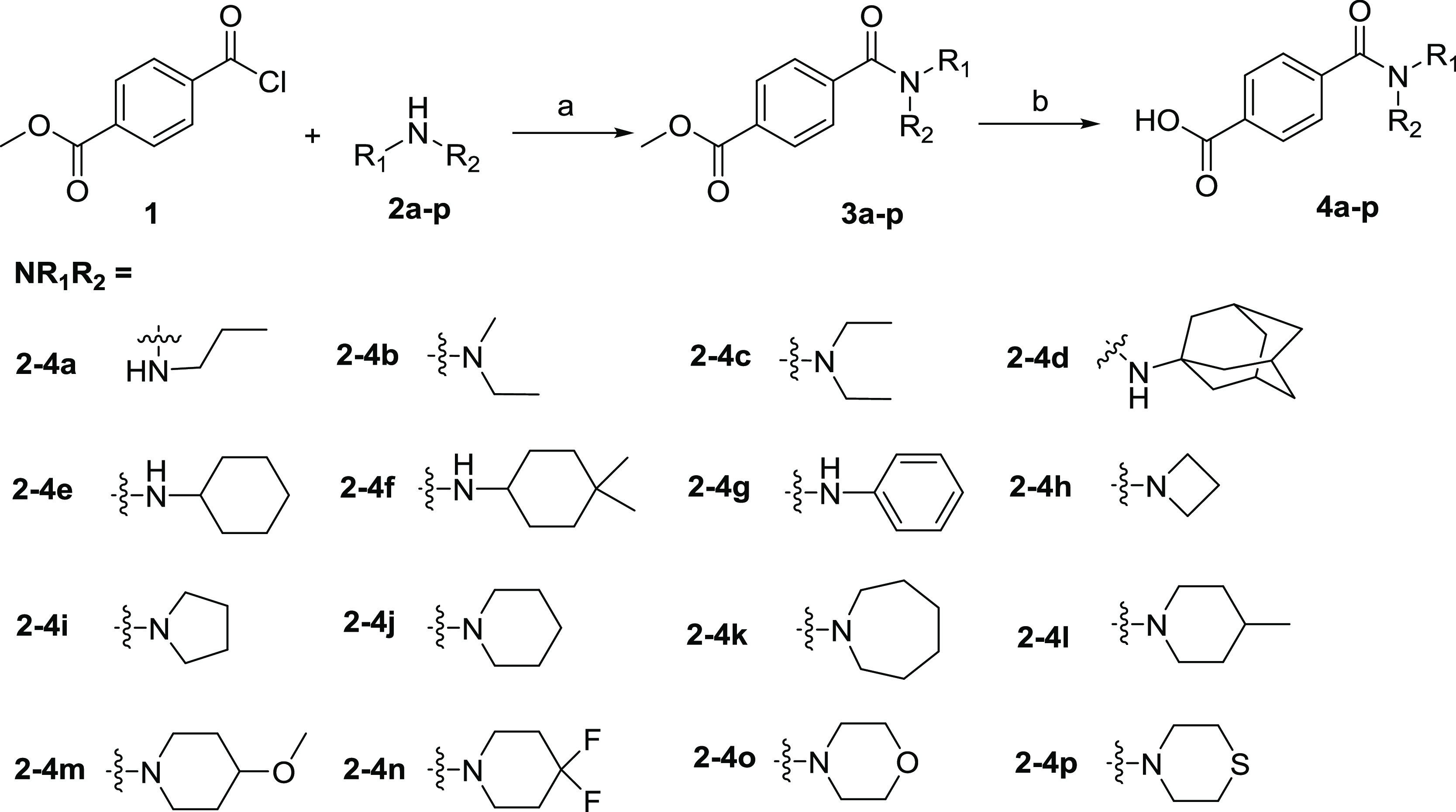
Synthesis of Benzoic Acid with Different Amide Substituents **4a**–**p** Reagents and conditions: (a) Et_3_N, CH_2_Cl_2_, room temperature (rt), 3 h; (b) 1 mol/L LiOH aqueous solution, CH_3_OH, rt, 3 h.

**Scheme 2:**
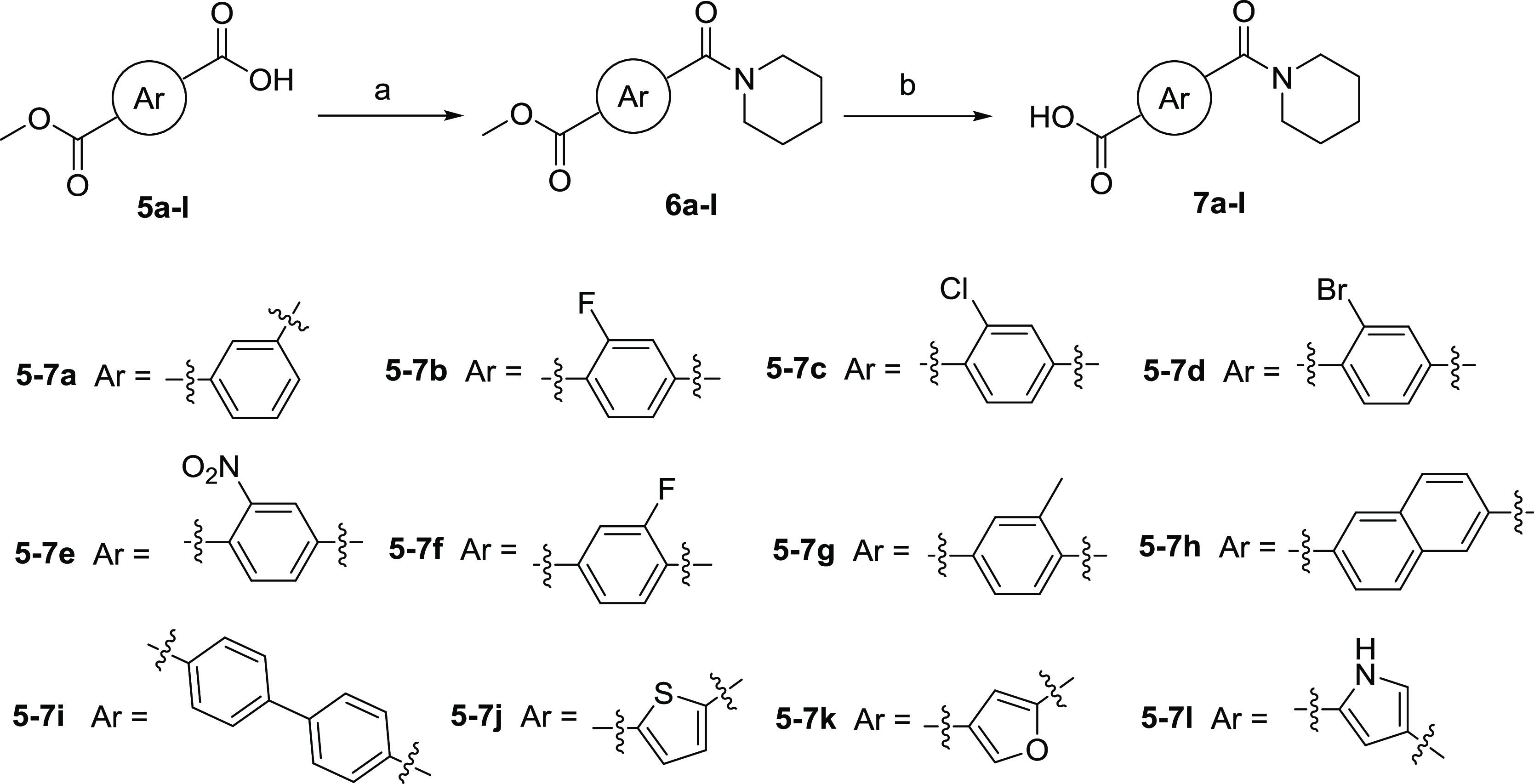
Synthesis of Aryl Carboxylic Acid with Piperidinamide Substituents **7a**–**l** Reagents and conditions: (a) piperidine, 2-(7-aza-1*H*-benzotriazole-1-yl)-1,1,3,3-tetramethyluronium hexafluorophosphate (HATU), Et_3_N, dimethylformamide (DMF), rt, 10 h; (b) 1 mol/L LiOH aqueous solution, CH_3_OH, rt, 3 h.

**Scheme 3:**
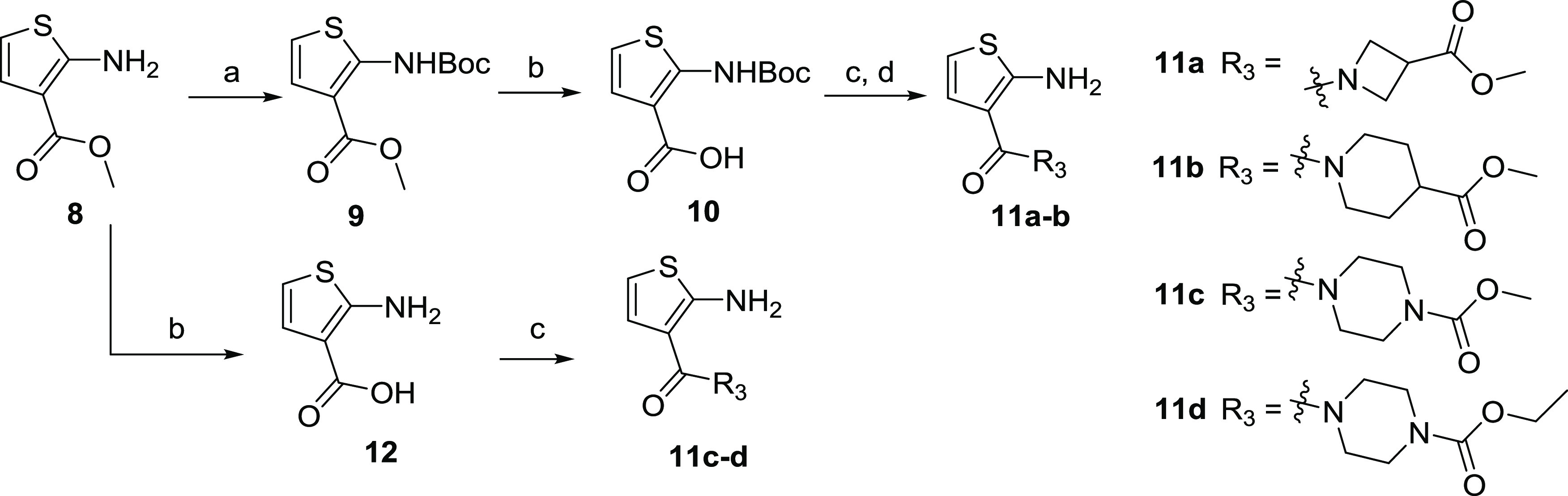
Synthesis of Aminothiophene with Different Amide Substituents **11a**–**d** Reagents and conditions: (a) di-*tert*-butyl decarbonate ((Boc)_2_O), 4-dimethylaminopyridine (DMAP), Et_3_N, CH_2_Cl_2_, rt, 6 h; (b) 1 mol/L NaOH aqueous solution, CH_3_OH, reflux, 4 h; (c) alicyclic amines, 1-(3-dimethylaminopropyl)-3-ethylcarbodiimide hydrochloride (EDCI), 1-hydroxybenzotriazole (HOBt), Et_3_N, DMF, rt, 12 h; (d) trifluoroacetic acid (TFA), CH_2_Cl_2_, rt, 2.5 h.

**Scheme 4:**
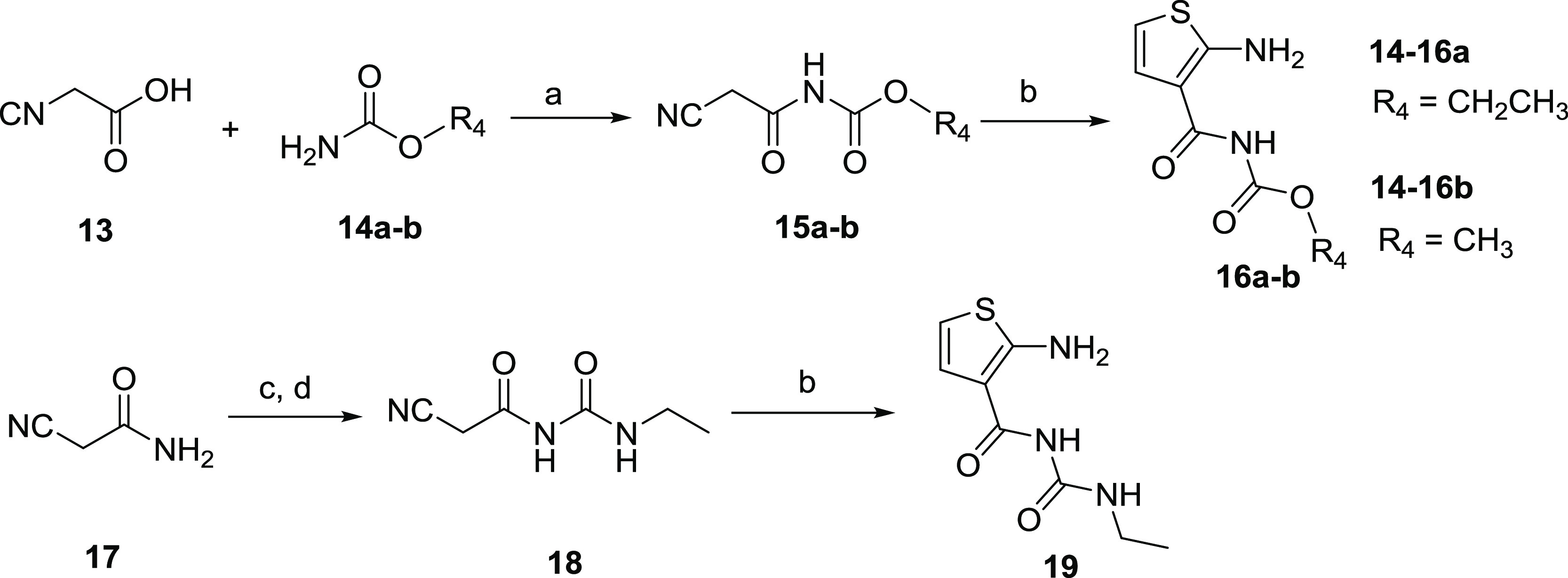
Synthesis of Aminothiophene Intermediates **16a**, **16b**, and **19** Reagents and conditions: (a) phosphorus oxychloride, DMF, toluene, 80 °C, 3 h; (b) 2,5-dihydroxy-1,4-dithiane, Et_3_N, CH_3_OH, 50 °C, 2.5 h; (c) oxalyl chloride, 1,2-dichloroethane, reflux, 4 h; (d) ethylamine, CH_3_CN, −10 °C, 3 h.

**Scheme 5:**
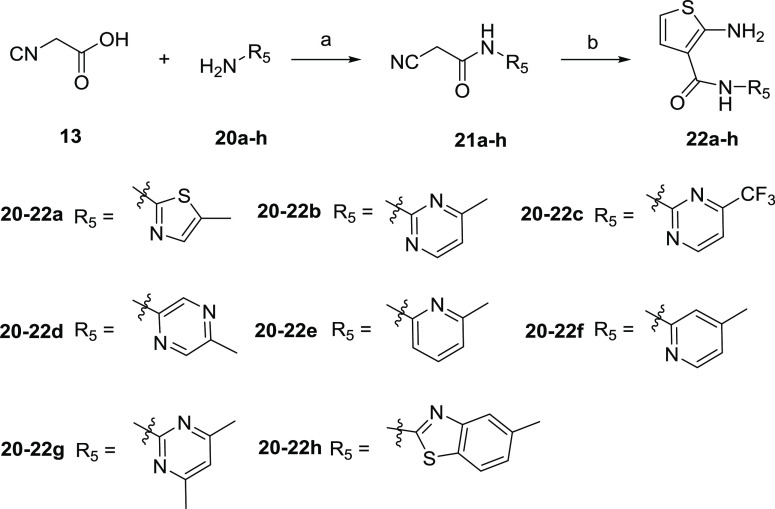
Synthesis of Aminothiophene Intermediates **22a**–**h** Reagents and conditions: (a) EDCI, DMAP, DMF, rt, 24 h; (b) 2,5-dihydroxy-1,4-dithiane, Et_3_N, CH_3_OH, 50 °C, 7 h.

**Scheme 6:**
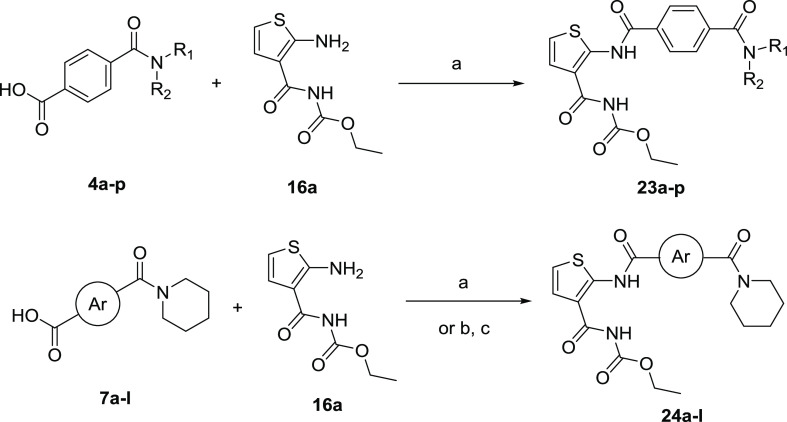
Synthesis of Target Compounds **23a**–**p** and **24a**–**l** Reagents and conditions: (a) HATU, Et_3_N, DMF, rt, 12 h; (b) SOCl_2_, DMF, CH_2_Cl_2_, reflux, 3 h; (c) Et_3_N, DMAP, tetrahydrofuran (THF), rt, 3 h.

**Scheme 7:**
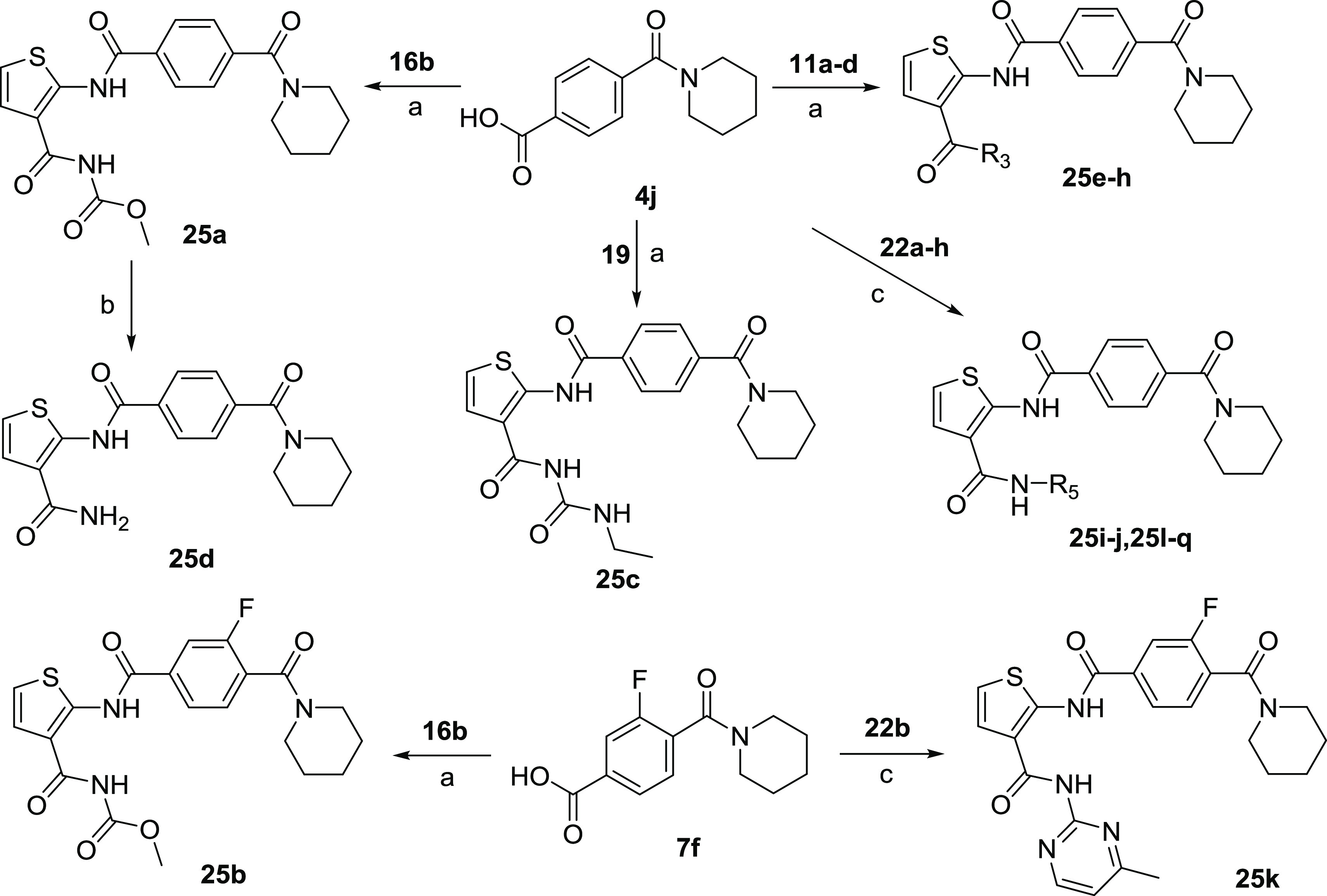
Synthesis of Target Compounds **25a**–**q** Reagents and conditions: (a) HATU, Et_3_N, DMF, rt, 12 h; (b) 1 mol/L LiOH aqueous solution, CH_3_OH, reflux, 2 h; (c) EDCI, HOBt, Et_3_N, DMF, rt, 24 h.

The synthesis of aryl carboxylic acids with various amide motifs **4a**–**p** and **7a**–**l** is outlined in Schemes 1 and 2. The substituted aminothiophene intermediates **11a**–**d**, **16a**, **16b**, **19**, and **22a**–**h** were synthesized following the procedures summarized in Schemes 3–5. The general synthetic procedures of target compounds **23a**–**p**, **24a**–**l**, and **25a**–**q** through condensation reactions are illustrated in Schemes 6 and 7.

As shown in Scheme 1, intermediates **3a**–**p** were obtained *via* amidation of commercially available methyl 4-(chlorocarbonyl)benzoate (**1**) with the corresponding amines **2a**–**p** in the presence of triethylamine. The obtained **3a**–**p** were converted to intermediates **4a**–**p***via* hydrolysis with aqueous lithium hydroxide solution.

According to Scheme 2, the condensation reactions of various aryl carboxylic acids **5a**–**l** with piperidine afforded corresponding intermediates **6a**–**l** in the presence of 2-(7-aza-1*H*-benzotriazole-1-yl)-1,1,3,3-tetramethyluronium hexafluorophosphate (HATU). The hydrolysis of **6a**–**l** led to key intermediates **7a**–**l**.

As shown in Scheme 3, methyl 2-aminothiophene-3-carboxylate (**8**) was reacted with (Boc)_2_O to form intermediate **9**, and subsequent hydrolysis led to thiophene carboxylic acid **10**. Key intermediates **11a** and **11b** were obtained *via* condensation of **10** with alicyclic amines and routine *N*-Boc deprotection. In addition, intermediates **11c** and **11d** were prepared from **8***via* a two-step hydrolysis/condensation strategy without the need for *N*-Boc protection and deprotection.

The synthesis of aminothiophene intermediates **16a**, **16b**, **19**, and **22a**–**h** is summarized in Schemes 4 and 5. 2-Cyanoacetic acid (**13**) was treated with carbamates **14a**, **14b** in the presence of phosphorus oxychloride to form intermediates **15a** and **15b**. Aminothiophene intermediates **16a** and **16b** were obtained by heterocyclization of **15a**, **15b** with 2,5-dihydroxy-1,4-dithiane *via* a Gewald reaction.^23^ 2-Cyanoacetamide (**17**) was reacted with oxalyl chloride under reflux to provide the isocyanate, and a subsequent reaction with ethylamine gave intermediate **18** without further purification, which followed the Gewald reaction to afford aminothiophene intermediate **19**. The condensation reactions of 2-cyanoacetic acid (**13**) with various aryl amines **20a**–**h** delivered the corresponding intermediates **21a**–**h** in the presence of EDCI with DMAP at room temperature. The Gewald heterocyclization of **21a**–**h** with 2,5-dihydroxy-1,4-dithiane produced aminothiophene intermediates **22a**–**h**.

As illustrated in Schemes 6 and 7, target compounds **23a**–**p**, **24a**–**l**, and **25a**–**q** were conveniently obtained through the condensation reaction with aryl carboxylic acids and aminothiophenes. Ethyl(2-aminothiophene-3-carbonyl)carbamate (**16a**) was subjected to the condensation reaction with various 4-carbamoylbenzoic acids **4a**–**p** in the presence of HATU to afford the corresponding products **23a**–**p**. In the same way, the target products **24a**–**l** were obtained from **16a** and the corresponding carbamoyl aromatic acid or carbamoyl benzoyl chloride.

4-(Piperidine-1-carbonyl)benzoic acid (**4j**) was reacted with **16b**, **19**, **11a**–**d**, and **22a**–**h** to afford the corresponding products **25a**, **25c**, **25e**–**h**, **25i**–**j**, and **25l**–**q** under the standard condensation conditions. The subsequent hydrolysis of **25a** with aqueous lithium hydroxide provided compound **25d**. In addition, 3-fluoro-4-(piperidine-1-carbonyl)benzoic acid (**7f**) was condensed with **16b** or **22b** to form the desired products **25b** or **25k**, respectively.

### SAR Optimization Strategy

The target compounds were evaluated for their activities against *M. tuberculosis* H_37_Rv using the microplate Alamar blue assay (MABA). Minimum inhibitory concentration (MIC) was defined as the lowest concentration resulting in a reduction in fluorescence of ≥90% relative to the mean of replicate bacterium-only controls. The compounds with MIC less than 1 μg/mL were further tested for mammalian cell cytotoxicity using Vero cells measured by the concentration required for inhibiting 50% cell growth (half maximal inhibitory concentration (IC_50_)) as compared to the no-treatment control. Tables 1–9 summarize the biological data including *in vitro* and *in vivo* anti-TB activity, toxicity, metabolic stability, PK, and target validation for these novel thiophene-arylamide derivatives. TCA1, isoniazid (INH), and rifampicin (RFP) were used as reference compounds for the anti-TB activity assay.

**Table 1:**
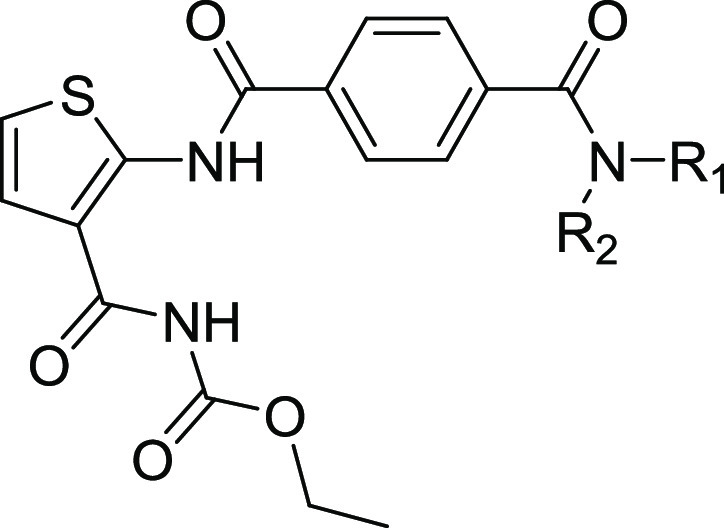

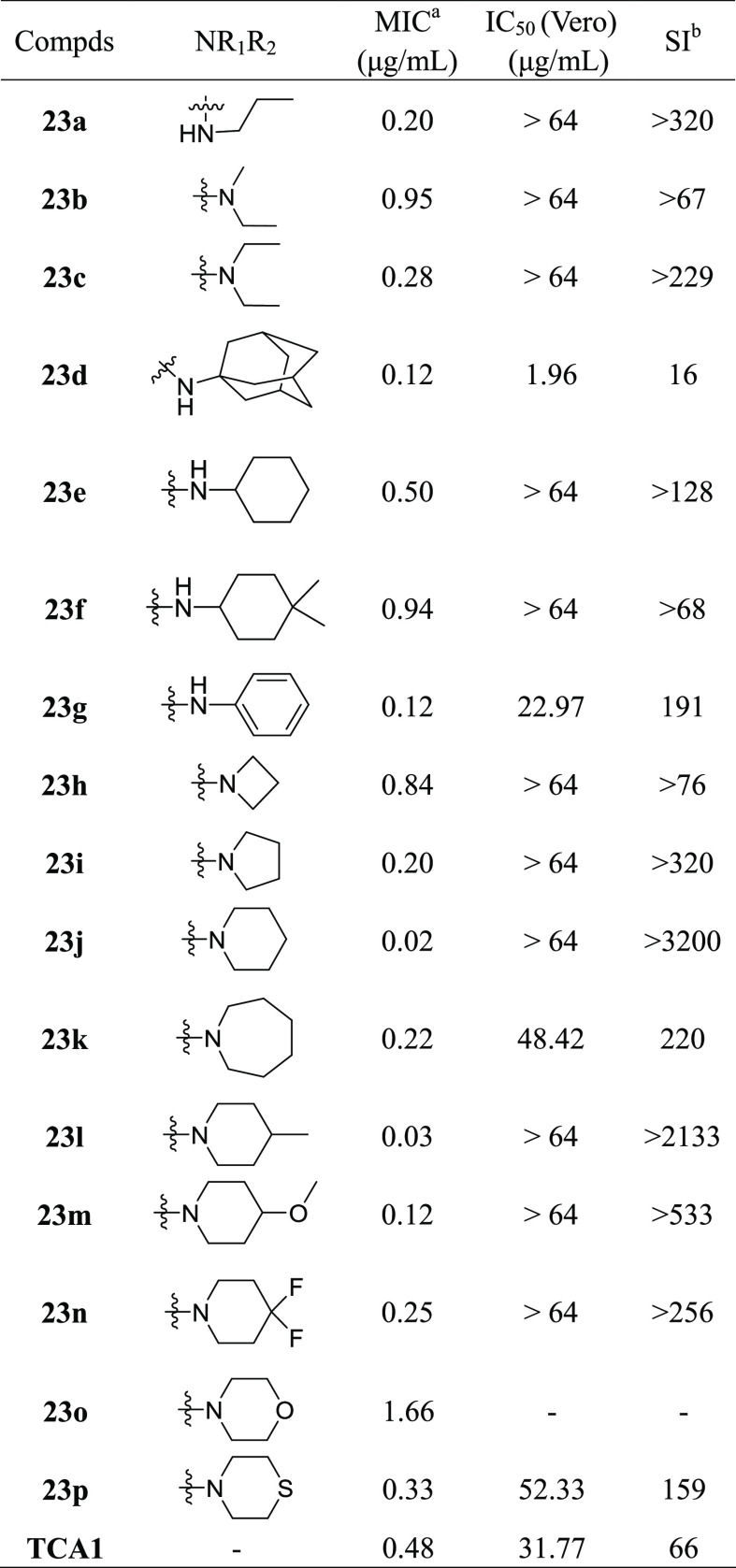
SAR of Thiophene-arylamide Compounds at R_1_ and R_2_ Sites

a MIC against *M. tuberculosis* H_37_Rv.

b SI = selectivity index, IC_50_/MIC.

**Table 2:**
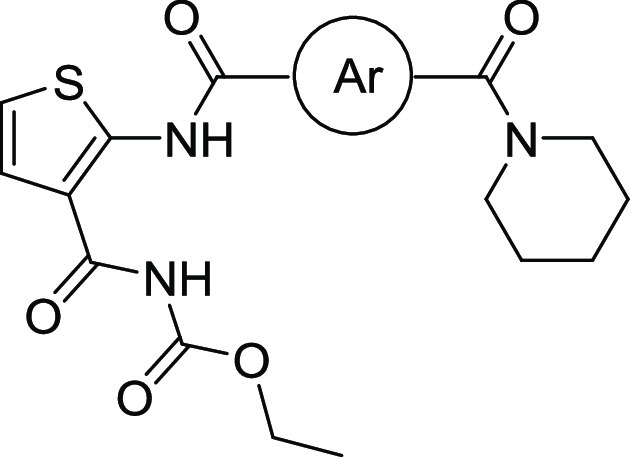

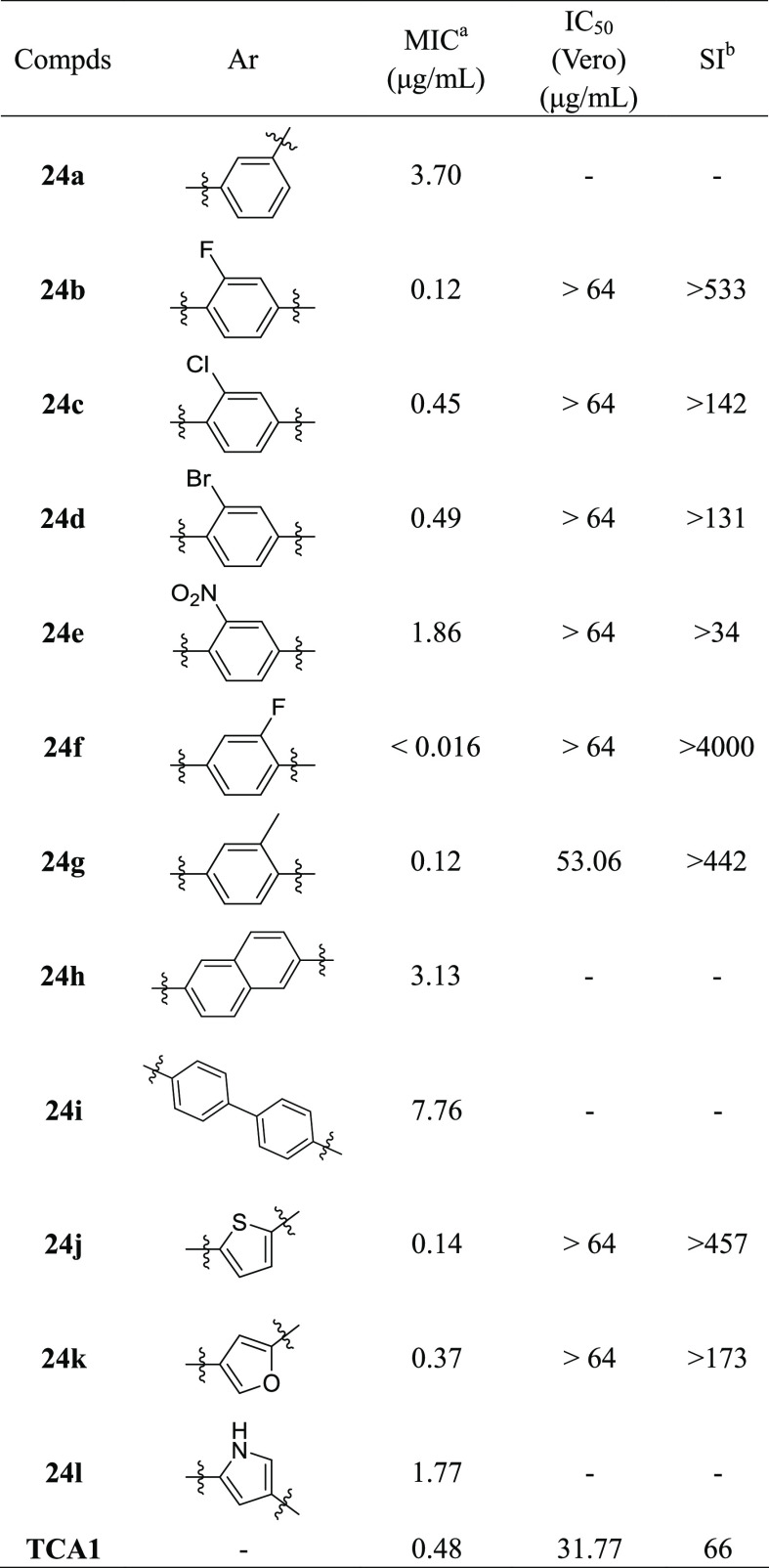
SAR of Thiophene-arylamide Compounds at the Ar Site

a MIC against *M. tuberculosis* H_37_Rv.

b SI = selectivity index, IC_50_/MIC.

Keeping 2,3-disubstituted thiophene as the key core, initial MIC-based SAR studies against *M. tuberculosis* around the lead compound TCA1 led to the first thiophene-benzamide series. The analysis of noncovalent interactions of TCA1 with DprE1 and modification sites (Figure 2) indicated that the binding pocket in this active site is deep and not fully occupied by the terminal benzothiazole moiety of the 2-position side chain on the thiophene ring. Therefore, a variety of benzamides with different physical and chemical properties were introduced to further improve the antimycobacterial activity and druggability profile. As listed in Table 1, to our delight, compound **23a** with the terminal propylamine exhibited good potency with improved MIC compared with TCA1 (0.2 *vs* 0.48 μg/mL). Furthermore, compound **23a** displayed lower cytotoxicity against Vero cells with IC_50_ > 64 μg/mL (SI > 320). Encouraged by the promising results, we evaluated a series of secondary amines and the bulky cyclic amines at the terminal of the side chain. The subsequent results revealed that diethylamine (**23c**, MIC = 0.28 μg/mL) was tolerated at the R_1_ and R_2_ positions, whereas the slightly smaller ethyl(methyl) amine (**23b**, MIC = 0.95 μg/mL) showed a decrease in activity. Encouraged by this result, large substituents, such as amantadine, cyclohexylamine, and aniline moieties were introduced to the side chain, aiming to explore the optimum volume at the R_1_ and R_2_ sites. Compounds **23d** and **23g** bearing amantadine and aniline moieties, respectively, showed improved antimycobacterial activities as compared to the reference compound TCA1 (0.12 *vs* 0.48 μg/mL) but showed a certain level of cytotoxicity. Additionally, increasing the overall volume through the addition of gem-dimethyl to the cyclohexylamine led to lower potency (**23e** and **23f**). Based on the above results, conformationally restricted secondary amines were embedded at the terminal of the side chain to further investigate the most favorable size for optimal occupancy of hydrophobic pockets of DprE1. The results indicated that smaller substituents such as azetidine (**23h**, MIC = 0.84 μg/mL) led to lower potency, while the larger pyrrolidine (**23i**, MIC = 0.20 μg/mL), piperidine (**23j**, MIC = 0.02 μg/mL), and azepane (**23k**, MIC = 0.22 μg/mL) moieties showed improved antimycobacterial activities. In particular, compound **23j** showed a significant improvement in MIC, corresponding to a 24-fold enhancement of potency relative to TCA1 (0.02 *vs* 0.48 μg/mL). In addition, **23j** also displayed high selectivity index (SI > 3200), indicating a good safety profile. Subsequently, the addition of methyl to the piperidine generated compound **23l**, which showed a MIC of 0.03 μg/mL, demonstrating its equivalency in antimycobacterial activity to **23j**. The replacement of methyl (**23l**, MIC = 0.03 μg/mL) with methoxyl (**23m**, MIC = 0.12 μg/mL) or difluoro (**23n**, MIC = 0.25 μg/mL) substituents resulted in lower potency. The bioisosteric replacement strategy to replace methylene (**23j**, MIC = 0.02 μg/mL) with an oxygen (**23o**, MIC = 1.66 μg/mL) or a sulfur (**23p**, MIC = 0.33 μg/mL) atom caused a significant decrease in antimycobacterial activity.

Based on the results from Table 1, coupled with the molecular docking studies, and keeping the privileged acyl piperidine fragment, we decided to explore the effect of the aryl moiety on antimycobacterial activity. Compared to compound **23j**, substitution at the meta-position of the phenyl ring instead of the para-position led to a marked decrease in potency, as exemplified by compound **24a** (MIC = 3.70 μg/mL). We next explored the introduction of halo, nitro, and methyl substituents on the phenyl ring, which have the potential to form π–π interactions in the hydrophobic pocket of DprE1. Introducing fluoro, chloro, and bromo substituents to the phenyl ring afforded compounds **24b**–**d**, which displayed good activity with a range of MICs 0.12–0.49 μg/mL. The involvement of the strongly electron-withdrawing nitro group (**24e**, MIC = 1.86 μg/mL) led to lower potency. Compound **24f** exhibited a significant increase in potency by altering the position of the fluoro group, corresponding to a 10-fold enhancement of potency relative to **24b** (MIC < 0.016 *vs* 0.12 μg/mL). The introduction of a methyl group (**24g**, MIC = 0.12 μg/mL) at the same position resulted in equal potency compared to **24b**. However, replacement with naphthalene (**24h**, MIC = 3.13 μg/mL) and biphenyl (**24i**, MIC = 7.76 μg/mL) caused an obvious decrease in activity. Subsequently, introduction of smaller five-membered aromatic heterocycles, such as thiophene (**24j**, MIC = 0.14 μg/mL), furan (**24k**, MIC = 0.37 μg/mL), and pyrrole (**24l**, MIC = 1.77 μg/mL) resulted in moderate to good antimycobacterial activity. Accordingly, we drew the conclusion that a phenyl ring bearing the electron-withdrawing fluoro substituent was the best tolerated at the Ar site.

Since the thorough SAR at the 2-position side chain of thiophene has been investigated, our attention shifted to the exploration of the 3-position with substituents at the R_3_ site. As listed in Table 3, compounds **25a** and **25b** with imide methyl ester showed better antimycobacterial activity than TCA1 (MIC 0.19 and 0.03 μg/mL *vs* 0.48 μg/mL). Among them, fluoro-substituted phenyl derivative (**25b**) showed a MIC of 0.03 μg/mL and an SI value above 2133. Replacing the ethyl ester (**23j**) with ethyl amide (**25c**) resulted in a dramatic loss of antimycobacterial activity. Removing the terminal acyl ester also led to an inactive compound (**25d**). Next, keeping the terminal acyl ester moiety, rigid alicyclic amines (**25e**–**h**) were introduced to further evaluate the size differential of substituents at the R_3_ site; however, all sterically encumbered substituents were not tolerated, resulting in a large loss of potency. When we replaced the carbamate with various rigid aromatic heterocycles aiming to enhance metabolic stability, compounds with meta-substituted pyrimidine (**25j**, **25k**, and **25l**) showed good antimycobacterial activity, especially **25l** with a MIC of 0.2 μg/mL. The introduction of thiazole, pyridine, and benzothiazole led to the total loss of potency (**25i**, **25n**, **25o**, and **25q**).

**Table 3:**
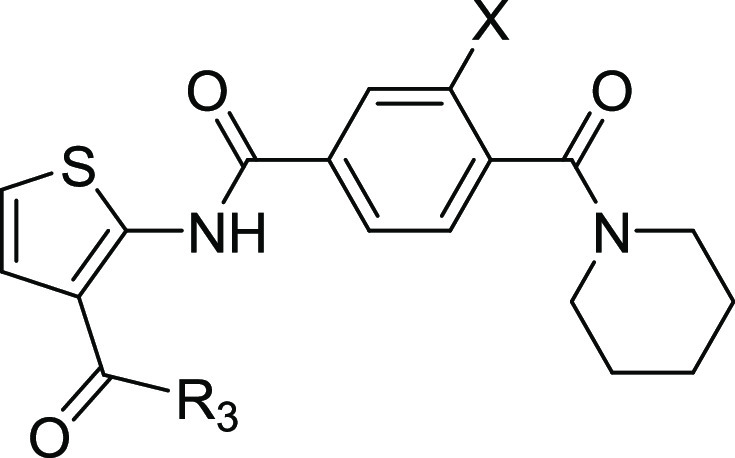

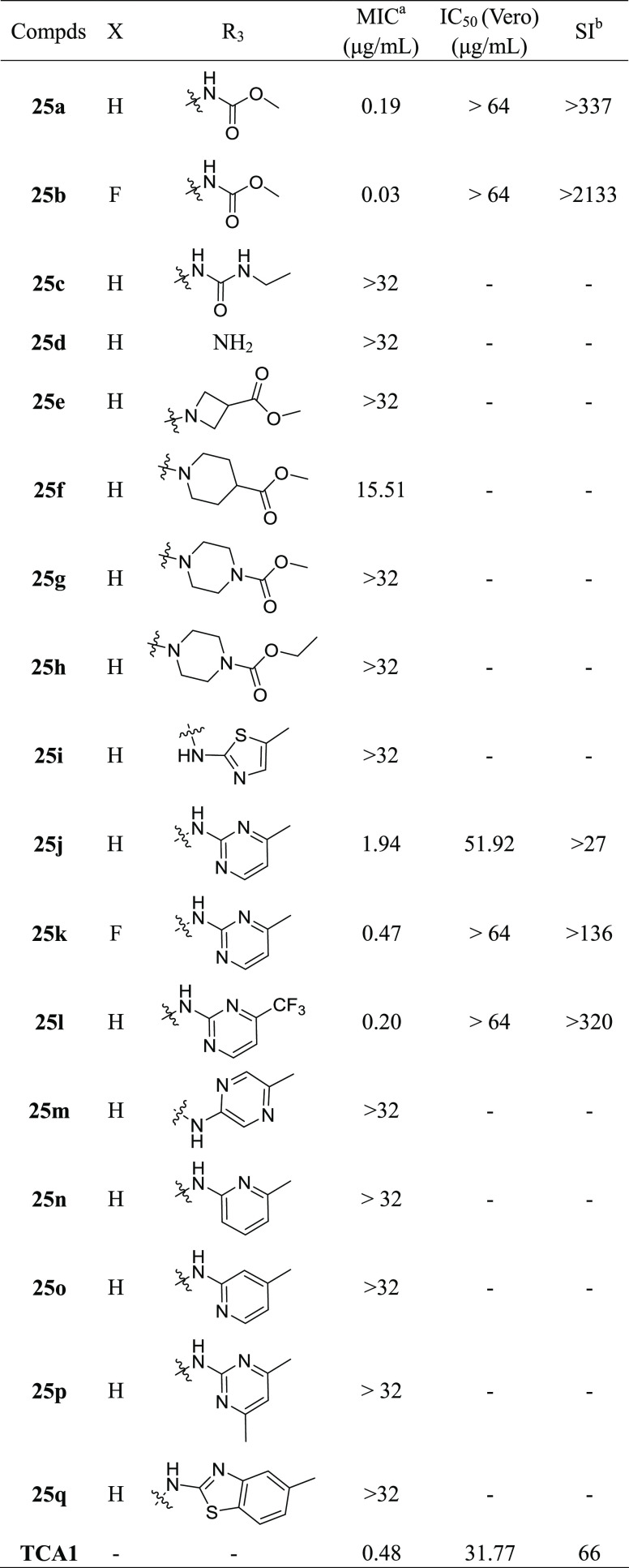
SAR of Thiophene-arylamide Compounds at X and R_3_ Sites

a MIC against *M. tuberculosis* H_37_Rv.

b SI = selectivity index, IC_50_/MIC.

### *In Vitro* Anti-XDR-TB Activity and Intracellular Antimycobacterial Activity

Considering that most thiophene-arylamide derivatives displayed potent activity against *M. tuberculosis* H_37_Rv, some representative compounds were further tested against two XDR-TB isolated clinical strains (Table 4). It is gratifying to note that the selected compounds, together with reference TCA1, demonstrated potent activities against XDR-TB strains. In particular, compounds **23j**, **24f**, and **25b** displayed very potent activity against both drug-susceptible and drug-resistant tuberculosis *in vitro*, compared to TCA1. Furthermore, we conducted an additional assay to assess the MIC against PBTZ169- and bedaquiline-resistant strains of *M. tuberculosis* for the representative compounds. The results revealed that the thiophene-arylamide derivatives showed no significant cross resistance to the covalent DprE1 inhibitor PBTZ169 and ATP synthase inhibitor bedaquiline. The results indicated that these novel thiophene-arylamide derivatives are noncovalent DprE1 inhibitors and represent promising additions in the combination regiment for the treatment of drug-resistant TB.

**Table 4:** Activity of Representative Compounds against Clinical Isolates of *M. tuberculosis*

	MIC (μg/mL)				
compounds	H_37_Rv	13946^a^	14862^b^	PBTZ169-resistant strain	bedaquiline-resistant strain
**23j**	0.055	0.082	0.059	0.14	
**24f**	0.020	0.031	0.031	0.062	0.054
**24j**	0.059	0.12	0.12		
**24k**	0.15	0.44	0.23		
**25a**	0.12	0.24	0.24	0.48	
**25b**	0.030	0.062	0.12		
**25l**	0.20	0.94	0.48		
TCA1	0.47	0.95	0.48	1.23	
INH	0.019	2.46	>10		
RFP	0.015	>10	9.24		
PBTZ169	0.0001			0.005	
bedaquiline	0.036				2.24

a Resistance to isoniazid (INH), streptomycin (SM), rifampicin (RFP), ethambutol (EMB), rifabutin (RBT), paza-aminosalicylate (PAS), and ofloxacin (OFLX).

b Resistance to INH, SM, RFP, EMB, PAS, prothionamide (1321), and capreomycin (CPM).

Since *M. tuberculosis* is an intracellular pathogen and survives in macrophages, we investigated the selected potent compounds for antimycobacterial activity in an intracellular macrophage infection model. As shown in Table 5, 0.75–1.34 log_10_ CFU reduction in macrophages was observed following treatment with selected compounds for 3 days at 10 μg/mL. Most notably, compounds **23j**, **24f**, and **25l** exhibited better intracellular activity with a reduction of ∼1 log_10_ CFU (compared to TCA1 = 0.61 log_10_ CFU) and similar to the positive control RFP at 5 μg/mL, although these compounds did not display the significant dose–response compared to the reduced colony-forming unit (CFU) data at 10 μg/mL, which may be related to either the mechanism of action of the DprE1 inhibitor as a fast bactericidal or the permeability of the cell wall to the specific compounds. The above encouraging data drove us to further explore these promising thiophene-arylamides in preliminary druggability profiles.

**Table 5:** Activity of Selected Compounds in an Intracellular Macrophage Infection Model

	log_10_ CFU/macrophages^a^			
compounds	10 μg/mL	Δlog_10_ CFU^b^	5 μg/mL	Δlog_10_ CFU^b^
**23j**	4.45 ± 0.03	1.07	4.54 ± 0.01	0.98
**24f**	4.18 ± 0.04	1.34	4.41 ± 0.10	1.11
**25a**	4.77 ± 0.10	0.75	5.06 ± 0.03	0.46
**25b**	4.59 ± 0.05	0.93	4.75 ± 0.21	0.77
**25l**	4.54 ± 0.00	0.98	4.59 ± 0.03	0.93
TCA1	4.36 ± 0.08	1.16	4.91 ± 0.19	0.61
RFP			4.49 ± 0.11	1.03
untreated	5.52 ± 0.18		5.52 ± 0.18	

a log_10_ CFU against *M. tuberculosis* (H_37_Rv) in infected mouse J774A.1 macrophages.

b Δlog_10_ CFU = log_10_ CFU (untreated) – log_10_ CFU (treated with the selected compounds).

### *In Vitro* ADME/T Assay and *In Vivo* Pharmacokinetic Property Evaluation

To identify the metabolic and toxic liabilities of our thiophene-arylamide scaffold, the selected compounds, along with the reference TCA1, were evaluated for their hepatocyte stability, cytotoxicity against mammalian HepG2 cells, and human ether-à-go-go related gene (hERG) liability. As presented in Table 6, we were pleased to find that all selected compounds exhibited no cytotoxicity against HepG2 cells with IC_50_ > 64 μg/mL. In addition, low inhibition profiles of the hERG channel (IC_50_ > 20 μM) across the series indicated a low risk of blocking the cardiac potassium channel and causing QT prolongation. Although the selected compounds showed some metabolic liability in mouse hepatocytes compared to TCA1, they displayed significant superior stability in human hepatocytes (*t*_1/2_ = 20.3–72.5 *vs* 5.97 min).

**Table 6:** Hepatocyte Stability, Cytotoxicity, and hERG Inhibition of Selected Compounds

compounds	hepatocyte stability				cytotoxicity (HepG2)	hERG K^+^
	mouse		human			
	*t*_1/2_ (min)	%remaining^a^	*t*_1/2_ (min)	%remaining^a^	IC_50_ (μg/mL)	IC_50_ (μM)
**23j**	56.4	69.1	50.8	66.4	>64	23.7
**24f**	43.7	62.1	38.1	58.0	>64	>30
**24j**	19.3	34.0	72.5	75.1	>64	28.3
**25a**	27.1	46.5	20.3	35.9	>64	22.6
**25b**	12.5	19.0	32.6	52.8	>64	>30
TCA1	85.3	74.8	5.97	3.1	46.1	18.3

a Substrate concentrations were determined in incubations after 30 min and normalized to concentrations at time zero.

Spurred on by the pronounced anti-TB activity and structural diversity, compounds **24f** and **25a** were chosen for further evaluation of their metabolic features in Balb/c mice. Pharmacokinetic (PK) studies for compounds **24f**, **25a**, and TCA1 were performed in Balb/c mice, following a single oral and an intravenous dose (Table 7). Compound **25a** exhibited high plasma exposure (area under the curve (AUC)_0–∞_ = 657 ng·h/mL) and high maximum plasma concentration (*C*_max_ = 486 ng/mL) after oral administration, compared to compound **24f** with AUC_0–∞_ = 57.9 ng·h/mL and *C*_max_ = 25.4 ng/mL. In addition, the oral bioavailability of **24f** was very low (*F* = 2.3%) and clearance was high, which was dropped off in further studies. Moreover, TCA1 produced a much higher exposure concentration than **25a** and possessed a 5-fold enhancement of oral bioavailability. Based on the results of PK profiles, compound **25a** with acceptable oral bioavailability (*F* = 7.9%) was deemed worthy of further evaluation in *in vivo* efficacy studies.

**Table 7:** Mouse PK Properties of Compounds **24f**, **25a**, and TCA1

		**24f**		**25a**		TCA1	
parameters	units	p.o.	i.v.	p.o.	i.v.	p.o.	i.v.
dose	mg/kg	50	5	50	5	50	5
*t*_1/2_^a^	h	1.70	0.13	0.85	0.26	2.19	0.47
*t*_max_	h	1.00		0.58		1.00	
*C*_max_	ng/mL	25.4	1299	486	4555	10 603	14 304
AUC_0–*t*_	ng·h/mL	41.9	183	651	830	33 715	7910
AUC_0–∞_^b^	ng·h/mL	57.9	184	657	833	33 724	7912
MRT_0–∞_^c^	h	2.10	0.13	1.10	0.20	2.87	0.39
clearance	mL/(min·kg)		479		101		10.6
*F*^d^	%	2.3		7.9		42.6	

a Plasma elimination half-life.

b Plasma exposure.

c Mean residence time.

d Oral bioavailability.

### *In vivo* Efficacy Study on Compound **25a** in a Mouse Model of TB

The *in vivo* efficacy of compound **25a** and TCA1 was conducted in Balb/c mice in an acute TB infection model. Compound **25a** and reference TCA1 were orally administered at 100 mg/kg, whereas the positive control, INH, was given at 25 mg/kg. The same formulation, 0.5% carboxymethylcellulose (CMC) in water, was used for all compounds tested. After three weeks of treatment, the mice were sacrificed and the number of colony-forming units (CFUs) in the lungs were counted and compared with those in the untreated control group. As shown in Table 8, compound **25a** showed potent *in vivo* activity, reducing the bacterial burden in the lungs by 2.02 log_10_ CFU compared with the untreated control group. In another batch, TCA1 displayed similar *in vivo* bactericidal activity compared to **25a**, which resulted in a reduction of 2.86 log_10_ CFU in the lungs. These results showed that compound **25a** exhibited similar potency even at low bioavailability compared to TCA1, which indicates that further optimization of improving the PK profiles and therefore enhancing the *in vivo* efficacy is required.

**Table 8:** *In Vivo* Efficacy of Compound **25a**

compounds	Dose (mg/kg)	log_10_ CFU/lung	log_10_ CFU/lung	Δlog_10_ CFU^a^
untreated		6.44 ± 0.34	7.10 ± 0.15	
INH	25	1.76 ± 0.49	1.93 ± 0.21	4.68/5.17
TCA1	100		4.24 ± 0.25	2.86
**25a**	100	4.42 ± 0.45		2.02

a Δlog_10_ CFU = log_10_ CFU (untreated) – log_10_ CFU (treated with selected compounds).

### Target Validation of the Mode of Action

Finally, to identify and confirm the biological target for this novel thiophene-arylamide series, the selected compounds with potent antimycobacterial activities and diversified side chains were measured against an overexpressed Mt-DprE1 in *M. bovis* BCG (Table 9). Compounds **23j**, **24f**, **24j**, **25a**, **25b**, and **25l** showed a decrease in potency against only DprE1-overexpressing strains but not against DprE2-overexpressing and wild-type strains. These compounds, as well as positive control TCA1, displayed a 4-fold higher shift in the MIC when Mt-DprE1 was overexpressed in *M. bovis* BCG. The promising results indicate that DprE1 could be the target for this novel thiophene-arylamide series.

**Table 9:** Inhibition of DprE1 and Cellular Potency by Thiophene-arylamides

compounds	MIC_OE_ (μg/mL)^a^ (DprE1)	MIC_OE_ (μg/mL)^b^ (DprE2)	MIC_WT_ (μg/mL)^c^	MIC foldshift^d^	IC_50_ DprE1 (μg/mL)^e^
**23j**	>1.28	0.32	0.32	>4	0.3 ± 0.1
**24f**	>0.51	0.13	0.064	>8	0.2 ± 0.04
**24j**	8.96	0.56	0.28	32	0.3 ± 0.1
**25a**	>12.16	1.52	0.76	>16	0.9 ± 0.2
**25b**	>1.98	0.25	0.12	>16	0.4 ± 0.1
**25l**	12.8	1.60	1.60	8	0.4 ± 0.1
**TCA1**	15.36	0.96	0.48	32	0.1 ± 0.01

a Overexpressor (OE) MICs are for *Mycobacterium bovis* bacillus Calmette-Guerin (BCG) Pasteur transformed with pMV261:Mt-*dprE*1.

b Overexpressor (OE) MICs are for *M. bovis* BCG Pasteur transformed with pMV261:Mt-*dprE2*.

c MICs against *M. bovis* BCG Pasteur strain transformed with pMV261.

d Ratio of MIC values against the DprE1-overexpressing strain and wild-type strain.

e All *in vitro* assays were performed using Mt-DprE1.

To further verify that thiophene-arylamides inhibit the catalytic activity of DprE1, representative compounds were tested against *M. tuberculosis* DprE1. Potent IC_50_ values in the range of 0.2–0.9 μg/mL were obtained, showing a good correlation with the MIC for the compounds tested. These results confirm a similar mode of action of the thiophene-arylamides described here to TCA1. Thus, our scaffold hopping design strategy has resulted in the successful identification of thiophenes containing the key arylamide moiety as potent DprE1 inhibitors.

## Conclusions

DprE1 has emerged as a promising target for the treatment of tuberculosis, and previous studies have been indicated that the inhibition of DprE1 causes loss of its ability to construct the bacterial cell wall. Based on the crystal structure of the TCA1–DprE1 complex, we have reported the design, synthesis, and SAR study of a series of novel thiophene-arylamide compounds. Molecular docking studies of template compound **23j** with DprE1 indicated that the hydrogen bond interaction with Tyr60 provided additional binding affinity in reinforcing the interaction of thiophene containing the critical benzamide moiety. Subsequent scaffold hopping from the benzothiazole to arylamide moiety led to a new series of lead compounds with improved antimycobacterial activity and low cytotoxicity. In particular, the representative compounds displayed very potent activity against both drug-susceptible and drug-resistant tuberculosis compared to the target compound TCA1. In addition, the selected compounds also displayed good inhibition of intracellular TB growth in infected macrophages. Furthermore, the preliminary druggability study demonstrated that the selected compounds exhibited good hepatocyte stability and low hERG liability. Further biological studies revealed that these novel thiophene-arylamide compounds targeted DprE1, like representative compounds **23j**, **24f**, **25a**, and **25b** directly bound to DprE1, and displayed good to excellent DprE1 inhibition. Importantly, compound **25a** with acceptable PK profiles demonstrated significant efficacy *in vivo* in an acute mouse model of TB. Our efforts are ongoing to improve the druggability profiles of this series of thiophene-arylamide compounds by maintaining good antituberculosis activity with the aim of developing more promising candidates as anti-TB agents targeting DprE1.

## Experimental Section

### Chemistry

All reagents and solvents were purchased from commercial suppliers and used without further purification. Reactions were monitored by thin-layer chromatography (TLC) with visualization of components by UV light (254 nm) or exposure to I_2_. Flash column chromatography was conducted on silica gel (300–400 mesh). Melting points were determined on a Yanaco MP-J3 microscope melting point apparatus, which is uncorrected. ^1^H NMR and ^13^C NMR spectra were recorded on Varian-400 and Mercury-500/600 spectrometers in CDCl_3_ or dimethyl sulfoxide (DMSO)-*d*_6_. Electrospray ionization-high-resolution mass spectrometry (ESI-HRMS) data were measured on a Thermo Exactive Orbitrap plus spectrometer.

All target compounds were purified by chromatography and have a purity of ≥95% as determined by high-performance liquid chromatography (HPLC)/MS analysis conducted on a Thermo Exactive Plus system using a reversed-phase C18 column with 5–95% CH_3_CN in water (0.1% HCOOH) for 5 min at a flow rate of 0.4 mL/min.

#### General Procedure for the Synthesis of Intermediates **3a**–**p**

To a solution of methyl 4-(chlorocarbonyl)benzoate **1** (1 equiv) in anhydrous CH_2_Cl_2_ were added the corresponding amines **2a**–**p** (1.5 equiv) and Et_3_N (5 equiv) cooled with an ice bath. The reaction mixture was stirred at room temperature for 3 h under argon, then quenched with water, and extracted with CH_2_Cl_2_ twice. The combined organic phase was washed with 1 N HCl, H_2_O, saturated NaHCO_3_, and brine in turn. The obtained organic phase was dried over anhydrous Na_2_SO_4_, filtered, and evaporated *in vacuo* to give intermediates **3a**–**p**.

##### Methyl 4-(Propylcarbamoyl)benzoate (**3a**)

White solid; yield 93%; mp 101–102 °C. ^1^H NMR (400 MHz, CDCl_3_) δ: 8.09 (d, *J* = 8.4 Hz, 2H), 7.82 (d, *J* = 8.4 Hz, 2H), 3.94 (s, 3H), 3.46–3.41 (m, 2H), 1.67–1.65 (m, 2H), 1.00 (t, *J* = 7.6 Hz, 3H). MS (ESI): *m*/*z* 222.11 (M + H)^+^.

##### Methyl 4-(Ethyl(methyl)carbamoyl)benzoate (**3b**)

White solid; yield 92%; mp 50–51 °C. ^1^H NMR (400 MHz, CDCl_3_) δ: 8.08 (d, *J* = 8.4 Hz, 2H), 7.46 (d, *J* = 8.4 Hz, 2H), 3.94 (s, 3H), 3.24–2.91 (m, 2H), 1.26–1.23 (m, 3H). MS (ESI): *m*/*z* 222.11 (M + H)^+^.

##### Methyl 4-(Diethylcarbamoyl)benzoate (**3c**)

White solid; yield 94%; mp 154–155 °C. ^1^H NMR (400 MHz, CDCl_3_) δ: 8.08 (d, *J* = 8.4 Hz, 2H), 7.44 (d, *J* = 8.4 Hz, 2H), 3.94 (s, 3H), 3.56 (brs, 2H), 3.22 (brs, 2H), 1.26 (brs, 3H), 1.10 (brs, 3H). MS (ESI): *m*/*z* 236.13 (M + H)^+^.

##### Methyl 4-(((3*S*,5*S*,7*S*)-Adamantan-1-yl)carbamoyl)benzoate (**3d**)

White solid; yield 98%; mp 182–183 °C. ^1^H NMR (400 MHz, CDCl_3_) δ: 8.07 (d, *J* = 8.4 Hz, 2H), 7.77 (d, *J* = 8.4 Hz, 2H), 3.94 (s, 3H), 2.13 (brs, 9H), 1.73 (brs, 6H). MS (ESI): *m*/*z* 314.17 (M + H)^+^.

##### Methyl 4-(Cyclohexylcarbamoyl)benzoate (**3e**)

White solid; yield 91%; mp 199–200 °C. ^1^H NMR (500 MHz, CDCl_3_) δ: 8.09 (d, *J* = 8.0 Hz, 2H), 7.80 (d, *J* = 8.0 Hz, 2H), 6.00 (brs, 1H), 3.98 (m, 1H), 3.94 (s, 3H), 2.06–2.03 (m, 2H), 1.78–1.75 (m, 2H), 1.68–1.66 (m, 1H), 1.45–1.40 (m, 2H), 1.29–1.24 (m, 3H). MS (ESI): *m*/*z* 262.14 (M + H)^+^.

##### Methyl 4-((4,4-Dimethylcyclohexyl)carbamoyl)benzoate (**3f**)

White solid; yield 95%; mp 106–107 °C. ^1^H NMR (400 MHz, CDCl_3_) δ: 8.09 (d, *J* = 8.8 Hz, 2H), 7.81 (d, *J* = 8.8 Hz, 2H), 6.06–6.04 (m, 1H), 3.94 (m, 4H), 1.92–1.88 (m, 2H), 1.46–1.39 (m, 6H), 0.95 (s, 6H). MS (ESI): *m*/*z* 290.18 (M + H)^+^.

##### Methyl 4-(Phenylcarbamoyl)benzoate (**3g**)

White solid; yield 91%; mp 189–190 °C. ^1^H NMR (400 MHz, DMSO-*d*_6_) δ: 10.45 (brs, 1H), 8.11–8.06 (m, 4H), 7.80–7.77 (m, 2H), 7.39–7.35 (m, 2H), 7.14–7.10 (m, 1H), 3.90 (s, 3H). MS (ESI): *m*/*z* 256.10 (M + H)^+^.

##### Methyl 4-(Azetidine-1-carbonyl)benzoate (**3h**)

White solid; yield 94%; mp 88–89 °C. ^1^H NMR (400 MHz, CDCl_3_) δ: 8.07 (d, *J* = 8.8 Hz, 2H), 7.68 (d, *J* = 8.8 Hz, 2H), 4.31–4.22 (m, 4H), 3.94 (s, 3H), 2.40–2.33 (m, 2H). MS (ESI): *m*/*z* 220.09 (M + H)^+^.

##### Methyl 4-(Pyrrolidine-1-carbonyl)benzoate (**3i**)

White solid; yield 95%; mp 103–104 °C. ^1^H NMR (400 MHz, CDCl_3_) δ: 8.07 (d, *J* = 8.4 Hz, 2H), 7.57 (d, *J* = 8.4 Hz, 2H), 3.94 (s, 3H), 3.64 (brs, 2H), 3.40 (brs, 2H) 1.93 (brs, 4H). MS (ESI): *m*/*z* 234.11 (M + H)^+^.

##### Methyl 4-(Piperidine-1-carbonyl)benzoate (**3j**)

White solid; yield 92%; mp 127–128 °C. ^1^H NMR (500 MHz, CDCl_3_) δ: 8.07 (d, *J* = 8.0 Hz, 2H), 7.45 (d, *J* = 8.0 Hz, 2H), 3.93 (s, 3H), 3.72 (brs, 2H), 3.29 (brs, 2H), 1.69 (brs, 4H), 1.52 (brs, 2H). MS (ESI): *m*/*z* 248.13 (M + H)^+^.

##### Methyl 4-(Azepane-1-carbonyl)benzoate (**3k**)

Colorless oil; yield 99%; ^1^H NMR (400 MHz, CDCl_3_) δ: 8.07 (d, *J* = 8.4 Hz, 2H), 7.44 (d, *J* = 8.4 Hz, 2H), 3.93 (s, 3H), 3.69 (t, *J* = 6.0 Hz, 2H), 3.32 (brs, 2H), 1.88–1.82 (m, 2H), 1.65 (brs, 2H), 1.60 (brs, 4H). MS (ESI): *m*/*z* 262.14 (M + H)^+^.

##### Methyl 4-(4-Methylpiperidine-1-carbonyl)benzoate (**3l**)

Colorless oil; yield 95%; ^1^H NMR (400 MHz, CDCl_3_) δ: 8.07 (d, *J* = 8.4 Hz, 2H), 7.45 (d, *J* = 8.4 Hz, 2H), 4.69–4.67 (m, 1H), 3.93 (s, 3H), 3.62–3.59 (m, 1H), 2.99 (brs, 1H), 2.78 (brs, 1H), 1.76 (brs, 1H), 1.72–1.61 (m, 2H), 1.26–1.07 (m, 2H), 0.98 (d, *J* = 6.4 Hz, 3H). MS (ESI): *m*/*z* 262.14 (M + H)^+^.

##### Methyl 4-(4-Methoxypiperidine-1-carbonyl)benzoate (**3m**)

Light yellow oil; yield 99%; ^1^H NMR (500 MHz, CDCl_3_) δ: 8.08 (d, *J* = 8.0 Hz, 2H), 7.45 (d, *J* = 8.0 Hz, 2H), 4.01 (brs, 1H), 3.94 (s, 3H), 3.55 (brs, 2H), 3.50–3.49 (m, 1H), 3.37 (s, 3H), 3.18 (brs, 1H), 1.95 (brs, 1H), 1.78–1.72 (m, 2H), 1.56 (brs, 1H). MS (ESI): *m*/*z* 278.14 (M + H)^+^.

##### Methyl 4-(4,4-Difluoropiperidine-1-carbonyl)benzoate (**3n**)

White solid; yield 97%; mp 83–84 °C. ^1^H NMR (400 MHz, CDCl_3_) δ: 8.11 (d, *J* = 8.4 Hz, 2H), 7.48 (d, *J* = 8.4 Hz, 2H), 3.95 (s, 3H), 3.88 (brs, 2H), 3.51 (brs, 2H), 2.45–2.33 (m, 1H), 2.08–1.95 (m, 3H). MS (ESI): *m*/*z* 284.11 (M + H)^+^.

##### Methyl 4-(Morpholine-4-carbonyl)benzoate (**3o**)

White solid; yield 98%; mp 79–80 °C.^1^H NMR (400 MHz, CDCl_3_) δ: 8.07 (d, *J* = 8.4 Hz, 2H), 7.46 (d, *J* = 8.4 Hz, 2H), 3.92 (s, 3H), 3.81–3.74 (m, 4H), 3.61 (brs, 2H), 3.38 (brs, 2H). MS (ESI): *m*/*z* 250.11 (M + H)^+^.

##### Methyl 4-(Thiomorpholine-4-carbonyl)benzoate (**3p**)

White solid; yield 93%; mp 101–102 °C. ^1^H NMR (500 MHz, CDCl_3_) δ: 8.09 (d, *J* = 8.0 Hz, 2H), 7.44 (d, *J* = 8.0 Hz, 2H), 4.04 (brs, 2H) 3.94 (s, 3H), 3.62 (brs, 2H), 2.75 (brs, 2H), 2.56 (brs, 2H). MS (ESI): *m*/*z* 266.08 (M + H)^+^.

#### General Procedure for the Synthesis of Intermediates **4a**–**p**

To a solution of **3a**–**p** (1 equiv) in CH_3_OH was added 1 mol/L LiOH aqueous solution (2 equiv). The reaction mixture was stirred at room temperature for 3 h and then evaporated *in vacuo*. The residue was diluted with H_2_O, and the aqueous solution was acidified with 6 N HCl to pH 6–7. The precipitated solid was filtered to afford intermediates **4a**–**p**.

##### 4-(Propylcarbamoyl)benzoic Acid (**4a**)

White solid; yield 91%; mp 233–234 °C. ^1^H NMR (400 MHz, DMSO-*d*_6_) δ: 13.18 (brs, 1H), 8.61 (brs, 1H), 8.00 (d, *J* = 8.4 Hz, 2H), 7.92 (d, *J* = 8.4 Hz, 2H), 3.25–3.20 (m, 2H), 1.58–1.49 (m, 2H), 0.89 (t, *J* = 7.6 Hz, 3H). MS (ESI): *m*/*z* 206.08 (M – H)^−^.

##### 4-(Ethyl(methyl)carbamoyl)benzoic Acid (**4b**)

White solid; yield 46%; mp 177–178 °C. ^1^H NMR (400 MHz, DMSO-*d*_6_) δ: 13.11 (brs, 1H), 8.00–7.97 (m, 2H), 7.49–7.46 (m, 2H), 3.49–3.45 (m, 1H), 3.18–3.13 (m, 1H), 2.96–2.84 (m, 3H), 1.14–1.03 (m, 3H). MS (ESI): *m*/*z* 206.08 (M – H)^−^.

##### 4-(Diethylcarbamoyl)benzoic Acid (**4c**)

White solid; yield 56%; mp 154–155 °C. ^1^H NMR (500 MHz, DMSO-*d*_6_) δ: 13.10 (brs, 1H), 7.99 (d, *J* = 8.0 Hz, 2H), 7.45 (d, *J* = 8.0 Hz, 2H), 3.44 (brs, 2H), 3.14 (brs, 2H), 1.15 (brs, 3H), 1.03 (brs, 3H). MS (ESI): *m*/*z* 220.10 (M – H)^−^.

##### 4-(((3*S*,5*S*,7*S*)-Adamantan-1-yl)carbamoyl)benzoic Acid (**4d**)

White solid; yield 93%; mp 245–246 °C. ^1^H NMR (400 MHz, DMSO-*d*_6_) δ: 13.18 (brs, 1H), 7.96 (d, *J* = 8.4 Hz, 2H), 7.85 (d, *J* = 8.4 Hz, 2H), 7.80 (brs, 1H), 2.07 (brs, 9H), 1.65 (brs, 6H). MS (ESI): *m*/*z* 298.15 (M – H)^−^.

##### 4-(Cyclohexylcarbamoyl)benzoic Acid (**4e**)

White solid; yield 97%; mp > 250 °C. ^1^H NMR (500 MHz, DMSO-*d*_6_) δ: 13.16 (brs, 1H), 8.37 (d, *J* = 8.0 Hz, 1H), 7.99 (d, *J* = 8.0 Hz, 2H), 7.92 (d, *J* = 8.0 Hz, 2H), 3.76 (brs, 1H), 1.81 (brs, 2H), 1.74 (brs, 2H), 1.62–1.59 (m, 1H), 1.31–1.23 (m, 4H), 1.13–1.12 (m, 1H). MS (ESI): *m*/*z* 246.12 (M – H)^−^.

##### 4-((4,4-Dimethylcyclohexyl)carbamoyl)benzoic Acid (**4f**)

White solid; yield 90%; mp > 250 °C. ^1^H NMR (400 MHz, DMSO-*d*_6_) δ: 8.38 (d, *J* = 8.0 Hz, 1H), 7.99 (d, *J* = 8.4 Hz, 2H), 7.93 (d, *J* = 8.4 Hz, 2H), 3.77–3.67 (m, 1H), 1.65–1.61 (m, 2H), 1.56–1.52 (m, 2H), 1.41–1.38 (m, 2H), 1.29–1.23 (m, 2H), 0.93 (brs, 3H), 0.92 (brs, 3H). MS (ESI): *m*/*z* 274.15 (M – H)^−^.

##### 4-(Phenylcarbamoyl)benzoic Acid (**4g**)

White solid; yield 72%; mp > 250 °C. ^1^H NMR (400 MHz, DMSO-*d*_6_) δ: 13.25 (brs, 1H), 10.42 (brs, 1H),8.09–8.04 (m, 4H), 7.90 (d, *J* = 8.0 Hz, 2H), 7.36 (t, *J* = 8.0 Hz, 2H), 7.12 (t, *J* = 7.4 Hz, 1H). MS (ESI): *m*/*z* 240.07 (M – H)^−^.

##### 4-(Azetidine-1-carbonyl)benzoic Acid (**4h**)

White solid; yield 78%; mp 213–215 °C. ^1^H NMR (400 MHz, DMSO-*d*_6_) δ: 13.19 (brs, 1H), 7.90 (d, *J* = 8.4 Hz, 2H), 7.10 (d, *J* = 8.4 Hz, 2H), 4.27 (t, *J* = 7.6 Hz, 2H), 4.05 (t, *J* = 7.6 Hz, 2H), 2.29–2.21 (m, 2H). MS (ESI): *m*/*z* 204.07 (M – H)^−^.

##### 4-(Pyrrolidine-1-carbonyl)benzoic Acid (**4i**)

White solid; yield 89%; mp 219–220 °C. ^1^H NMR (400 MHz, DMSO-*d*_6_) δ: 13.14 (brs, 1H), 7.98 (d, *J* = 8.4 Hz, 2H), 7.60 (d, *J* = 8.4 Hz, 2H), 3.47 (t, *J* = 6.8 Hz, 2H), 3.33 (t, *J* = 6.4 Hz, 2H), 1.88–1.79 (m, 4H). MS (ESI): *m*/*z* 218.08 (M – H)^−^.

##### 4-(Piperidine-1-carbonyl)benzoic Acid (**4j**)

White solid; yield 89%; mp 240–241 °C. ^1^H NMR (500 MHz, DMSO-*d*_6_) δ: 13.11 (brs, 1H), 7.98 (d, *J* = 8.0 Hz, 2H), 7.47 (d, *J* = 8.0 Hz, 2H), 3.59 (brs, 2H), 3.21 (brs, 2H), 1.60–1.56 (m, 4H), 1.44 (brs, 2H). MS (ESI): *m*/*z* 232.10 (M – H)^−^.

##### 4-(Azepane-1-carbonyl)benzoic Acid (**4k**)

White solid; yield 92%; mp 238–239 °C. ^1^H NMR (400 MHz, DMSO-*d*_6_) δ: 13.10 (brs, 1H), 7.98 (d, *J* = 8.4 Hz, 2H), 7.46 (d, *J* = 8.4 Hz, 2H), 3.56 (t, *J* = 6.0 Hz, 2H), 3.25 (t, *J* = 5.6 Hz, 2H), 1.73–1.70 (m, 2H), 1.58–1.55 (m, 2H), 1.64–1.48 (m, 4H). MS (ESI): *m*/*z* 246.11 (M – H)^−^.

##### 4-(4-Methylpiperidine-1-carbonyl)benzoic Acid (**4l**)

White solid; yield 92%; mp 196–197 °C. ^1^H NMR (500 MHz, DMSO-*d*_6_) δ: 13.12 (brs, 1H), 7.98 (d, *J* = 8.0 Hz, 2H), 7.47 (d, *J* = 8.0 Hz, 2H), 4.45–4.43 (m, 1H), 3.45–3.43 (m, 1H), 3.00 (brs, 1H), 2.76 (brs, 1H), 1.69–1.53 (m, 3H), 1.08–1.05 (m, 2H), 0.92 (d, *J* = 6.0 Hz, 3H). MS (ESI): *m*/*z* 246.11 (M – H)^−^.

##### 4-(4-Methoxypiperidine-1-carbonyl)benzoic Acid (**4m**)

White solid; yield 49%. mp 184–185 °C. ^1^H NMR (400 MHz, DMSO-*d*_6_) δ: 13.13 (brs, 1H), 7.98 (d, *J* = 8.4 Hz, 2H), 7.49 (d, *J* = 8.4 Hz, 2H), 3.91 (brs, 1H), 3.45–3.42 (m, 3H), 3.25 (s, 3H), 3.11 (brs, 1H), 1.87–1.77 (m, 2H), 1.47–1.40 (m, 2H). MS (ESI): *m*/*z* 262.11 (M – H)^−^.

##### 4-(4,4-Difluoropiperidine-1-carbonyl)benzoic Acid (**4n**)

White solid; yield 83%; mp 228–229 °C. ^1^H NMR (400 MHz, DMSO-*d*_6_) δ: 13.14 (brs, 1H), 8.00 (d, *J* = 8.4 Hz, 2H), 7.56 (d, *J* = 8.4 Hz, 2H), 3.73 (brs, 2H), 3.37 (brs, 2H), 2.03 (brs, 4H). MS (ESI): *m*/*z* 268.08 (M – H)^−^.

##### 4-(Morpholine-4-carbonyl)benzoic Acid (**4o**)

White solid; yield 76%; mp 194–195 °C. ^1^H NMR (400 MHz, DMSO-*d*_6_) δ: 13.16 (brs, 1H), 7.99 (d, *J* = 8.4 Hz, 2H), 7.52 (d, *J* = 8.4 Hz, 2H), 3.64 (brs, 4H), 3.54 (brs, 2H), 3.29 (brs, 2H). MS (ESI): *m*/*z* 234.08 (M – H)^−^.

##### 4-(Thiomorpholine-4-carbonyl)benzoic Acid (**4p**)

White solid; yield 82%; mp > 250 °C. ^1^H NMR (400 MHz, DMSO-*d*_6_) δ: 13.14 (brs, 1H), 7.99 (d, *J* = 8.4 Hz, 2H), 7.50 (d, *J* = 8.4 Hz, 2H), 3.87 (brs, 2H), 3.49 (brs, 2H), 2.69 (brs, 2H), 2.59 (brs, 2H). MS (ESI): *m*/*z* 250.05 (M – H)^−^.

#### General Procedure for the Synthesis of Intermediates **6a**–**l**

To a solution of aryl carboxylic acids **5a**–**l** (1 equiv) in DMF were added HATU (2 equiv), piperidine (1.5 equiv), and Et_3_N (3 equiv). The reaction mixture was stirred at room temperature for 10 h and then concentrated *in vacuo*. The residue was diluted with CH_2_Cl_2_, washed with water and brine, dried over anhydrous Na_2_SO_4_, filtered, and evaporated *in vacuo*. The residue was purified by silica gel column chromatography (petroleum ether (PE)/ethyl acetate (EA) = 100/30) to afford intermediates **6a**–**l**.

##### Methyl 3-(Piperidine-1-carbonyl)benzoate (**6a**)

Colorless oil; yield 63%. ^1^H NMR (500 MHz, CDCl_3_) δ: 8.07 (brs, 2H), 7.60–7.59 (m, 1H), 7.51–7.48 (m, 1H), 3.93 (s, 3H), 3.72 (brs, 2H), 3.33 (brs, 2H), 1.69 (brs, 4H), 1.53 (brs, 2H).

##### Methyl 2-Fluoro-4-(piperidine-1-carbonyl)benzoate (**6b**)

White solid; yield 90%;. ^1^H NMR (400 MHz, CDCl_3_) δ: 7.98 (t, *J* = 7.6 Hz, 1H), 7.21 (dd, *J*_1_ = 8.0 Hz, *J*_2_ = 1.6 Hz, 1H), 7.17 (dd, *J*_1_ = 10.8 Hz, *J*_2_ = 1.6 Hz, 1H), 3.95 (s, 3H), 3.70 (brs, 2H), 3.29 (brs, 2H), 1.69 (brs, 4H), 1.53 (brs, 2H).

##### Methyl 2-Chloro-4-(piperidine-1-carbonyl)benzoate (**6c**)

Yellow oil; yield 95%. ^1^H NMR (400 MHz, CDCl_3_) δ: 7.84 (d, *J* = 8.0 Hz, 1H), 7.45 (d, *J* = 1.6 Hz, 1H), 7.29 (dd, *J*_1_ = 8.0 Hz, *J*_2_ = 1.6 Hz, 1H), 3.93 (s, 3H), 3.68 (brs, 2H), 3.28 (brs, 2H), 1.67 (brs, 4H), 1.50 (brs, 2H). MS (ESI): *m*/*z* 282.09 (M + H)^+^.

##### Methyl 2-Bromo-4-(piperidine-1-carbonyl)benzoate (**6d**)

Colorless oil; yield 75%. ^1^H NMR (400 MHz, CDCl_3_) δ: 7.82 (d, *J* = 7.6 Hz, 1H), 7.68 (d, *J* = 1.6 Hz, 1H), 7.36 (dd, *J*_1_ = 8.0 Hz, *J*_2_ = 1.6 Hz, 1H), 3.95 (s, 3H), 3.70 (brs, 2H), 3.30 (brs, 2H), 1.69 (brs, 4H), 1.53 (brs, 2H). MS (ESI): *m*/*z* 326.04 (M + H)^+^.

##### Methyl 2-Nitro-4-(piperidine-1-carbonyl)benzoate (**6e**)

White solid; yield 97%; mp 73–74 °C. ^1^H NMR (400 MHz, CDCl_3_) δ: 7.94–7.93 (m, 1H), 7.79 (d, *J* = 8.0 Hz, 1H), 7.70 (dd, *J*_1_ = 8.0 Hz, *J*_2_ = 1.6 Hz, 1H), 3.94 (s, 3H), 3.73 (brs, 2H), 3.33 (brs, 2H), 1.71 (brs, 4H), 1.62–1.56 (m, 2H). MS (ESI): *m*/*z* 293.11 (M + H)^+^.

##### Methyl 3-Fluoro-4-(piperidine-1-carbonyl)benzoate (**6f**)

White solid; yield 93%; mp 64–65 °C. ^1^H NMR (400 MHz, CDCl_3_) δ: 7.88 (dd, *J*_1_ = 8.0 Hz, *J*_2_ = 1.6 Hz, 1H), 7.76 (dd, *J*_1_ = 9.6 Hz, *J*_2_ = 1.6 Hz, 1H), 7.43 (dd, *J*_1_ = 8.0 Hz, *J*_2_ = 2.4 Hz, 1H), 3.94 (s, 3H), 3.74 (brs, 2H), 3.23 (brs, 2H), 1.69–1.67 (m, 4H), 1.53 (brs, 2H). MS (ESI): *m*/*z* 266.12 (M + H)^+^.

##### Methyl 3-Methyl-4-(piperidine-1-carbonyl)benzoate (**6g**)

White solid; yield 90%; mp 77–78 °C. ^1^H NMR (400 MHz, CDCl_3_) δ: 7.90 (brs, 1H), 7.88 (dd, *J*_1_ = 8.0 Hz, *J*_2_ = 1.6 Hz, 1H), 7.23 (d, *J* = 8.0 Hz, 1H), 3.92 (s, 3H), 3.83–3.80 (m, 1H), 3.71–3.68 (m, 1H), 3.15–3.12 (m, 2H), 2.36 (s, 3H), 1.67 (brs, 4H), 1.47 (brs, 2H). MS (ESI): *m*/*z* 262.14 (M + H)^+^.

##### Methyl 6-(Piperidine-1-carbonyl)-2-naphthoate (**6h**)

White solid; yield 79%; mp 127–128 °C. ^1^H NMR (400 MHz, CDCl_3_) δ: 8.61 (s, 1H), 8.10 (dd, *J*_1_ = 8.0 Hz, *J*_2_ = 1.6 Hz, 1H), 7.98 (d, *J* = 8.0 Hz, 1H), 7.92–7.90 (m, 2H), 7.54 (dd, *J*_1_ = 8.0 Hz, *J*_2_ = 1.6 Hz, 1H), 3.99 (s, 3H), 3.81 (brs, 2H), 3.39 (brs, 2H), 1.71 (brs, 4H), 1.56 (brs, 2H). MS (ESI): *m*/*z* 298.14 (M + H)^+^.

##### Methyl 4′-(Piperidine-1-carbonyl)-[1,1′-biphenyl]-4-carboxylate (**6i**)

White solid; yield 91%; mp 114–115 °C. ^1^H NMR (400 MHz, DMSO-*d*_6_) δ: 8.05 (d, *J* = 7.6 Hz, 2H), 7.87 (d, *J* = 7.6 Hz, 2H), 7.81 (d, *J* = 7.6 Hz, 2H), 7.49 (d, *J* = 7.6 Hz, 2H), 3.88 (s, 3H), 3.60 (brs, 2H), 3.26 (brs, 2H), 1.62 (brs, 2H), 1.53 (brs, 4H). MS (ESI): *m*/*z* 324.16 (M + H)^+^.

##### Methyl 5-(Piperidine-1-carbonyl)thiophene-2-carboxylate (**6j**)

White solid; yield 92%; mp 83–84 °C. ^1^H NMR (400 MHz, CDCl_3_) δ: 7.70 (dd, *J*_1_ = 8.0 Hz, *J*_2_ = 1.2 Hz, 1H), 7.20 (dd, *J*_1_ = 8.0 Hz, *J*_2_ = 1.2 Hz, 1H), 3.91 (s, 3H), 3.64 (brs, 4H), 1.71–1.70 (brs, 2H), 1.65–1.64 (brs, 4H). MS (ESI): *m*/*z* 254.08 (M + H)^+^.

##### Methyl 5-(Piperidine-1-carbonyl)furan-3-carboxylate (**6k**)

White solid; yield 74%; mp 79–80 °C. ^1^H NMR (400 MHz, CDCl_3_) δ: 8.03 (s, 1H), 7.15 (s, 1H), 3.86 (s, 3H), 3.69–3.66 (m, 4H), 1.71–1.69 (m, 2H), 1.67–1.63 (m, 4H). MS (ESI): *m*/*z* 238.11 (M + H)^+^.

##### Methyl 4-(Piperidine-1-carbonyl)-1*H*-pyrrole-2-carboxylate (**6l**)

White solid; yield 53%; mp 105–106 °C. ^1^H NMR (400 MHz, CDCl_3_) δ: 9.87 (brs, 1H), 6.87 (dd, *J*_1_ = 4.0 Hz, *J*_2_ = 2.8 Hz, 1H), 6.46 (dd, *J*_1_ = 4.0 Hz, *J*_2_ = 2.8 Hz, 1H), 3.87 (s, 3H), 3.74 (brs, 4H), 1.72–1.69 (m, 2H), 1.67–1.63 (m, 4H). MS (ESI): *m*/*z* 237.12 (M + H)^+^.

#### General Procedure for the Synthesis of Intermediates **7a**–**l**

Compounds **7a**–**l** were prepared from **6a**–**l** in the same manner as described for **4a**–**p**.

##### 3-(Piperidine-1-carbonyl)benzoic Acid (**7a**)

White solid; yield 59%; mp 123–124 °C. ^1^H NMR (500 MHz, DMSO-*d*_6_) δ: 13.16 (brs, 1H), 7.99 (brs, 1H), 7.87 (brs, 1H), 7.61–7.57 (m, 2H), 3.59 (brs, 2H), 3.25 (brs, 2H), 1.61–1.56 (m, 4H), 1.46 (brs, 2H). MS (ESI): *m*/*z* 232.08 (M – H)^−^.

##### 2-Fluoro-4-(piperidine-1-carbonyl)benzoic Acid (**7b**)

White solid; yield 97%; mp 202–203 °C. ^1^H NMR (400 MHz, DMSO-*d*_6_) δ: 13.41 (brs, 1H), 7.91 (dd, *J*_1_ = 7.6 Hz, *J*_2_ = 1.2 Hz, 1H), 7.73 (dd, *J*_1_ = 9.6 Hz, *J*_2_ = 1.2 Hz, 1H), 7.27 (dd, *J*_1_ = 7.6 Hz, *J*_2_ = 2.8 Hz, 1H), 3.58 (brs, 2H), 3.21 (m, 2H), 1.61–1.56 (m, 4H), 1.45 (brs, 2H). MS (ESI): *m*/*z* 250.11 (M – H)^−^.

##### 2-Chloro-4-(piperidine-1-carbonyl)benzoic Acid (**7c**)

White solid; yield 70%; mp 162–163 °C. ^1^H NMR (400 MHz, DMSO-*d*_6_) δ: 13.53 (brs, 1H), 7.83 (d, *J* = 7.6 Hz, 1H), 7.53 (d, *J* = 1.6 Hz, 1H), 7.40 (dd, *J*_1_ = 8.0 Hz, *J*_2_ = 1.6 Hz, 1H), 3.57 (brs, 2H), 3.22 (brs, 2H), 1.60–1.56 (m, 4H), 1.45 (brs, 2H). MS (ESI): *m*/*z* 266.06 (M – H)^−^.

##### 2-Bromo-4-(piperidine-1-carbonyl)benzoic Acid (**7d**)

White solid; yield 64%; mp 175–176 °C. ^1^H NMR (400 MHz, DMSO-*d*_6_) δ: 13.50 (brs, 1H), 7.74 (d, *J* = 8.0 Hz, 1H), 7.64 (d, *J* = 1.2 Hz, 1H), 7.40 (dd, *J*_1_ = 8.0 Hz, *J*_2_ = 1.2 Hz, 1H), 3.53 (brs, 2H), 3.19 (brs, 2H), 1.57–1.50 (m, 4H), 1.42 (brs, 2H). MS (ESI): *m*/*z* 310.01 (M – H)^−^.

##### 2-Nitro-4-(piperidine-1-carbonyl)benzoic Acid (**7e**)

White solid; yield 88%; mp 90–91 °C. ^1^H NMR (400 MHz, DMSO-*d*_6_) δ: 7.99 (d, *J* = 1.2 Hz, 1H), 7.91 (d, *J* = 7.6 Hz, 1H), 7.72 (dd, *J*_1_ = 7.6 Hz, *J*_2_ = 1.2 Hz, 1H), 3.59 (brs, 2H), 3.23 (brs, 2H), 1.59 (brs, 4H), 1.47 (brs, 2H). MS (ESI): *m*/*z* 277.09 (M – H)^−^.

##### 3-Fluoro-4-(piperidine-1-carbonyl)benzoic Acid (**7f**)

White solid; yield 98%; mp 202–203 °C. ^1^H NMR (400 MHz, DMSO-*d*_6_) δ: 13.38 (brs, 1H), 7.88 (dd, *J*_1_ = 7.6 Hz, *J*_2_ = 1.2 Hz, 1H), 7.69 (dd, *J*_1_ = 9.6 Hz, *J*_2_ = 1.2 Hz, 1H), 7.47 (dd, *J*_1_ = 7.6 Hz, *J*_2_ = 2.8 Hz, 1H), 3.59–3.56 (m, 2H), 3.13–3.11 (m, 2H), 1.58–1.51 (m, 4H), 1.42–1.37 (m, 2H). MS (ESI): *m*/*z* 250.09 (M – H)^−^.

##### 3-Methyl-4-(piperidine-1-carbonyl)benzoic Acid (**7g**)

White solid; yield 82%; mp 146–147 °C. ^1^H NMR (500 MHz, DMSO-*d*_6_) δ: 13.00 (brs, 1H), 7.83 (brs, 1H), 7.78 (d, *J* = 8.0 Hz, 1H), 7.26 (d, *J* = 8.0 Hz, 1H), 3.67–3.57 (m, 2H), 3.06–3.05 (m, 2H), 2.25 (s, 3H), 1.60–1.55 (m, 4H), 1.40–1.37 (m, 2H). MS (ESI): *m*/*z* 246.10 (M – H)^−^.

##### 6-(Piperidine-1-carbonyl)-2-naphthoic Acid (**7h**)

White solid; yield 91%; mp 181–182 °C. ^1^H NMR (500 MHz, DMSO-*d*_6_) δ: 13.19 (brs, 1H), 8.64 (s, 1H), 8.19–8.17 (m, 1H), 8.09–8.08 (m, 1H), 8.03–8.02 (m, 2H), 7.57–7.55 (m, 1H), 3.64 (brs, 2H), 3.30 (brs, 2H), 1.62 (brs, 4H), 1.47 (brs, 2H). MS (ESI): *m*/*z* 282.10 (M – H)^−^.

##### 4′-(Piperidine-1-carbonyl)-[1,1′-biphenyl]-4-carboxylic Acid (**7i**)

White solid; yield 53%; mp 247–248 °C. ^1^H NMR (500 MHz, DMSO-*d*_6_) δ: 13.03 (brs, 1H), 8.03 (d, *J* = 8.0 Hz, 2H), 7.84 (d, *J* = 8.0 Hz, 2H), 7.89 (d, *J* = 8.0 Hz, 2H), 7.48 (d, *J* = 8.0 Hz, 2H), 3.60 (brs, 2H), 3.20 (brs, 2H), 1.62–1.47 (m, 6H). MS (ESI): *m*/*z* 308.12 (M – H)^−^.

##### 5-(Piperidine-1-carbonyl)thiophene-2-carboxylic Acid (**7j**)

White solid; yield 90%; mp 183–184 °C. ^1^H NMR (500 MHz, DMSO-*d*_6_) δ: 13.36 (brs, 1H), 7.66 (d, *J* = 3.5 Hz, 1H), 7.34 (d, *J* = 3.5 Hz, 1H), 3.56–3.54 (m, 4H), 1.63–1.62 (m, 2H), 1.54–1.53 (m, 4H). MS (ESI): *m*/*z* 238.04 (M – H)^−^.

##### 5-(Piperidine-1-carbonyl)furan-3-carboxylic Acid (**7k**)

White solid; yield 50%; mp 169–170 °C. ^1^H NMR (500 MHz, DMSO-*d*_6_) δ: 13.01 (brs, 1H), 8.41 (s, 1H), 7.06 (s, 1H), 3.57(brs, 4H), 1.66–1.60 (brs, 2H), 1.56–1.53 (brs, 4H). MS (ESI): *m*/*z* 222.06 (M – H)^−^.

##### 4-(Piperidine-1-carbonyl)-1*H*-pyrrole-2-carboxylic Acid (**7l**)

White solid; yield 86%; mp 228–229 °C. ^1^H NMR (500 MHz, DMSO-*d*_6_) δ: 12.50 (brs, 1H), 11.87 (brs, 1H), 6.71 (s, 1H), 6.35(s, 1H), 3.53 (brs, 4H), 1.62 (brs, 2H), 1.51 (m, 4H). MS (ESI): *m*/*z* 221.08 (M – H)^−^.

#### Procedure for the Synthesis of Intermediate **9**

To a solution of compound **8** (1.00 g, 6.36 mmol) and (Boc)_2_O (1.50 g, 9.54 mmol) in CH_2_Cl_2_ (100 mL) were added DMAP (0.08 g, 0.64 mmol) and Et_3_N (1.29 g, 12.72 mmol). The reaction mixture was stirred under argon at room temperature for 6 h and then quenched with water (100 mL). The organic phase was washed with brine, dried over anhydrous Na_2_SO_4_, filtered, and evaporated *in vacuo*. The residue was purified by silica gel column chromatography (PE/EA = 100/20) to give intermediate **9**.

##### Methyl 2-((*tert*-Butoxycarbonyl)amino)thiophene-3-carboxylate (**9**)

Yellow oil; yield 34%. ^1^H NMR (500 MHz, DMSO-*d*_6_) δ: 9.95 (brs, 1H), 7.12 (d, *J* = 5.5 Hz, 1H), 6.97 (d, *J* = 6.0 Hz, 1H), 3.81 (s, 3H), 1.50 (s, 9H).

#### Procedure for the Synthesis of Intermediate **10**

To a solution of **9** (2.00 g, 7.77 mmol) in CH_3_OH (100 mL) was added 1 mol/L NaOH aqueous solution (20 mL). The reaction mixture was heated to reflux for 4 h. After cooling to room temperature, the solution was acidified with 1 N HCl aqueous solution to pH 2 at 0 °C, and the precipitated solid was filtered to afford intermediate **10**.

##### 2-((*tert*-Butoxycarbonyl)amino)thiophene-3-carboxylic Acid (**10**)

White solid; yield 63%; mp 174–175 °C. ^1^H NMR (500 MHz, DMSO-*d*_6_) δ: 13.13 (brs, 1H), 10.15 (brs, 1H), 7.10 (d, *J* = 5.5 Hz, 1H), 6.93 (d, *J* = 6.0 Hz, 1H), 1.50 (s, 9H).

#### General Procedure for the Synthesis of Intermediates **11a** and **11b**

To a solution of compound **10** (1 equiv) and alicyclic amines (1.1 equiv) in anhydrous DMF were added EDCI (1.1 equiv), HOBt (1.1 equiv), and Et_3_N (1.1 equiv) in turn. The reaction mixture was stirred under argon at room temperature for 12 h. The reaction mixture was quenched with water and extracted with ethyl acetate twice. The combined organic phase was washed with brine, dried over anhydrous Na_2_SO_4_, filtered, and evaporated *in vacuo*. The residue was purified by silica gel column chromatography (CH_2_Cl_2_/MeOH = 100/1) to give intermediates with *N*-Boc. The above intermediates (1.36 mmol) and trifluoroacetic acid (4 mL) were dissolved in CH_2_Cl_2_ (12 mL). The reaction mixture was stirred at room temperature for 2.5 h and then poured into water (15 mL). The aqueous phase was separated and basified with K_2_CO_3_ to pH 7–8. The precipitated solid was filtered to obtain intermediates **11a** and **11b**.

##### Methyl 1-(2-Aminothiophene-3-carbonyl)azetidine-3-carboxylate (**11a**)

Brown oil; yield 11%. ^1^H NMR (400 MHz, DMSO-*d*_6_) δ: 7.33 (brs, 2H), 6.76 (d, *J* = 6.0 Hz, 1H), 6.27 (d, *J* = 5.6 Hz, 1H), 4.23–4.18 (m, 4H), 3.67 (s, 3H), 3.57–3.49 (m, 1H).

##### Methyl 1-(2-Aminothiophene-3-carbonyl)piperidine-4-carboxylate (**11b**)

Black oil; yield 50%. ^1^H NMR (500 MHz, DMSO-*d*_6_) δ: 6.63 (d, *J* = 5.5 Hz, 1H), 6.36 (brs, 2H), 6.32 (d, *J* = 5.5 Hz, 1H), 4.00 (d, *J* = 13.0 Hz, 2H), 3.61 (s, 3H), 2.97 (t, *J* = 12.0 Hz, 2H), 2.62 (t, *J* = 11.0 Hz, 1H), 1.85 (d, *J* = 11.0 Hz, 2H), 1.55–1.47 (m, 2H).

#### General Procedure for the Synthesis of Intermediates **11c** and **11d**

To a solution of compound **12** (1 equiv) and alicyclic amines (1.1 equiv) in anhydrous DMF were added EDCI (1.1 equiv), HOBt (1.1 equiv), and Et_3_N (1.1 equiv) in turn. The reaction mixture was stirred under argon at room temperature for 12 h. The reaction mixture was quenched with water and extracted with ethyl acetate twice. The combined organic phase was washed with brine, dried over anhydrous Na_2_SO_4_, filtered, and evaporated *in vacuo*. The residue was purified by silica gel column chromatography (CH_2_Cl_2_/MeOH = 100/1) to give intermediates **11c** and **11d**.

##### Methyl 4-(2-Aminothiophene-3-carbonyl)piperazine-1-carboxylate (**11c**)

Purple oil; yield 49%. ^1^H NMR (500 MHz, DMSO-*d*_6_) δ: 6.66 (d, *J* = 6.0 Hz, 1H), 6.46 (brs, 2H), 6.34 (d, *J* = 6.0 Hz, 1H), 3.61 (s, 3H), 3.47 (brs, 4H), 3.41 (brs, 4H).

##### Ethyl 4-(2-Aminothiophene-3-carbonyl)piperazine-1-carboxylate (**11d**)

Brown oil; yield 46%. ^1^H NMR (500 MHz, DMSO-*d*_6_) δ: 6.66 (d, *J* = 4.0 Hz, 1H), 6.46 (brs, 2H), 6.34 (d, *J* = 3.5 Hz, 1H), 4.06 (d, *J* = 6.5 Hz, 2H), 3.47 (brs, 4H), 3.41 (brs, 4H), 1.19 (brs, 3H).

#### Procedure for the Synthesis of Intermediate **12**

To a solution of methyl 2-aminothiophene-3-carboxylate **8** (1.22 g, 7.77 mmol) in CH_3_OH (25 mL) was added 1 mol/L NaOH aqueous solution (15 mL). The reaction mixture was heated to reflux for 4 h. After cooling to room temperature, the solution was acidified with 1 N HCl solution to pH 2 at 0 °C and the precipitated solid was filtered to afford intermediate **12**.

##### 2-Aminothiophene-3-carboxylic Acid (**12**)

Brown solid; yield 62%; mp 138–140 °C. ^1^H NMR (400 MHz, DMSO-*d*_6_) δ: 11.82 (brs, 1H), 7.16 (brs, 2H), 6.79 (d, *J* = 5.6 Hz, 1H), 6.23 (d, *J* = 6.0 Hz, 1H).

#### General Procedure for the Synthesis of Intermediates **15a** and **15b**

To a solution of 2-cyanoacetic acid **13** (15.0 g, 176.35 mmol) and carbamate **14a** or **14b** (176.35 mmol) in the mixed solvents of toluene (90 mL) and DMF (5.4 mL) was added POCl_3_ (8.22 mL, 88.18 mmol) cooled with an ice bath. The reaction mixture was stirred at 80 °C for 3 h under argon and then slowly poured into ice-cold water (500 mL). The precipitated solid was filtered and washed with saturated NH_4_Cl and water to afford intermediates **15a** and **15b**, respectively.

##### Ethyl (2-Cyanoacetyl)carbamate (**15a**)

Light yellow solid; yield 64%; mp 175–176 °C. ^1^H NMR (400 MHz, DMSO-*d*_6_) δ: 10.98 (brs, 1H), 4.15–4.10 (m, 2H), 4.09 (s, 2H), 1.21 (t, *J* = 6.8 Hz, 3H).

##### Methyl (2-Cyanoacetyl)carbamate (**15b**)

White solid; yield 59%; mp 175–176 °C. ^1^H NMR (400 MHz, DMSO-*d*_6_) δ: 11.04 (brs, 1H), 4.11 (s, 2H), 3.67 (s, 3H).

#### General Procedure for the Synthesis of Intermediates **16a** and **16b**

To a solution of **15a** or **15b** (102.3 mmol) in CH_3_OH (150 mL) were added 2,5-dihydroxy-1,4-dithiane (7.77 g, 51.0 mmol) and Et_3_N (15.6 mL, 112.3 mmol) in turn cooled with an ice bath. The reaction mixture was stirred at 50 °C for 2.5 h under argon and then concentrated. The residue was diluted with CH_2_Cl_2_ (100 mL) and filtered. The obtained solid was washed with saturated NH_4_Cl solution (100 mL) and water to afford intermediates **16a** and **16b**, respectively.

##### Ethyl (2-Aminothiophene-3-carbonyl)carbamate (**16a**)

Light yellow solid; yield 78%; mp 144–145 °C. ^1^H NMR (400 MHz, DMSO-*d*_6_) δ: 10.02 (brs, 1H), 7.65 (brs, 2H), 7.25 (d, *J* = 6.0 Hz, 1H), 6.22 (d, *J* = 6.0 Hz, 1H), 4.16–4.10 (m, 2H), 1.23 (t, *J* = 7.6 Hz, 3H). MS (ESI): *m*/*z* 215.05 (M + H)^+^.

##### Methyl (2-Aminothiophene-3-carbonyl)carbamate (**16b**)

Light Yellow solid; yield 59%; mp 152–153 °C. ^1^H NMR (400 MHz, DMSO-*d*_6_) δ: 10.07 (brs, 1H), 7.65 (brs, 2H), 7.23 (d, *J* = 6.0 Hz, 1H), 6.23 (d, *J* = 6.0 Hz, 1H), 3.67 (s, 3H). MS (ESI): *m*/*z* 201.03 (M + H)^+^.

#### Procedure for the Synthesis of Intermediate **18**

To a magnetically stirred solution of 2-cyanoacetamide **17** (2.0 g, 23.79 mmol) in 1,2-dichloroethane (50 mL) was added oxalyl chloride (6.0 mL, 71.36 mmol). The reaction mixture was heated to reflux for 4 h under an argon atmosphere. The solvent was evaporated under reduced pressure. To a solution of the residue in anhydrous acetonitrile (30 mL) was added ethylamine (20.0 mL) in anhydrous acetonitrile (80 mL) dropwise keeping the reaction under −10 °C. The reaction mixture was stirred for additional 3 h at −10 °C and then concentrated. The residue was washed with water, filtered, dried, and used in the next step without further purification.

#### Procedure for the Synthesis of Intermediate **19**

To a solution of compound **18** (20.0 mg, 1.24 mmol) in CH_3_OH (20 mL) were added 2,5-dihydroxy-1,4-dithiane (98.0 mg, 0.65 mmol) and Et_3_N (0.27 mL, 1.94 mmol) cooled with an ice bath. The reaction mixture was stirred at 50 °C for 2.5 h under argon and then concentrated. The residue was purified by silica gel column chromatography (CH_2_Cl_2_/MeOH = 100/1) to give compound **19**.

##### 2-Amino-*N*-(ethylcarbamoyl)thiophene-3-carboxamide (**19**)

Yellow solid; yield 54%; mp 155–156 °C. ^1^H NMR (400 MHz, DMSO-*d*_6_) δ: 9.68 (brs, 1H), 8.69–8.66 (m, 1H), 7.59 (brs, 2H), 7.36 (d, *J* = 6.0 Hz, 1H), 6.25 (d, *J* = 6.0 Hz, 1H), 3.23–3.20 (m, 2H), 1.09 (t, *J* = 6.8 Hz, 3H).

#### General Procedure for the Synthesis of Intermediates **21a**–**h**

To a solution of compounds **20a**–**h** (1 equiv) and cyanoacetic acid **13** (3 equiv) in anhydrous DMF were added EDCI (2 equiv) and DMAP (0.5 equiv). The reaction mixture was stirred under argon at room temperature for 24 h. The reaction mixture was quenched with water and extracted with ethyl acetate twice. The combined organic phase was washed with brine, dried over anhydrous Na_2_SO_4_, filtered, and evaporated *in vacuo*. The residue was purified by silica gel column chromatography (CH_2_Cl_2_/MeOH = 100/1) to give compounds **21a**–**h**.

##### 2-Cyano-*N*-(5-methylthiazol-2-yl)acetamide (**21a**)

White solid; yield 95%; mp 154–155 °C. ^1^H NMR (500 MHz, DMSO-*d*_6_) δ: 12.26 (brs, 1H), 7.15 (s, 1H), 4.00 (s, 2H), 2.35 (s, 3H).

##### 2-Cyano-*N*-(4-methylpyrimidin-2-yl)acetamide (**21b**)

White solid; yield 82%; mp 133–134 °C. ^1^H NMR (400 MHz, DMSO-*d*_6_) δ: 10.88 (brs, 1H), 8.51 (d, *J* = 4.8 Hz, 1H), 7.10 (d, *J* = 4.8 Hz, 1H), 4.16 (s, 2H), 2.42 (s, 3H).

##### 2-Cyano-*N*-(4-(trifluoromethyl)pyrimidin-2-yl)acetamide (**21c**)

White solid; yield 50%; mp 186–188 °C. ^1^H NMR (400 MHz, DMSO-*d*_6_) δ: 11.46 (brs, 1H), 9.05 (d, *J* = 4.8 Hz, 1H), 7.71 (d, *J* = 5.2 Hz, 1H), 4.15 (s, 2H).

##### 2-Cyano-*N*-(5-methylpyrazin-2-yl)acetamide (**21d**)

White solid; yield 76%; mp 177–179 °C. ^1^H NMR (500 MHz, DMSO-*d*_6_) δ: 11.00 (brs, 1H), 9.12 (s, 1H), 8.29(s, 1H), 4.00 (s, 2H), 2.44 (s, 3H).

##### 2-Cyano-*N*-(6-methylpyridin-2-yl)acetamide (**21e**)

White solid; yield 63%; mp 93–95 °C. ^1^H NMR (500 MHz, DMSO-*d*_6_) δ: 10.77 (brs, 1H), 7.81 (d, *J* = 6.0 Hz, 1H), 7.70 (t, *J* = 8.0 Hz, 1H), 7.01 (d, *J* = 7.5 Hz, 1H), 3.94 (s, 2H), 2.40 (s, 3H).

##### 2-Cyano-*N*-(4-methylpyridin-2-yl)acetamide (**21f**)

White solid; yield 42%; mp 93–95 °C. ^1^H NMR (400 MHz, DMSO-*d*_6_) δ: 10.73 (brs, 1H), 8.18 (d, *J* = 5.2 Hz, 1H), 7.86 (brs, 1H), 6.99–6.97 (m, 1H), 3.96 (s, 2H), 2.32 (s, 3H).

##### 2-Cyano-*N*-(4,6-dimethylpyrimidin-2-yl)acetamide (**21g**)

White solid; yield 38%; mp 157–158 °C. ^1^H NMR (500 MHz, DMSO-*d*_6_) δ: 10.78 (brs, 1H), 6.98 (s, 1H), 4.16 (s, 2H), 2.36 (s, 6H).

##### 2-Cyano-*N*-(5-methylbenzo[*d*]thiazol-2-yl)acetamide (**21h**)

White solid; yield 13%; mp 174–176 °C. ^1^H NMR (400 MHz, DMSO-*d*_6_) δ: 12.56 (brs, 1H), 7.75 (s, 1H), 7.61 (d, *J* = 8.0 Hz, 1H), 7.24–7.21 (m, 1H), 4.06 (s, 2H), 2.37 (s, 3H).

#### General Procedure for the Synthesis of Intermediates **22a**–**h**

To a solution of compound **21a**–**h** (1 equiv) in methanol were added 2,5-dihydroxy-1,4-dithiane (0.5 equiv) and Et_3_N (1.1 equiv). The reaction mixture was stirred for 7 h under argon at 50 °C and then evaporated *in vacuo*. The residue was purified by silica gel column chromatography (CH_2_Cl_2_/MeOH = 100/1) to give compounds **22a**–**h**.

##### 2-Amino-*N*-(5-methylthiazol-2-yl)thiophene-3-carboxamide (**22a**)

Yellow solid; yield 66%; mp 178–180 °C. ^1^H NMR (500 MHz, DMSO-*d*_6_) δ: 11.51 (brs, 1H), 7.57 (brs, 2H), 7.46 (d, *J* = 5.5 Hz, 1H), 7.14 (s, 1H), 6.30 (d, *J* = 6.0 Hz, 1H), 2.34 (s, 3H).

##### 2-Amino-*N*-(4-methylpyrimidin-2-yl)thiophene-3-carboxamide (**22b**)

Yellow solid; yield 77%; mp 108–110 °C. ^1^H NMR (400 MHz, CDCl_3_) δ: 8.49 (d, *J* = 5.2 Hz, 1H), 8.13 (brs, 1H), 6.89 (d, *J* = 5.6 Hz, 1H), 6.86 (d, *J* = 5.2 Hz, 1H), 6.44 (brs, 2H), 6.25 (d, *J* = 5.6 Hz, 1H), 2.49 (s, 3H).

##### 2-Amino-*N*-(4-(trifluoromethyl)pyrimidin-2-yl)thiophene-3-carboxamide (**22c**)

Yellow solid; yield 46%; mp 134–135 °C. ^1^H NMR (400 MHz, DMSO-*d*_6_) δ: 10.61 (brs, 1H), 9.01 (d, *J* = 4.8 Hz, 1H), 7.64 (brs, 2H), 7.61 (d, *J* = 5.2 Hz, 1H), 7.35 (d, *J* = 6.0 Hz, 1H), 6.27 (d, *J* = 6.0 Hz, 1H).

##### 2-Amino-*N*-(5-methylpyrazin-2-yl)thiophene-3-carboxamide (**22d**)

White solid; yield 31%; mp 178–179 °C. ^1^H NMR (400 MHz, DMSO-*d*_6_) δ: 10.07 (s, 1H), 9.22 (s, 1H), 8.29 (s, 1H), 7.56 (brs, 2H), 7.47 (d, *J* = 6.0 Hz, 1H), 6.29 (d, *J* = 6.0 Hz, 1H), 2.45 (s, 3H).

##### 2-Amino-*N*-(6-methylpyridin-2-yl)thiophene-3-carboxamide (**22e**)

Yellow solid; yield 70%; mp 140–141 °C. ^1^H NMR (500 MHz, DMSO-*d*_6_) δ: 9.75 (brs, 1H), 7.93 (d, *J* = 8.0 Hz, 1H), 7.63 (t, *J* = 8.0 Hz, 1H), 7.50 (s, 1H), 7.49 (brs, 2H), 6.93 (d, *J* = 7.5 Hz, 1H), 6.27 (d, *J* = 5.5 Hz, 1H), 2.42 (s, 3H).

##### 2-Amino-*N*-(4-methylpyridin-2-yl)thiophene-3-carboxamide (**22f**)

Brown oil; yield 40%. ^1^H NMR (500 MHz, DMSO-*d*_6_) δ: 9.79 (brs, 1H), 8.17 (s, 1H), 7.99 (s, 1H), 7.52 (brs, 2H), 7.48 (d, *J* = 4.5 Hz, 1H), 6.92 (s, 1H), 6.28 (d, *J* = 4.5 Hz, 1H), 2.31 (s, 3H).

##### 2-Amino-*N*-(4,6-dimethylpyrimidin-2-yl)thiophene-3-carboxamide (**22g**)

White solid; yield 78%; mp 166–167 °C. ^1^H NMR (500 MHz, DMSO-*d*_6_) δ: 9.95 (brs, 1H), 7.51 (brs, 2H), 7.35 (d, *J* = 4.5 Hz, 1H), 6.93 (s, 1H), 6.25 (d, *J* = 4.0 Hz, 1H), 2.36 (s, 6H).

##### 2-Amino-*N*-(5-methylbenzo[*d*]thiazol-2-yl)thiophene-3-carboxamide (**22h**)

White solid; yield 74%; mp 217–218 °C. ^1^H NMR (400 MHz, DMSO-*d*_6_) δ: 11.88 (brs, 1H), 7.74 (s, 1H), 7.70 (brs, 2H), 7.60 (d, *J* = 8.4 Hz, 1H), 7.52 (d, *J* = 5.6 Hz, 1H), 7.24 (dd, *J* = 8.4, 1.2 Hz, 1H), 6.34 (d, *J* = 6.0 Hz, 1H), 2.41 (s, 3H).

#### General Procedure for the Synthesis of Target Compounds **23a**–**p**

To a solution of benzoic acids **4a**–**p** (1.2 equiv) and 2-aminothiophene **16a** (1 equiv) in DMF were added HATU (2 equiv) and Et_3_N (3 equiv) in turn. The reaction mixture was stirred at room temperature for 12 h, then quenched with water, and extracted with CH_2_Cl_2_ thrice. The combined organic phase was washed with brine, dried over anhydrous Na_2_SO_4_, filtered, and evaporated *in vacuo*. The residue was purified by silica gel column chromatography (CH_2_Cl_2_/MeOH = 100/1) to give target compounds **23a**–**p**.

##### Ethyl (2-(4-(Propylcarbamoyl)benzamido)thiophene-3-carbonyl)carbamate (**23a**)

White solid; yield 35%; mp 219–220 °C. ^1^H NMR (400 MHz, DMSO-*d*_6_) δ: 12.64 (brs, 1H), 10.83 (brs, 1H), 8.67 (brs, 1H), 8.06–8.00 (m, 4H), 7.74 (d, *J* = 6.0 Hz, 1H), 7.10 (d, *J* = 6.0 Hz, 1H), 4.25–4.19 (m, 2H), 3.26–3.24 (m, 2H), 1.57–1.55 (m, 2H), 1.29 (t, *J* = 7.2 Hz, 3H), 0.91 (t, *J* = 7.2 Hz, 3H). ^13^C NMR (100 MHz, DMSO-*d*_6_) δ: 165.3, 164.1, 162.6, 151.3, 149.0, 138.6, 133.9, 128.1, 127.3, 123.4, 117.0, 115.4, 61.4, 41.2, 22.4, 14.3, 11.6. HRMS (ESI): *m*/*z* [M + H]^+^ calcd for C_19_H_22_N_3_O_5_S, 404.1275; found, 404.1267.

##### Ethyl (2-(4-(Ethyl(methyl)carbamoyl)benzamido)thiophene-3-carbonyl)carbamate (**23b**)

White solid; yield 57%; mp 161–162 °C. ^1^H NMR (400 MHz, DMSO-*d*_6_) δ: 12.66 (brs, 1H), 10.83 (brs, 1H), 8.01 (m, 2H), 7.75–7.64 (m, 3H), 7.11 (m, 1H), 4.23 (m, 2H), 3.20–3.10 (m, 2H), 2.98–2.88 (m, 3H), 1.29 (brs, 3H), 1.17–1.08 (m, 3H). ^13^C NMR (100 MHz, DMSO-*d*_6_) δ: 169.2 (168.6), 164.1, 162.4, 151.1, 149.1, 141.1, 132.2, 127.6 (127.5), 127.4 (127.1), 123.3, 116.8, 115.2, 61.3, 45.2 (41.5), 36.2 (31.6), 14.3, 13.3 (11.9). HRMS (ESI): *m*/*z* [M + H]^+^ calcd for C_19_H_22_N_3_O_5_S, 404.1275; found, 404.1273.

##### Ethyl (2-(4-(Diethylcarbamoyl)benzamido)thiophene-3-carbonyl)carbamate (**23c**)

White solid; yield 10%; mp 178–179 °C. ^1^H NMR (500 MHz, DMSO-*d*_6_) δ: 12.65 (brs, 1H), 10.82 (brs, 1H), 8.00 (d, *J* = 7.5 Hz, 2H), 7.74 (d, *J* = 5.0 Hz, 1H), 7.61 (d, *J* = 7.5 Hz, 2H), 7.10 (d, *J* = 5.0 Hz, 1H), 4.22–4.21 (m, 2H), 3.46 (brs, 2H), 3.17 (brs, 2H), 1.29 (t, *J* = 7.0 Hz, 3H), 1.17 (brs, 3H), 1.06 (brs, 3H). ^13^C NMR (100 MHz, DMSO-*d*_6_) δ: 169.0, 164.2, 162.5, 151.2, 149.2, 141.5, 132.1, 127.6, 127.0, 123.4, 116.9, 115.3, 61.4, 42.9, 38.8, 14.3, 14.1, 12.9. HRMS (ESI): *m*/*z* [M + H]^+^ calcd for C_20_H_24_N_3_O_5_S, 418.1431; found, 418.1411.

##### Ethyl (2-(4-(((3*S*,5*S*,7*S*)-Adamantan-1-yl)carbamoyl)benzamido)thiophene-3-carbonyl)carbamate (**23d**)

White solid; yield 4%; mp 157–158 °C. ^1^H NMR (400 MHz, DMSO-*d*_6_) δ: 12.65 (brs, 1H), 10.82 (brs, 1H), 8.00–7.95 (m, 4H), 7.84 (brs, 1H), 7.74 (d, *J* = 6.0 Hz, 1H), 7.10 (d, *J* = 6.0 Hz, 1H), 4.25–4.19 (m, 2H), 2.09–2.07 (m, 10H), 1.67 (brs, 5H), 1.29 (t, *J* = 7.2 Hz, 3H). ^13^C NMR (100 MHz, DMSO-*d*_6_) δ: 165.3, 164.2, 162.6, 151.3, 149.1, 139.9, 133.6, 128.3, 127.1, 123.4, 116.9, 115.3, 61.4, 51.9, 40.9, 36.1, 29.0, 14.3. HRMS (ESI): *m*/*z* [M + H]^+^ calcd for C_26_H_30_N_3_O_5_S, 496.1901; found, 496.1881.

##### Ethyl (2-(4-(Cyclohexylcarbamoyl)benzamido)thiophene-3-carbonyl)carbamate (**23e**)

White solid; yield 11%; mp > 250 °C. ^1^H NMR (400 MHz, DMSO-*d*_6_) δ: 12.64 (brs, 1H), 10.82 (brs, 1H), 8.42 (d, *J* = 8.0 Hz, 1H), 8.05–7.99 (m, 4H), 7.74 (d, *J* = 6.0 Hz, 1H), 7.10 (d, *J* = 6.0 Hz, 1H), 4.25–4.19 (m, 2H), 3.79–3.77 (m, 1H), 1.84–1.72 (m, 4H), 1.64–1.59 (m, 1H), 1.35–1.27 (m, 7H), 1.24–1.22 (m, 1H). ^13^C NMR (100 MHz, DMSO-*d*_6_) δ: 164.6, 164.1, 162.6, 151.3, 149.1, 138.8, 133.8, 128.2, 127.2, 123.4, 117.0, 115.4, 61.4, 48.6, 32.4, 25.3, 25.0, 14.3. HRMS (ESI): *m*/*z* [M + H]^+^ calcd for C_22_H_26_N_3_O_5_S, 444.1588; found, 444.1597.

##### Ethyl (2-(4-((4,4-Dimethylcyclohexyl)carbamoyl)benzamido)thiophene-3-carbonyl)carbamate (**23f**)

White solid; yield 17%; mp > 250 °C. ^1^H NMR (400 MHz, DMSO-*d*_6_) δ: 12.64 (brs, 1H), 10.82 (brs, 1H), 8.41 (d, *J* = 8.0 Hz, 1H), 8.05–8.00 (m, 4H), 7.74 (d, *J* = 6.0 Hz, 1H), 7.10 (d, *J* = 6.0 Hz, 1H), 4.24–4.19 (m, 2H), 3.75–3.72 (m, 1H), 1.65–1.64 (m, 2H), 1.59–1.50 (m, 2H), 1.42–1.39 (m, 2H), 1.30–1.27 (m, 5H), 0.94 (s, 3H), 0.92 (s, 3H). ^13^C NMR (100 MHz, DMSO-*d*_6_) δ: 164.6, 164.2, 162.6, 151.3, 149.1, 138.8, 133.9, 128.2, 127.3, 123.4, 117.0, 115.4, 61.4, 48.7, 37.7, 32.1, 29.4, 28.0, 24.4, 14.3. HRMS (ESI): *m*/*z* [M + H]^+^ calcd for C_24_H_30_N_3_O_5_S, 472.1901; found, 472.1909.

##### Ethyl (2-(4-(Phenylcarbamoyl)benzamido)thiophene-3-carbonyl)carbamate (**23g**)

White solid; yield 39%; mp > 250 °C. ^1^H NMR (400 MHz, DMSO-*d*_6_) δ: 12.69 (brs, 1H), 10.84 (brs, 1H), 10.46 (brs, 1H), 8.16 (d, *J* = 8,4 Hz, 2H), 8.08 (d, *J* = 8,4 Hz, 2H), 7.79 (d, *J* = 8,0 Hz, 2H), 7.75 (d, *J* = 6,0 Hz, 1H), 7.40–7.36 (m, 2H), 7.15–7.11 (m, 2H), 4.25–4.20 (m, 2H), 1.29 (t, *J* = 7.6 Hz, 3H). ^13^C NMR (100 MHz, DMSO-*d*_6_) δ: 164.8, 164.2, 162.5, 151.3, 149.1, 139.0, 138.9, 134.3, 128.7, 128.6, 127.4, 124.0, 123.4, 120.5, 116.9, 115.4, 61.4, 14.3. HRMS (ESI): *m*/*z* [M + H]^+^ calcd for C_22_H_20_N_3_O_5_S, 438.1118; found, 438.1119.

##### Ethyl (2-(4-(Azetidine-1-carbonyl)benzamido)thiophene-3-carbonyl)carbamate (**23h**)

White solid; yield 40%; mp 182–183 °C. ^1^H NMR (400 MHz, DMSO-*d*_6_) δ: 12.66 (brs, 1H), 10.82 (brs, 1H), 8.01 (d, *J* = 8.0 Hz, 2H), 7.85 (d, *J* = 8.0 Hz, 2H), 7.74 (d, *J* = 6.0 Hz, 1H), 7.10 (d, *J* = 6.0 Hz, 1H), 4.33 (t, *J* = 7.6 Hz, 2H). 4.24–4.19 (m, 2H), 4.08 (t, *J* = 7.6 Hz, 2H), 2.32–2.24 (m, 2H), 1.29 (t, *J* = 7.6 Hz, 3H). ^13^C NMR (100 MHz, DMSO-*d*_6_) δ: 167.8, 164.2, 162.4, 151.2, 149.1, 137.2, 133.6, 128.6, 127.4, 123.4, 116.9, 115.3, 61.4, 53.0, 48.7, 15.6, 14.3. HRMS (ESI): *m*/*z* [M + H]^+^ calcd for C_19_H_20_N_3_O_5_S, 402.1118; found, 402.1097.

##### Ethyl (2-(4-(Pyrrolidine-1-carbonyl)benzamido)thiophene-3-carbonyl)carbamate (**23i**)

White solid; yield 8%; mp 172–173 °C. ^1^H NMR (400 MHz, DMSO-*d*_6_) δ: 12.66 (brs, 1H), 10.83 (brs, 1H), 8.00 (d, *J* = 8.0 Hz, 2H), 7.77–7.73 (m, 3H), 7.10 (d, *J* = 5.6 Hz, 1H), 4.24–4.19 (m, 2H), 3.50 (t, *J* = 6.4 Hz, 2H), 3.38 (t, *J* = 6.4 Hz, 2H), 1.90–1.81 (m, 4H), 1.29 (t, *J* = 7.2 Hz, 3H). ^13^C NMR (100 MHz, DMSO-*d*_6_) δ: 167.2, 164.2, 162.5, 151.2, 149.1, 141.3, 132.7, 127.9, 127.3, 123.4, 116.9, 115.3, 61.4, 48.9, 46.0, 26.0, 24.0, 14.3. HRMS (ESI): *m*/*z* [M + H]^+^ calcd for C_20_H_22_N_3_O_5_S, 416.1275; found, 416.1256.

##### Ethyl (2-(4-(Piperidine-1-carbonyl)benzamido)thiophene-3-carbonyl)carbamate (**23j**)

White solid; yield 33%; mp 177–178 °C. ^1^H NMR (400 MHz, DMSO-*d*_6_) δ: 12.64 (brs, 1H), 10.81 (brs, 1H), 8.00 (d, *J* = 8.0 Hz, 2H), 7.74 (d, *J* = 6.0 Hz, 1H), 7.62 (d, *J* = 8.0 Hz, 2H), 7.10 (d, *J* = 6.0 Hz, 1H), 4.24–4.19 (m, 2H), 3.61 (brs, 2H), 3.25 (brs, 2H), 1.62 (brs, 4H), 1.47 (brs, 2H), 1.29 (t, *J* = 6.8 Hz, 3H). ^13^C NMR (100 MHz, DMSO-*d*_6_) δ: 167.8, 164.2, 162.5, 151.2, 149.1, 140.8, 132.4, 127.6, 127.5, 123.4, 116.9, 115.3, 61.4, 48.0, 42.4, 26.0, 25.3, 24.1, 14.3. HRMS (ESI): *m*/*z* [M + H]^+^ calcd for C_21_H_24_N_3_O_5_S, 430.1431; found, 430.1435.

##### Ethyl (2-(4-(Azepane-1-carbonyl)benzamido)thiophene-3-carbonyl)carbamate (**23k**)

White solid; yield 34%; mp 148–149 °C. ^1^H NMR (400 MHz, DMSO-*d*_6_) δ: 12.65 (brs, 1H), 10.82 (brs, 1H), 8.00 (d, *J* = 8.2 Hz, 2H), 7.74 (d, *J* = 5.6 Hz, 1H), 7.61 (d, *J* = 8.2 Hz, 2H), 7.10 (d, *J* = 5.6 Hz, 1H), 4.24–4.19 (m, 2H), 3.58 (brs, 2H), 3.29 (brs, 2H), 1.73 (brs, 2H), 1.62–1.48 (m, 6H), 1.28 (t, *J* = 7.0 Hz, 3H). ^13^C NMR (100 MHz, DMSO-*d*_6_) δ: 169.2, 164.1, 162.5, 151.2, 149.1, 141.5, 132.0, 127.4, 127.1, 123.3, 116.8, 115.2, 61.3, 49.0, 45.4, 28.7, 27.1, 26.8, 25.8, 14.2. HRMS (ESI): *m*/*z* [M + H]^+^ calcd for C_22_H_26_N_3_O_5_S, 444.1588; found, 444.1563.

##### Ethyl (2-(4-(4-Methylpiperidine-1-carbonyl)benzamido)thiophene-3-carbonyl)carbamate (**23l**)

White solid; yield 47%; mp 79–81 °C. ^1^H NMR (400 MHz, DMSO-*d*_6_) δ: 12.64 (brs, 1H), 10.82 (brs, 1H), 8.00 (d, *J* = 8.4 Hz, 2H), 7.74 (d, *J* = 6.0 Hz, 1H), 7.62 (d, *J* = 8.4 Hz, 2H), 7.10 (d, *J* = 6.0 Hz, 1H), 4.24–4.19 (m, 2H), 3.13–3.07 (m, 4H), 1.29 (t, *J* = 6.0 Hz, 3H), 1.19–1.15 (m, 5H), 0.93 (d, *J* = 6.8 Hz, 3H). ^13^C NMR (100 MHz, DMSO-*d*_6_) δ: 167.7, 164.1, 162.5, 151.2, 149.1, 140.7, 132.3, 127.5, 127.4, 123.3, 116.8, 115.2, 61.3, 47.2, 41.6, 34.0, 33.4, 30.4, 21.6, 14.2. HRMS (ESI): *m*/*z* [M + H]^+^ calcd for C_22_H_26_N_3_O_5_S, 444.1588; found, 444.1577.

##### Ethyl (2-(4-(4-Methoxypiperidine-1-carbonyl)benzamido)thiophene-3-carbonyl)carbamate (**23m**)

White solid; yield 15%; mp 160–161 °C. ^1^H NMR (400 MHz, DMSO-*d*_6_) δ: 12.64 (brs, 1H), 10.82 (brs, 1H), 8.00 (d, *J* = 8.0 Hz, 2H), 7.74 (d, *J* = 6.0 Hz, 1H), 7.64 (d, *J* = 8.0 Hz, 2H), 7.10 (d, *J* = 6.0 Hz, 1H), 4.24–4.19 (m, 2H), 3.93 (brs, 1H), 3.45–3.32 (m, 3H), 3.26 (s, 3H), 3.15 (brs, 1H), 1.90–1.80 (m, 2H), 1.44 (brs, 2H), 1.29 (t, *J* = 7.2 Hz, 3H). ^13^C NMR (100 MHz, DMSO-*d*_6_) δ: 167.9, 164.2, 162.5, 151.2, 149.1, 140.5, 132.5, 127.6, 123.4, 116.9, 115.3, 74.9, 61.4, 55.1, 44.5, 30.8, 30.1, 14.3. HRMS (ESI): *m*/*z* [M + H]^+^ calcd for C_22_H_26_N_3_O_6_S, 460.1537; found, 460.1533.

##### Ethyl (2-(4-(4,4-Difluoropiperidine-1-carbonyl)benzamido)thiophene-3-carbonyl)carbamate (**23n**)

White solid; yield 57%; mp 177–178 °C. ^1^H NMR (400 MHz, DMSO-*d*_6_) δ: 12.65 (brs, 1H), 10.82 (brs, 1H), 8.01 (d, *J* = 8.4 Hz, 2H), 7.74–7.70 (m, 3H), 7.10 (d, *J* = 5.6 Hz, 1H), 4.24–4.19 (m, 2H), 3.87–3.76 (m, 2H), 3.48–3.40 (m, 2H), 2.06 (brs, 4H), 1.29 (t, *J* = 6.8 Hz, 3H). ^13^C NMR (100 MHz, DMSO-*d*_6_) δ: 168.2, 164.2, 162.5, 151.2, 149.1, 139.9, 132.8, 127.7, 127.6, 123.4, 122.8 (*J* = 240 Hz), 116.9, 115.3, 61.4, 44.0, 38.5, 33.7, 14.3. HRMS (ESI): *m*/*z* [M + H]^+^ calcd for C_21_H_22_F_2_N_3_O_5_S, 466.1243; found, 466.1220.

##### Ethyl (2-(4-(Morpholine-4-carbonyl)benzamido)thiophene-3-carbonyl)carbamate (**23o**)

White solid; yield 79%; mp 159–160 °C. ^1^H NMR (400 MHz, DMSO-*d*_6_) δ: 12.65 (brs, 1H), 10.82 (brs, 1H), 8.01 (d, *J* = 7.6 Hz, 2H), 7.74 (d, *J* = 5.6 Hz, 1H), 7.67 (d, *J* = 7.6 Hz, 2H), 7.10 (d, *J* = 5.6 Hz, 1H), 4.22–4.21 (m, 2H), 3.66–3.57 (m, 6H), 3.33 (brs, 2H), 1.29 (t, *J* = 6.8 Hz, 3H). ^13^C NMR (100 MHz, DMSO-*d*_6_) δ: 168.0, 164.1, 162.4, 151.2, 149.0, 139.7, 132.6, 127.8, 127.5, 123.3, 116.8, 115.2, 66.0, 61.3, 47.6, 42.0, 14.2. HRMS (ESI): *m*/*z* [M + H]^+^ calcd for C_20_H_22_N_3_O_6_S, 432.1224; found, 432.1207.

##### Ethyl (2-(4-(Thiomorpholine-4-carbonyl)benzamido)thiophene-3-carbonyl)carbamate (**23p**)

White solid; yield 17%; mp 178–179 °C. ^1^H NMR (400 MHz, DMSO-*d*_6_) δ: 12.64 (brs, 1H), 10.82 (brs, 1H), 8.01 (d, *J* = 8.4 Hz, 2H), 7.74 (d, *J* = 6.0 Hz, 1H), 7.66 (d, *J* = 8.4 Hz, 2H), 7.10 (d, *J* = 6.0 Hz, 1H), 4.24–4.19 (m, 2H), 3.90–3.89 (m, 2H), 3.52 (brs, 2H), 2.70–2.50 (m, 4H), 1.29 (t, *J* = 7.6 Hz, 3H). ^13^C NMR (100 MHz, DMSO-*d*_6_) δ: 168.4, 164.2, 162.5, 151.2, 149.1, 140.3, 132.5, 127.6, 127.5, 123.4, 116.9, 115.3, 61.4, 49.7, 44.0, 27.0, 26.7, 14.3. HRMS (ESI): *m*/*z* [M + H]^+^ calcd for C_20_H_22_N_3_O_5_S_2_, 448.0995; found, 448.0971.

#### General Procedure for the Synthesis of Target Compounds **24a**, **24c**, **24d**, **24f**, and **24h**–**l**

The target compounds were prepared from **16a** and corresponding aryl carboxylic acids **7** in the same manner as described for **23a**–**p**.

#### General Procedure for the Synthesis of Target Compounds **24b**, **24e**, and **24g**

To a magnetically stirred solution of the benzoic acids **7b**, **7e** or **7g** (1 equiv) in CH_2_Cl_2_ were added thionyl chloride (2 equiv) and a drop of DMF. The reaction mixture was heated to reflux for 3 h under an argon atmosphere. The solvent was evaporated under reduced pressure. The residue was dissolved in anhydrous THF. To a solution of 2-aminothiophene **16a** (0.7 equiv), DMAP (0.07 equiv), and Et_3_N (3.5 equiv) in anhydrous THF was slowly added the above THF solution cooled with an ice bath. The reaction mixture was stirred for additional 3 h at room temperature and then concentrated. The residue was purified by silica gel column chromatography (CH_2_Cl_2_/MeOH = 100/1) to give target compounds **24b**, **24e**, and **24g**, respectively.

##### Ethyl (2-(3-(Piperidine-1-carbonyl)benzamido)thiophene-3-carbonyl)carbamate (**24a**)

White solid; yield 18%; mp 175–176 °C. ^1^H NMR (500 MHz, DMSO-*d*_6_) δ: 12.63 (brs, 1H), 10.81 (brs, 1H), 8.00 (brs, 1H), 7.89 (brs, 1H), 7.79–7.72 (m, 3H), 7.10 (s, 1H), 4.22–4.21 (m, 2H), 3.62 (brs, 2H), 3.30 (brs, 2H), 1.62 (brs, 4H), 1.50 (brs, 2H), 1.28 (brs, 3H). ^13^C NMR (100 MHz, DMSO-*d*_6_) δ: 167.8, 164.1, 162.5, 151.3, 149.1, 137.4, 132.2, 131.0, 129.6, 128.0, 125.6, 123.4, 116.9, 115.3, 61.4, 48.2, 42.5, 26.0, 25.3, 24.1, 14.3. HRMS (ESI): *m*/*z* [M + H]^+^ calcd for C_21_H_24_N_3_O_5_S, 430.1431; found, 430.1434.

##### Ethyl (2-(2-Fluoro-4-(piperidine-1-carbonyl)benzamido)thiophene-3-carbonyl)carbamate (**24b**)

White solid; yield 38%; mp 163–164 °C. ^1^H NMR (400 MHz, DMSO-*d*_6_) δ: 12.71 (d, *J* = 9.6 Hz, 1H), 10.77 (brs, 1H), 8.10 (t, *J* = 7.6 Hz, 1H), 7.73 (d, *J* = 6.0 Hz, 1H), 7.53 (d, *J* = 7.6 Hz, 1H), 7.41 (dd, *J*_1_ = 8.0 Hz, *J*_2_ = 1.2 Hz, 1H), 7.12 (d, *J* = 6.0 Hz, 1H), 4.24–4.18 (m, 2H), 3.60 (brs, 2H), 3.25 (brs, 2H), 1.61–1.60 (m, 4H), 1.47 (brs, 2H), 1.28 (t, *J* = 7.6 Hz, 3H). ^13^C NMR (100 MHz, DMSO-*d*_6_) δ: 166.4, 163.6, 159.8 (q, *J* = 250 Hz), 159.2 (q, *J* = 2 Hz), 151.3, 148.3, 143.0 (q, *J* = 8 Hz), 139.2, 132.1, 123.5, 119.8 (*J* = 11 Hz), 117.3, 115.7, 115.1 (q, *J* = 25 Hz), 61.4, 48.0, 42.4, 25.9, 25.2, 24.0, 14.3. HRMS (ESI): *m*/*z* [M + H]^+^ calcd for C_21_H_23_FN_3_O_5_S, 448.1337; found, 448.1342.

##### Ethyl (2-(2-Chloro-4-(piperidine-1-carbonyl)benzamido)thiophene-3-carbonyl)carbamate (**24c**)

White solid; yield 16%; mp 59–60 °C. ^1^H NMR (400 MHz, DMSO-*d*_6_) δ: 12.12 (brs, 1H), 10.74 (brs, 1H), 7.79 (d, *J* = 7.6 Hz, 1H), 7.65 (d, *J* = 6.0 Hz, 1H), 7.58 (d, *J* = 1.2 Hz, 1H), 7.46 (dd, *J*_1_ = 7.6 Hz, *J*_2_ = 1.2 Hz, 1H), 7.09 (d, *J* = 6.0 Hz, 1H), 4.17–4.11 (m, 2H), 3.55 (brs, 2H), 3.23 (brs, 2H), 1.58–1.52 (m, 4H), 1.44 (br, 2H), 1.22 (t, *J* = 7.2 Hz, 3H). ^13^C NMR (100 MHz, DMSO-*d*_6_) δ: 166.4, 163.5, 162.5, 151.3, 147.8, 140.8, 133.8, 130.7, 130.3, 128.4, 125.8, 123.5, 117.2, 116.0, 61.3, 48.1, 42.4, 25.9, 25.2, 24.0, 14.3. HRMS (ESI): *m*/*z* [M + H]^+^ calcd for C_21_H_23_ClN_3_O_5_S, 464.1042; found, 464.0981.

##### Ethyl (2-(2-Bromo-4-(piperidine-1-carbonyl)benzamido)thiophene-3-carbonyl)carbamate (**24d**)

White solid; yield 9%; mp 144–145 °C. ^1^H NMR (400 MHz, DMSO-*d*_6_) δ: 12.04 (brs, 1H), 10.79 (brs, 1H), 7.78–7.76 (m, 2H), 7.69 (d, *J* = 6.0 Hz, 1H), 7.54 (d, *J* = 8.0 Hz, 1H), 7.14 (d, *J* = 6.0 Hz, 1H), 4.21–4.16 (m, 2H), 3.59 (brs, 2H), 3.28 (brs, 2H), 1.61 (brs, 4H), 1.49 (brs, 2H), 1.26 (t, *J* = 7.2 Hz, 3H). ^13^C NMR (100 MHz, DMSO-*d*_6_) δ: 166.4, 163.6, 151.3, 147.8, 140.6, 136.3, 131.4, 129.8, 126.2, 123.5, 119.4, 117.2, 116.0, 61.4, 48.1, 42.4, 25.9, 25.2, 24.0, 14.3. HRMS (ESI): *m*/*z* [M + H]^+^ calcd for C_21_H_23_BrN_3_O_5_S, 508.0536; found, 508.0471.

##### Ethyl (2-(2-Nitro-4-(piperidine-1-carbonyl)benzamido)thiophene-3-carbonyl)carbamate (**24e**)

White solid; yield 27%; mp 113–114 °C. ^1^H NMR (400 MHz, DMSO-*d*_6_) δ: 12.05 (brs, 1H), 10.79 (brs, 1H), 8.15 (m, 1H), 7.91–7.90 (m, 2H), 7.65 (d, *J* = 6.0 Hz, 1H), 7.15 (d, *J* = 6.0 Hz, 1H), 4.20–4.15 (m, 2H), 3.62 (brs, 2H), 3.32 (brs, 2H), 1.61 (brs, 4H), 1.49 (brs, 2H), 1.26 (t, *J* = 7.2 Hz, 3H). ^13^C NMR (100 MHz, DMSO-*d*_6_) δ: 165.9, 163.2, 162.5, 151.3, 147.4, 146.9, 139.9, 132.0, 130.6, 129.4, 123.6, 122.9, 117.3, 116.6, 61.3, 48.1, 42.6, 25.9, 25.2, 24.0, 14.3. HRMS (ESI): *m*/*z* [M + H]^+^ calcd for C_21_H_23_N_4_O_7_S, 475.1282; found, 475.1288.

##### Ethyl (2-(3-Fluoro-4-(piperidine-1-carbonyl)benzamido)thiophene-3-carbonyl)carbamate (**24f**)

Off white solid; yield 48%; mp 186–187 °C. ^1^H NMR (400 MHz, DMSO-*d*_6_) δ: 12.59 (brs, 1H), 10.84 (brs, 1H), 7.83–7.79 (m, 2H), 7.73 (d, *J* = 6.0 Hz, 1H), 7.69–7.65 (m, 1H), 7.12 (d, *J* = 6.0 Hz, 1H), 4.22–4.19 (m, 2H), 3.65–3.62 (m, 2H), 3.21–3.18 (m, 2H), 1.63–1.57 (m, 4H), 1.46 (brs, 2H), 1.28 (t, *J* = 7.2 Hz, 3H). ^13^C NMR (100 MHz, DMSO-*d*_6_) δ: 164.0, 162.8, 161.5, 157.6 (q, *J* = 246 Hz), 151.2, 148.7, 134.8 (q, *J* = 13 Hz), 129.7, 128.7 (*J* = 9 Hz), 123.7, 123.5, 117.1, 115.7, 115.1 (q, *J* = 24 Hz), 61.4, 47.6, 42.1, 26.1, 25.3, 23.9, 14.3. HRMS (ESI): *m*/*z* [M + H]^+^ calcd for C_21_H_23_FN_3_O_5_S, 448.1337; found, 448.1338.

##### Ethyl (2-(3-Methyl-4-(piperidine-1-carbonyl)benzamido)thiophene-3-carbonyl)carbamate (**24g**)

White solid; yield 9%; mp 103–104 °C. ^1^H NMR (150 MHz, DMSO-*d*_6_) δ: 12.58 (brs, 1H), 10.80 (brs, 1H), 7.84 (s, 1H), 7.79 (d, *J* = 7.8 Hz, 1H), 7.73 (d, *J* = 6.0 Hz, 1H), 7.42 (d, *J* = 7.2 Hz, 1H), 7.09 (d, *J* = 6.0 Hz, 1H), 4.23–4.21 (m, 2H), 3.68–3.59 (m, 2H), 3.10–3.09 (m, 2H), 2.32 (s, 3H), 1.60–1.56 (m, 4H), 1.43–1.39 (m, 2H), 1.28 (t, *J* = 7.2 Hz, 3H). ^13^C NMR (150 MHz, DMSO-*d*_6_) δ: 167.3, 164.1, 162.7, 151.2, 149.2, 141.2, 134.9, 131.9, 129.2, 126.5, 124.8, 123.4, 116.8, 115.2, 61.4, 47.2, 41.7, 26.1, 25.4, 24.0, 18.7, 14.3. HRMS (ESI): *m*/*z* [M + H]^+^ calcd for C_22_H_26_N_3_O_5_S, 444.1588; found, 444.1575.

##### Ethyl (2-(6-(Piperidine-1-carbonyl)-2-naphthamido)thiophene-3-carbonyl)carbamate (**24h**)

White solid; yield 22%; mp 177–178 °C. ^1^H NMR (500 MHz, DMSO-*d*_6_) δ: 12.75 (brs, 1H), 10.86 (brs, 1H), 8,63 (s, 1H), 8.24 (d, *J* = 8.5 Hz, 2H), 8.08 (s, 1H), 8.02 (d, *J* = 8.5 Hz, 1H), 7.75 (d, *J* = 5.5 Hz, 1H), 7.62 (d, *J* = 8.5 Hz, 1H), 7.12 (d, *J* = 5.5 Hz, 1H), 4.25–4.21 (m, 2H), 3.65 (brs, 2H), 3.35 (brs, 2H), 1.63 (brs, 4H), 1.49 (brs, 2H), 1.30 (t, *J* = 9.0 Hz, 3H). ^13^C NMR (100 MHz, DMSO-*d*_6_) δ: 168.4, 164.2, 163.1, 151.3, 149.3, 136.5, 134.3, 132.2, 130.0, 129.7, 129.6, 128.3, 125.9, 125.6, 123.8, 123.4, 116.9, 115.3, 61.4, 48.2, 42.5, 26.1, 25.4, 24.1, 14.3. HRMS (ESI): *m*/*z* [M + H]^+^ calcd for C_25_H_26_N_3_O_5_S, 480.1588; found, 480.1594.

##### Ethyl (2-(4′-(Piperidine-1-carbonyl)-[1,1′-biphenyl]-4-carboxamido)thiophene-3-carbonyl)carbamate (**24i**)

Light yellow solid; yield 83%; mp 187–188 °C. ^1^H NMR (400 MHz, DMSO-*d*_6_) δ: 12.70 (brs, 1H), 10.83 (brs, 1H), 8.06 (d, *J* = 8.0 Hz, 2H), 7.99 (d, *J* = 8.0 Hz, 2H), 7.84 (d, *J* = 8.0 Hz, 2H), 7.74 (d, *J* = 6.0 Hz, 1H), 7.51 (d, *J* = 8.0 Hz, 2H), 7.10 (d, *J* = 6.0 Hz, 1H), 4.25–4.20 (m, 2H), 3.60 (brs, 2H), 3.32 (brs, 2H), 1.63 (brs, 2H), 1.52 (brs, 4H), 1.29 (t, *J* = 7.2 Hz, 3H). ^13^C NMR (100 MHz, DMSO-*d*_6_) δ: 168.5, 164.2, 162.7, 151.3, 149.3, 143.7, 139.5, 136.5, 130.9, 128.0, 127.7, 127.6, 127.1, 123.4, 116.8, 115.1, 61.4, 48.1, 42.4, 26.1, 25.3, 24.1, 14.3. HRMS (ESI): *m*/*z* [M + H]^+^ calcd for C_27_H_28_N_3_O_5_S, 506.1477; found, 506.1756.

##### Ethyl (2-(5-(Piperidine-1-carbonyl)thiophene-2-carboxamido)thiophene-3-carbonyl)carbamate (**24j**)

Light yellow solid; yield 21%; mp 175–176 °C. ^1^H NMR (400 MHz, DMSO-*d*_6_) δ: 12.50 (brs, 1H), 10.82 (brs, 1H), 7.75–7.74 (m, 1H), 7.71 (d, *J* = 6.0 Hz, 1H), 7.49–7.48 (m, 1H), 7.10 (d, *J* = 6.0 Hz, 1H), 4.24–4.20 (m, 2H), 3.59 (brs, 4H), 1.64 (brs, 2H), 1.56 (brs, 4H), 1.29 (t, *J* = 7.2 Hz, 3H). ^13^C NMR (100 MHz, DMSO-*d*_6_) δ: 164.0, 161.0, 157.5, 151.2, 148.5, 143.6, 137.9, 129.5, 129.4, 123.4, 117.1, 115.4, 61.4, 47.8, 43.3, 25.8, 24.0, 14.3. HRMS (ESI): *m*/*z* [M + H]^+^ calcd for C_19_H_22_N_3_O_5_S_2_, 436.0995; found, 436.0985.

##### Ethyl (2-(5-(Piperidine-1-carbonyl)furan-3-carboxamido)thiophene-3-carbonyl)carbamate (**24k**)

White solid; yield 18%; mp 157–158 °C. ^1^H NMR (400 MHz, DMSO-*d*_6_) δ: 12.12 (brs, 1H), 10.78 (brs, 1H), 8.60 (s, 1H), 7.68 (d, *J* = 6.0 Hz, 1H), 7.22 (s, 1H), 7.07 (d, *J* = 6.0 Hz, 1H), 4.24–4.18 (m, 2H), 3.62 (brs, 4H), 1.65–1.64 (m, 2H), 1.57–1.56 (m, 4H), 1.28 (t, *J* = 6.8 Hz, 3H). ^13^C NMR (150 MHz, DMSO-*d*_6_) δ: 163.8, 158.1, 157.5, 151.3, 148.8, 148.4, 147.5, 123.4, 121.8, 116.9, 115.3, 112.2, 61.4, 47.3, 43.2, 26.3, 25.4, 24.1, 14.3. HRMS (ESI): *m*/*z* [M + H]^+^ calcd for C_19_H_22_N_3_O_6_S, 420.1224; found, 420.1217.

##### Ethyl (2-(4-(Piperidine-1-carbonyl)-1*H*-pyrrole-2-carboxamido)thiophene-3-carbonyl)carbamate (**24l**)

White solid; yield 8%; mp 183–184 °C. ^1^H NMR (400 MHz, DMSO-*d*_6_) δ: 12.42 (brs, 1H), 12.15 (brs, 1H), 10.74 (brs, 1H), 7.66 (d, *J* = 6.0 Hz, 1H), 7.01 (d, *J* = 6.0 Hz, 1H), 6.83–6.81 (m, 1H), 6.51–6.50 (m, 1H), 4.24–4.18 (m, 2H), 3.59–3.56 (m, 4H), 1.64–1.63 (m, 2H), 1.54–1.50 (m, 4H), 1.28 (t, *J* = 7.2 Hz, 3H). ^13^C NMR (150 MHz, DMSO-*d*_6_) δ: 163.9, 160.8, 156.5, 151.3, 149.2, 130.8, 125.6, 123.2, 116.3, 114.5, 112.0, 111.6, 61.3, 47.6, 43.1, 25.8, 24.2, 14.3. HRMS (ESI): *m*/*z* [M + H]^+^ calcd for C_19_H_23_N_4_O_5_S, 419.1384; found, 419.1370.

#### Procedure for the Synthesis of Target Compound **25a**

Target compound **25a** was prepared from **4j** (1.85 g, 7.93 mmol) and **16b** (1.32 g, 6.6 mmol) in the same manner as described for **23a**–**p**.

##### Methyl (2-(4-(Piperidine-1-carbonyl)benzamido)thiophene-3-carbonyl)carbamate (**25a**)

White solid; yield 13%; mp: 151–152 °C. ^1^H NMR (400 MHz, DMSO-*d*_6_) δ: 12.63 (brs, 1H), 10.85 (brs, 1H), 8.00 (d, *J* = 8.0 Hz, 2H), 7.73 (d, *J* = 6.0 Hz, 1H), 7.62 (d, *J* = 8.0 Hz, 2H), 7.11 (d, *J* = 6.0 Hz, 1H), 3.76 (s, 3H), 3.61 (brs, 2H), 3.25 (brs, 2H), 1.62 (brs, 4H), 1.47 (brs, 2H). ^13^C NMR (100 MHz, DMSO-*d*_6_) δ: 167.8, 164.0, 162.5, 151.8, 149.2, 140.8, 132.3, 127.6, 127.5, 123.3, 116.9, 115.2, 52.5, 48.0, 42.4, 26.0, 25.3, 24.1. HRMS (ESI): *m*/*z* [M + H]^+^ calcd for C_20_H_22_N_3_O_5_S, 416.1275; found, 416.1261.

#### Procedure for the Synthesis of Target Compound **25b**

Target compound **25b** was prepared from **7f** (326.7 mg, 1.3 mmol) and **16b** (200.0 mg, 1.0 mmol) in the same manner as described for **23a**–**p**.

##### Methyl (2-(3-Fluoro-4-(piperidine-1-carbonyl)benzamido)thiophene-3-carbonyl)carbamate (**25b**)

White solid; yield 51%; mp 159–160 °C. ^1^H NMR (400 MHz, DMSO-*d*_6_) δ: 12.58 (brs, 1H), 10.86 (brs, 1H), 7.82–7.78 (m, 2H), 7.72 (d, *J* = 6.0 Hz, 1H), 7.69–7.65 (m, 1H), 7.12 (d, *J* = 6.0 Hz, 1H), 3.75 (s, 3H), 3.63 (brs, 2H), 3.20 (brs, 2H), 1.61–1.57 (m, 4H), 1.46 (brs, 2H). ^13^C NMR (100 MHz, DMSO-*d*_6_) δ: 163.8, 162.7, 161.4, 157.5 (*J* = 246 Hz), 151.7, 148.7, 134.6 (*J* = 7 Hz), 129.6, 128.7 (*J* = 19 Hz), 123.6, 123.3, 117.1, 115.5, 115.0 (*J* = 24 Hz), 52.4, 47.5, 42.1, 26.0, 25.2, 23.9. HRMS (ESI): *m*/*z* [M + H]^+^ calcd for C_20_H_21_FN_3_O_5_S, 434.1181; found, 434.1177.

#### Procedure for the Synthesis of Target Compound **25c**

Target compound **25c** was prepared from **4j** (131 mg, 0.56 mmol) and **19** (80 mg, 0.38 mmol) in the same manner as described for **23a**–**p**.

##### *N*-(Ethylcarbamoyl)-2-(4-(piperidine-1-carbonyl)benzamido)thiophene-3-carboxamide (**25c**)

Yellow solid; yield 31%; mp 223–224 °C. ^1^H NMR (400 MHz, DMSO-*d*_6_) δ: 12.49 (brs, 1H), 10.43 (brs, 1H), 8.55–8.52 (m, 1H), 8.00 (d, *J* = 8.0 Hz, 2H), 7.82 (d, *J* = 6.0 Hz, 1H), 7.60 (d, *J* = 8.0 Hz, 2H), 7.11 (d, *J* = 6.0 Hz, 1H), 3.61 (brs, 2H), 3.30–3.25 (m, 4H), 1.62–1.58 (m, 4H), 1.47 (brs, 2H), 1.14 (t, *J* = 7.2 Hz, 3H). ^13^C NMR (100 MHz, DMSO-*d*_6_) δ: 167.8, 166.0, 162.6, 153.2, 148.8, 140.8, 132.5, 127.6, 127.4, 123.2, 117.2, 114.7, 48.0, 42.4, 34.3, 26.0, 25.3, 24.1, 15.1. HRMS (ESI): *m*/*z* [M + H]^+^ calcd for C_21_H_25_N_4_O_4_S, 429.1591; found, 429.1596.

#### Procedure for the Synthesis of Target Compound **25d**

To a solution of **25a** (50 mg, 0.12 mmol) in 30 mL of CH_3_OH was added 1 mol/L LiOH aqueous solution (0.6 mL). The reaction mixture was heated to reflux for 2 h and then concentrated. The residue was diluted with CH_2_Cl_2_ (30 mL), washed with water (30 mL) and brine (30 mL), dried over anhydrous Na_2_SO_4_, filtered, and concentrated. The residue was washed with n-hexane and dried to give target compound **25d**.

##### 2-(4-(Piperidine-1-carbonyl)benzamido)thiophene-3-carboxamide (**25d**)

White solid; yield 70%; mp 180–181 °C. ^1^H NMR (400 MHz, DMSO-*d*_6_) δ: 13.48 (brs, 1H), 8.08 (brs, 1H), 7.97 (d, *J* = 8.0 Hz, 2H), 7.67 (brs, 1H), 7.60 (d, *J* = 8.0 Hz, 2H), 7.51 (d, *J* = 6.0 Hz, 1H), 7.07 (d, *J* = 6.0 Hz, 1H), 3.61 (brs, 2H), 3.25 (brs, 2H), 1.61–1.57 (m, 4H), 1.47 (brs, 2H). ^13^C NMR (100 MHz, DMSO-*d*_6_) δ: 167.9, 167.4, 162.0, 146.2, 140.6, 132.7, 127.5, 127.3, 123.3, 116.8, 115.9, 48.1, 42.4, 26.0, 25.3, 24.1. HRMS (ESI): *m*/*z* [M + H]^+^ calcd for C_18_H_20_N_3_O_3_S, 358.1220; found, 358.1222.

#### General Procedure for the Synthesis of Target Compounds **25e**–**h**

Target compounds **25e**–**h** were prepared from **4j** (1.6 equiv) and the corresponding aminothiophene intermediates **11a**–**d** (1 equiv) in the same manner as described for **23a**–**p**.

##### Methyl 1-(2-(4-(Piperidine-1-carbonyl)benzamido)thiophene-3-carbonyl)azetidine-3-carboxylate (**25e**)

Yellow solid; yield 29%; mp 154–155 °C. ^1^H NMR (500 MHz, CDCl_3_) δ: 13.22 (brs, 1H), 8.12 (d, *J* = 7.5 Hz, 2H), 7.57 (d, *J* = 7.5 Hz, 2H), 7.08 (d, *J* = 5.5 Hz, 1H), 6.89 (d, *J* = 5.5 Hz, 1H), 4.75 (brs, 2H), 4.47 (brs, 2H), 3.84 (s, 3H), 3.78 (s, 2H), 3.63–3.60 (m, 1H), 3.35 (brs, 2H), 1.74–1.57 (m, 6H). ^13^C NMR (150 MHz, DMSO-*d*_6_) δ: 173.1, 168.2, 166.5, 162.4, 147.6, 141.0, 132.9, 127.9, 127.7, 123.3, 117.8, 114.3, 55.8, 52.6, 51.6, 48.4, 42.7, 32.6, 26.4, 25.7, 24.5. HRMS (ESI): *m*/*z* [M + H]^+^ calcd for C_23_H_26_N_3_O_5_S, 456.1588; found, 456.1549.

##### Methyl 1-(2-(4-(Piperidine-1-carbonyl)benzamido)thiophene-3-carbonyl)piperidine-4-carboxylate (**25f**)

Grey solid; yield 19%; mp 169–170 °C. ^1^H NMR (400 MHz, DMSO-*d*_6_) δ: 11.54 (brs, 1H), 7.94 (d, *J* = 8.0 Hz, 2H), 7.57 (d, *J* = 8.0 Hz, 2H), 7.15 (d, *J* = 5.6 Hz, 1H), 7.04 (d, *J* = 5.6 Hz, 1H), 4.09 (brs, 2H), 3.61 (brs, 5H), 3.25 (brs, 2H), 3.10 (brs, 2H), 2.71–2.65 (m, 1H), 1.91–1.89 (m, 2H), 1.62–1.47 (m, 8H). ^13^C NMR (150 MHz, DMSO-*d*_6_) δ: 174.8, 168.3, 165.7, 163.1, 142.9, 140.7, 133.4, 128.1, 127.5, 124.7, 119.5, 118.1, 55.4, 52.0, 48.4, 42.7, 38.7, 28.3, 26.4, 25.7, 24.5. HRMS (ESI): *m*/*z* [M + H]^+^ calcd for C_25_H_30_N_3_O_5_S, 484.1901; found, 484.1908.

##### Methyl 4-(2-(4-(Piperidine-1-carbonyl)benzamido)thiophene-3-carbonyl)piperazine-1-carboxylate (**25g**)

White solid; yield 17%; mp 113–114 °C. ^1^H NMR (400 MHz, DMSO-*d*_6_) δ: 11.58 (s, 1H), 7.94 (d, *J* = 8.0 Hz, 2H), 7.58 (d, *J* = 8.0 Hz, 2H), 7.17 (d, *J* = 5.6 Hz, 1H), 7.07 (d, *J* = 5.6 Hz, 1H), 3.62 (s, 3H), 3.61–3.59 (m, 6H), 3.48–3.45 (m, 4H), 3.24 (brs, 2H), 1.62–1.57 (m, 4H), 1.47 (brs, 2H). ^13^C NMR (150 MHz, DMSO-*d*_6_) δ: 167.8, 165.6, 162.7, 155.0, 142.9, 140.3, 132.9, 127.6, 127.1, 124.2, 118.4, 117.6, 52.5, 47.9, 43.2, 42.2, 25.9, 25.2, 24.0. HRMS (ESI): *m*/*z* [M + H]^+^ calcd for C_24_H_29_N_4_O_5_S, 485.1853; found, 485.1859.

##### Ethyl 4-(2-(4-(Piperidine-1-carbonyl)benzamido)thiophene-3-carbonyl)piperazine-1-carboxylate (**25h)**

White solid; yield 21%; mp 134–135 °C. ^1^H NMR (500 MHz, DMSO-*d*_6_) δ: 11.59 (brs, 1H), 7.94 (d, *J* = 7.0 Hz, 2H), 7.58 (d, *J* = 7.5 Hz, 2H), 7.17 (d, *J* = 5.0 Hz, 1H), 7.08 (d, *J* = 5.0 Hz, 1H), 4.06 (d, *J* = 7.0 Hz, 2H), 3.60 (brs, 6H), 3.47 (brs, 4H), 3.24 (brs, 2H), 1.62–1.46 (m, 6H), 1.19 (t, *J* = 6.5 Hz, 3H). ^13^C NMR (100 MHz, DMSO-*d*_6_) δ: 168.3, 166.0, 163.1, 155.1, 143.4, 140.8, 133.3, 128.1, 127.6, 124.7, 118.9, 118.1, 61.4, 48.4, 43.6, 42.7, 26.3, 25.7, 24.5, 15.0. HRMS (ESI): *m*/*z* [M + H]^+^ calcd for C_25_H_31_N_4_O_5_S, 499.2010; found, 499.2003.

#### General Procedure for the Synthesis of Target Compounds **25i**–**q**

To a solution of compounds **22a**–**h** (1 equiv) in anhydrous DMF were added the corresponding benzoic acid **4j** or **7f** (1.2 equiv), EDCI (1.3 equiv), HOBt (1.3 equiv), and Et_3_N (2.5 equiv) in turn. The reaction mixture was stirred for 24 h under argon at room temperature, then quenched with water, and extracted with EA thrice. The combined organic phase was washed with brine, dried over anhydrous Na_2_SO_4_, filtered, and evaporated *in vacuo*. The residue was purified by silica gel column chromatography (CH_2_Cl_2_/MeOH = 100/1) to give target compounds **25i**–**q**.

##### *N*-(5-Methylthiazol-2-yl)-2-(4-(piperidine-1-carbonyl)benzamido)thiophene-3-carboxamide (**25i)**

White solid; yield 32%; mp 236–238 °C. ^1^H NMR (400 MHz, DMSO-*d*_6_) δ: 12.74 (brs, 1H), 12.38 (brs, 1H), 8.00 (d, *J* = 6.8 Hz, 2H), 7.90 (s, 1H), 7.64 (d, *J* = 6.4 Hz, 2H), 7.26 (s, 1H), 7.15 (s, 1H), 3.62 (brs, 2H), 3.27 (brs, 2H), 2.39 (s, 3H), 1.62–1.48 (m, 6H). ^13^C NMR (150 MHz, DMSO-*d*_6_) δ: 167.8, 163.7, 162.3, 162.2, 147.8, 140.6, 132.5, 129.6, 127.4, 127.3, 126.7, 123.2, 117.0, 115.1, 48.0, 42.3, 26.0, 25.2, 24.0, 11.2. HRMS (ESI): *m*/*z* [M + H]^+^ calcd for C_22_H_23_N_4_O_3_S_2_, 455.1206; found, 455.1199.

##### *N*-(4-Methylpyrimidin-2-yl)-2-(4-(piperidine-1-carbonyl)benzamido)thiophene-3-carboxamide (**25j)**

Yellow solid; yield 29%; mp 177–178 °C. ^1^H NMR (500 MHz, DMSO-*d*_6_) δ: 13.01 (brs, 1H), 10.93 (brs, 1H), 8.66 (d, *J* = 4.5 Hz, 1H), 8.03 (d, *J* = 7.5 Hz, 2H), 7.88 (d, *J* = 5.5 Hz, 1H), 7.64 (d, *J* = 7.5 Hz, 2H), 7.24 (d, *J* = 4.5 Hz, 1H), 7.16 (d, *J* = 5.5 Hz, 1H), 3.64 (s, 2H), 3.28 (s, 2H), 2.52 (s, 3H), 1.64–1.49 (m, 6H). ^13^C NMR (100 MHz, DMSO-*d*_6_) δ: 167.2, 166.6, 163.0, 161.1, 156.8, 156.1, 146.8, 139.4, 131.2, 126.2, 122.3, 116.1, 115.6, 114.6, 46.8, 41.1, 24.8, 24.1, 22.8, 22.3. HRMS (ESI): *m*/*z* [M + H]^+^ calcd for C_23_H_24_N_5_O_3_S, 450.1594; found, 450.1591.

##### 2-(3-Fluoro-4-(piperidine-1-carbonyl)benzamido)-*N*-(4-methylpyrimidin-2-yl)thiophene-3-carboxamide (**25k**)

Yellow solid; yield 17%; mp 148–150 °C. ^1^H NMR (400 MHz, DMSO-*d*_6_) δ: 12.95 (brs, 1H), 10.91 (brs, 1H), 8.63 (d, *J* = 5.2 Hz, 1H), 7.85 (d, *J* = 5.6 Hz, 1H), 7.80 (t, *J* = 8.4 Hz, 2H), 7.66 (t, *J* = 7.6 Hz, 1H), 7.21 (d, *J* = 5.2 Hz, 1H), 7.15 (d, *J* = 6.0 Hz, 1H), 3.63 (brs, 2H), 3.19 (brs, 2H), 2.48 (s, 3H), 1.65–1.53 (m, 4H), 1.45 (brs, 2H). ^13^C NMR (100 MHz, DMSO-*d*_6_) δ: 168.9, 164.5, 163.2, 161.7, 158.4, 158.0 (*J* = 246 Hz), 157.7, 156.8, 148.1, 135.3 (*J* = 7 Hz), 130.1, 129.0 (*J* = 19 Hz), 124.0, 117.7, 117.4, 116.6, 115.4 (*J* = 23 Hz), 47.9, 42.5, 26.4, 25.7, 24.3, 24.0. HRMS (ESI): *m*/*z* [M + H]^+^ calcd for C_23_H_23_FN_5_O_3_S, 468.1500; found, 468.1497.

##### 2-(4-(Piperidine-1-carbonyl)benzamido)-*N*-(4-(trifluoromethyl)pyrimidin-2-yl)thiophene-3-carboxamide (**25l)**

Yellow solid; yield 31%; mp 182–183 °C. ^1^H NMR (400 MHz, DMSO-*d*_6_) δ: 12.76 (brs, 1H), 11.40 (brs, 1H), 9.15 (d, *J* = 5.2 Hz, 1H), 7.99 (d, *J* = 8.4 Hz, 2H), 7.85 (d, *J* = 6.0 Hz, 1H), 7.79 (d, *J* = 5.2 Hz, 1H), 7.61 (d, *J* = 8.4 Hz, 2H), 7.15 (d, *J* = 5.6 Hz, 1H), 3.60 (brs, 2H), 3.24 (brs, 2H), 1.61–1.46 (m, 6H). ^13^C NMR (100 MHz, DMSO-*d*_6_) δ: 168.2, 164.6, 162.9, 162.7, 158.7, 155.3 (q, *J* = 35 Hz), 149.0, 141.1, 132.8, 127.9, 127.8, 124.1, 120.9 (q, *J* = 274 Hz), 117.3, 116.0, 113.5, 48.4, 42.7, 26.4, 25.7, 24.5. HRMS (ESI): *m*/*z* [M + H]^+^ calcd for C_23_H_21_F_3_N_5_O_3_S, 504.1312; found, 504.1306.

##### *N*-(5-Methylpyrazin-2-yl)-2-(4-(piperidine-1-carbonyl)benzamido)thiophene-3-carboxamide (**25m)**

White solid; yield 47%; mp 177–179 °C. ^1^H NMR (500 MHz, CDCl_3_) δ: 12.82 (brs, 1H), 9.54 (brs, 1H), 8.21–8.18 (m, 2H), 8.10 (d, *J* = 7.5 Hz, 2H), 7.58 (d, *J* = 7.0 Hz, 2H), 7.21 (d, *J* = 5.0 Hz, 1H), 6.94 (d, *J* = 5.0 Hz, 1H), 3.75 (brs, 2H), 3.34 (brs, 2H), 2.58 (s, 3H), 1.71–1.55 (m, 6H). ^13^C NMR (100 MHz, DMSO-*d*_6_) δ: 168.2, 164.9, 162.7, 149.5, 148.4, 146.4, 142.2, 141.1, 137.7, 132.9, 127.9, 127.8, 123.7, 117.5, 115.6, 48.4, 42.7, 26.4, 25.7, 24.5, 20.9. HRMS (ESI): *m*/*z* [M + H]^+^ calcd for C_23_H_24_N_5_O_3_S, 450.1594; found, 450.1588.

##### *N*-(6-Methylpyridin-2-yl)-2-(4-(piperidine-1-carbonyl)benzamido)thiophene-3-carboxamide (**25n)**

White solid; yield 46%; mp 171–174 °C. ^1^H NMR (500 MHz, DMSO-*d*_6_) δ: 13.03 (brs, 1H), 10.63 (brs, 1H), 8.01–7.98 (m, 4H), 7.76 (t, *J* = 8.0 Hz, 1H), 7.62 (d, *J* = 7.5 Hz, 2H), 7.13 (d, *J* = 5.5 Hz, 1H), 7.07 (d, *J* = 7.0 Hz, 1H), 3.61 (brs, 2H), 3.25 (brs, 2H), 2.47 (s, 3H), 1.62–1.47 (m, 6H). ^13^C NMR (100 MHz, DMSO-*d*_6_) δ: 168.2, 165.0, 162.6, 157.2, 151.3, 148.0, 141.1, 138.8, 133.0, 127.9, 127.8, 123.8, 119.9, 117.2, 116.1, 113.3, 48.4, 42.7, 26.4, 25.7, 24.5, 24.0. HRMS (ESI): *m*/*z* [M + H]^+^ calcd for C_24_H_25_N_4_O_3_S, 449.1642; found, 449.1641.

##### *N*-(4-Methylpyridin-2-yl)-2-(4-(piperidine-1-carbonyl)benzamido)thiophene-3-carboxamide (**25o)**

White solid; yield 27%; mp 148–149 °C. ^1^H NMR (400 MHz, DMSO-*d*_6_) δ: 12.96 (brs, 1H), 10.61 (brs, 1H), 8.28 (d, *J* = 5.2 Hz, 1H), 8.02 (s, 1H), 8.00 (s, 2H), 7.95 (d, *J* = 6.0 Hz, 1H), 7.63 (dd, *J* = 6.8, 1.6 Hz, 2H), 7.13 (d, *J* = 6.0 Hz, 1H), 7.06 (dd, *J* = 5.2, 0.8 Hz, 1H), 3.61 (brs, 2H), 3.26 (brs, 2H), 2.39 (s, 3H), 1.63–1.48 (m, 6H). ^13^C NMR (100 MHz, DMSO-*d*_6_) δ: 168.2, 164.9, 162.8, 152.0, 149.4, 148.1, 147.0, 141.1, 133.0, 127.9, 127.8, 123.8, 121.8, 117.3, 116.7, 116.1, 48.4, 42.8, 26.5, 25.7, 24.5, 21.4. HRMS (ESI): *m*/*z* [M + H]^+^ calcd for C_24_H_25_N_4_O_3_S, 449.1642; found, 449.1641.

##### *N*-(4,6-Dimethylpyrimidin-2-yl)-2-(4-(piperidine-1-carbonyl)benzamido)thiophene-3-carboxamide (**25p)**

White solid; yield 32%; mp 189–190 °C. ^1^H NMR (400 MHz, DMSO-*d*_6_) δ: 12.94 (brs, 1H), 10.81 (brs, 1H), 7.99 (d, *J* = 8.0 Hz, 2H), 7.83 (d, *J* = 6.0 Hz, 1H), 7.61 (d, *J* = 8.0 Hz, 2H), 7.12 (d, *J* = 5.6 Hz, 1H), 7.09 (s, 1H), 3.60 (brs, 2H), 3.24 (brs, 2H), 2.43 (s, 6H), 1.61–1.46 (m, 6H). ^13^C NMR (100 MHz, DMSO-*d*_6_) δ: 168.3, 168.2, 164.6, 162.8, 157.5, 148.2, 141.1, 132.9, 127.9, 127.8, 123.9, 117.2, 117.0, 116.3, 48.4, 42.7, 26.4, 25.8, 24.5, 23.8. HRMS (ESI): *m*/*z* [M + H]^+^ calcd for C_24_H_26_N_5_O_3_S, 464.1751; found, 464.1751.

##### *N*-(5-Methylbenzo[*d*]thiazol-2-yl)-2-(4-(piperidine-1-carbonyl)benzamido)thiophene-3-carboxamide (**25q)**

White solid; yield 56%. mp > 250 °C. ^1^H NMR (500 MHz, DMSO-*d*_6_) δ: 12.71 (brs, 1H), 12.61 (brs, 1H), 8.02 (d, *J* = 7.0 Hz, 2H), 7.98 (s, 1H), 7.81 (s, 1H), 7.66 (d, *J* = 7.0 Hz, 3H), 7.29 (d, *J* = 8.0 Hz, 1H), 7.19 (s, 1H), 3.62 (brs, 2H), 3.27 (brs, 2H), 2.43 (s, 3H), 1.63–1.49 (m, 6H). ^13^C NMR (100 MHz, DMSO-*d*_6_) δ: 167.7, 163.7, 162.5, 157.1, 148.8, 146.5, 140.7, 133.3, 132.4, 131.9, 127.6, 127.4, 123.0, 121.3, 120.0, 117.4, 114.3, 48.0, 42.3, 25.9, 25.2, 24.0, 21.0. HRMS (ESI): *m*/*z* [M + H]^+^ calcd for C_26_H_25_N_4_O_3_S_2_, 505.1363; found, 505.1360.

### Biological Evaluation

#### MIC Determination

MICs against replicating *M. tuberculosis* H_37_Rv or clinical isolates were determined by the microplate Alamar blue assay (MABA) following the protocol as described previously.^24,25^ RFP, INH, and TCA1 were included as positive controls. *M. tuberculosis* H_37_Rv or isolated clinical strains were grown to the late log phase (70–100 Klett units) in Difco Middlebrook 7H9 Broth (Seebio) supplemented with 0.2% (v/v) glycerol, 0.05% Tween 80, and 10% (v/v) albumin–dextrose–catalase (Seebio) (7H9-ADC-TG). Cultures were centrifuged, washed twice, and then resuspended in phosphate-buffered saline. Suspensions were then passed through an 8 μm pore size filter to remove clumps, and aliquots were frozen at −80 °C. Twofold dilutions of compounds were prepared in 7H9-ADC-TG in a volume of 100 μL in 96-well clear-bottom microplates (BD). *M. tuberculosis* (100 μL containing 2 × 10^5^ CFU) was added to yield a final testing volume of 200 μL. The plates were incubated at 37 °C; on day 7 of incubation, 12.5 μL of 20% Tween 80 and 20 μL of Alamar blue were added to all wells. After incubation at 37 °C for 16–24 h, the fluorescence was read at an excitation of 530 nm and an emission of 590 nm. The MIC was defined as the lowest concentration resulting in a reduction in fluorescence of ≥90% relative to the mean of replicate bacterium-only controls.

#### Cytotoxicity Assay^24,25^

Vero cells and HepG2 cells were cultured in Roswell Park Memorial Institute (RPMI) 1640 medium supplemented with 10% fetal bovine serum (FBS). The cells were incubated in a humidified atmosphere of 5% CO_2_ at 37 °C. Stocks of cells were cultured in 25 cm^2^ tissue culture flasks and subcultured two to three times per week. Cytotoxicity testing was performed in a transparent 96-well microplate. Outer perimeter wells were filled with sterile water to prevent dehydration in experimental wells. The cells were incubated at 37 °C under 5% CO_2_ until confluency and then diluted with the culture medium to 4 × 10^5^ cells/mL. Threefold serial dilutions of the stock solutions resulted in final concentrations of 64 to 0.26 μg/mL in a final volume of 100 μL. After incubation at 37 °C for 48 h, the medium was removed and the monolayers were washed twice with 100 μL of warm Hanks balanced salt solution (HBSS). Warm medium (100 μL) and 10 μL of freshly made methyl-thiazolyldiphenyl-tetrazolium bromide (MTT) were added to each well, and then, the plates were incubated for 4 h, after which the absorbance was determined at 492 nm.

#### Antituberculosis Activity in Macrophages

The assays were performed as described previously using mouse J774A.1 macrophages.^26^ The final concentrations of selected compounds were 10 μg/mL and 5 μg/mL. The concentration of rifampicin (RFP) as the positive control was 5 μg/mL. All assays were performed in triplicate in at least three separate experiments.

#### DprE1 Inhibition Assay

DprE1 assays were performed as described previously.^27^ Briefly, assays were performed at 30 °C in 384-well black plates in buffer containing 50 mM *N*-(2-hydroxyethyl)piperazine-*N*′-ethanesulfonic acid (Hepes), pH 7.5, 100 mM NaCl, 1.5% (v/v) DMSO, 100 μM Tween-20, 2 μM FAD, and 50 μM resazurin, with variable concentrations of FPR and DprE1. Reactions were monitored by following an increase in fluorescence intensity (λ_ex_ = 530 nm, λ_em_ = 595 nm) associated with the formation of resorufin. For inhibition studies, DprE1 (1 μM) was measured by the resazurin assay with 1 mM FPR in the presence of different inhibitor concentrations. The IC_50_ values were obtained by plotting the initial velocities with inhibitor concentrations. The IC_50_ values were calculated using the software program GraphPad Prism.

#### MIC Assay against the Strain Overexpressing DprE1^27^

Mt-DprE1 was cloned into the plasmid pMV261 to generate pMV261-Mt-*dprE1* and introduced into *M. bovis* BCG Pasteur. This host–plasmid system permits the constitutive expression of target proteins. After the bacteria were incubated in the presence of the compound for 7 days, cell viability can be assessed by the ability of endogenous reductases to reduce resazurin to resorufin. As a proof of concept, cells transformed with pMV261-Mt-*dprE1* grew in the presence of TCA1 with a MIC >16-fold higher than that when cells were transformed with an empty vector. Cells transformed with pMV261-Mt-*dprE1* did not confer any growth advantage over cells transformed with the vector alone when cells were grown in the presence of INH.

#### Hepatocyte Stability Assay

The assay was performed with hepatocytes from male mice (BioIVT) and mixed humans (BioIVT) following the protocol described previously.^25^ The assay evaluated the metabolic stability of compounds in hepatocytes by measuring the amount of parent compounds remaining of the test samples.

#### Inhibition Evaluation on the hERG K^+^ Channel

Electrophysiology recording of the hERG channel current was carried out following the standard protocol as described previously.^28^ hERG current inhibition in the presence of five concentrations, including 30, 10, 3.0, 1.0, and 0.3 μM, was tested for IC_50_ determination. Dofetilide was also included as a positive control to ensure the accuracy and sensitivity of the test system. All experiments were performed in duplicate for IC_50_ determination. The compound with IC_50_ > 30 μM was generally considered to have a lower potential for hERG K^+^ channel inhibition.

#### Pharmacokinetic Studies in Mice^29^

All animal protocols were approved by the Institute Animal Care and Welfare Committee of Shanghai Bioduro Biologics Co., Ltd. The selected compounds **25a**, **24f**, and TCA1 were subjected to pharmacokinetic studies in Balb/c mice (male) weighing 26–27 g with three mice in the oral administration group and three mice in the intravenous injection group. The tested compound was formulated at a concentration of 5 mg/mL for a dose of 50 mg/kg given orally (p.o.) and at 1 mg/mL for a dose of 5 mg/kg given intravenously (i.v.). The tested compound was formulated with 0.5% carboxymethyl cellulose for p.o. administration and with a mix solution (10%DMSO/50%poly(ethylene glycol) (PEG)400/40%water) for i.v. administration. Blood samples were collected at 5, 15, 30 min, 1, 2, 4, 7, 24 h after oral dosing and i.v. administration. Plasma was harvested and stored at −80 °C until analyzed. The pharmacokinetic parameters were calculated using WinNonlin software version 6.3 based on noncompartmental analysis (Pharsight Corporation, Mountain View). The oral bioavailability was calculated as the ratio between the area under the curve (AUC) following intravenous administration corrected for dose (*F* = (AUC_p.o._ × dose_i.v._)/(AUC_i.v._ × dose_p.o._)).

#### *In Vivo* TB Infection Assay^30^

All animal protocols were approved by the Institute Animal Care and Welfare Committee of Beijing Tuberculosis and Thoracic Tumor Research Institute, Beijing Chest Hospital, Capital Medical University. SPF Balb/c mice (female, 18–20 g) were used in this study. Each treated group was composed of six mice. Mice were infected *via* aerosol with a suspension of 5 × 10^6^ CFU/mL *M. tuberculosis* H_37_Rv using a Glas-Col inhalation system to deposit 50–100 bacilli into the lungs of each animal. The course of infection was followed by plating homogenates of harvested organs [*n* = 3] on 7H11 agar plates (7H11 plates containing 10% oleic acid–albumin–dextrose–catalase (OADC) enrichment and 50 μg/mL cycloheximide, 200 U/mL polymyxin B, 50 μg/mL carbenicillin, and 20 μg/mL trimethoprim) and determining CFUs on days 3, 10, and 30 postinfection. INH, TCA1, and **25a** were dissolved or suspended in 0.5% CMC and administered by oral gavage in a maximum volume of 200 μL such that doses of 25 and 100 mg/kg body weight were achieved. The control group received only 0.5% CMC. Mice were treated 5/7 days per week during the acute phase of infection, from day 10 to 30. Mice were sacrificed the day after the last day of treatment, and lungs were removed, homogenized, and serially diluted in 10-fold steps in HBSS. A total of 100 μL was spread on 7H11 agar in duplicate. The plates were incubated at 37 °C for 3 weeks. Data are expressed as log_10_ (and as log_10_ reduction) provided by the given dose of the compound against the growth of the organism in the untreated control group. Mean log_10_ values were calculated from bacterial burden counts. Student’s *t* test was used to compare means between the test and control groups. A *P* value of ≤0.05 was considered significant.

## Supporting Information Available

^1^H NMR and ^13^C NMR spectra of the target compounds and molecular docking studies of compound **23j** in DprE1 (PDF)
Molecular formula strings (CSV)

## Abbreviations

(Boc)_2_O: di-*tert*-butyl decarbonate
CFU: colony-forming unit
DMAP: 4-dimethylaminopyridine
DprE1: decaprenylphosphoryl-β-d-ribose 2′-epimerase
DMF: *N,N*-dimethylformamide
DMSO: dimethyl sulfoxide
DprE2: decaprenylphosphoryl-d-2-ketoerythropentose reductase
EA: ethyl acetate
EDCI: 1-(3-dimethylaminopropyl)-3-ethylcarbodiimide hydrochloride
ESI: electrospray ionization
FAD: flavin adenine dinucletide
HATU: 2-(7-aza-1*H*-benzotriazole-1-yl)-1,1,3,3-tetramethyluronium hexafluorophosphate
HBA: hydrogen-bonding acceptor
HOBt: 1-hydroxybenzotriazole
HepG2: human hepatocellular carcinoma
IC_50_: half maximal inhibitory concentration
LC–MS: liquid chromatography–mass spectrometry
*M. tuberculosis*: *Mycobacterium tuberculosis*
MABA: microplate Alamar blue assay
MDR-TB: multidrug-resistant tuberculosis
MIC: minimum inhibitory concentration
NaOH: sodium hydroxide
NMR: nuclear magnetic resonance
OE: overexpressor
PE: petroleum ether
PK: pharmacokinetic
rt: room temperature
SAR: structure–activity relationship
SI: selectivity index
TFA: trifluoroacetic acid
TB: tuberculosis
WT: wild type
XDR-TB: extensively drug-resistant tuberculosis
