# Supplementary material for: Discovery of Novel Thiophene-arylamide Derivatives as DprE1 Inhibitors with Potent Antimycobacterial Activities
Source: J Med Chem. 2021 Apr 14;64(9):6241–61. doi: 10.1021/acs.jmedchem.1c00263 (PMC8154581; doi:10.1021/acs.jmedchem.1c00263)
Supplement: Supplementary file 1 — jm1c00263_si_001.pdf [file jm1c00263_si_001.pdf]

Supporting Information

Discovery of Novel Thiophene-arylamide Derivatives as DprE1 Inhibitors with Potent Antimycobacterial Activities

Pengxu Wang,[||,†] *Sarah M. Batt,*[||,‡] *Bin Wang,*[§] *Lei Fu,*[§] *Rongfei Qin,*[†] *Yu Lu,*[§] *Gang Li,*[*,†] *Gurdyal S. Besra,*[*,‡] *and Haihong Huang*[*,†]

[†]Beijing Key Laboratory of Active Substance Discovery and Druggability Evaluation & Chinese Academy of Medical Sciences Key Laboratory of Anti-DR TB Innovative Drug Research, Institute of Materia Medica, Peking Union Medical College and Chinese Academy of Medical Sciences, 1 Xian Nong Tan Street, Beijing 100050, P. R. China

[‡]School of Biosciences, University of Birmingham, Birmingham B15 2TT, United Kingdom

[§]Beijing Key Laboratory of Drug Resistance Tuberculosis Research, Department of Pharmacology, Beijing Tuberculosis and Thoracic Tumor Research Institute, Beijing Chest Hospital, Capital Medical University, 97 Ma Chang Street, Beijing 101149, P. R. China

E-mail: ligang@imm.ac.cn, g.besra@bham.ac.uk, joyce@imm.ac.cn

List of Contents:

Title page……….…………………..….………………….....…..…………......…..S1

^{1}H NMR and ^{13}C NMR spectra of target compounds……………………………S2-46

Molecular docking………..……………….……………….…………………….………S47

^{1}H NMR and ^{13}C NMR spectra of target compounds

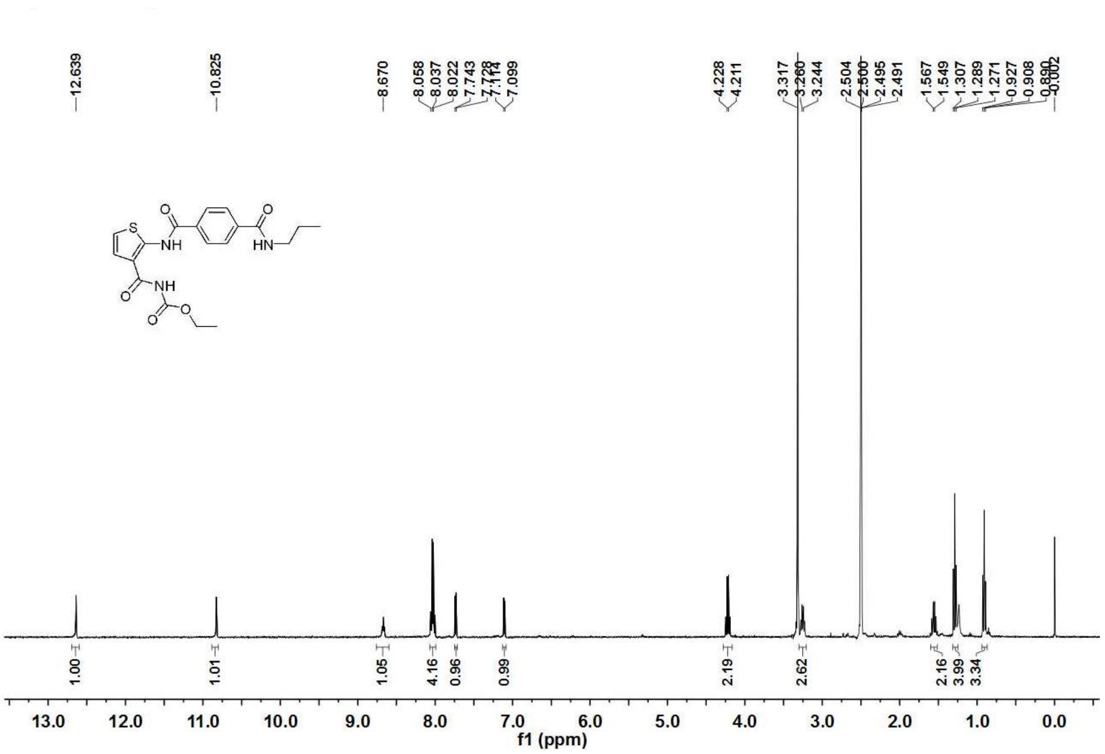

Figure S1. ^{1}H NMR spectrum of **23a**.

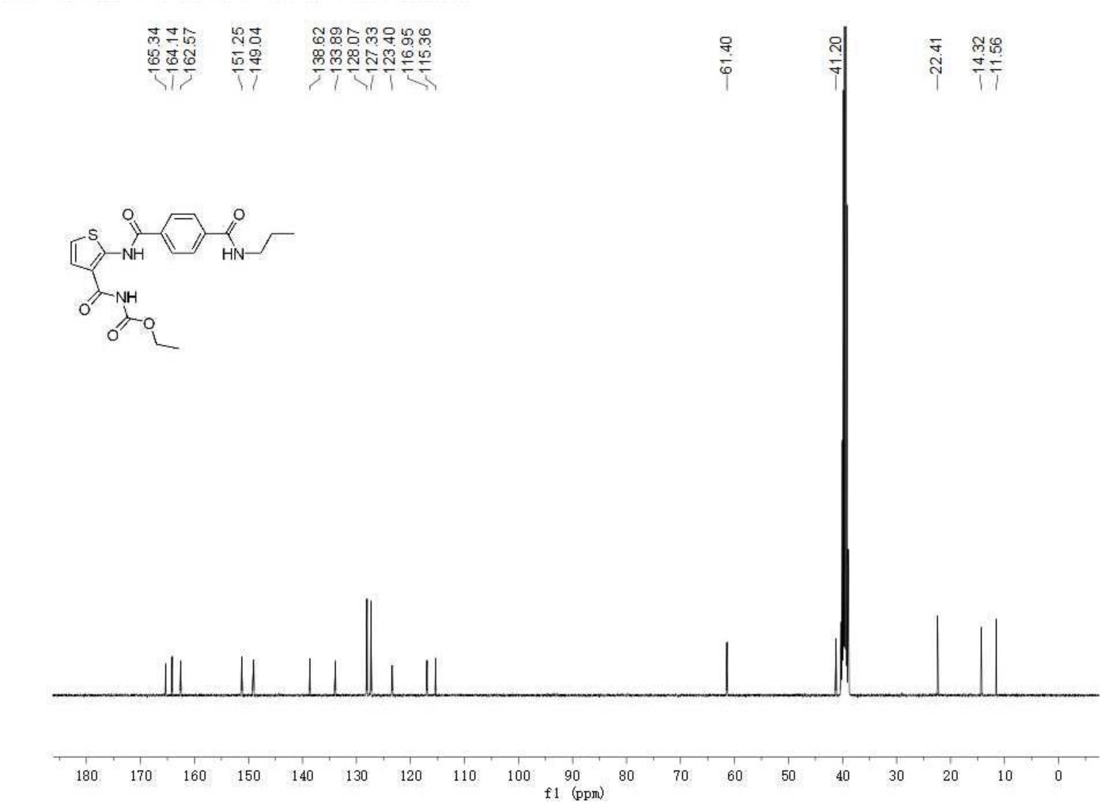

Figure S2. ^{13}C NMR spectrum of **23a**.

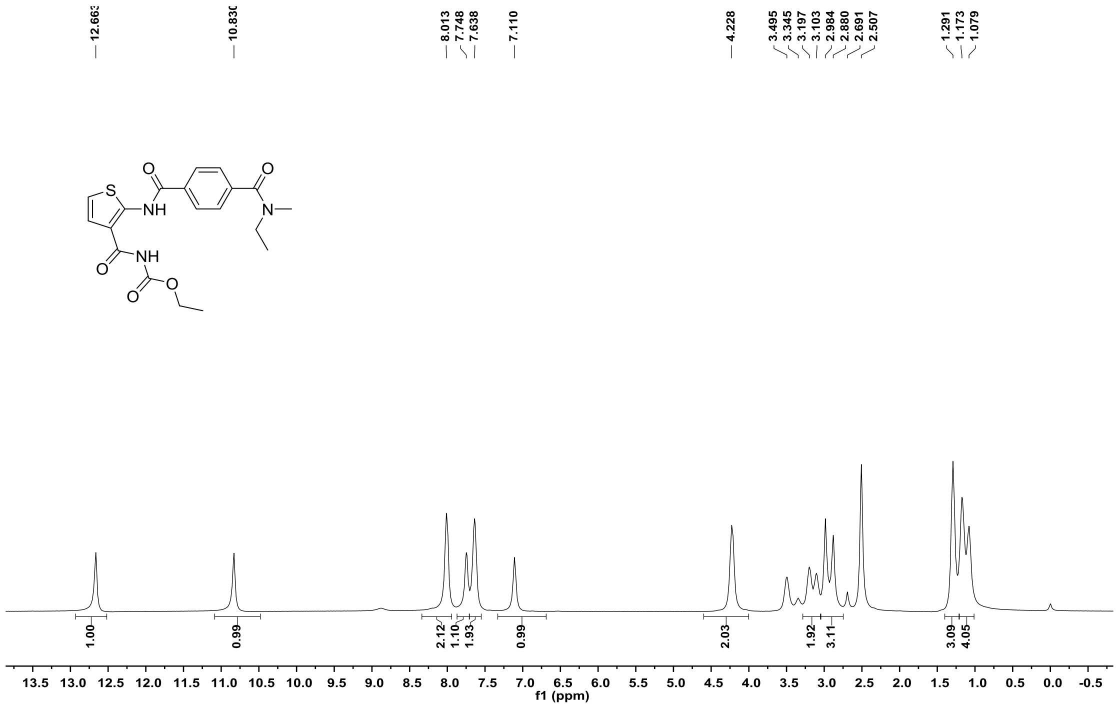

Figure S3. ^{1}H NMR spectrum of **23b**.

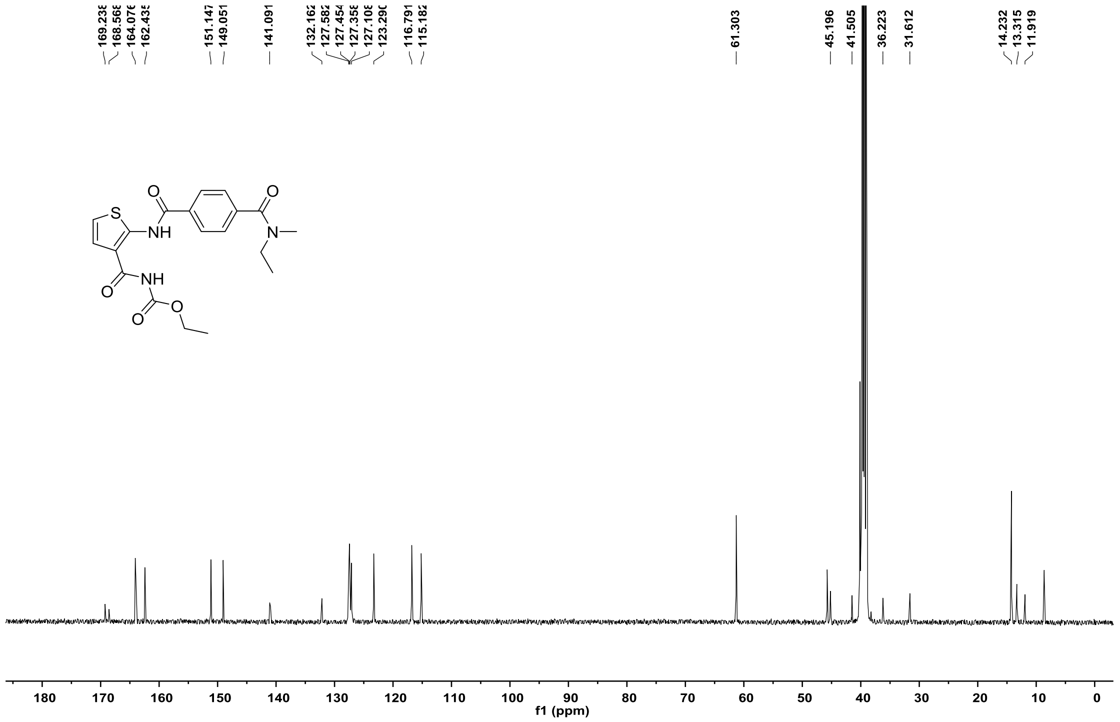

Figure S4. ^{13}C NMR spectrum of **23b**.

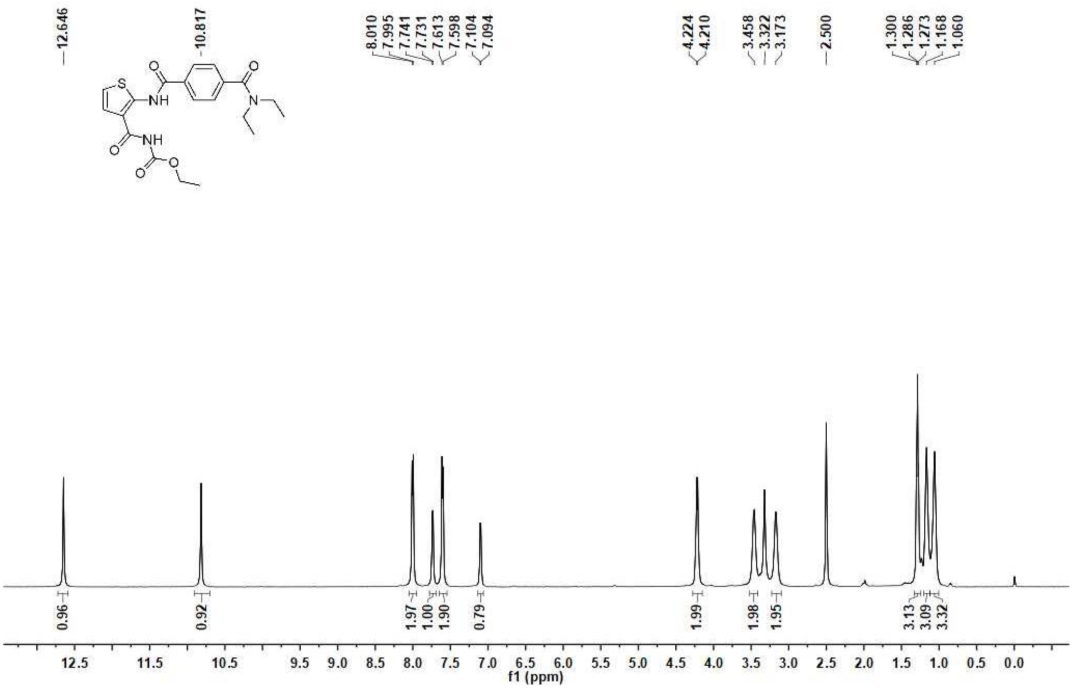

Figure S5. ^{1}H NMR spectrum of **23c**.

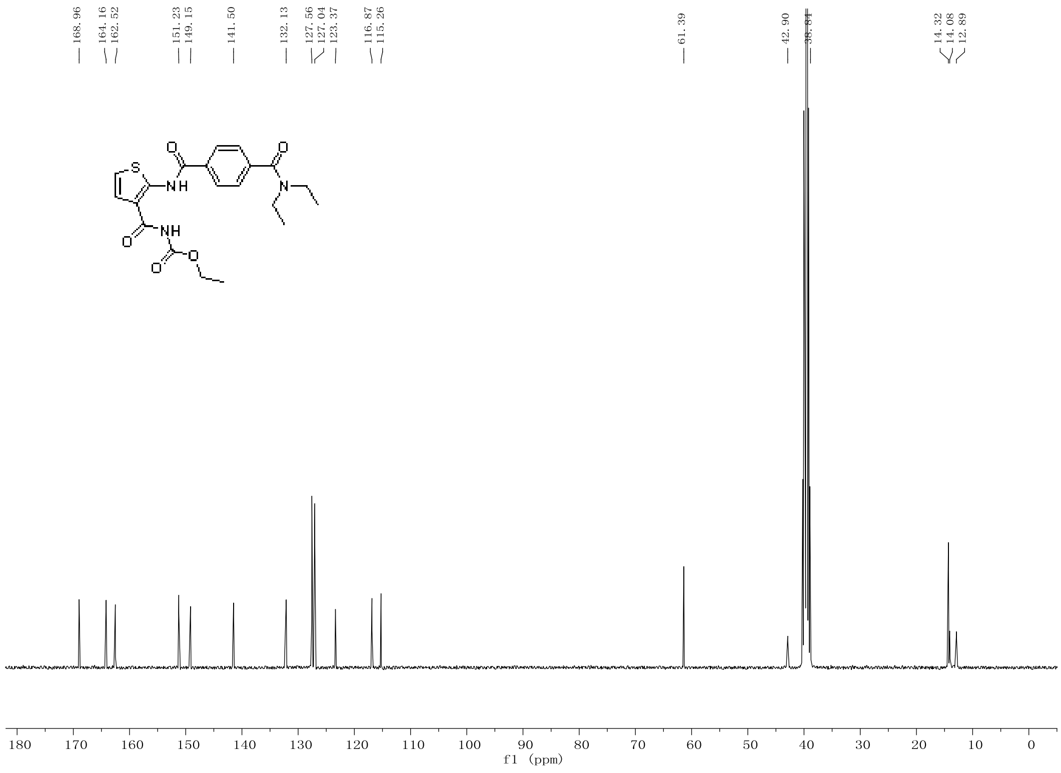

Figure S6. ^{13}C NMR spectrum of **23c**.

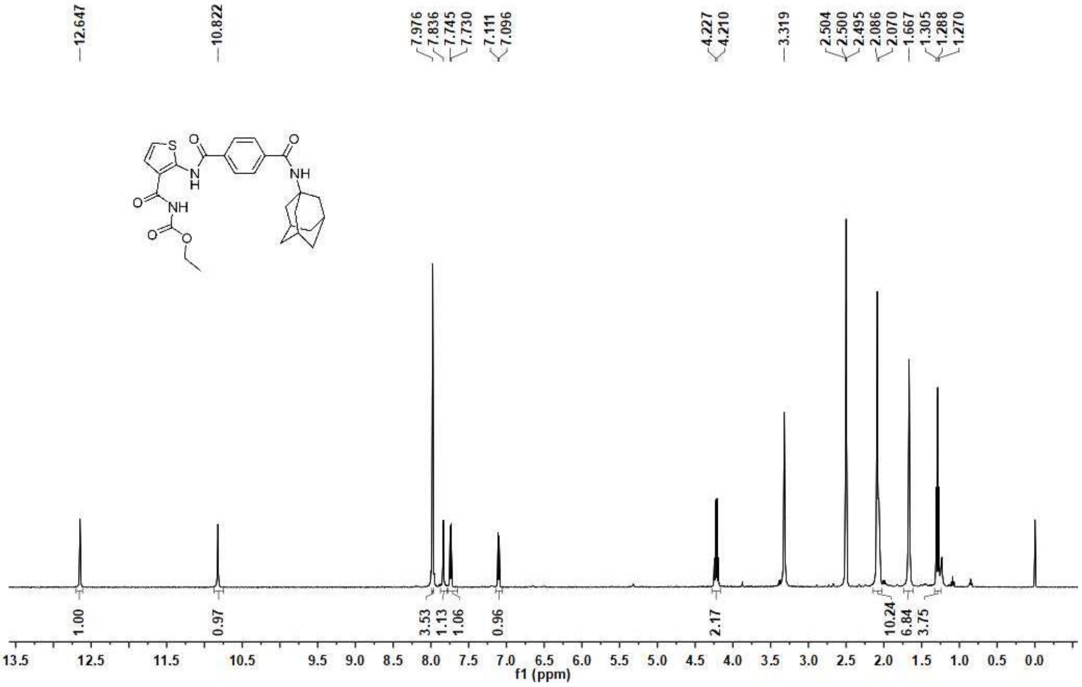

Figure S7. ^{1}H NMR spectrum of **23d**.

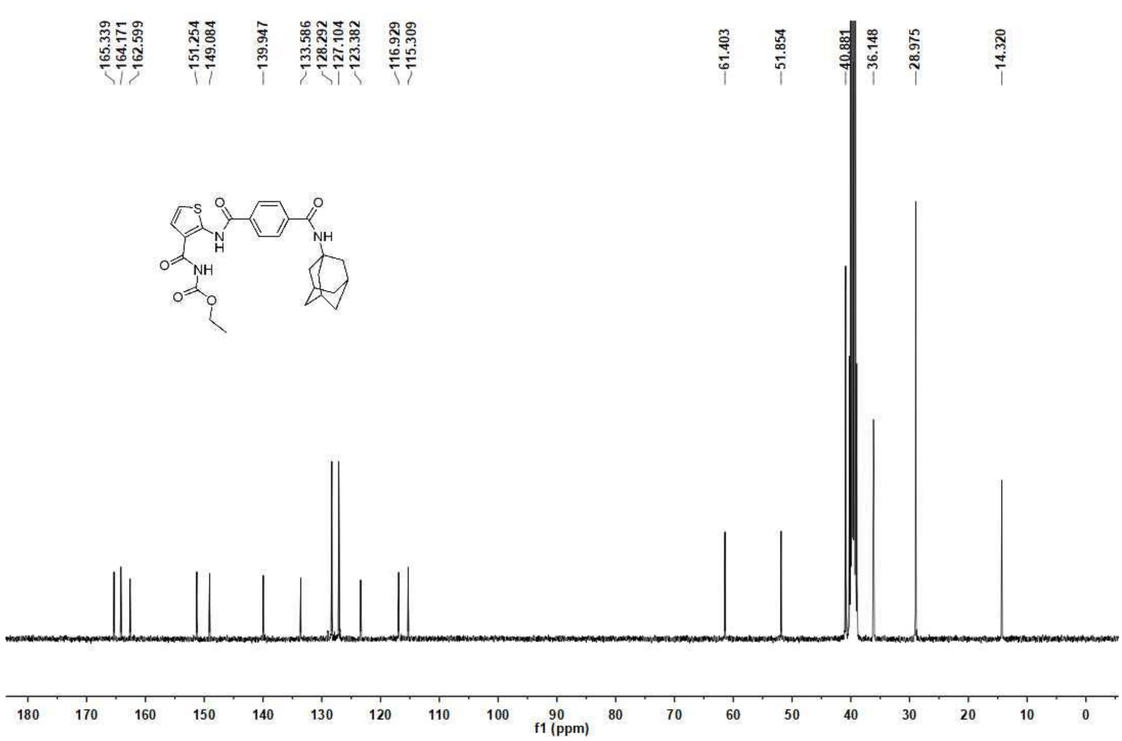

Figure S8. ^{13}C NMR spectrum of **23d**.

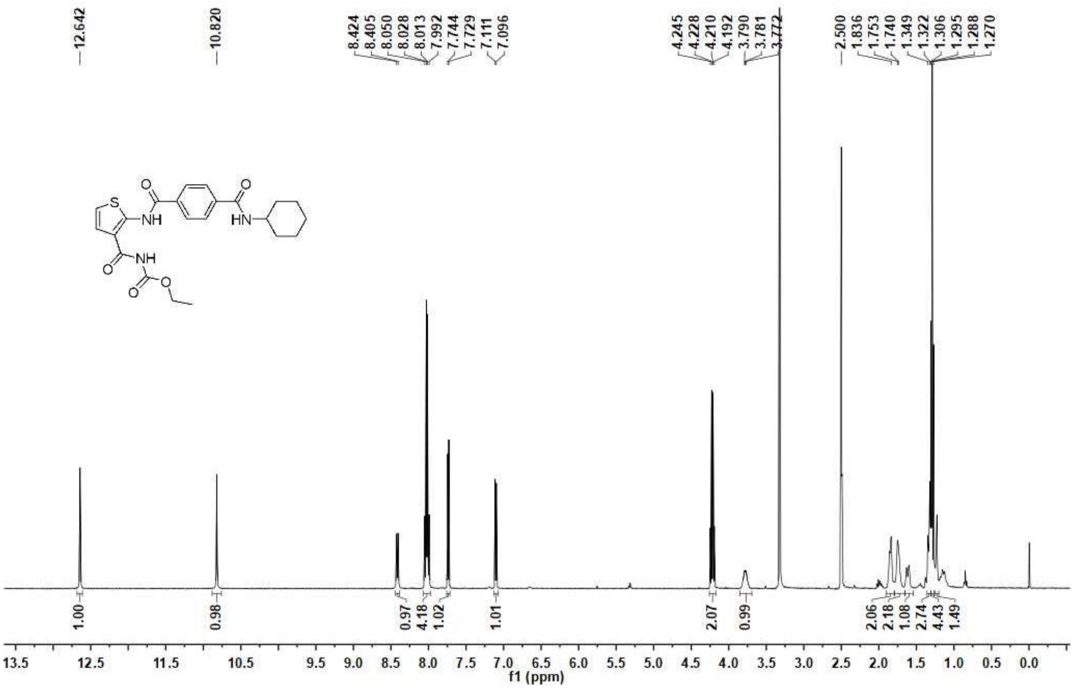

Figure S9. ^{1}H NMR spectrum of **23e**.

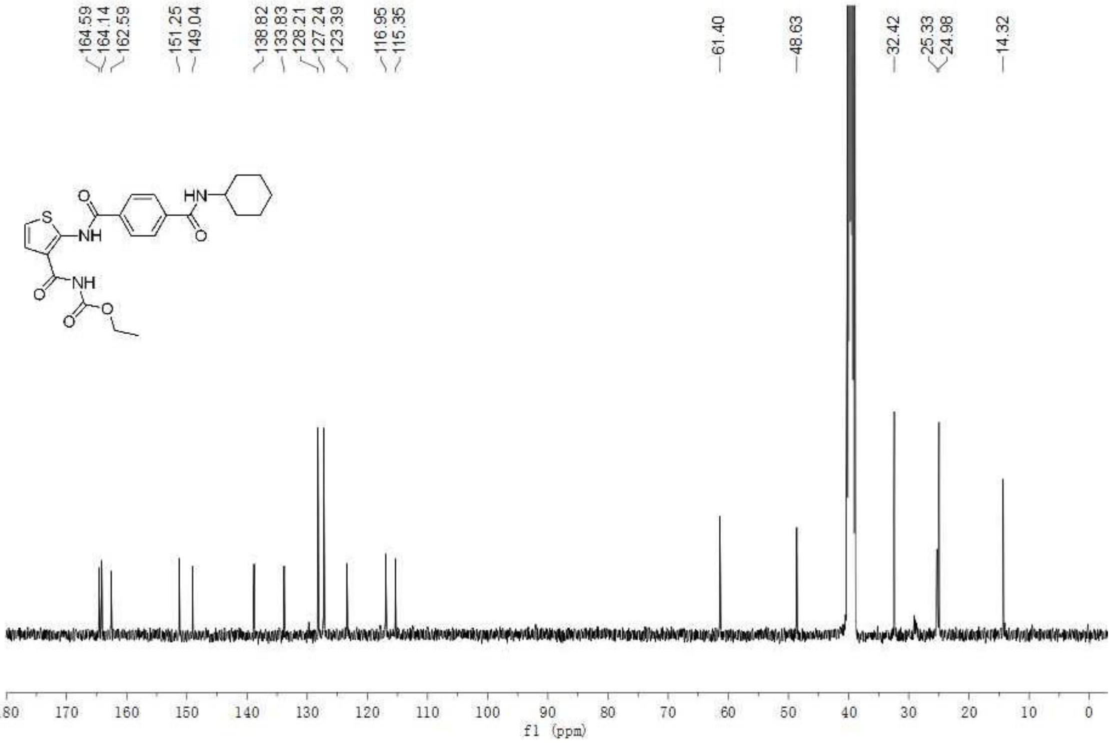

Figure S10. ^{13}C NMR spectrum of **23e**.

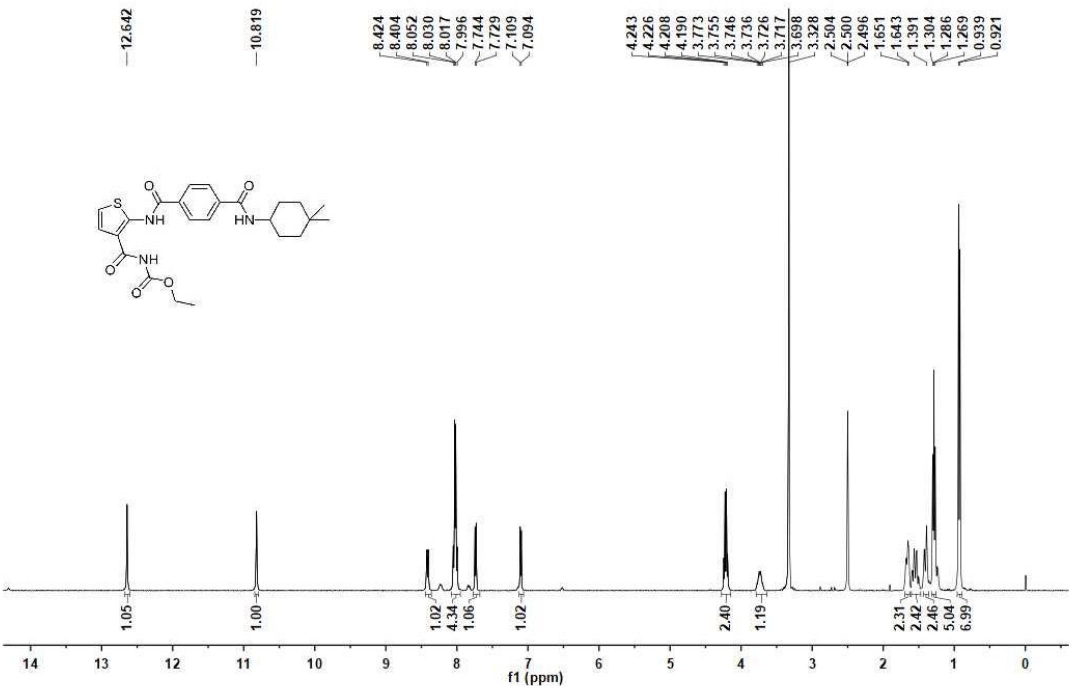

Figure S11. ^{1}H NMR spectrum of **23f**.

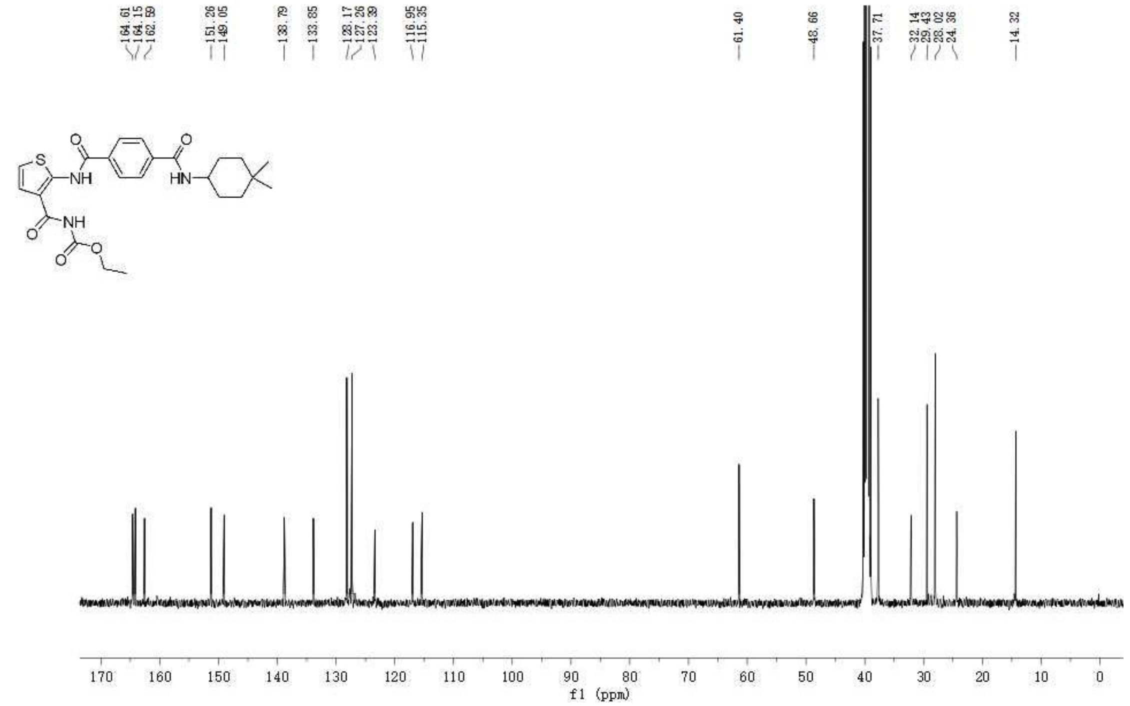

Figure S12. ^{13}C NMR spectrum of **23f**.

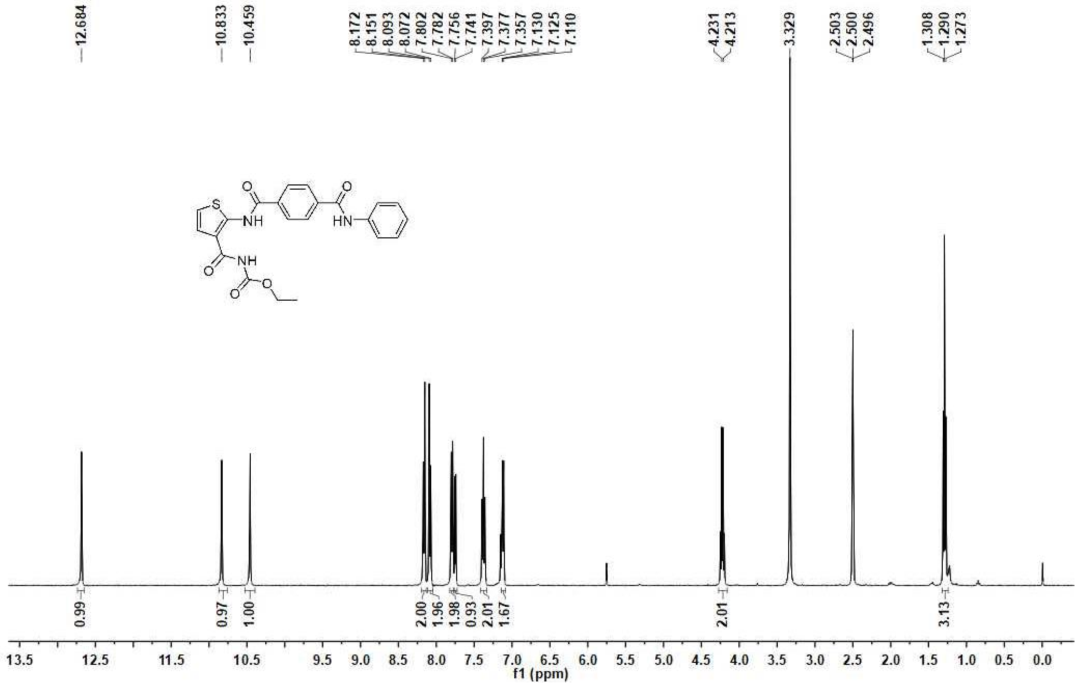

Figure S13. ^{1}H NMR spectrum of **23g**.

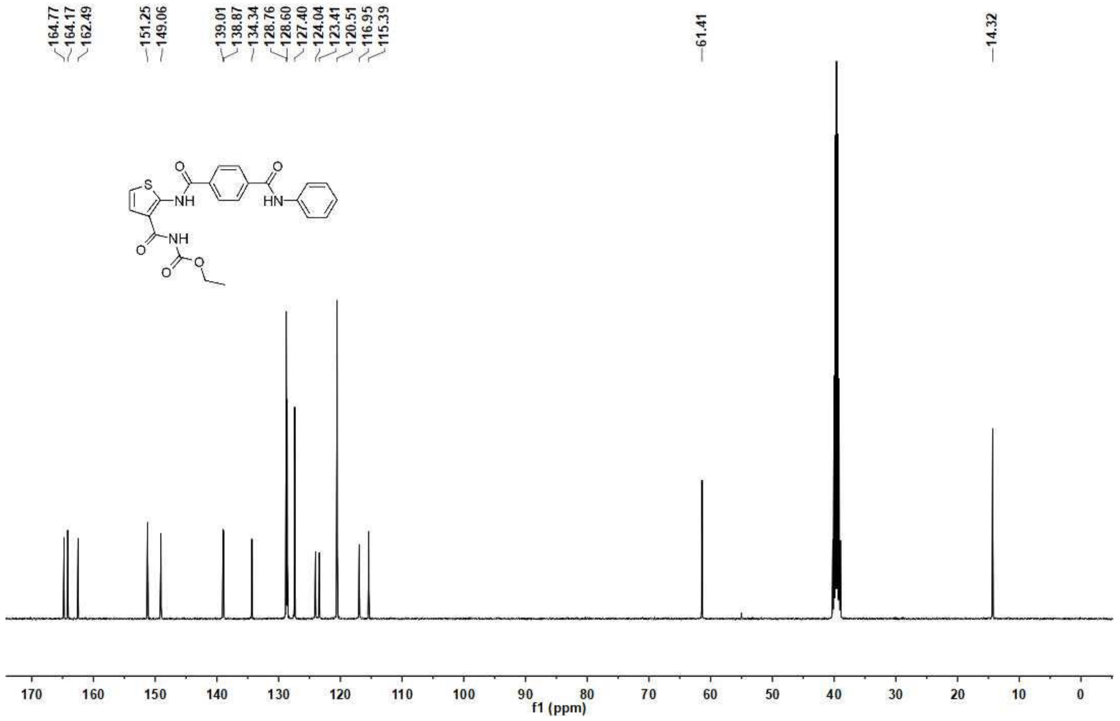

Figure S14. ^{13}C NMR spectrum of **23g**.

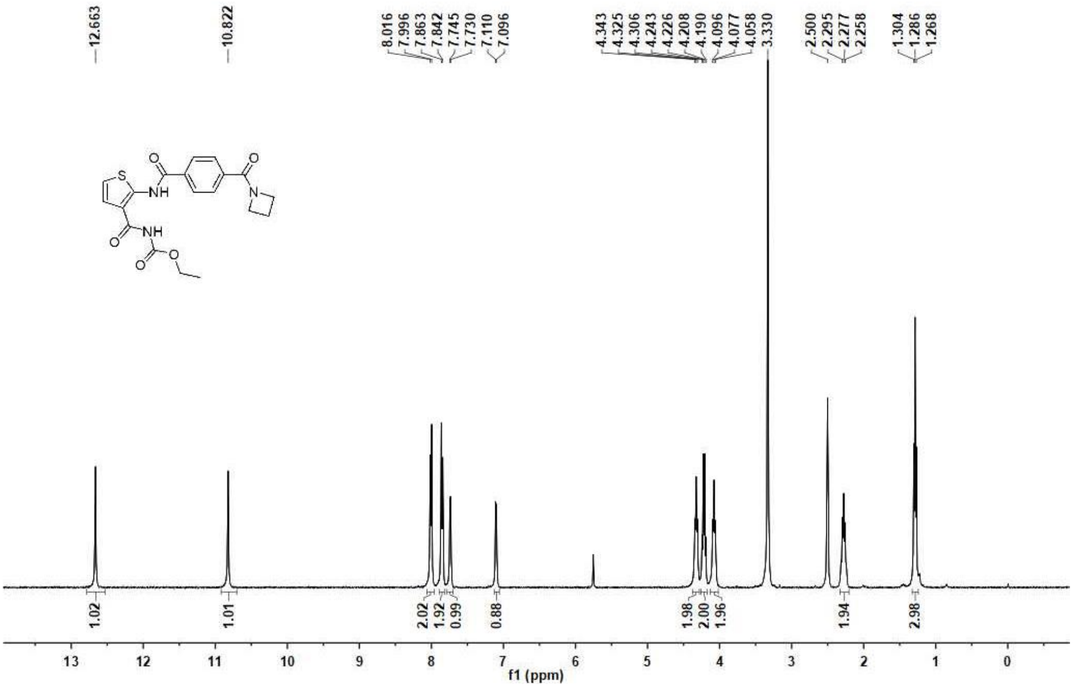

Figure S15. ^{1}H NMR spectrum of **23h**.

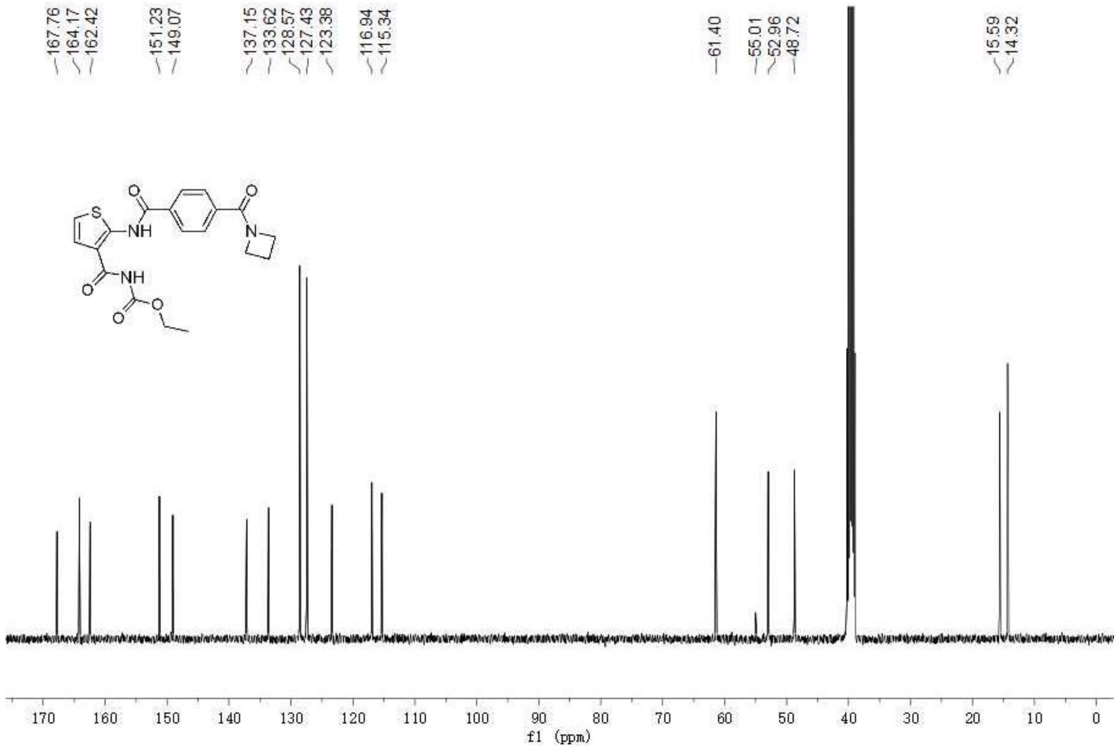

Figure S16. ^{13}C NMR spectrum of **23h**.

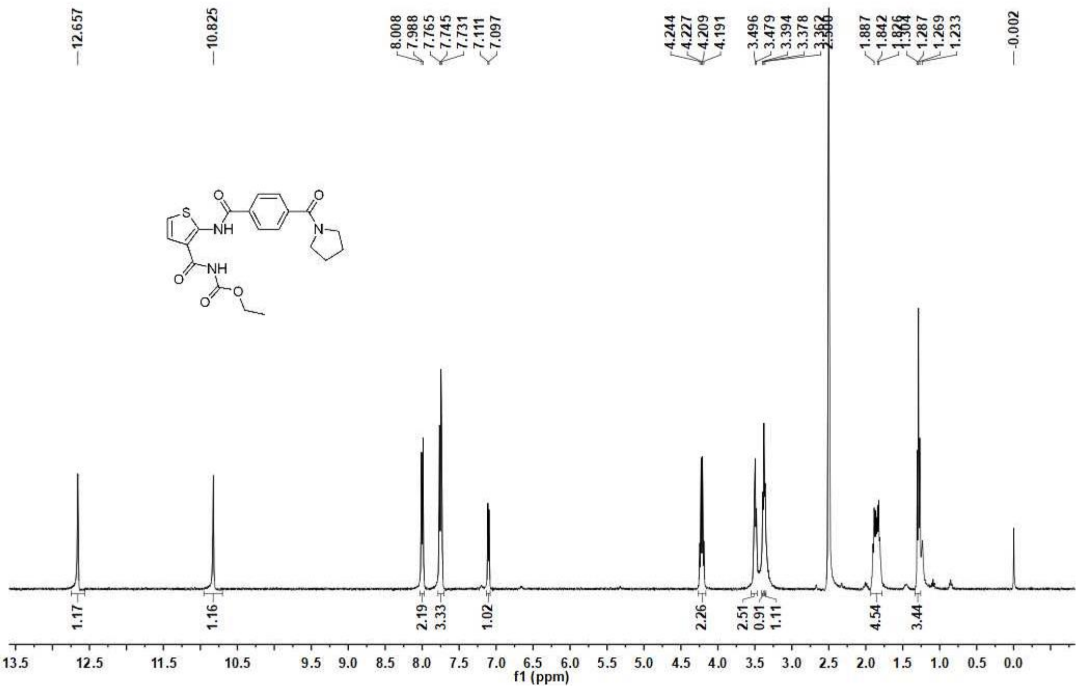

Figure S17. ^{1}H NMR spectrum of **23i**.

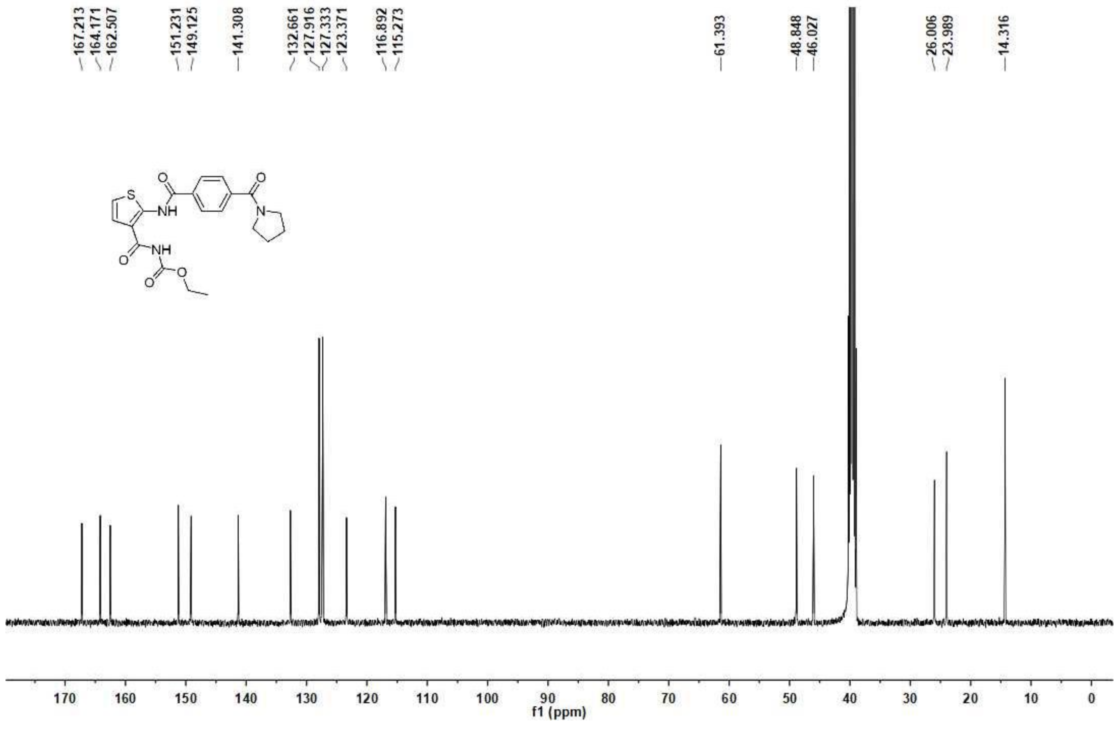

Figure S18. ^{13}C NMR spectrum of **23i**.

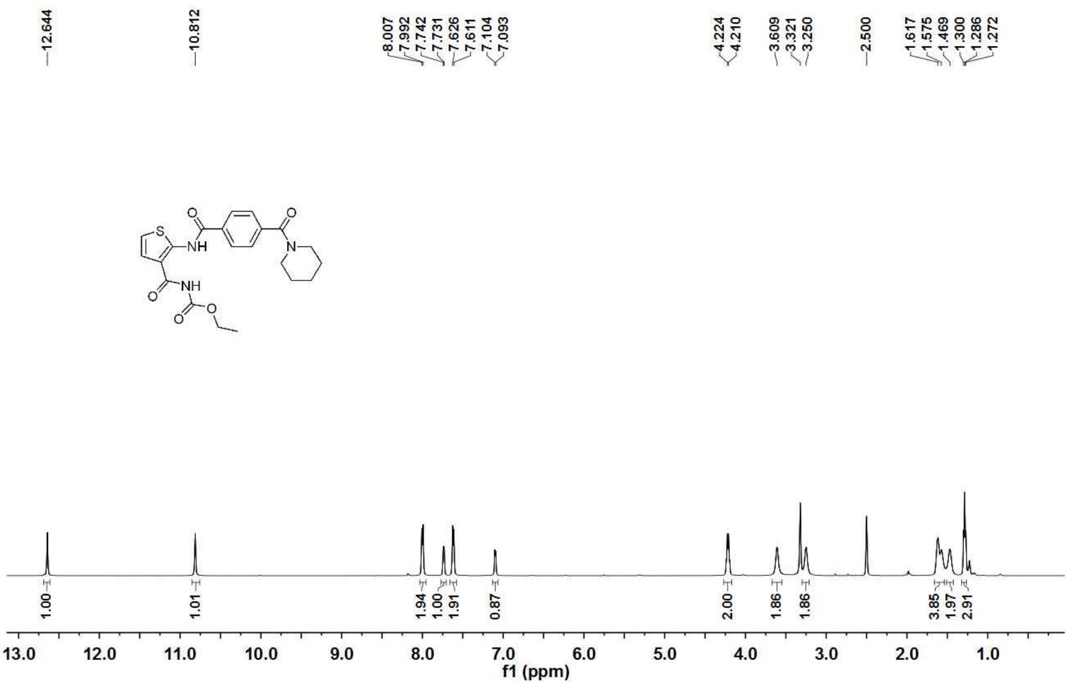

Figure S19. ^{1}H NMR spectrum of **23j**.

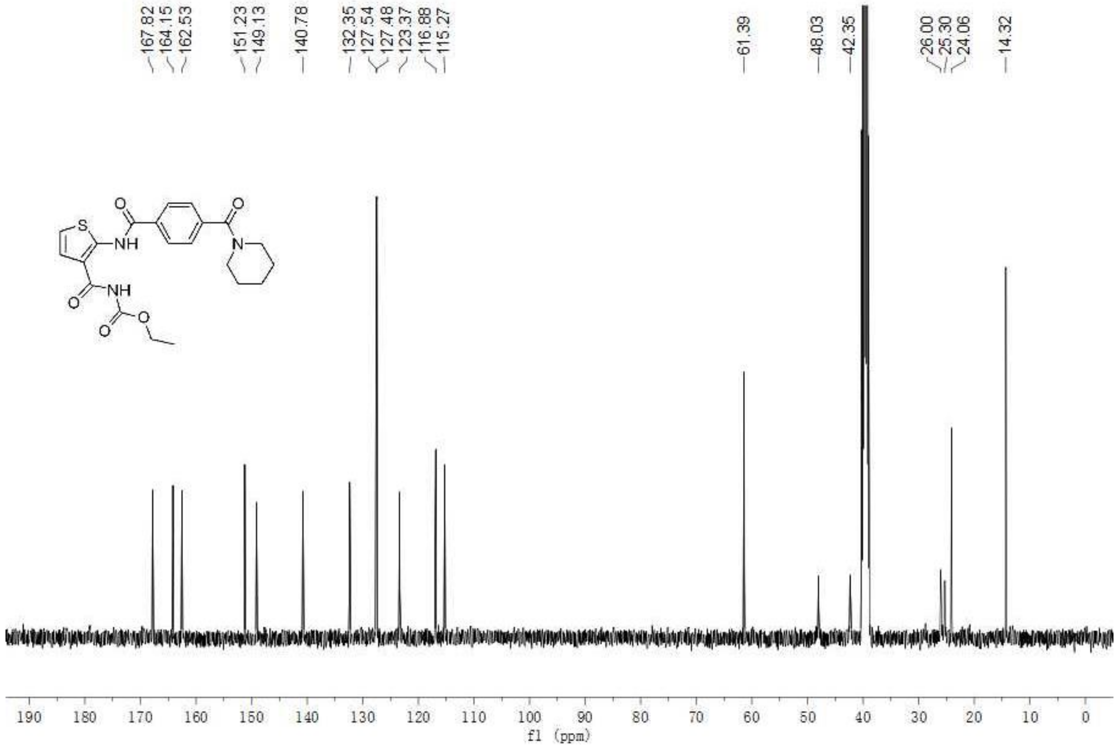

Figure S20. ^{13}C NMR spectrum of **23j**.

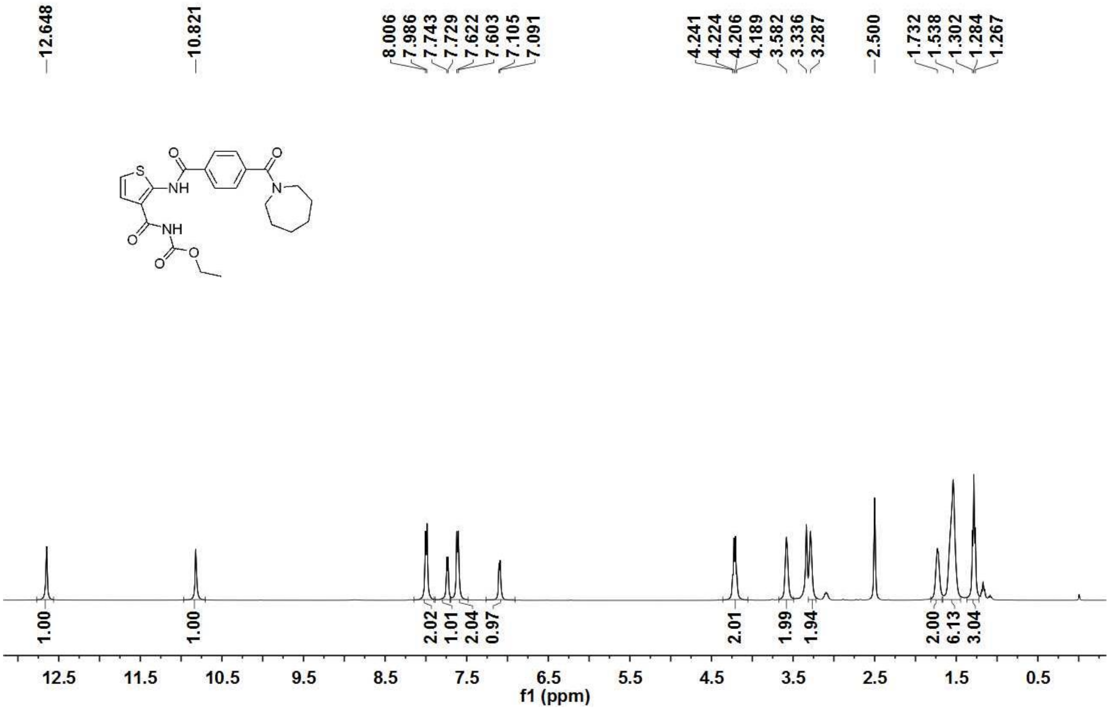

Figure S21. ^{1}H NMR spectrum of **23k**.

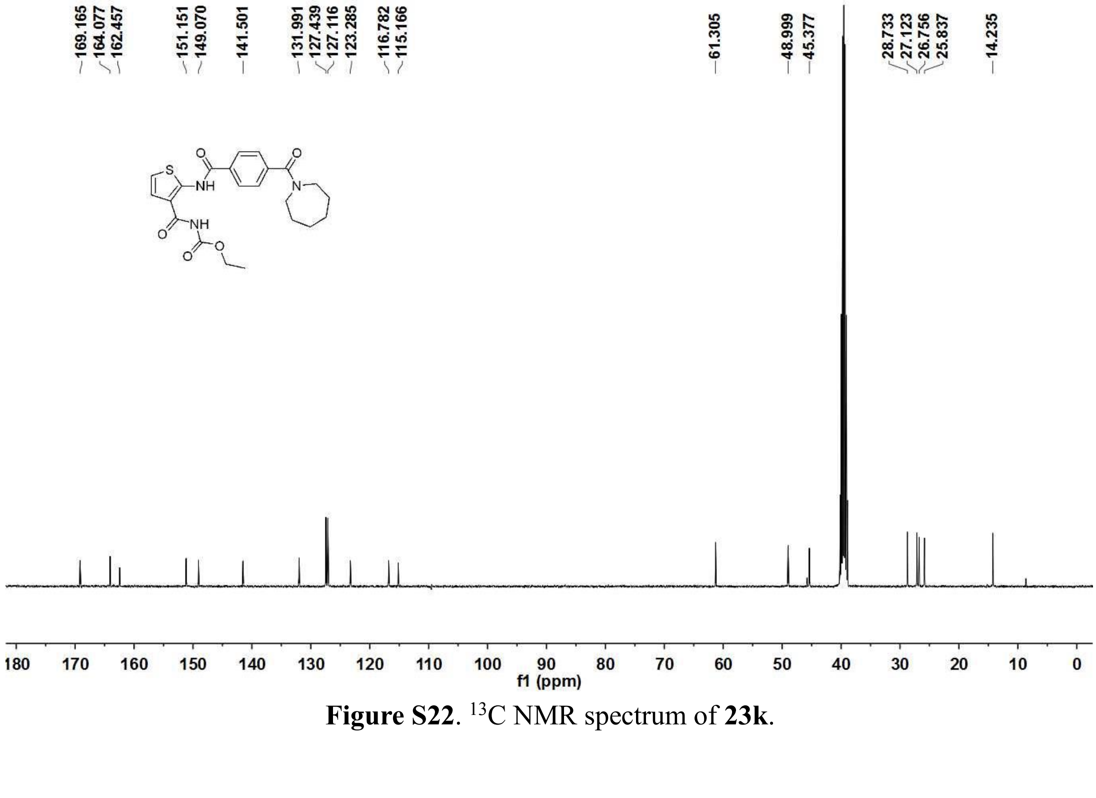

Figure S22. ^{13}C NMR spectrum of **23k**.

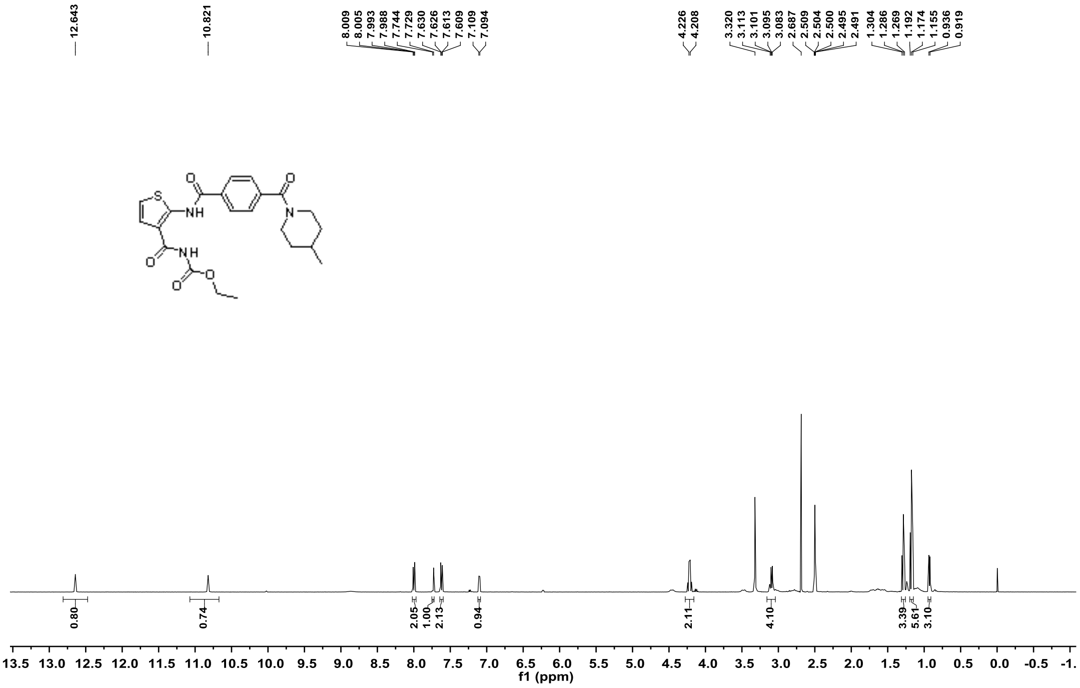

Figure S23. ^{1}H NMR spectrum of **23l**.

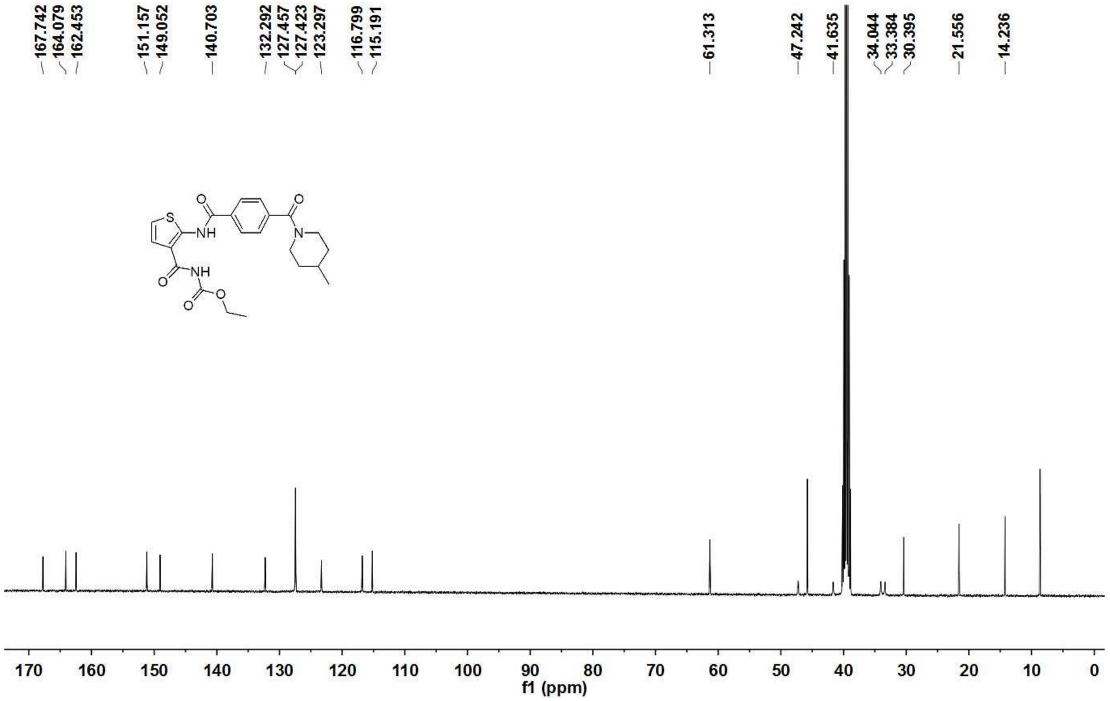

Figure S24. ^{13}C NMR spectrum of **23l**.

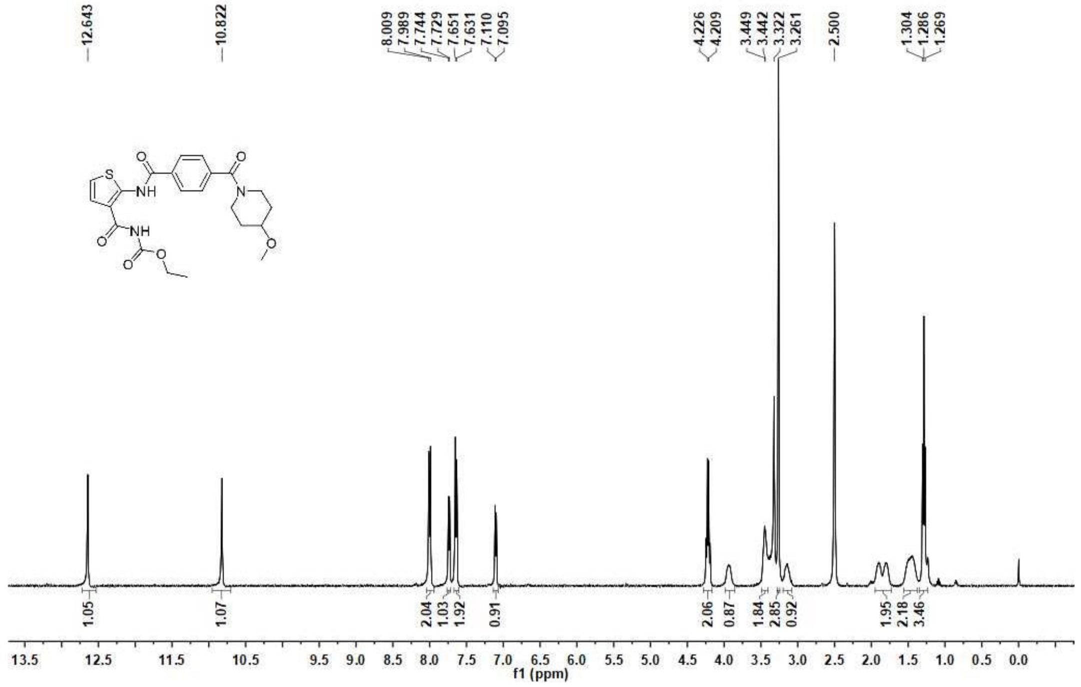

Figure S25. ^{1}H NMR spectrum of **23m**.

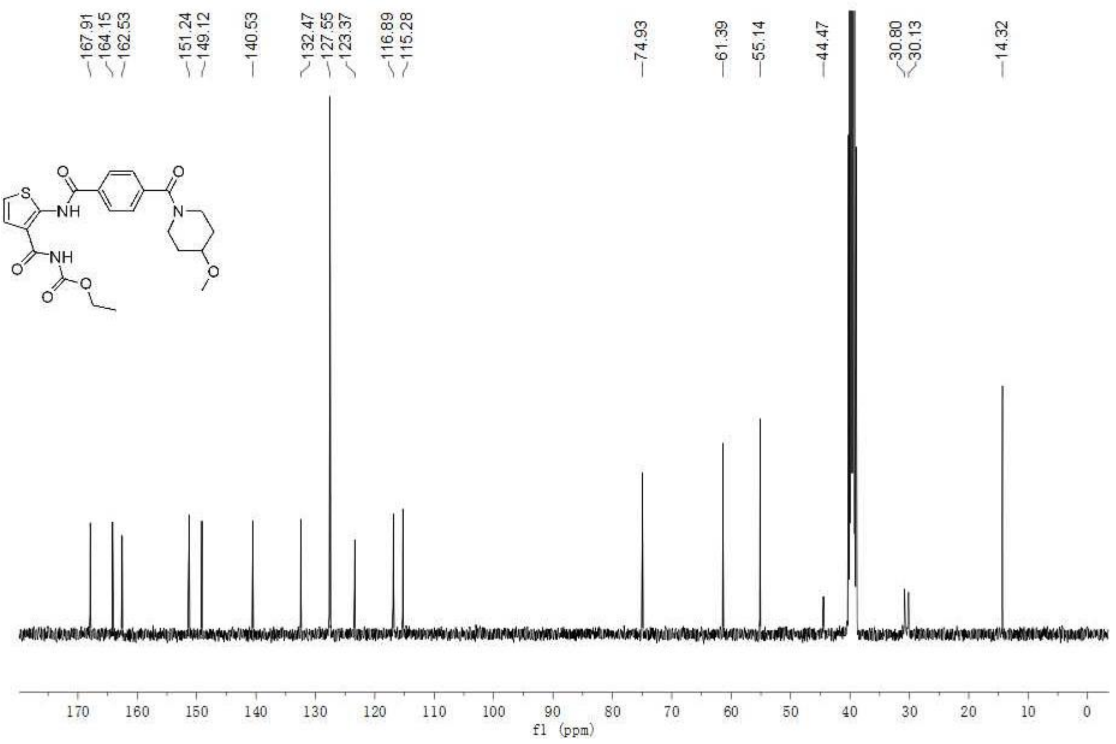

Figure S26. ^{13}C NMR spectrum of **23m**.

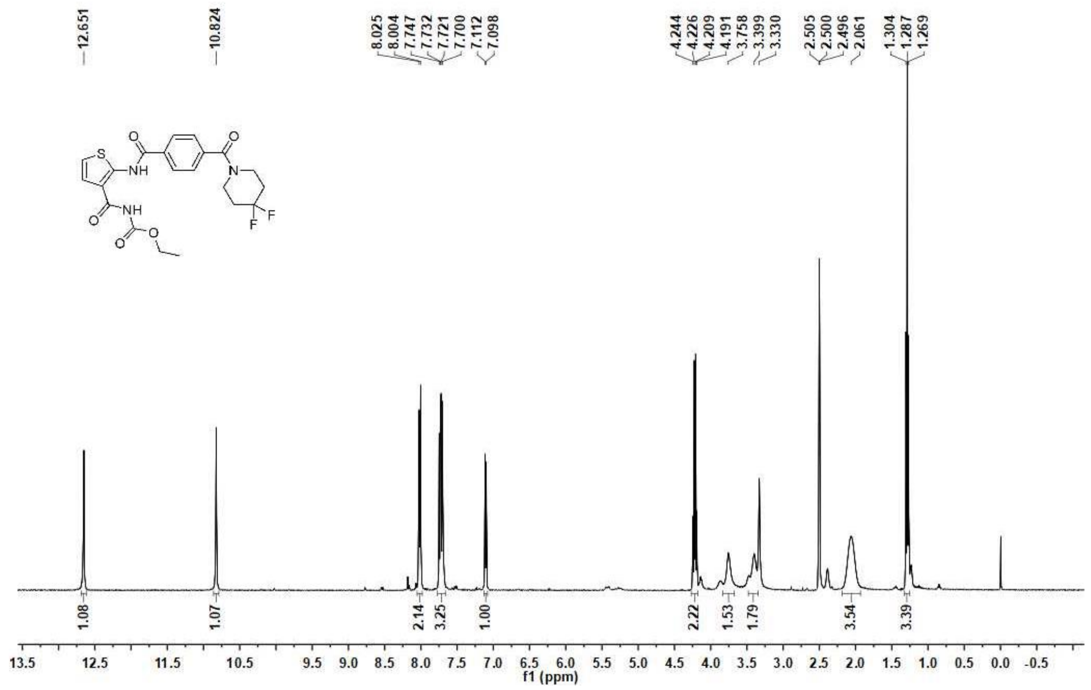

Figure S27. ^{1}H NMR spectrum of **23n**.

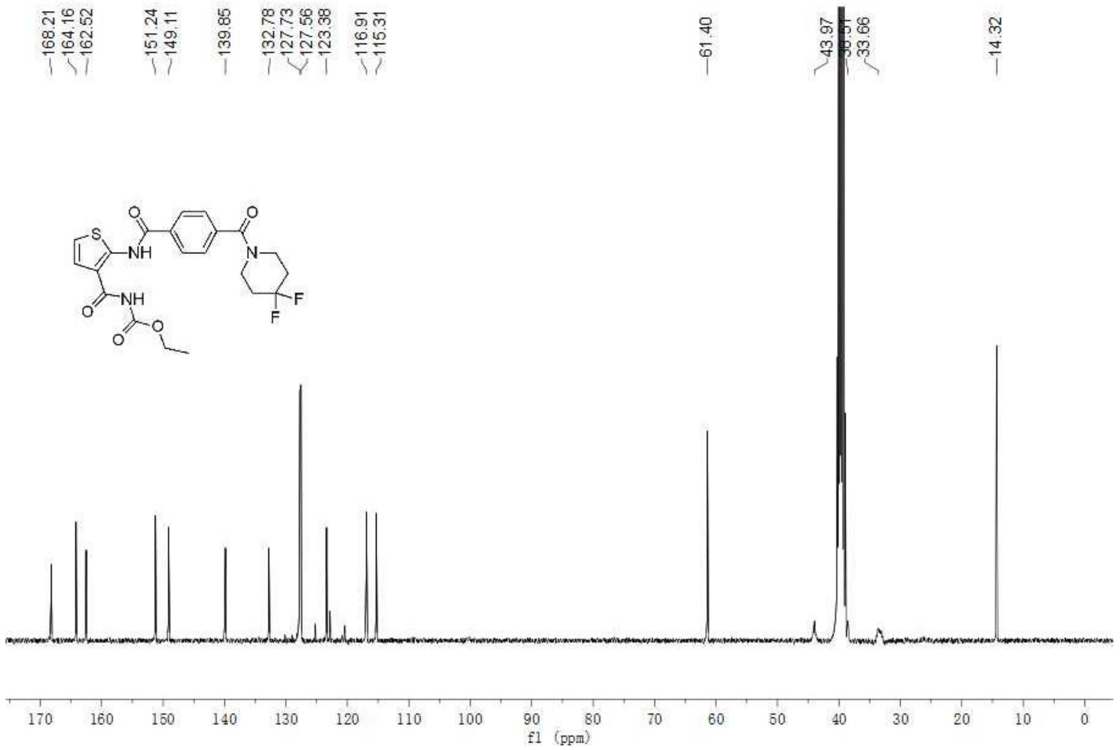

Figure S28. ^{13}C NMR spectrum of **23n**.

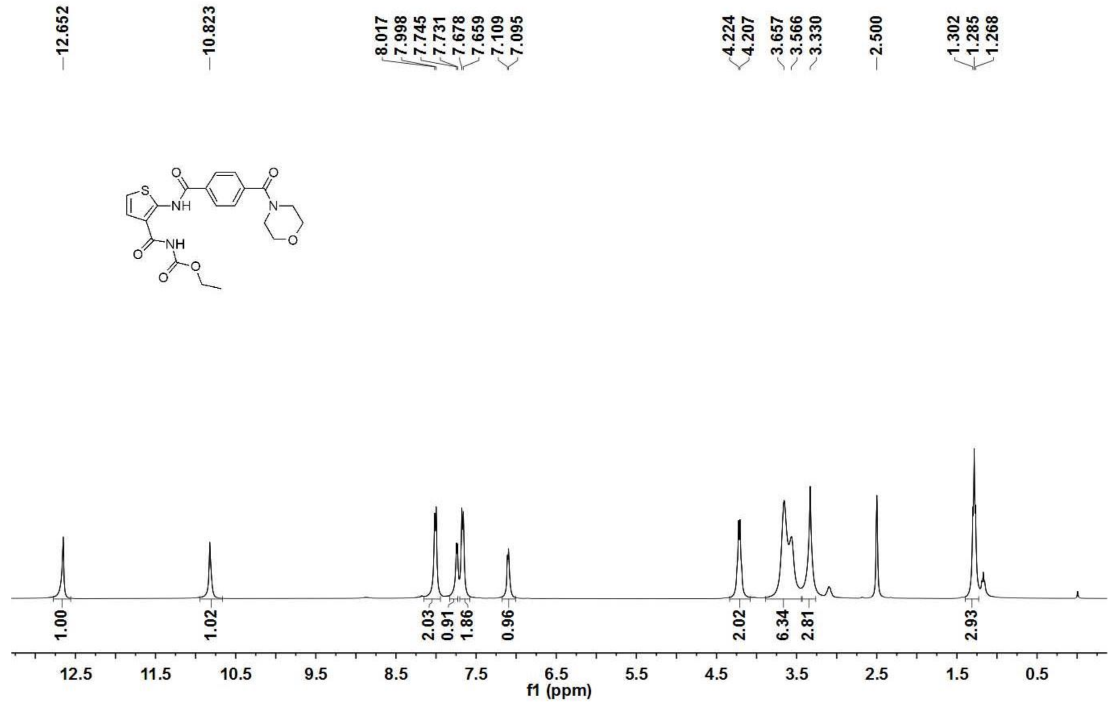

Figure S29. ^{1}H NMR spectrum of **23o**.

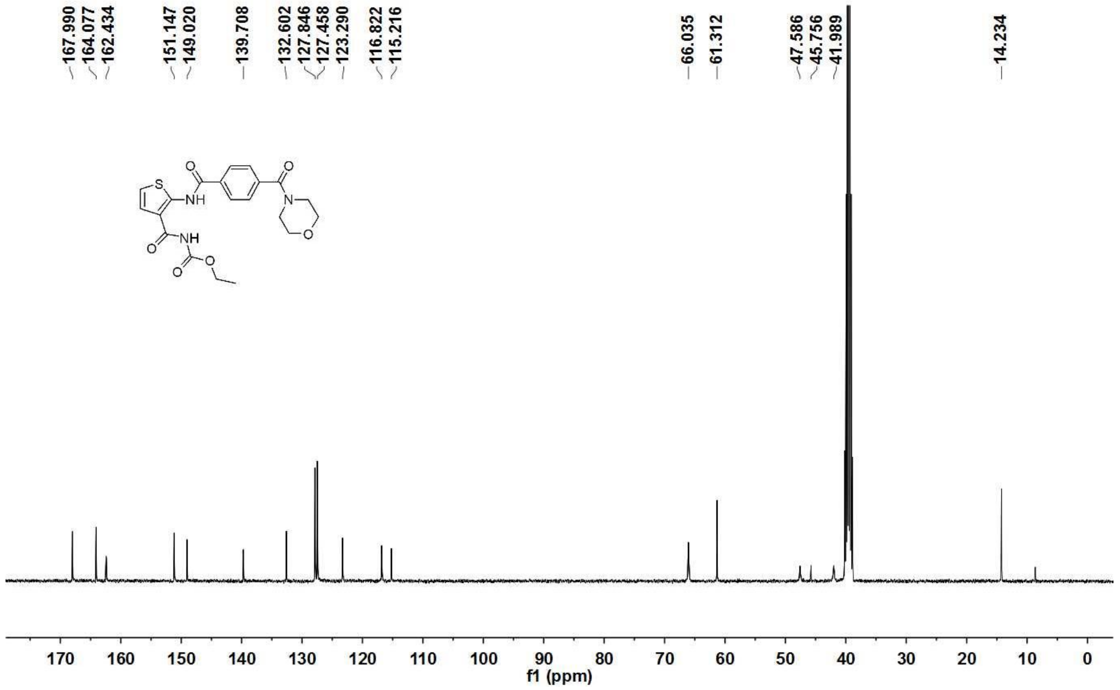

Figure S30. ^{13}C NMR spectrum of **23o**.

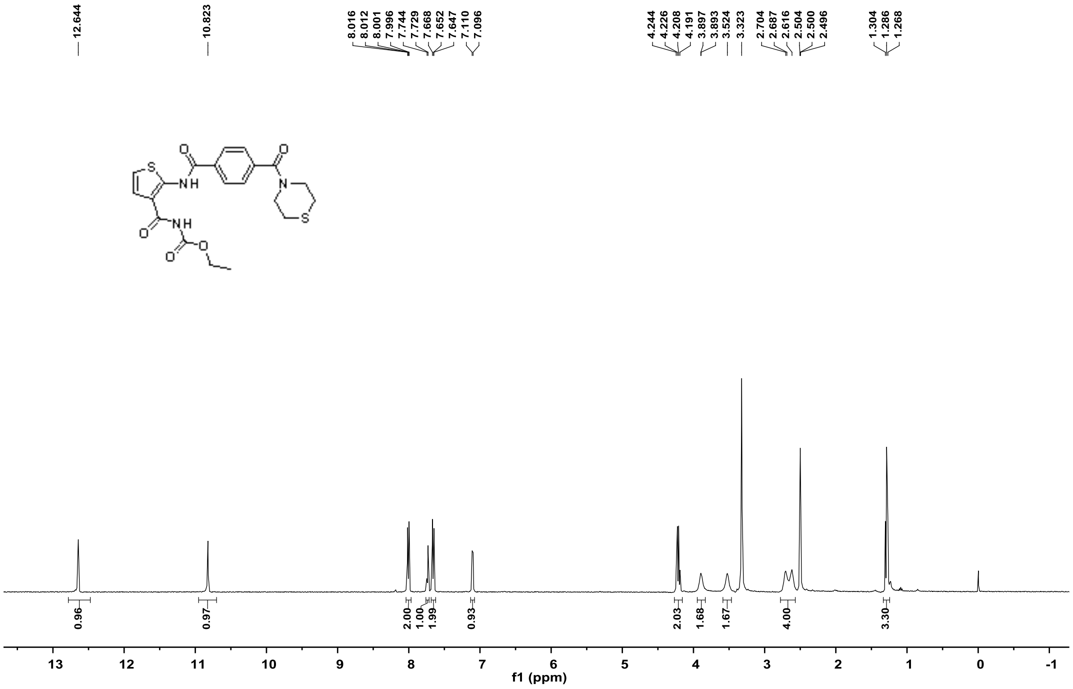

Figure S31. ^{1}H NMR spectrum of **23p**.

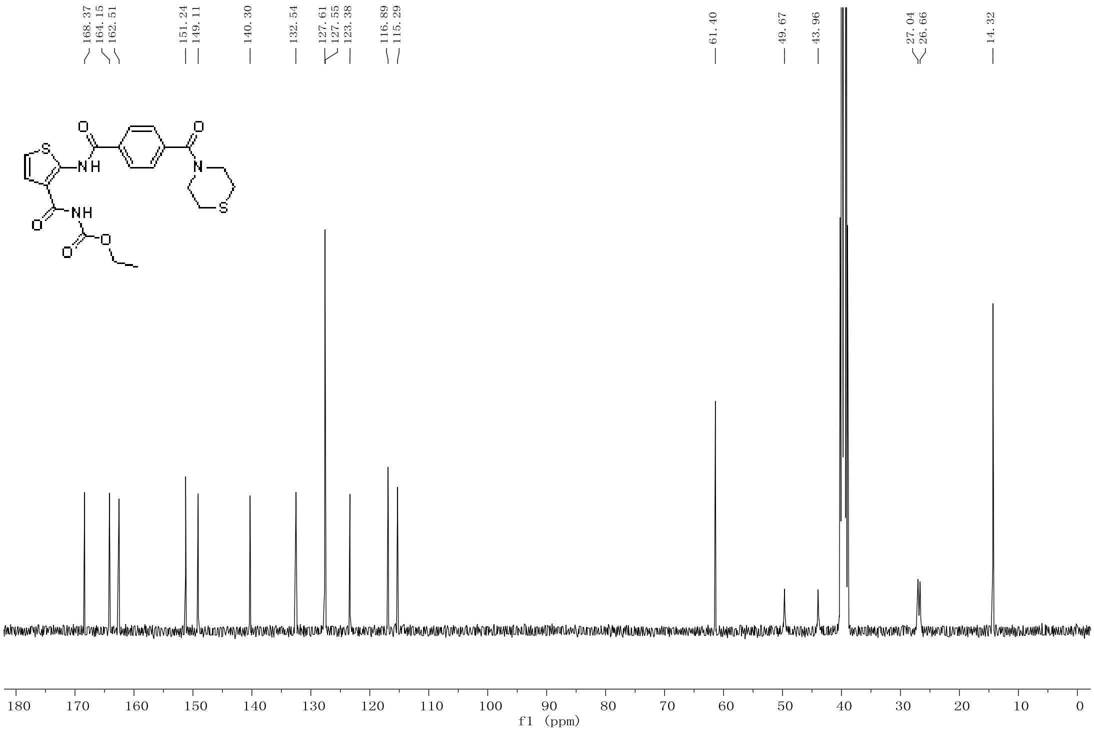

Figure S32. ^{13}C NMR spectrum of **23p**.

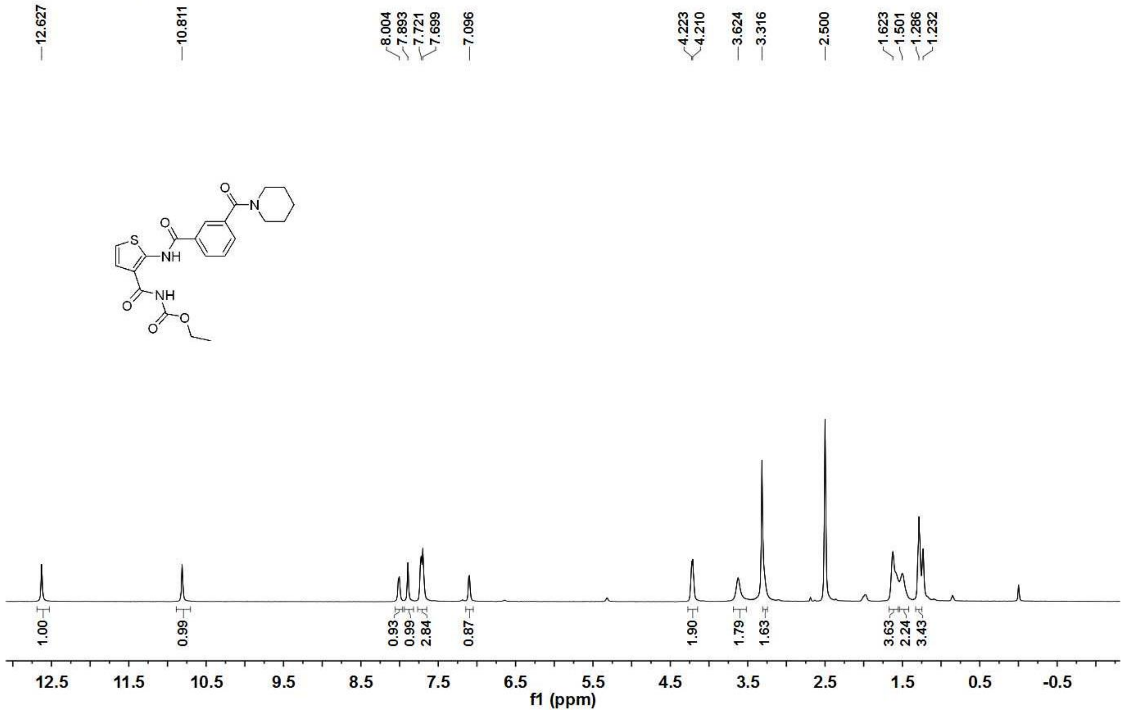

Figure S33. ^{1}H NMR spectrum of **24a**.

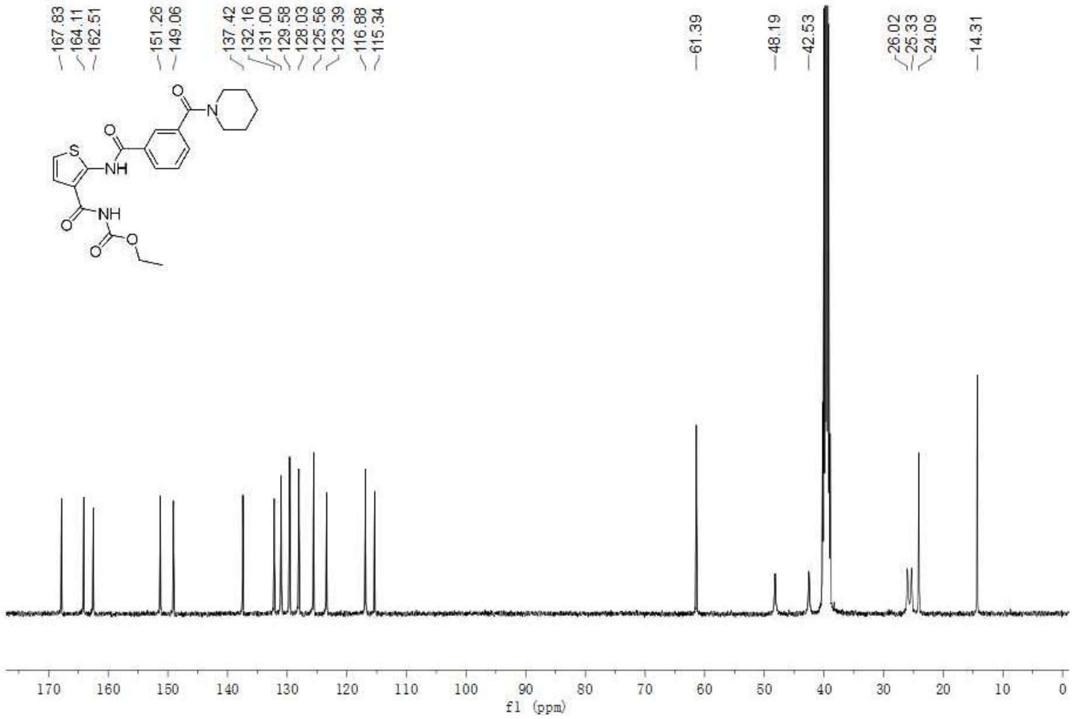

Figure S34. ^{13}C NMR spectrum of **24a**.

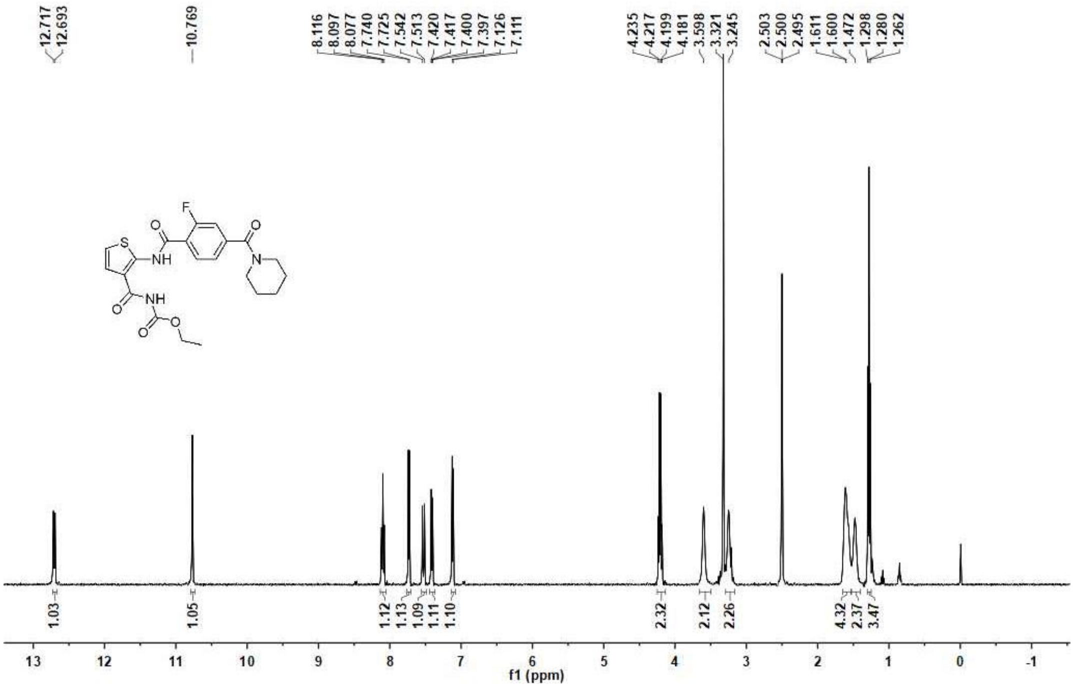

Figure S35. ^{1}H NMR spectrum of **24b**.

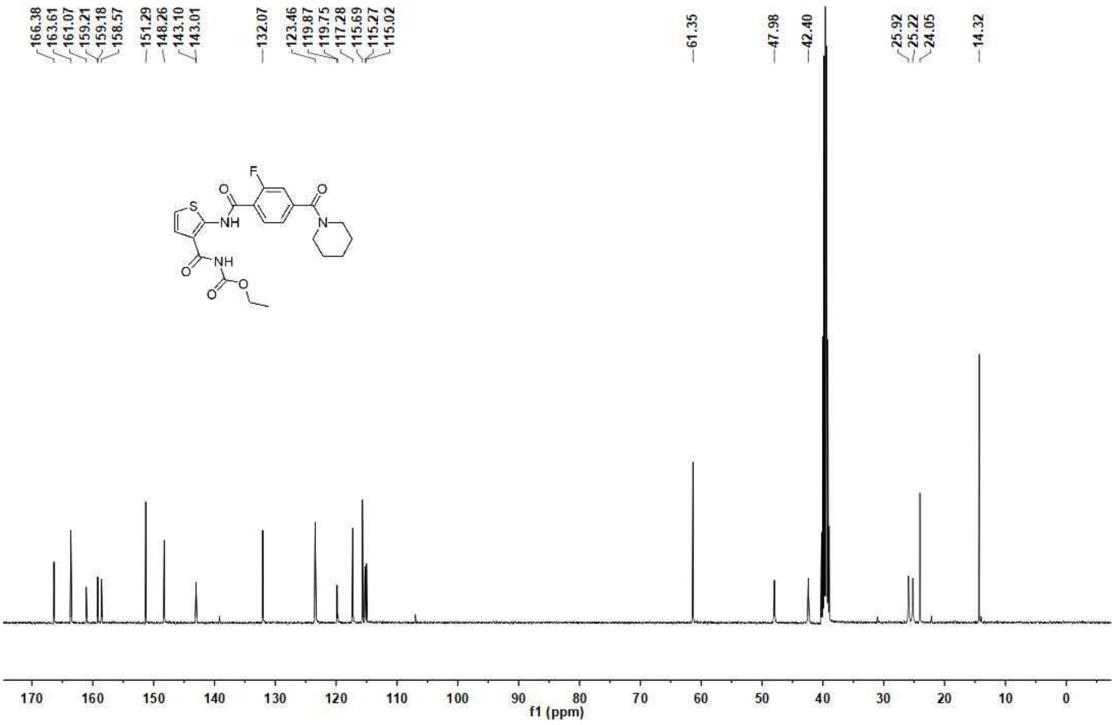

Figure S36. ^{13}C NMR spectrum of **24b**.

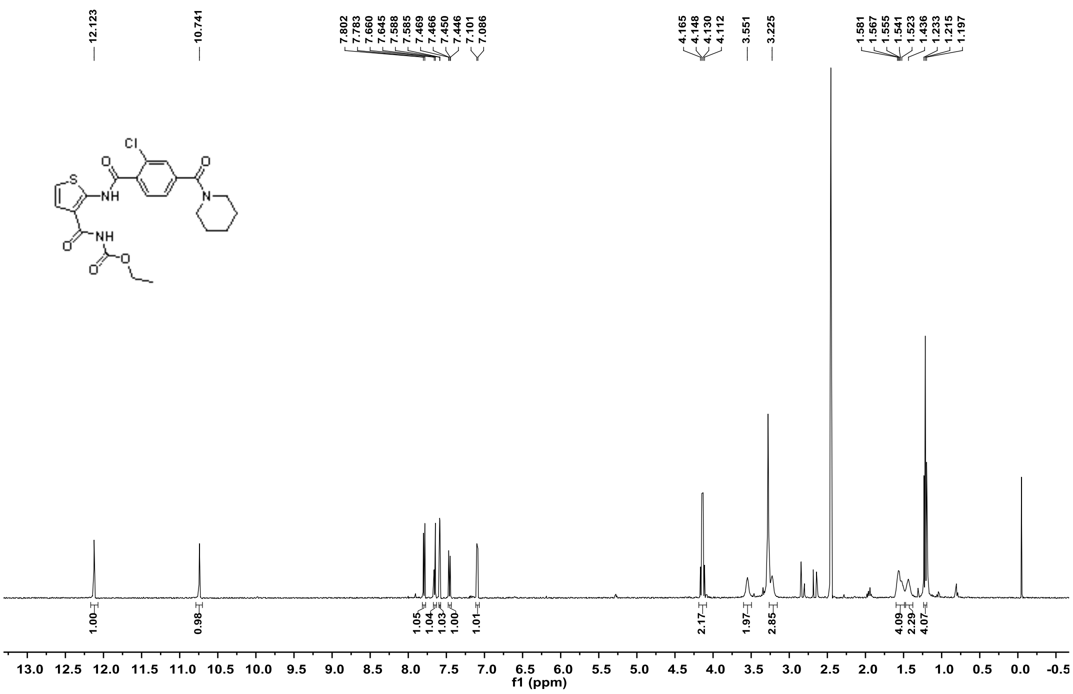

Figure S37. ^{1}H NMR spectrum of **24c**.

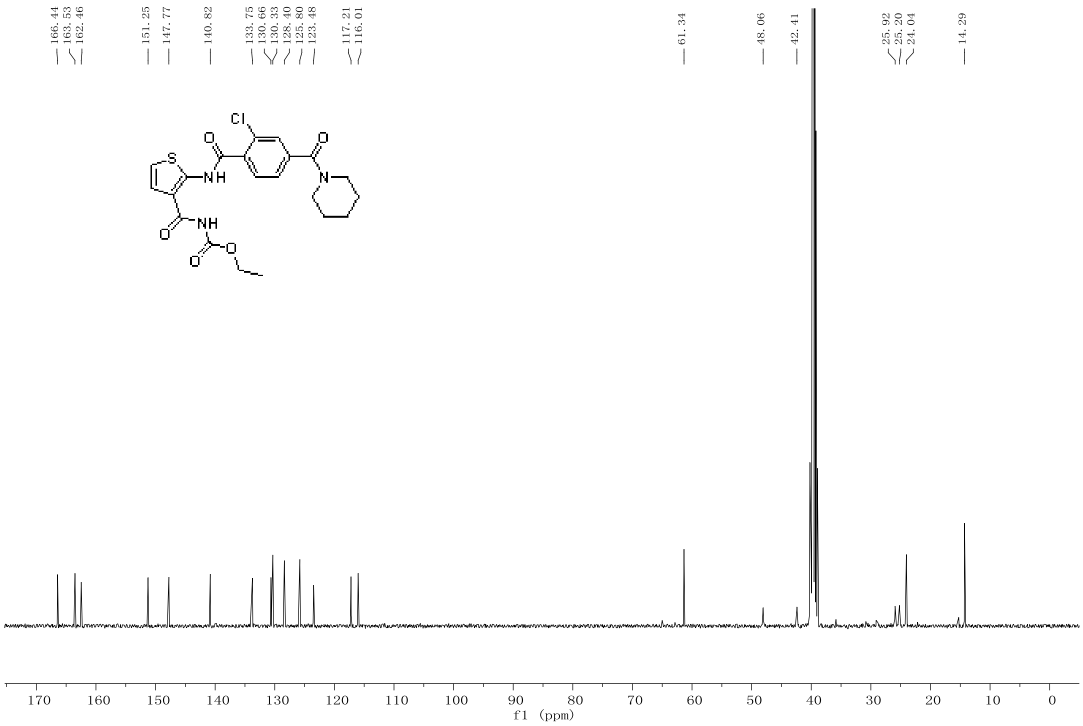

Figure S38. ^{13}C NMR spectrum of **24c**.

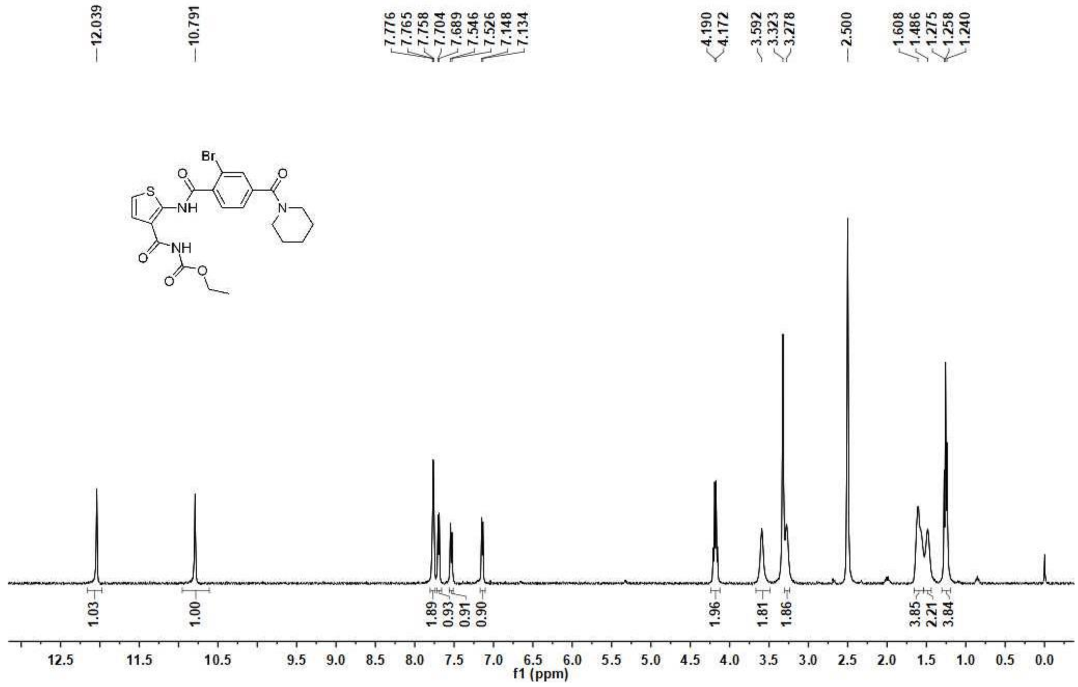

Figure S39. ^{1}H NMR spectrum of **24d**.

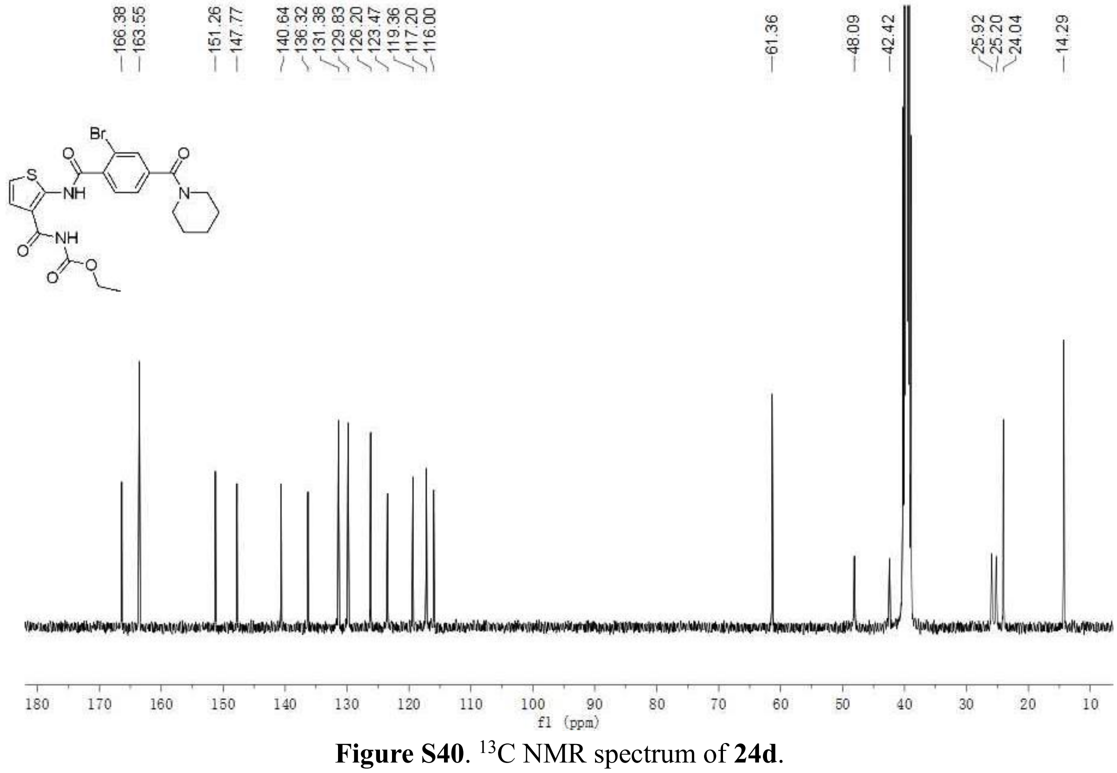

Figure S40. ^{13}C NMR spectrum of **24d**.

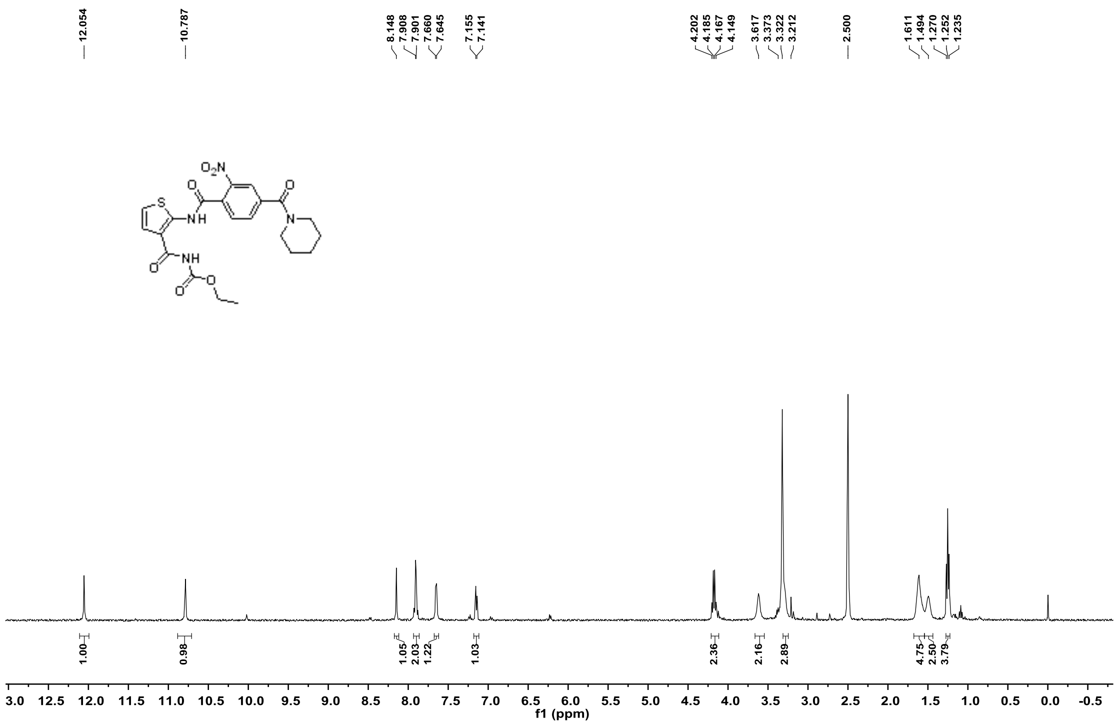

Figure S41. ^{1}H NMR spectrum of **24e**.

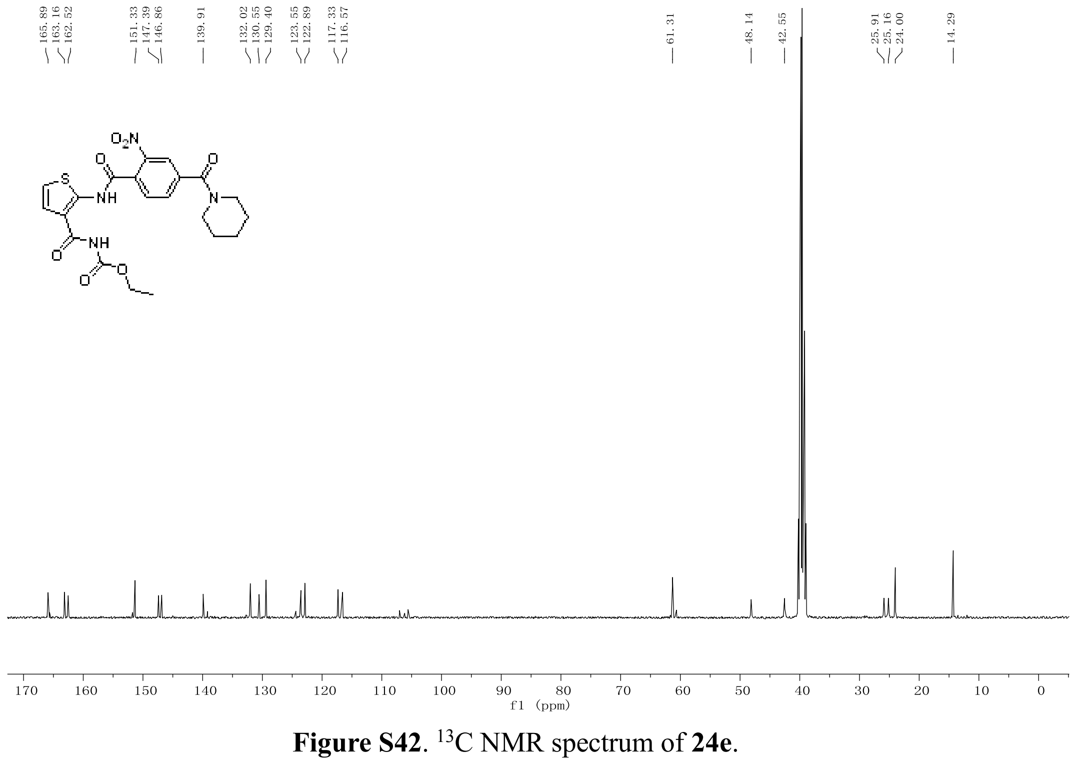

Figure S42. ^{13}C NMR spectrum of **24e**.

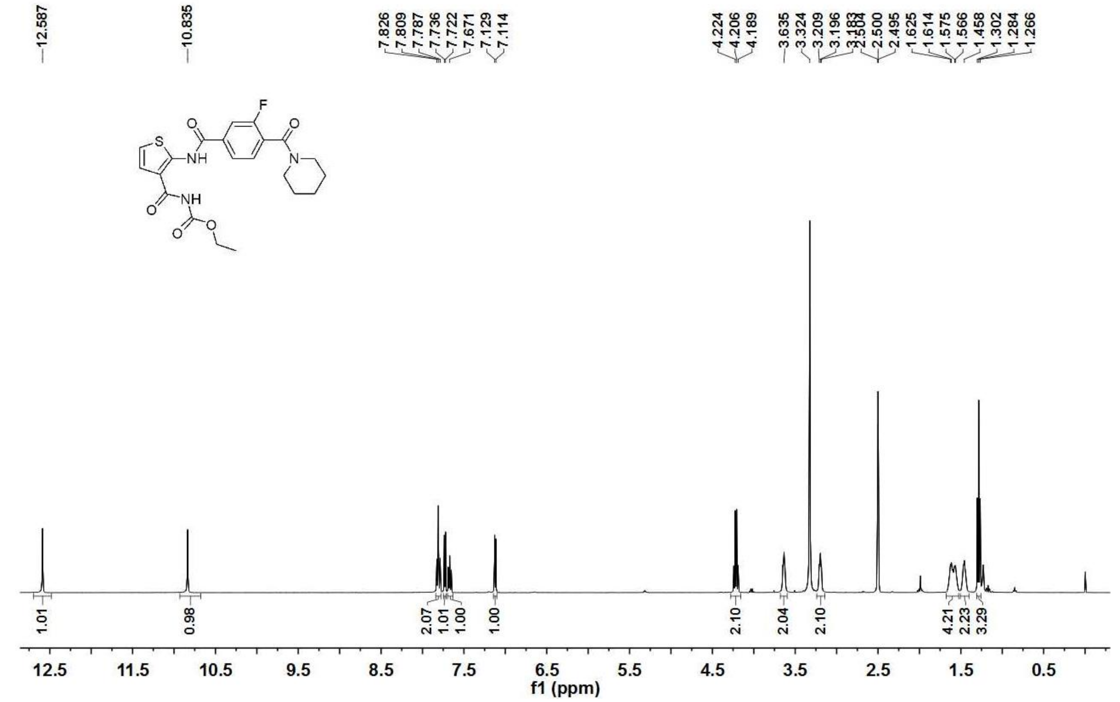

Figure S43. ^{1}H NMR spectrum of **24f**.

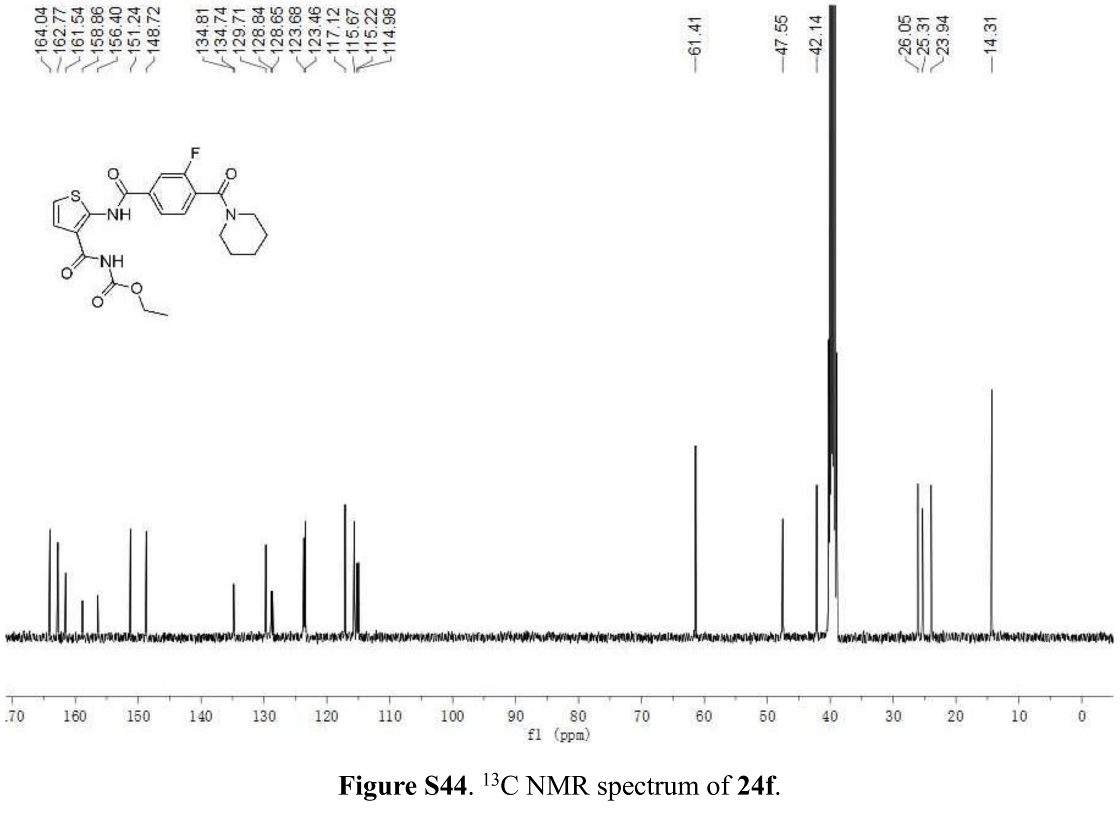

Figure S44. ^{13}C NMR spectrum of **24f**.

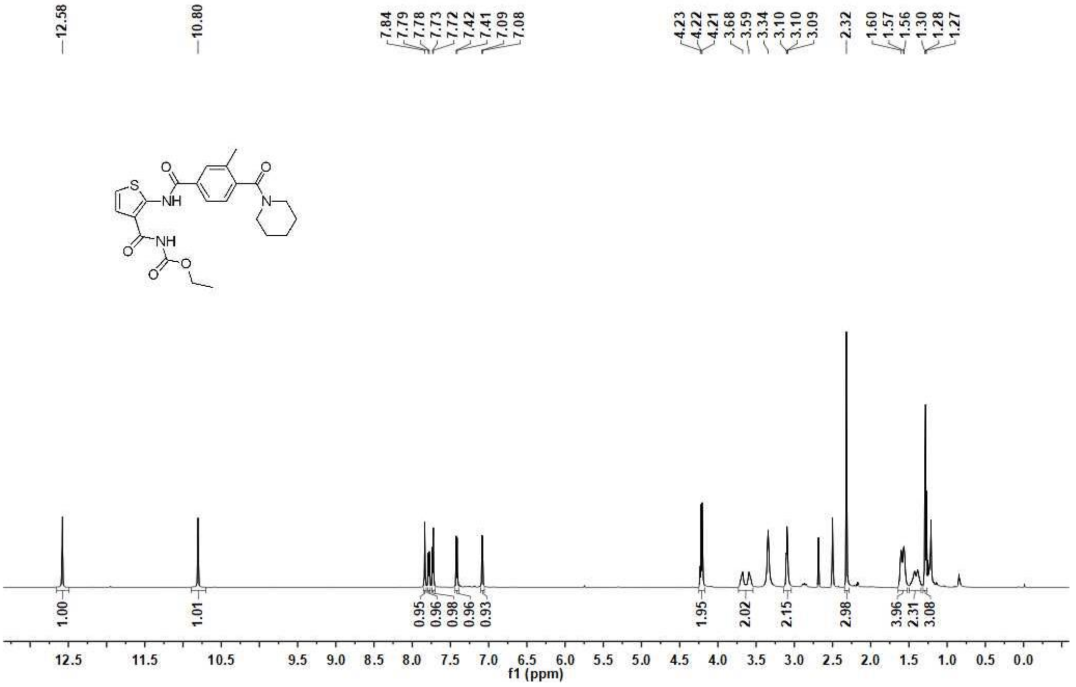

Figure S45. ^{1}H NMR spectrum of **24g**.

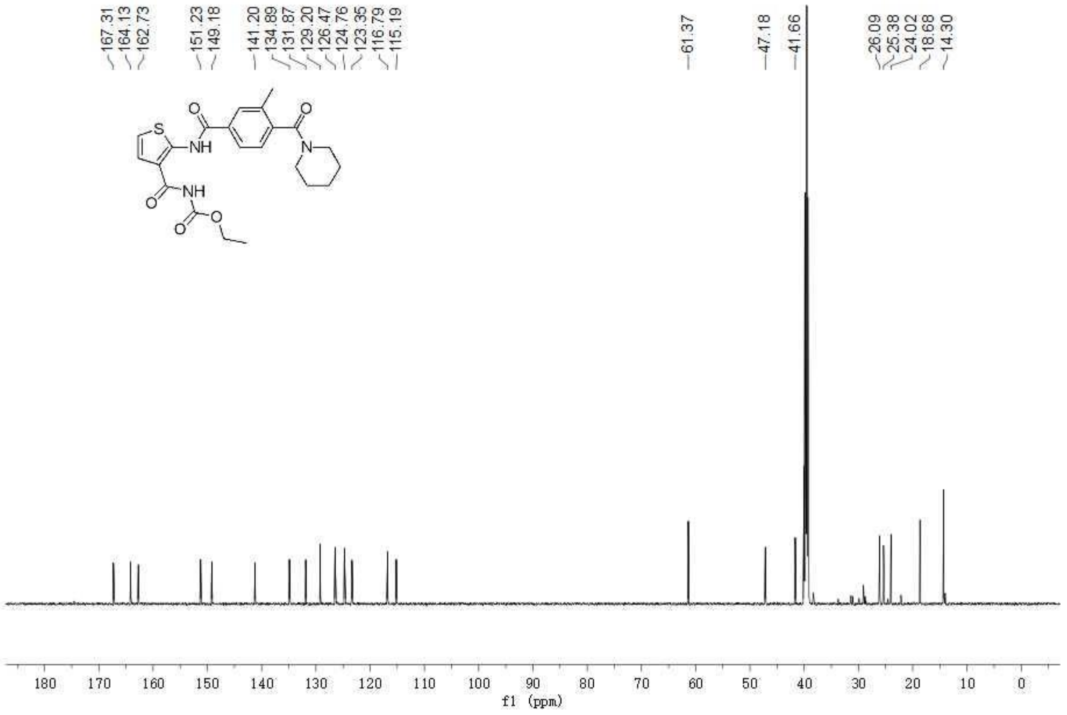

Figure S46. ^{13}C NMR spectrum of **24g**.

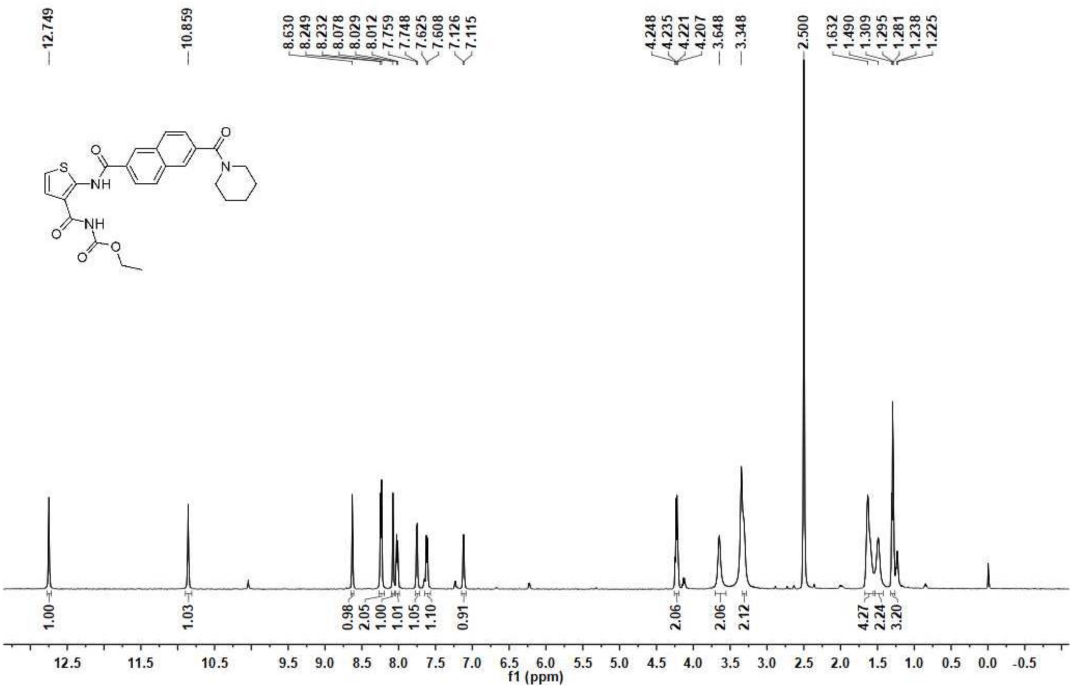

Figure S47. ^{1}H NMR spectrum of **24h**.

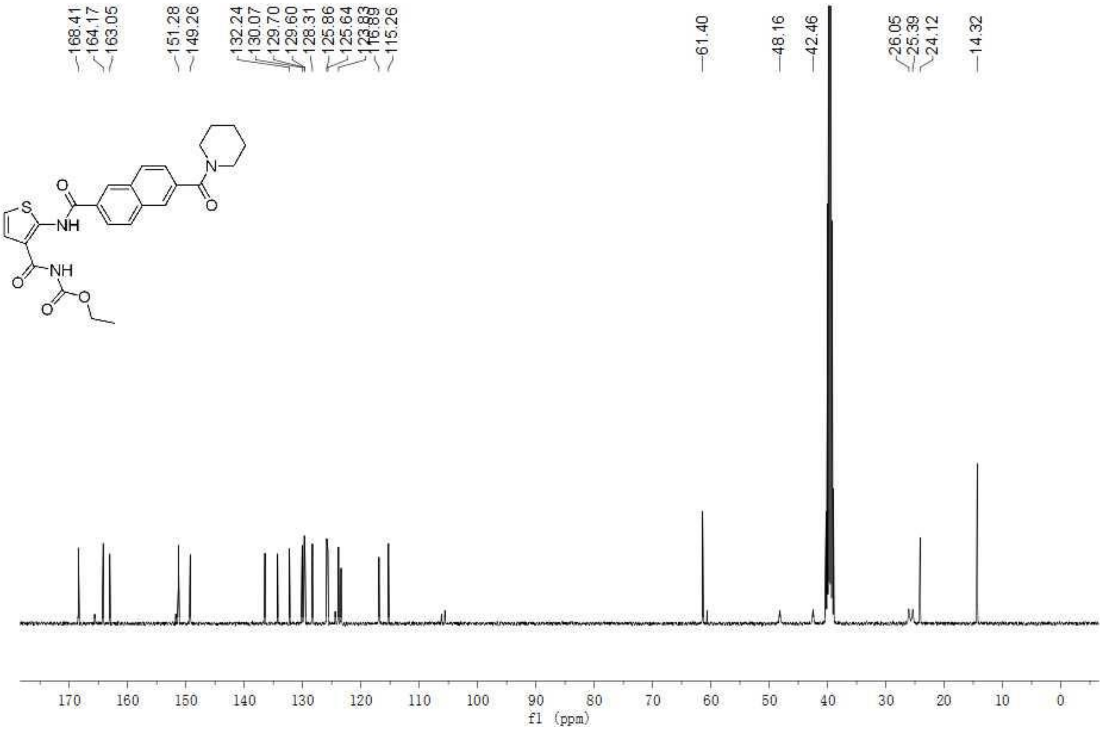

Figure S48. ^{13}C NMR spectrum of **24h**.

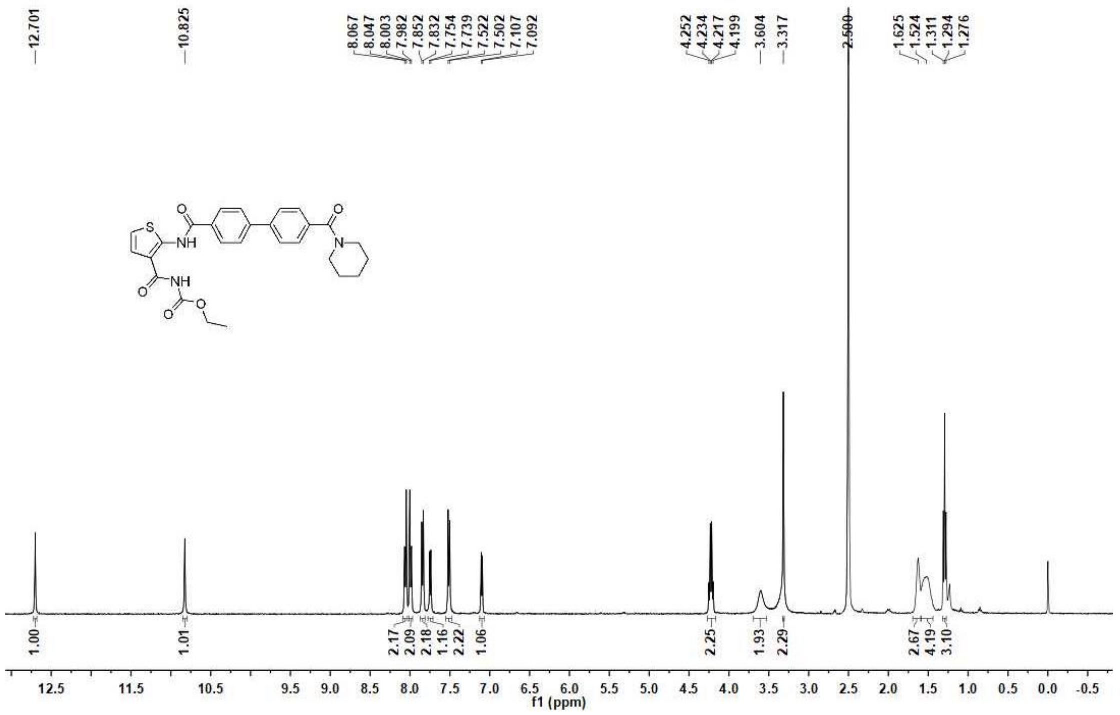

Figure S49. ^{1}H NMR spectrum of **24i**.

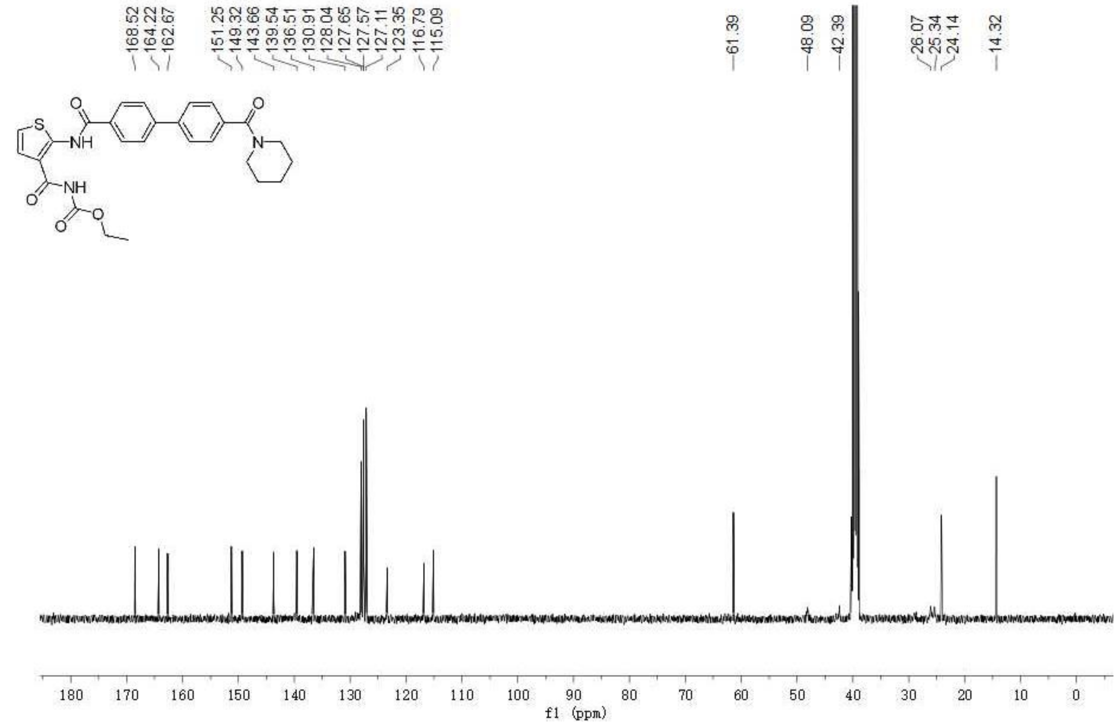

Figure S50. ^{13}C NMR spectrum of **24i**.

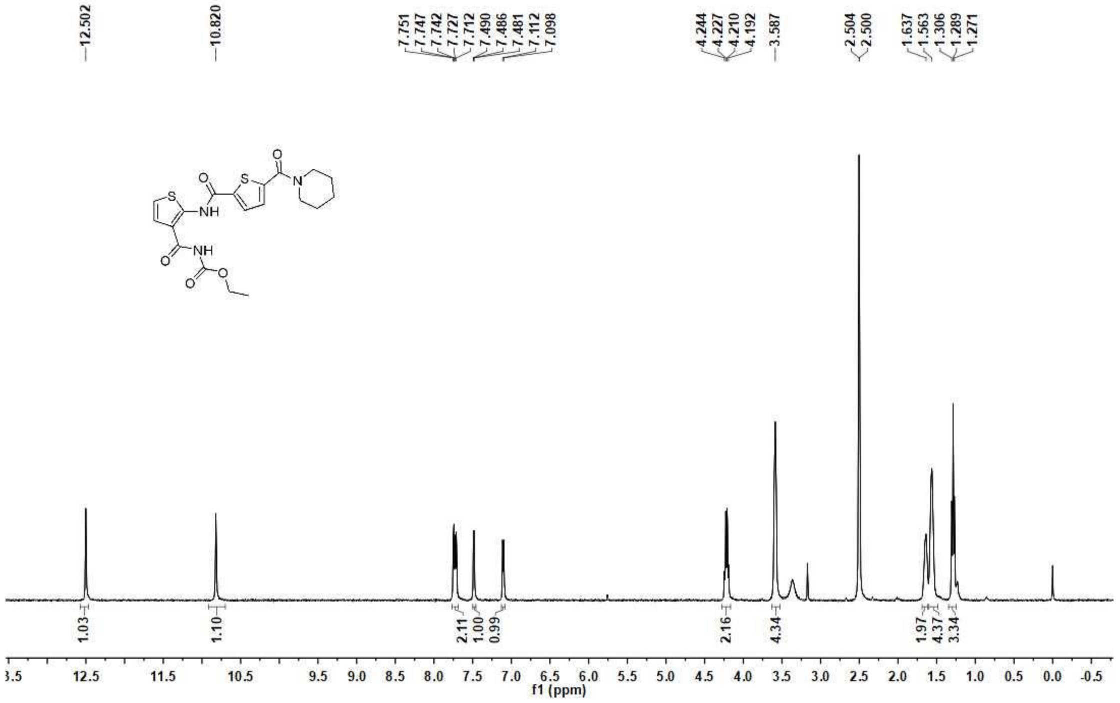

Figure S51. ^{1}H NMR spectrum of **24j**.

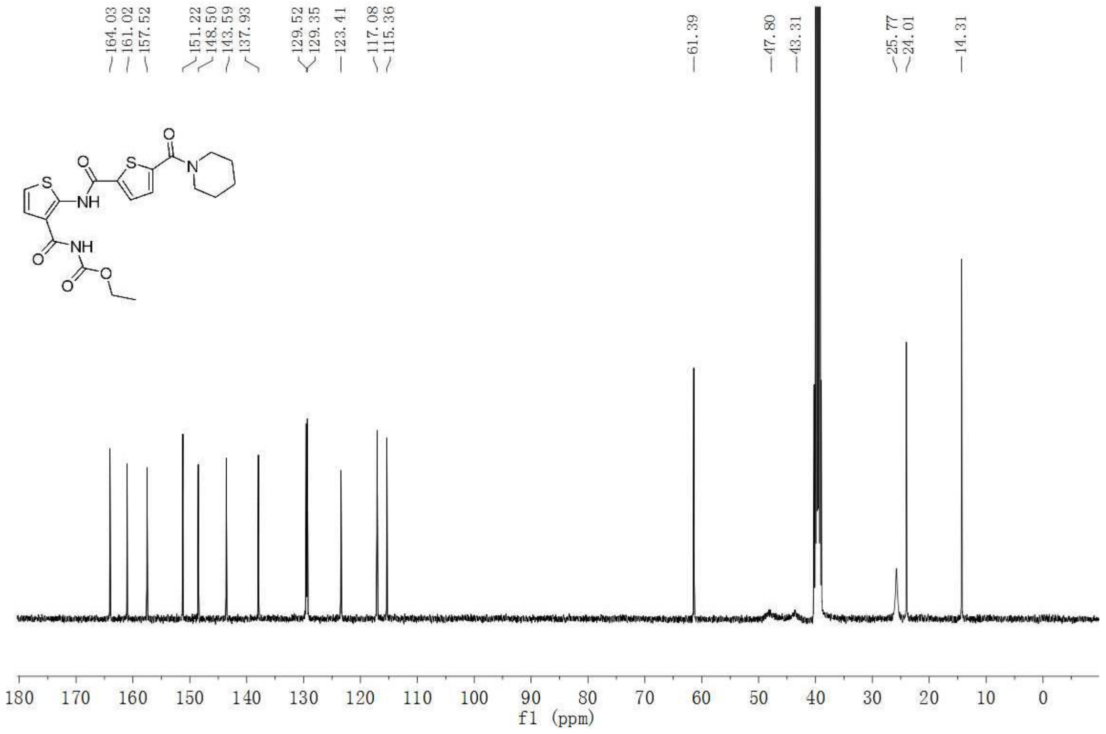

Figure S52. ^{13}C NMR spectrum of **24j**.

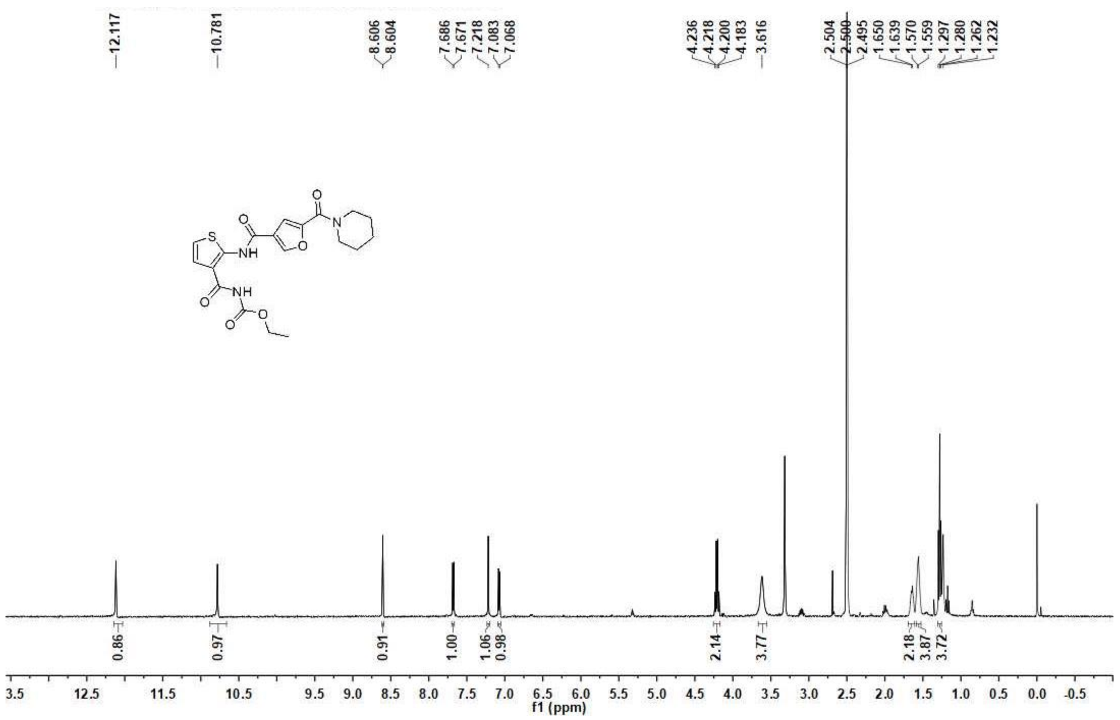

Figure S53. ^{1}H NMR spectrum of **24k**.

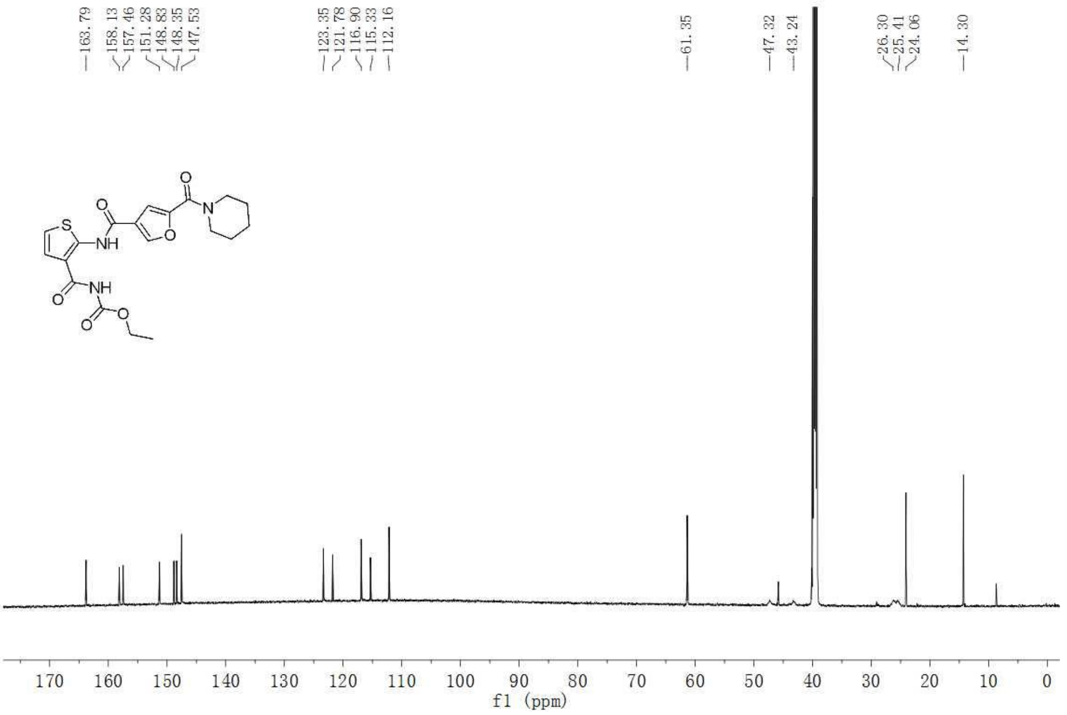

Figure S54. ^{13}C NMR spectrum of **24k**.

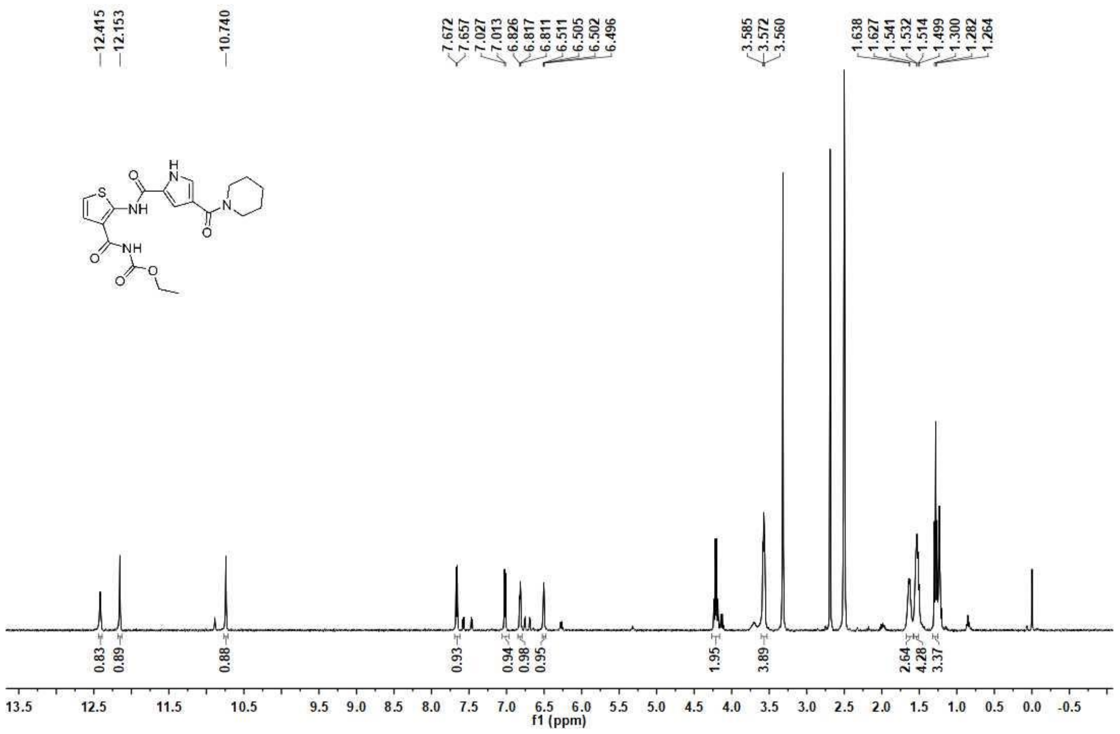

Figure S55. ^{1}H NMR spectrum of **24l**.

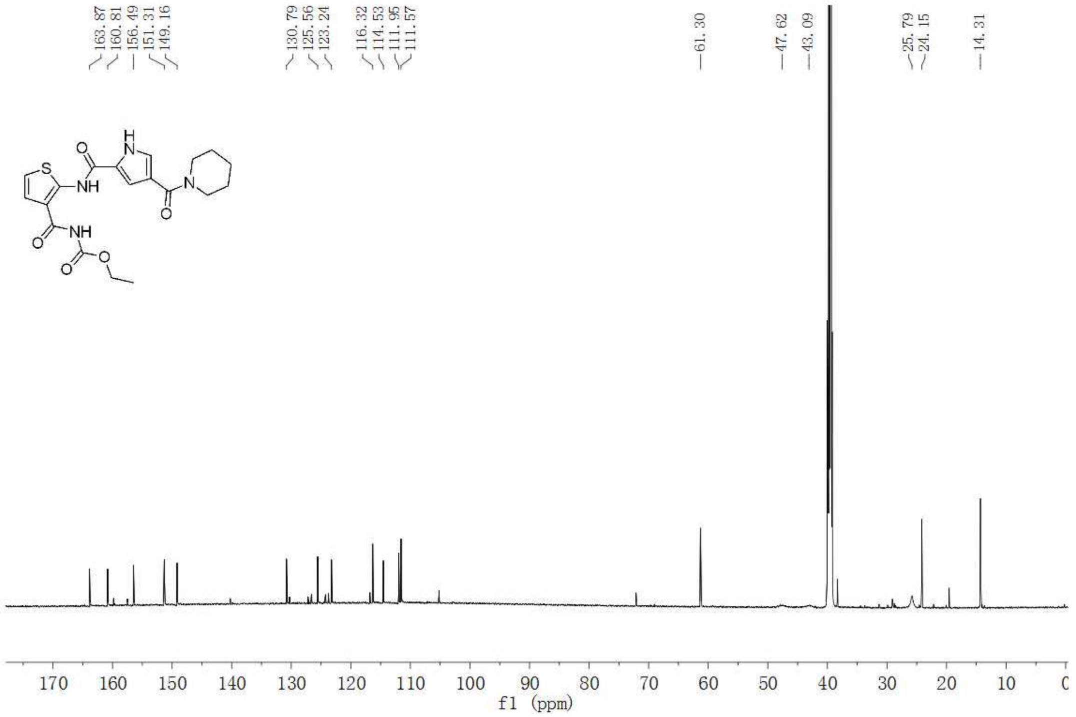

Figure S56. ^{13}C NMR spectrum of **24l**.

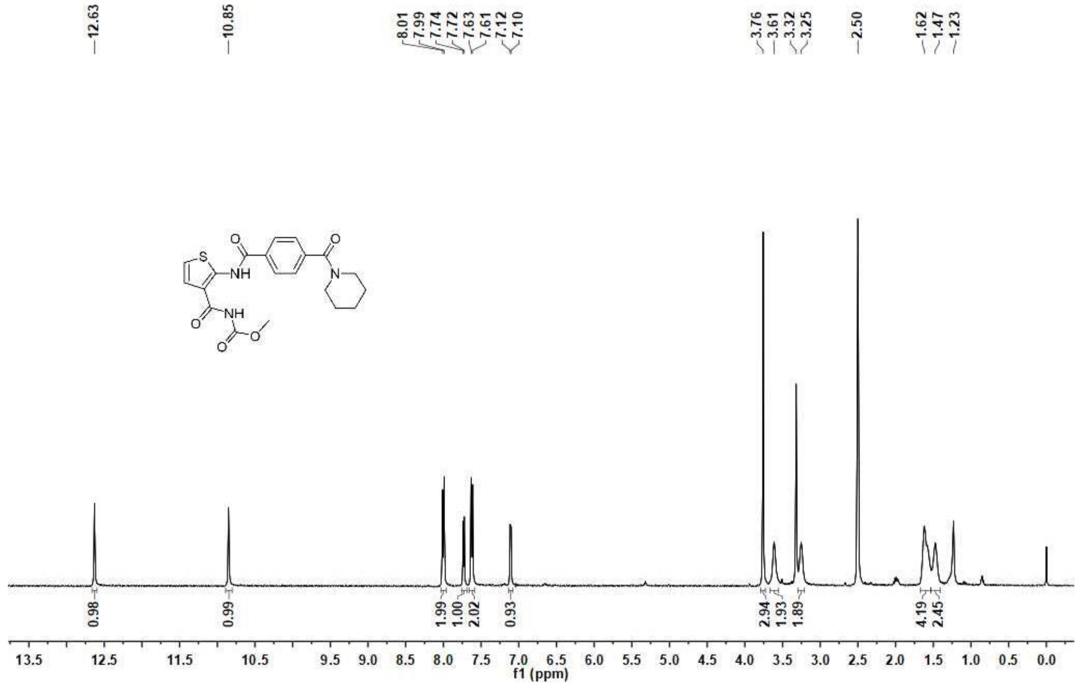

Figure S57. ^{1}H NMR spectrum of **25a**.

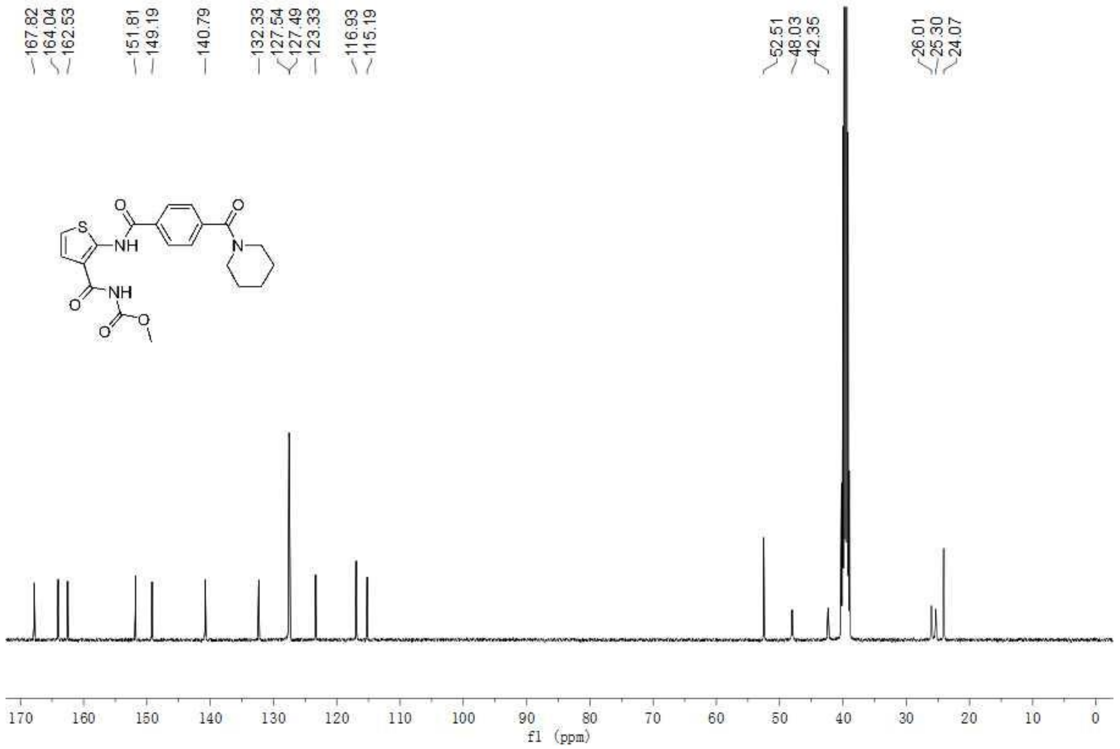

Figure S58. ^{13}C NMR spectrum of **25a**.

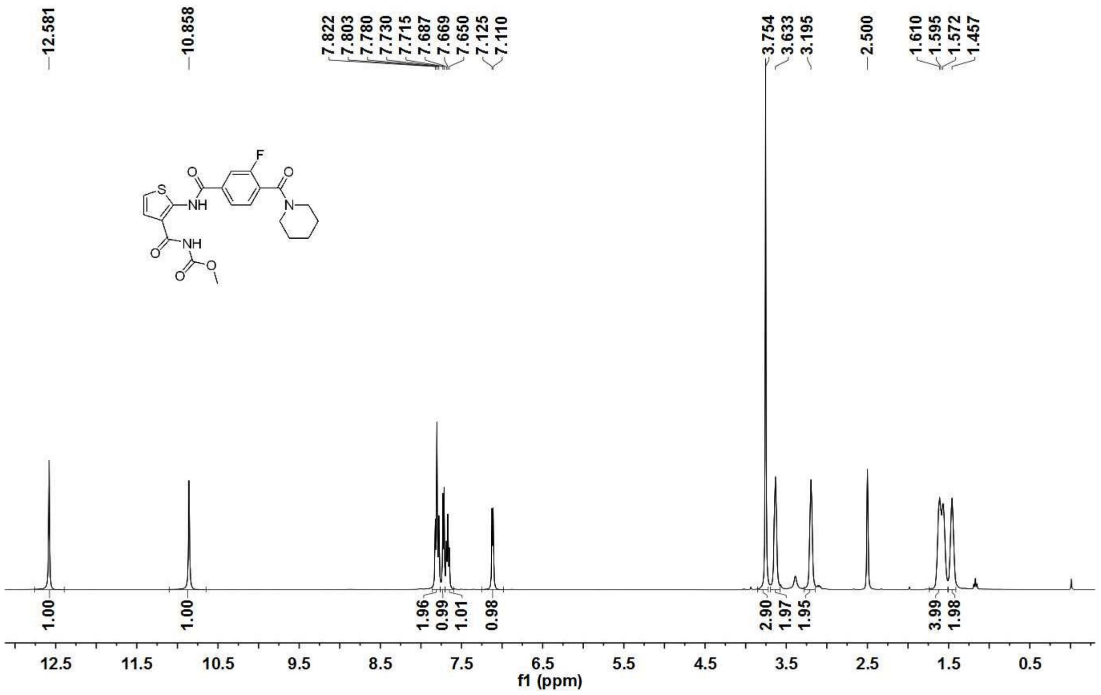

Figure S59. ^{1}H NMR spectrum of **25b**.

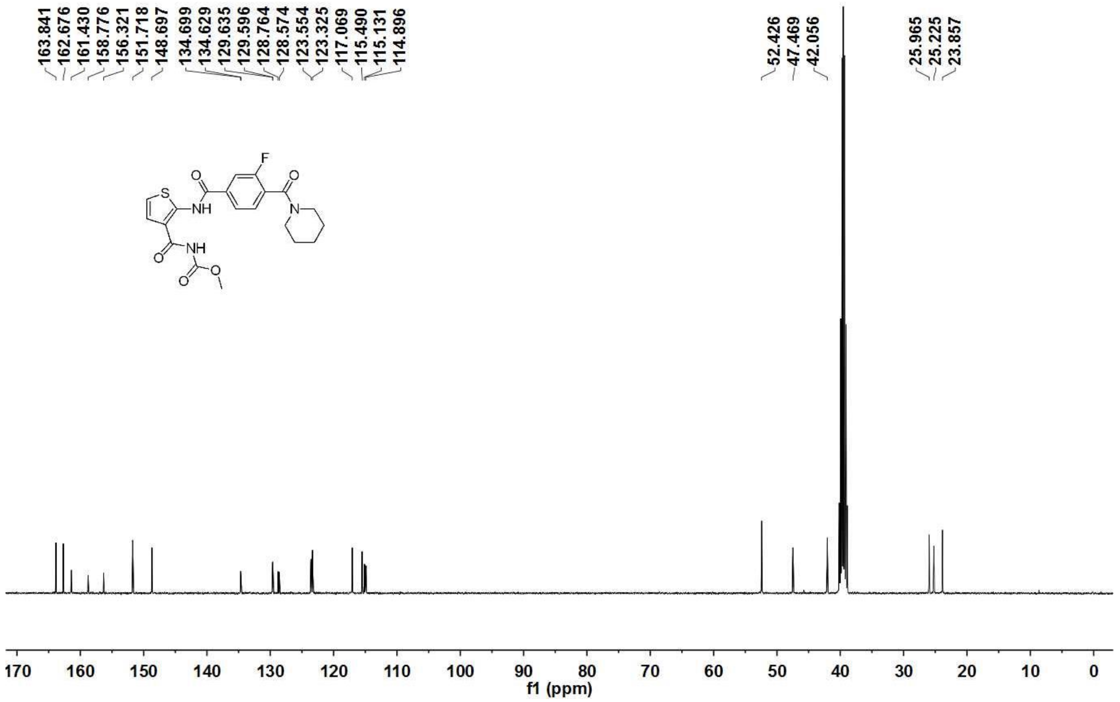

Figure S60. ^{13}C NMR spectrum of **25b**.

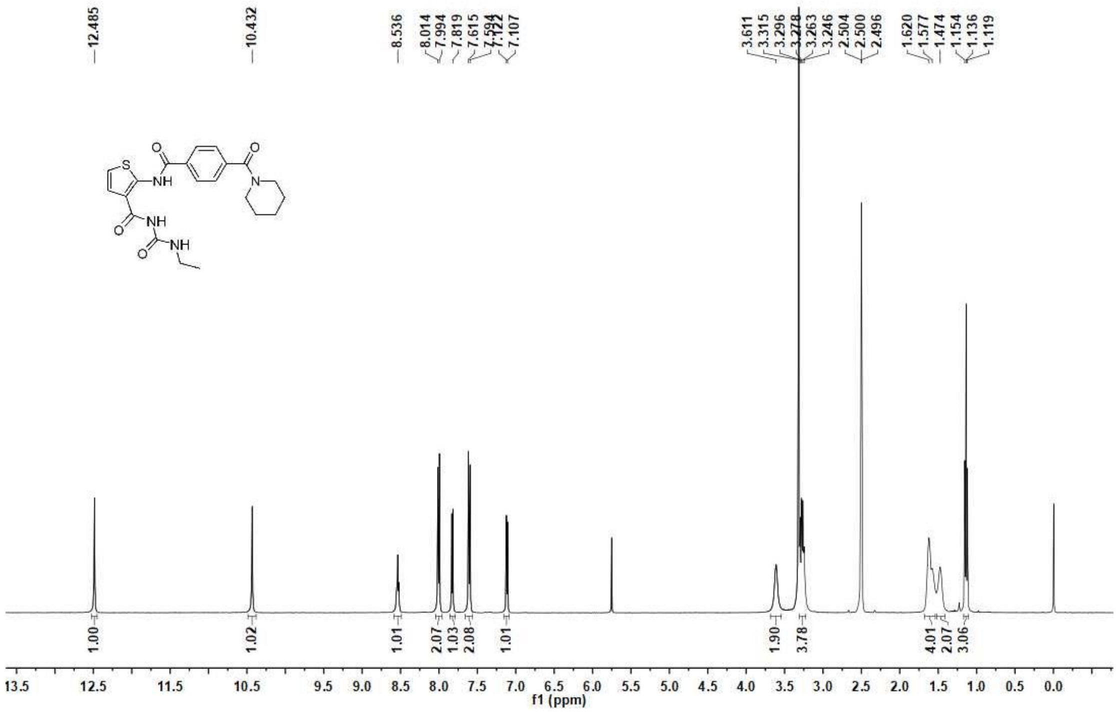

Figure S61. ^{1}H NMR spectrum of **25c**.

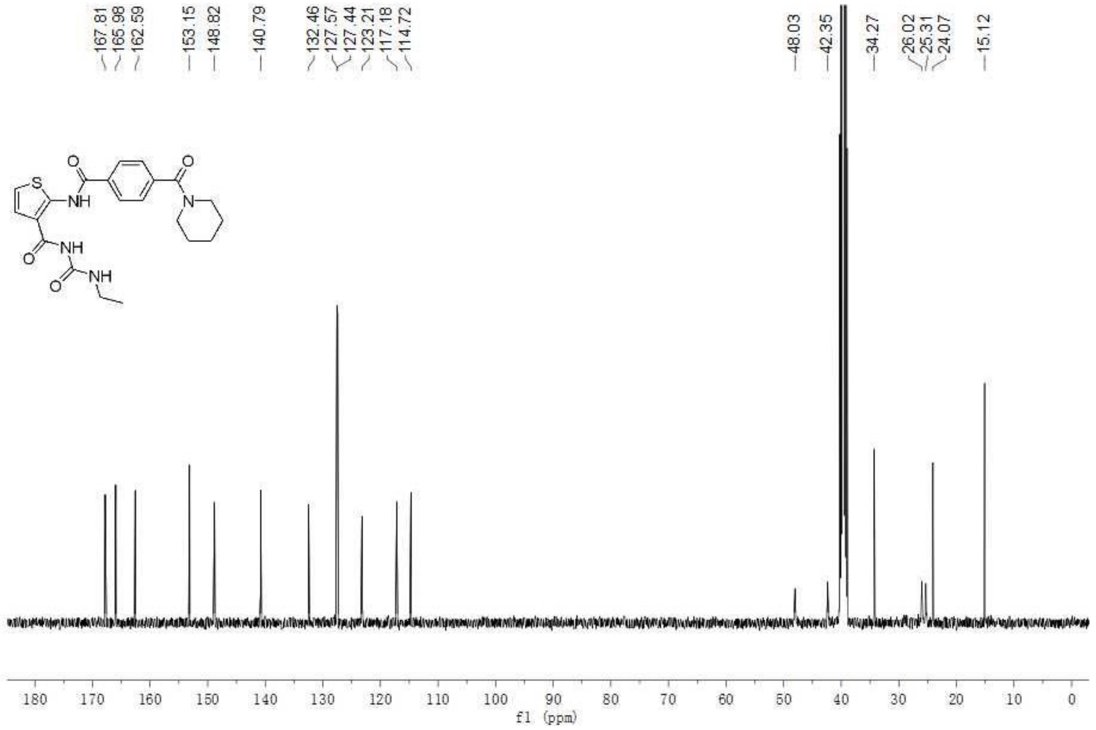

Figure S62. ^{13}C NMR spectrum of **25c**.

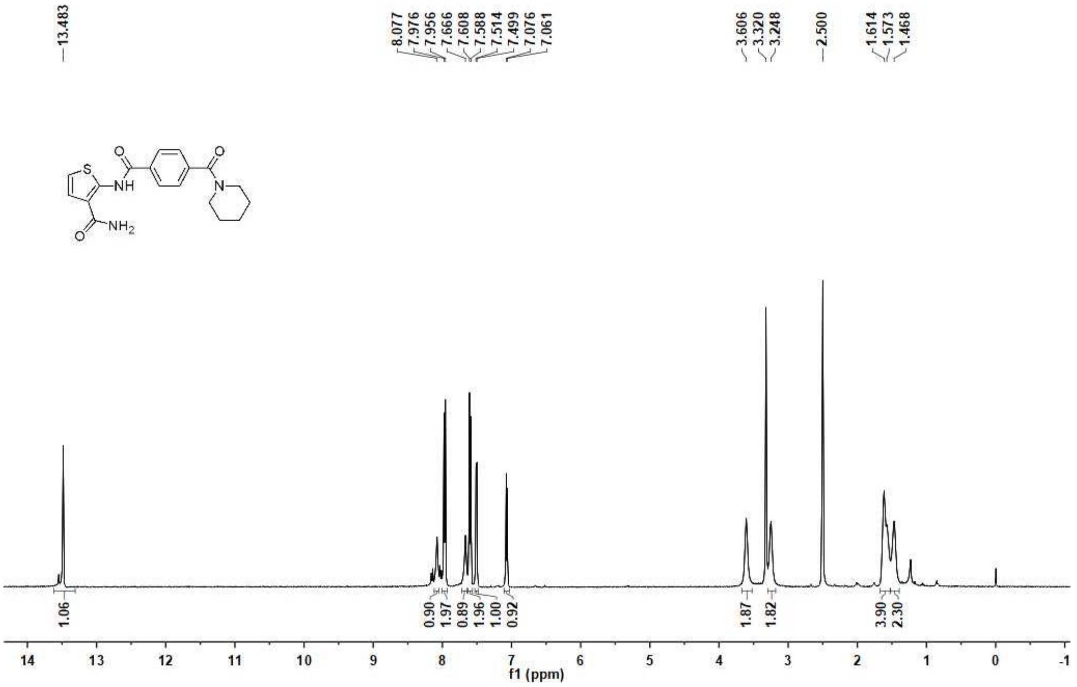

Figure S63. ^{1}H NMR spectrum of **25d**.

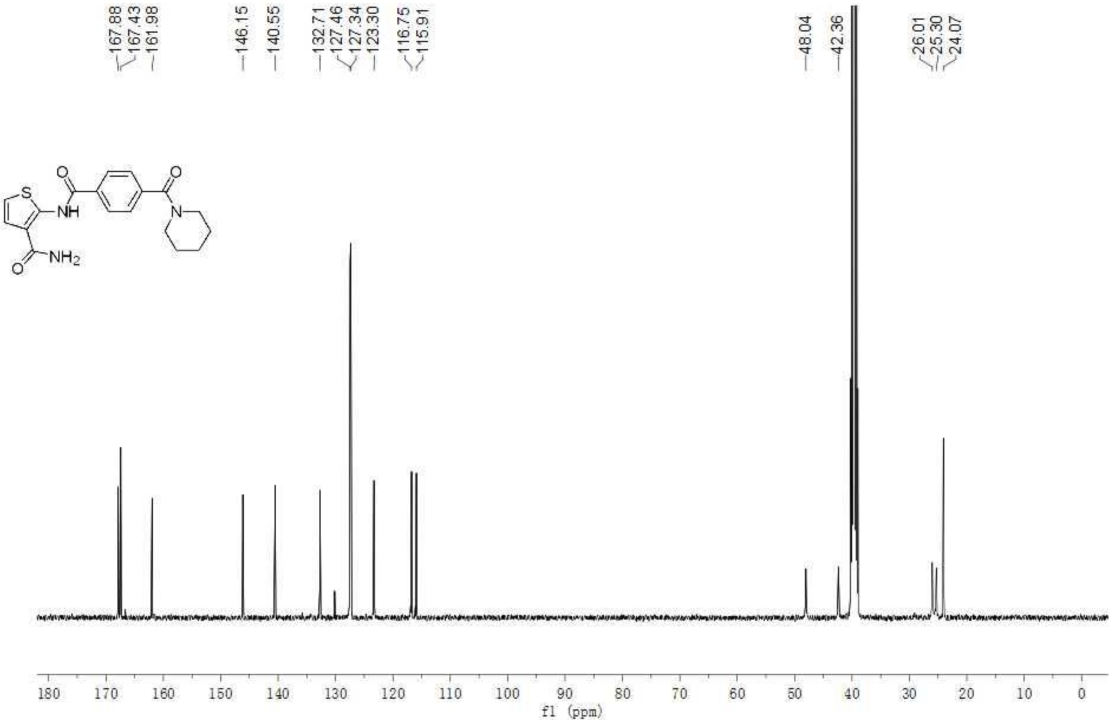

Figure S64. ^{13}C NMR spectrum of **25d**.

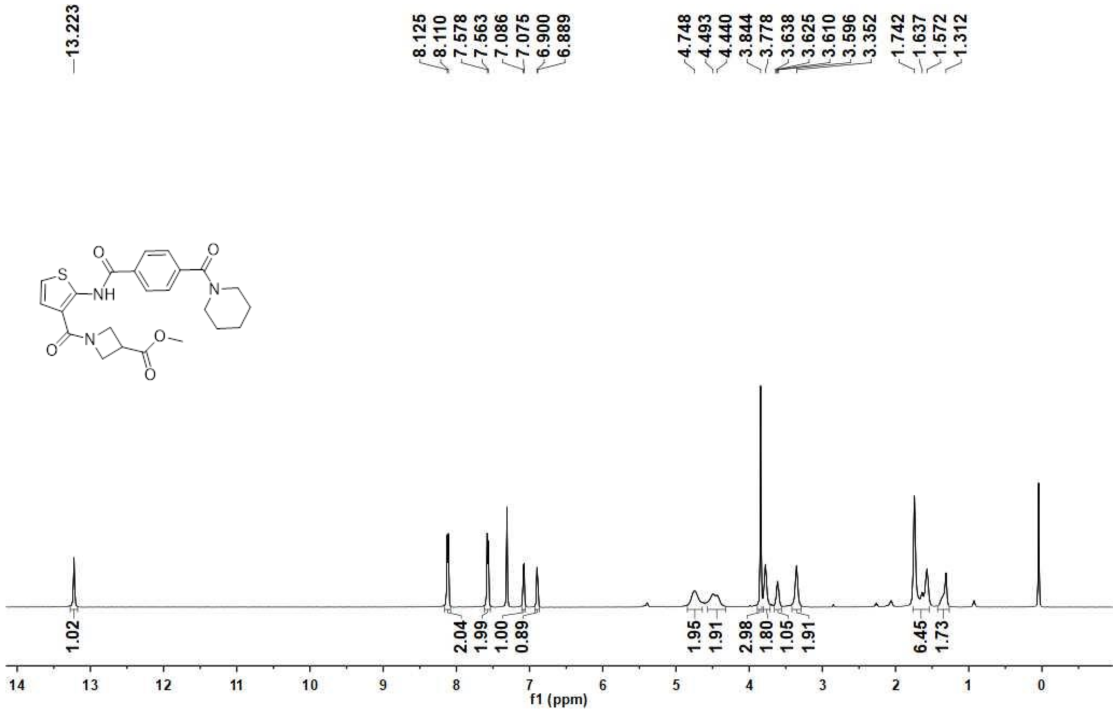

Figure S65. ^{1}H NMR spectrum of **25e**.

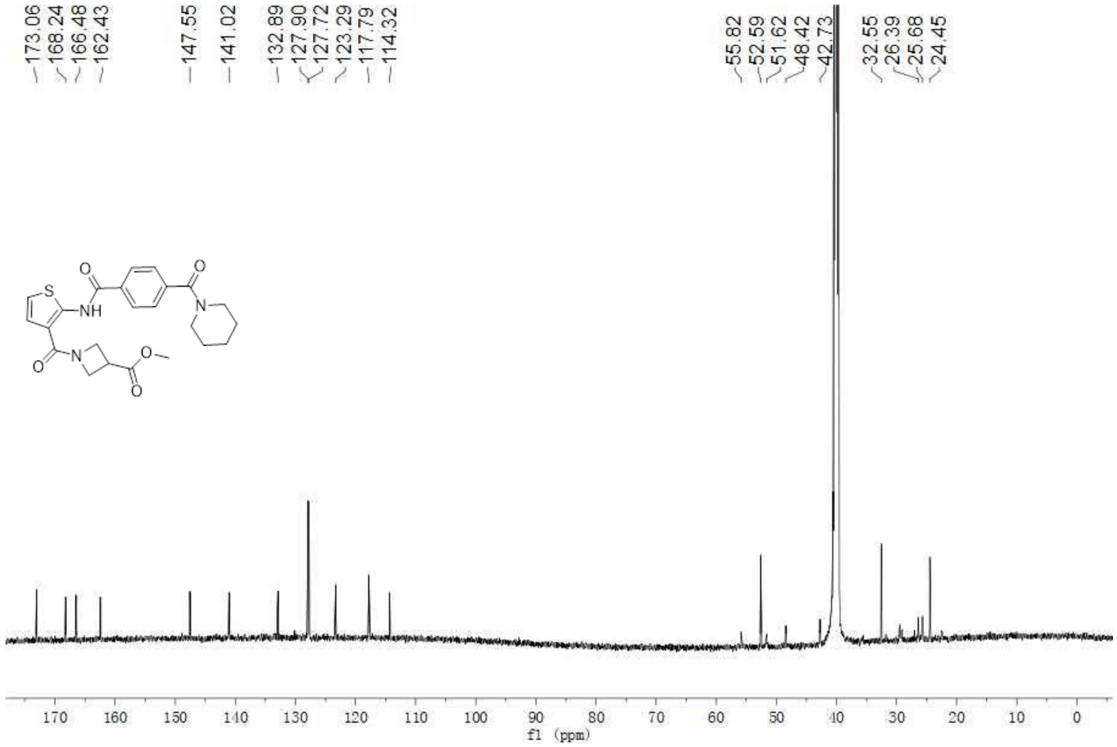

Figure S66. ^{13}C NMR spectrum of **25e**.

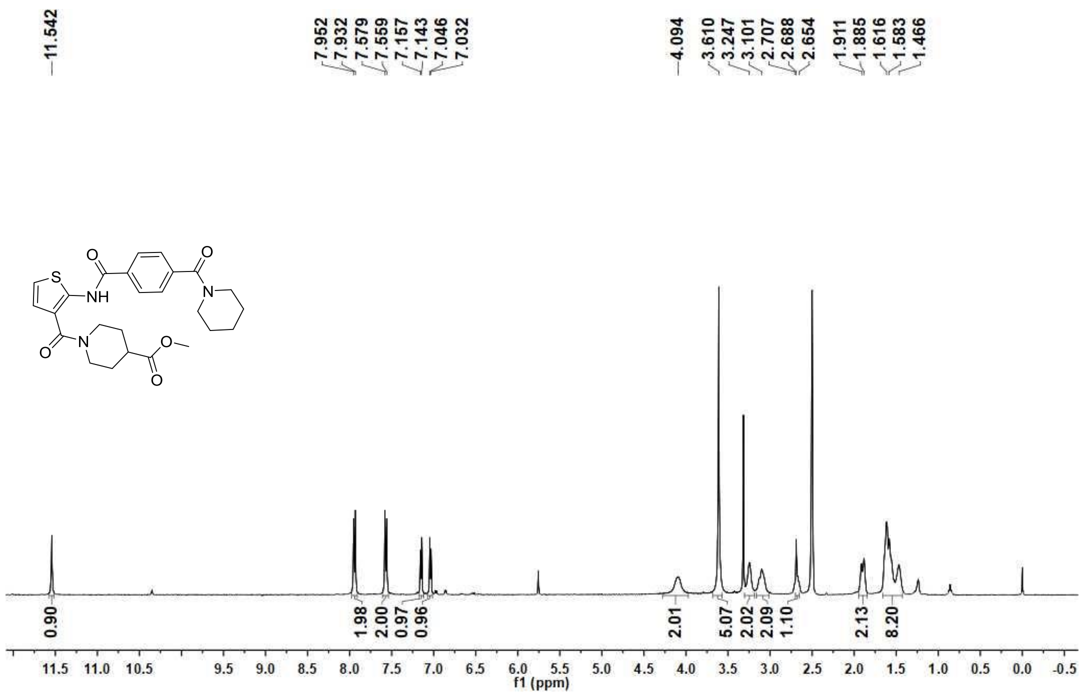

Figure S67. ^{1}H NMR spectrum of **25f**.

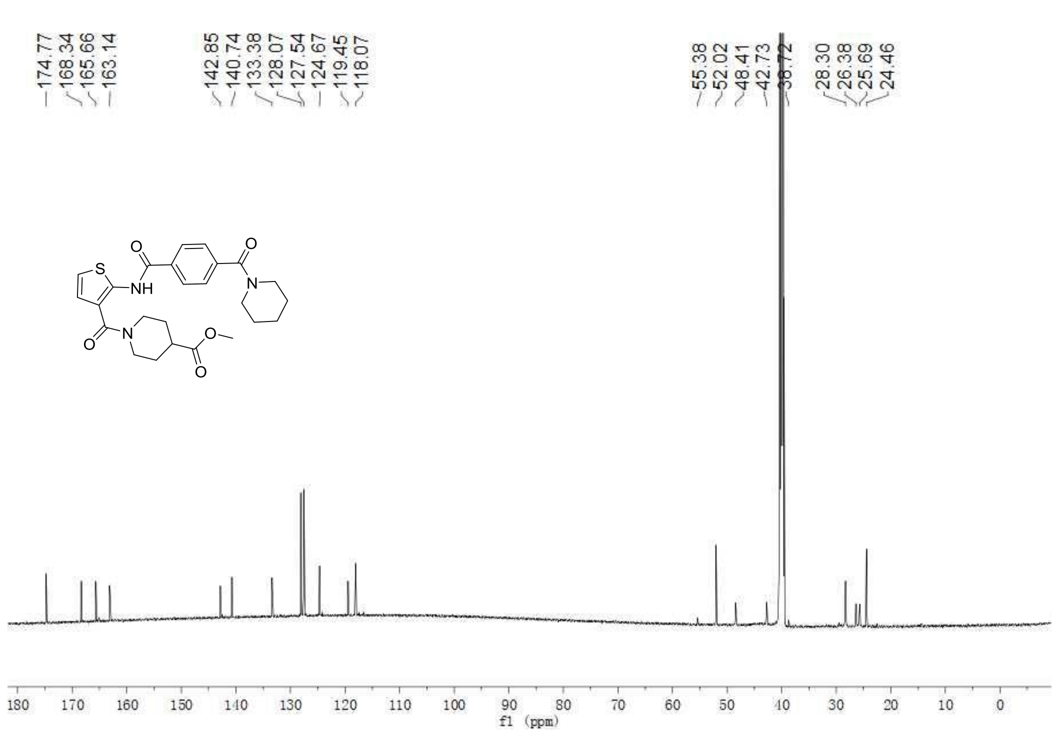

Figure S68. ^{13}C NMR spectrum of **25f**.

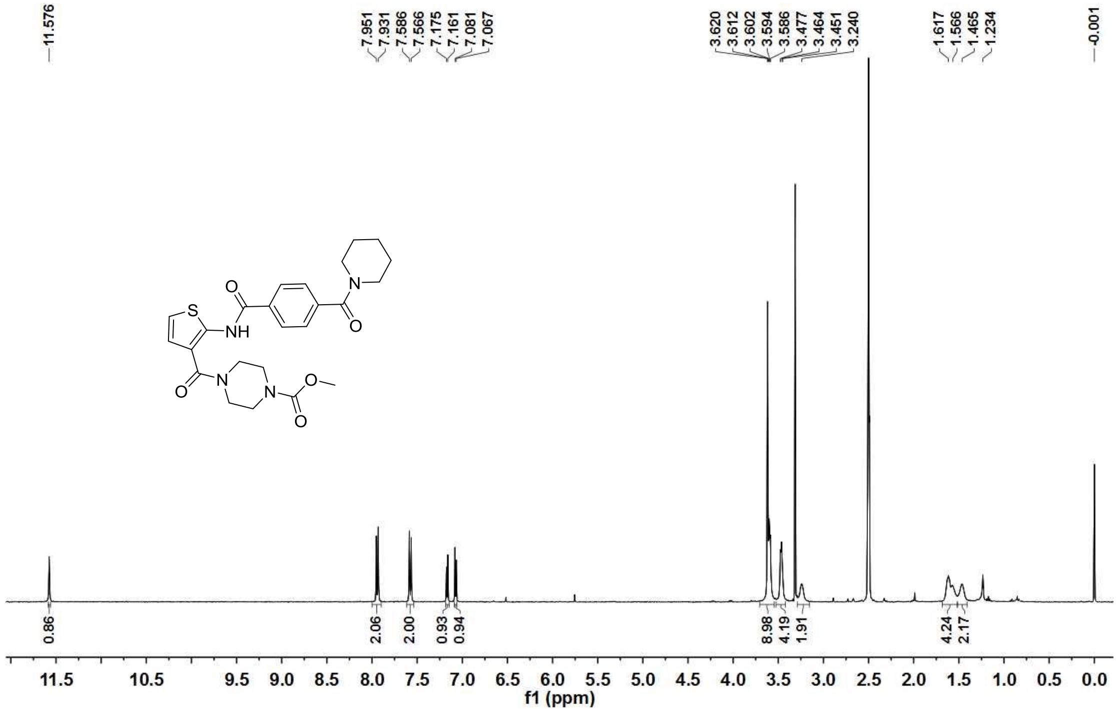

Figure S69. ^{1}H NMR spectrum of **25g**.

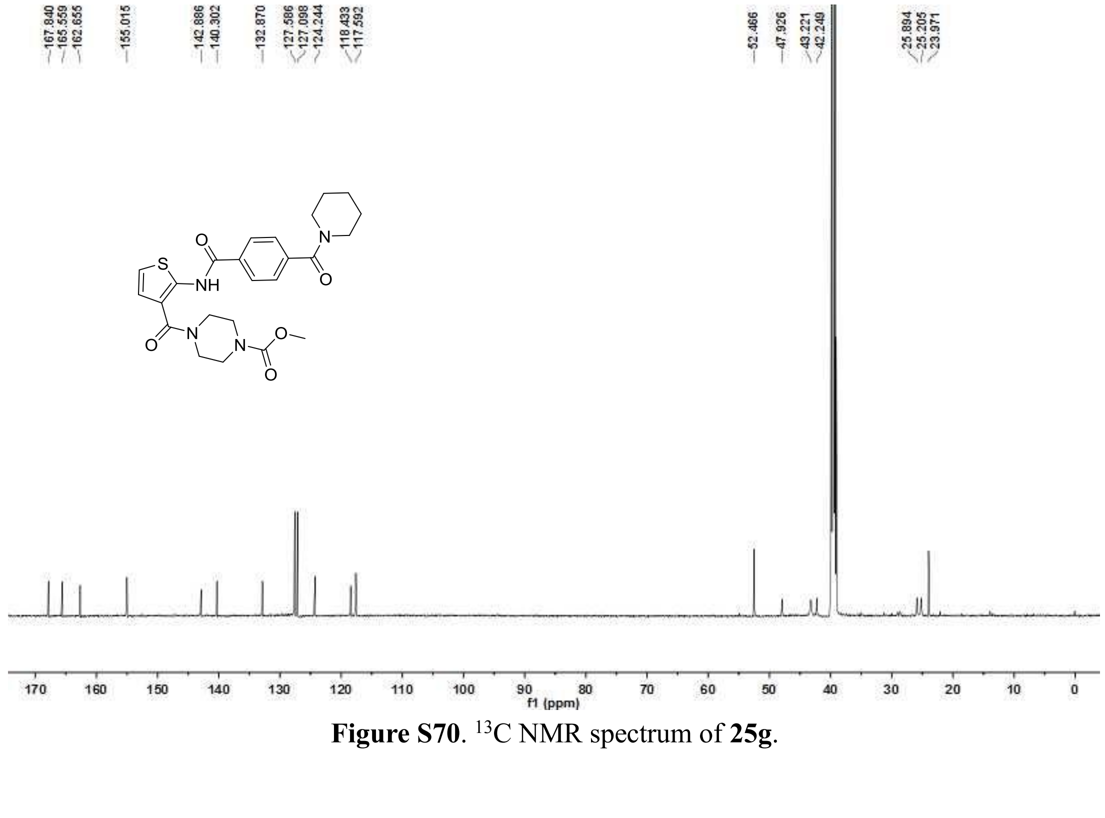

Figure S70. ^{13}C NMR spectrum of **25g**.

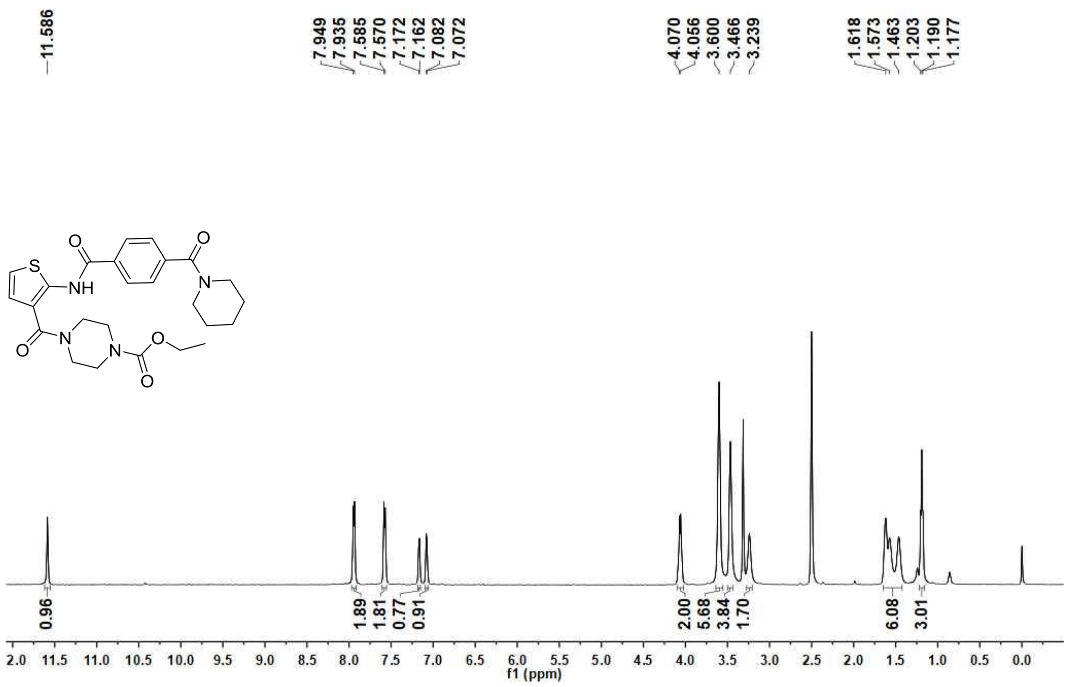

Figure S71. ^{1}H NMR spectrum of **25h**.

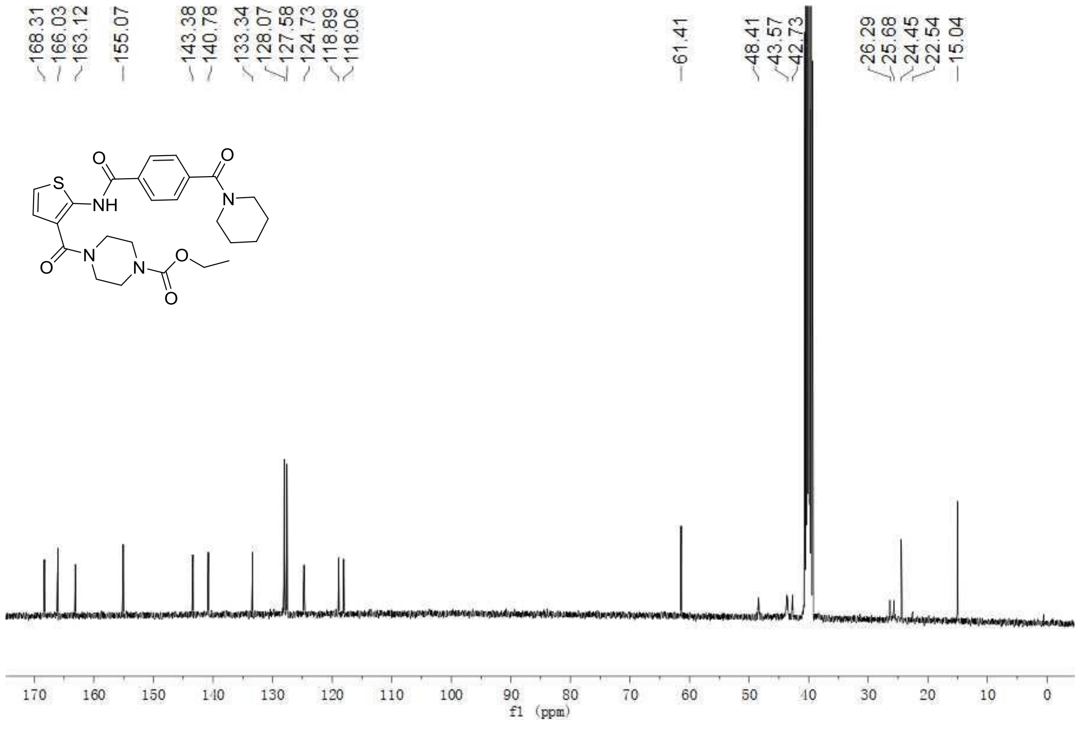

Figure S72. ^{13}C NMR spectrum of **25h**.

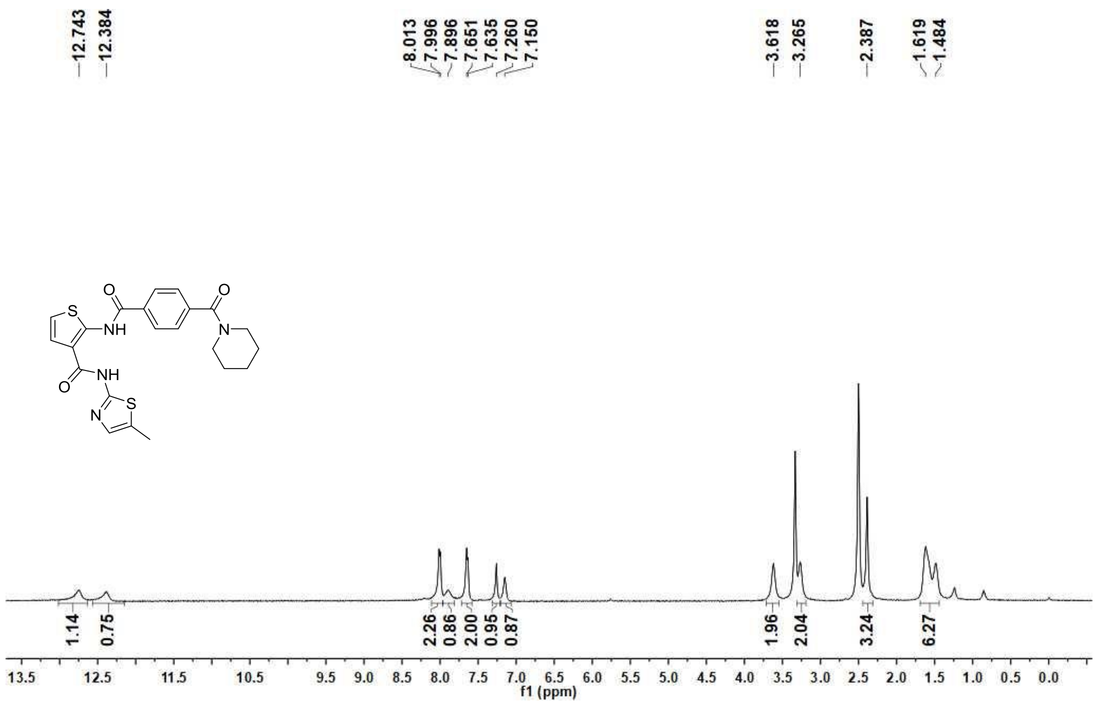

Figure S73. ^{1}H NMR spectrum of **25i**.

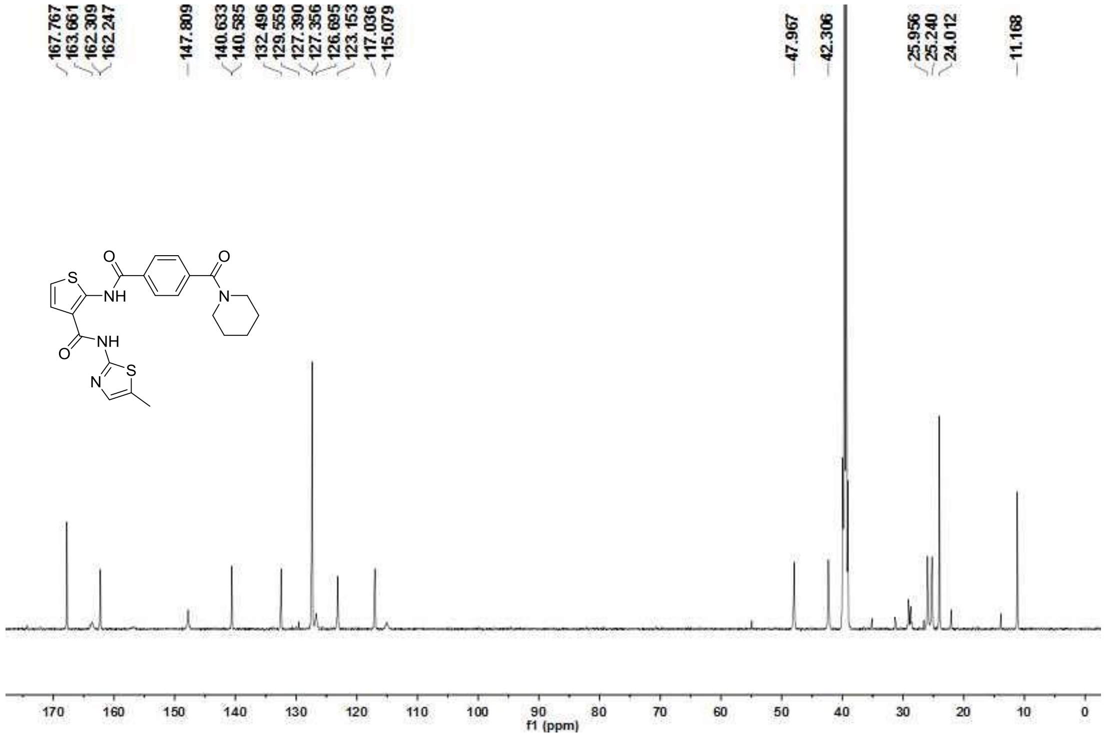

Figure S74. ^{13}C NMR spectrum of **25i**.

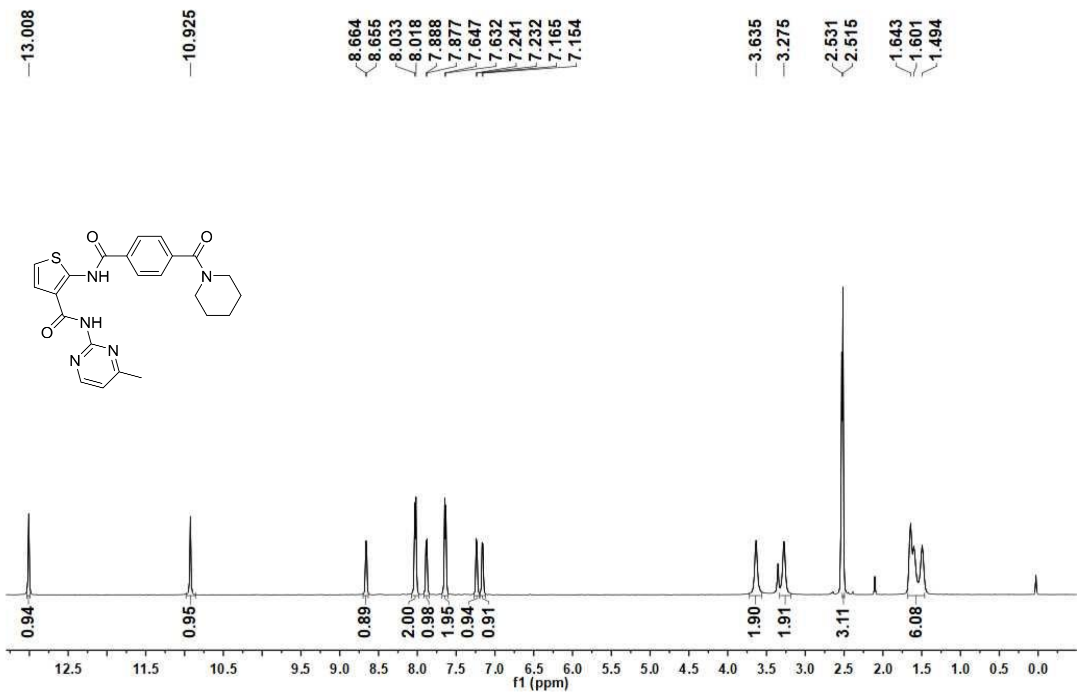

Figure S75. ^{1}H NMR spectrum of **25j**.

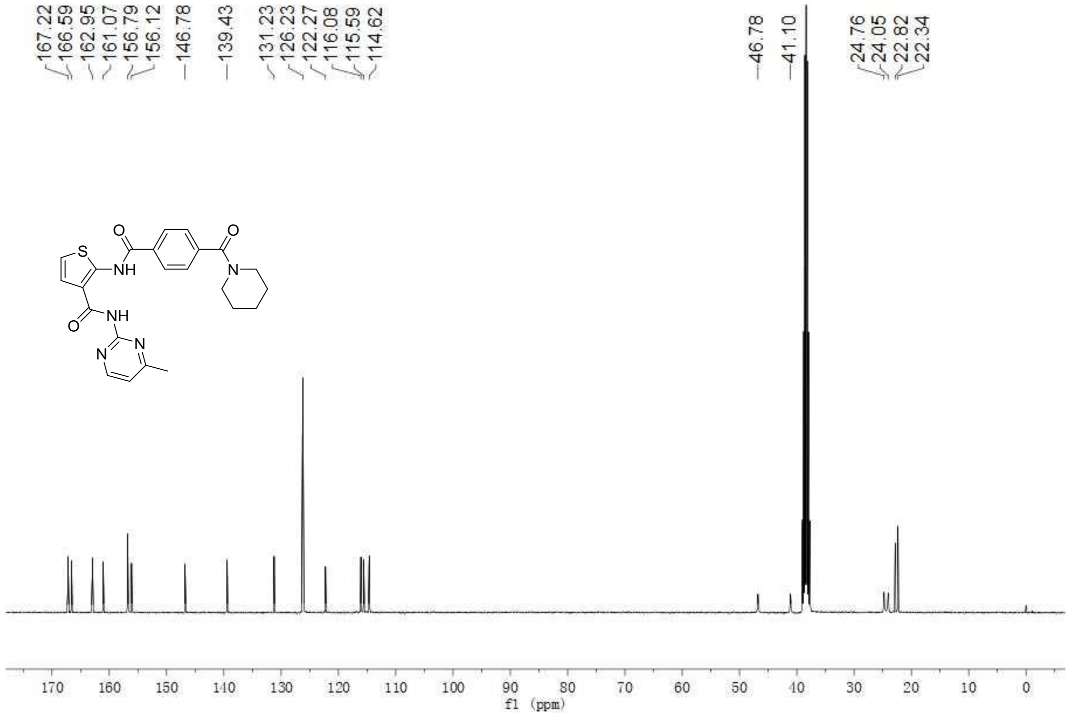

Figure S76. ^{13}C NMR spectrum of **25j**.

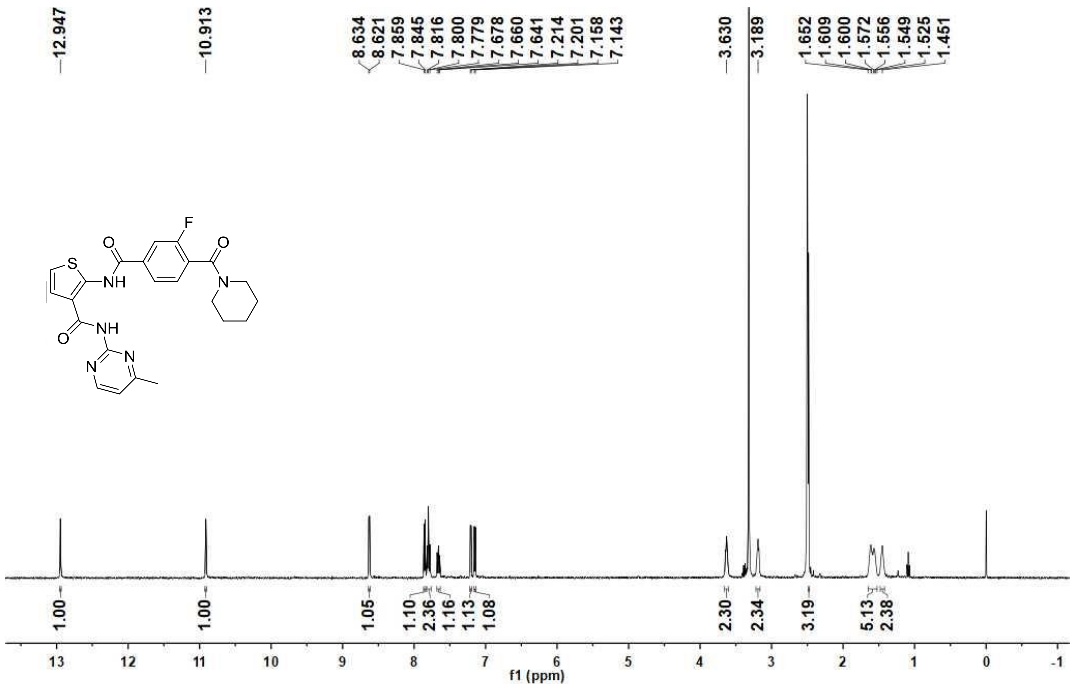

Figure S77. ^{1}H NMR spectrum of **25k**.

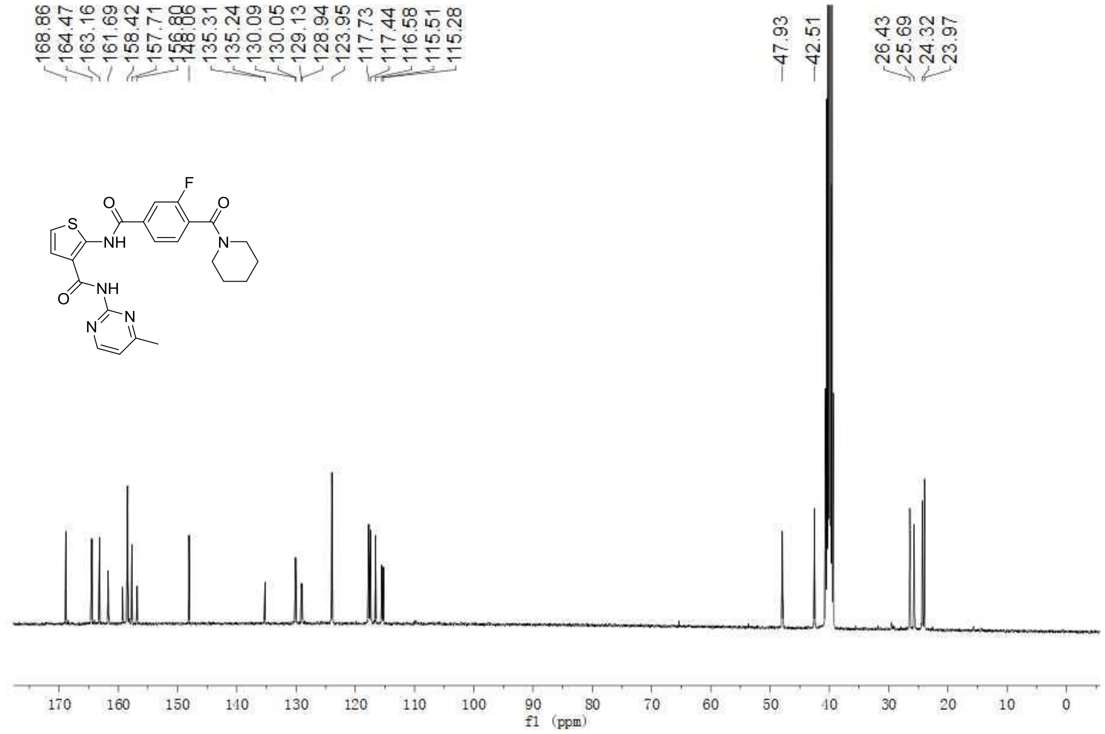

Figure S78. ^{13}C NMR spectrum of **25k**.

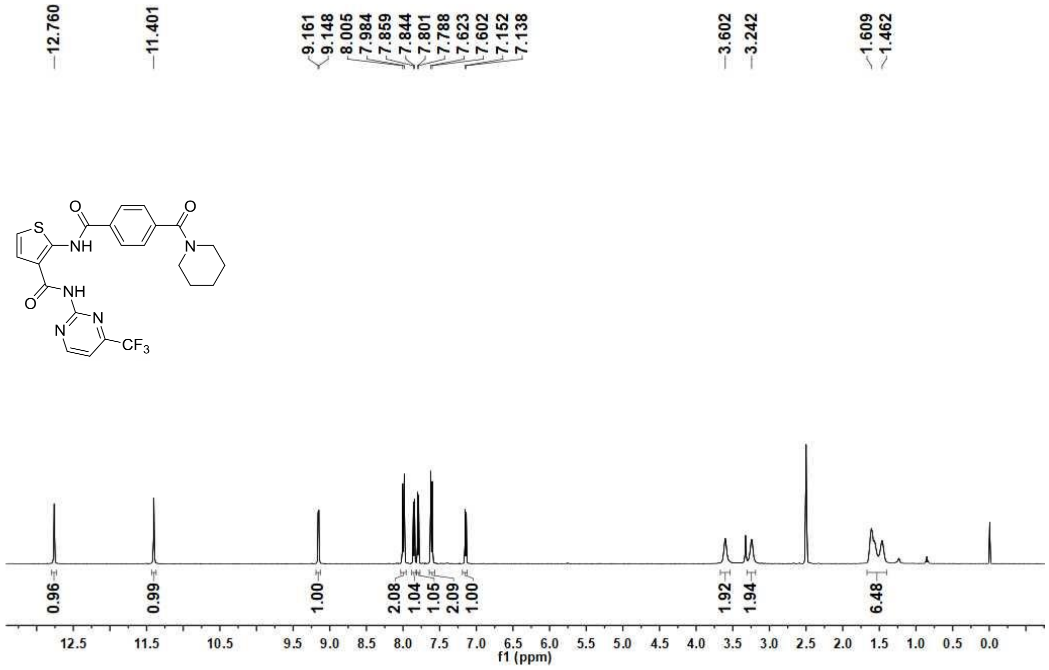

Figure S79. ^{1}H NMR spectrum of **25l**.

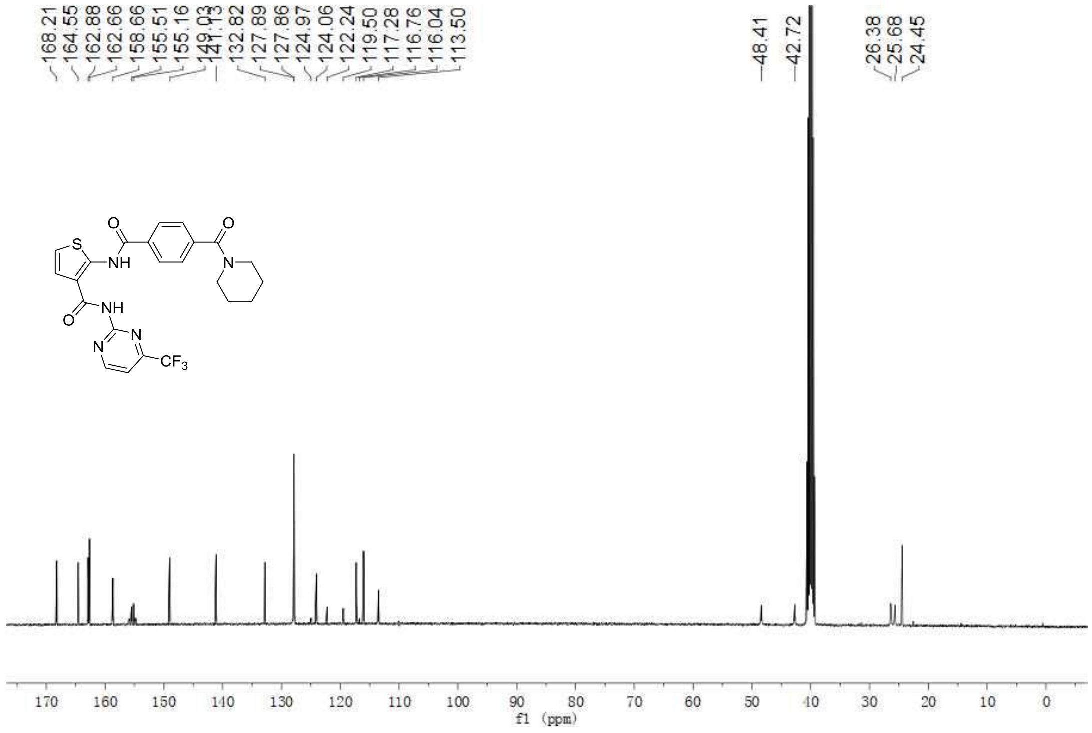

Figure S80. ^{13}C NMR spectrum of **25l**.

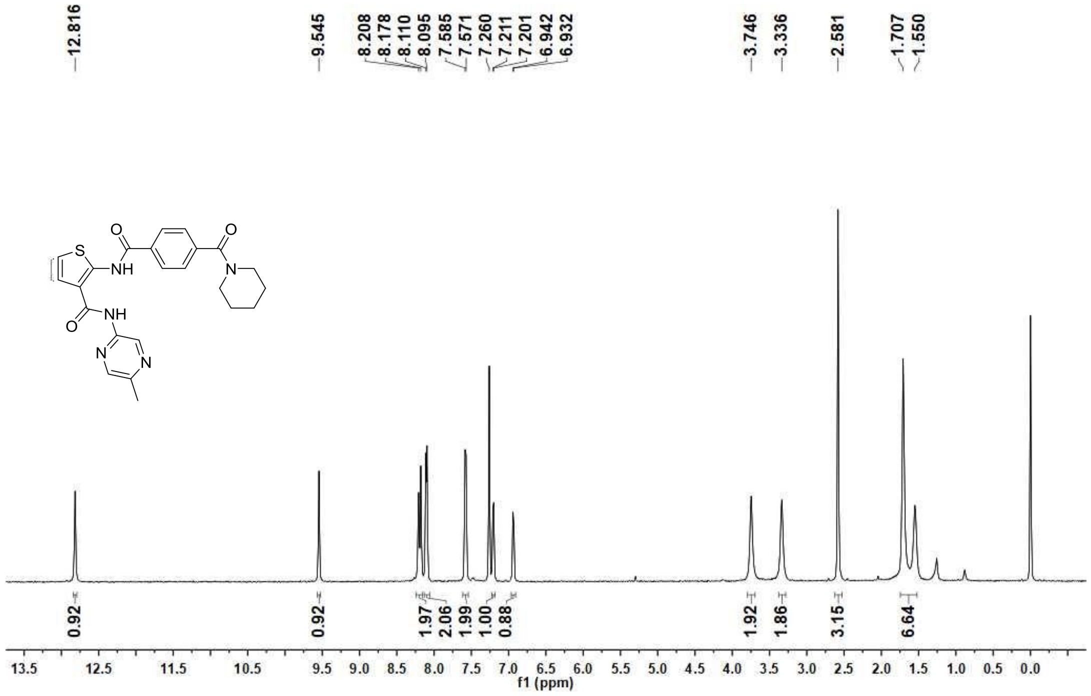

Figure S81. ^{1}H NMR spectrum of **25m**.

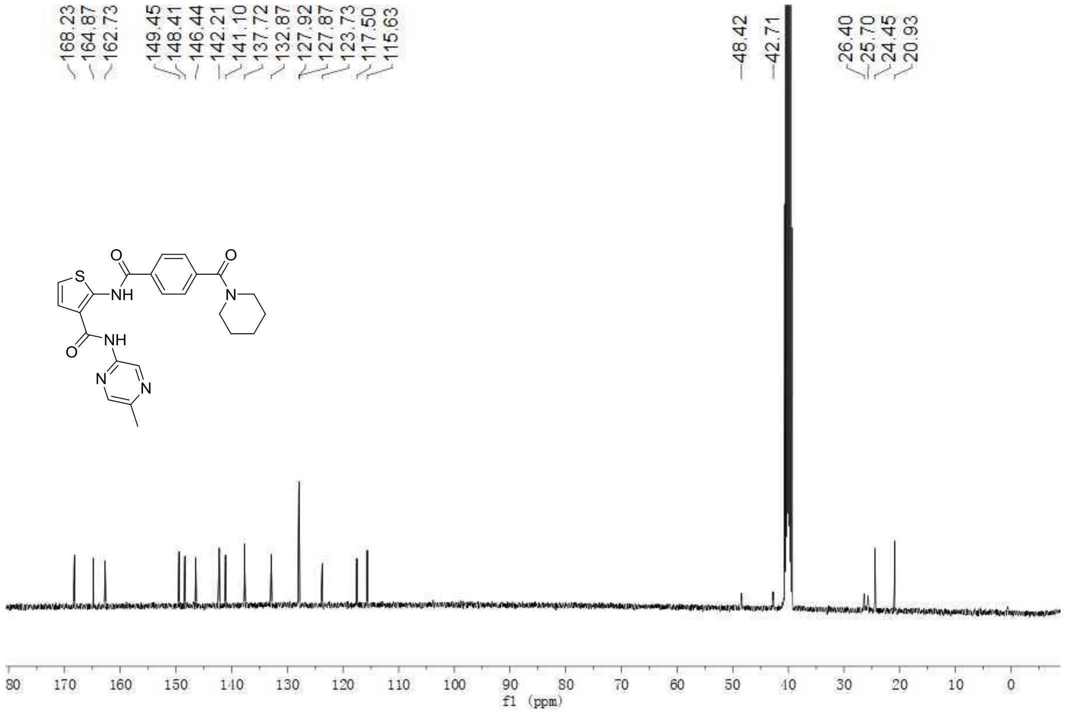

Figure S82. ^{13}C NMR spectrum of **25m**.

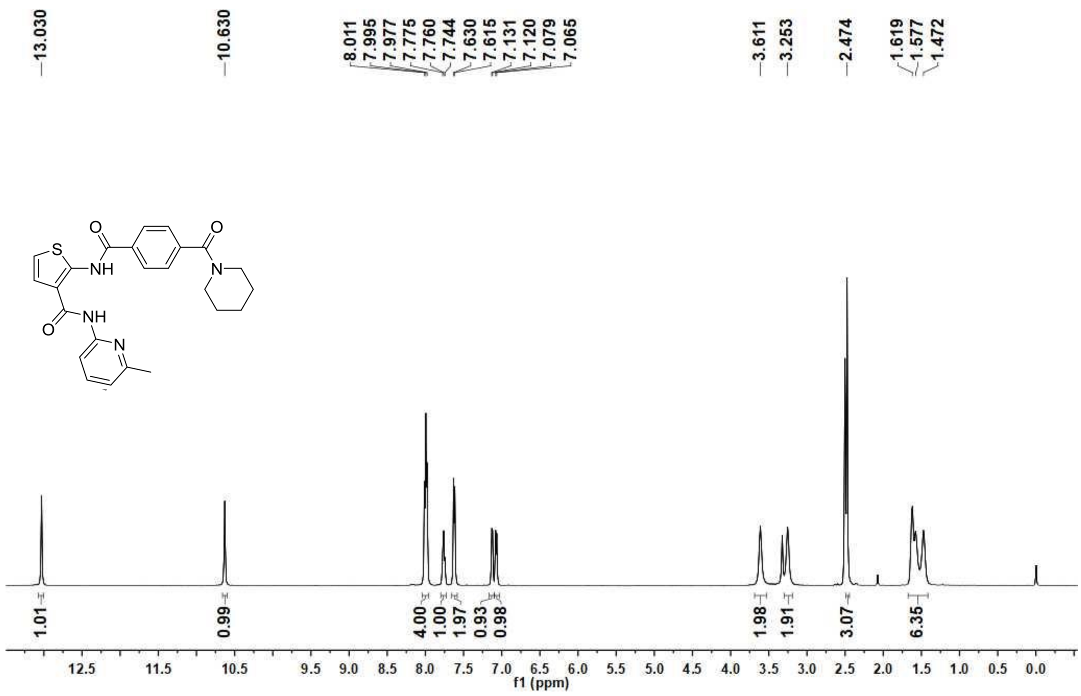

Figure S83. ^{1}H NMR spectrum of **25n**.

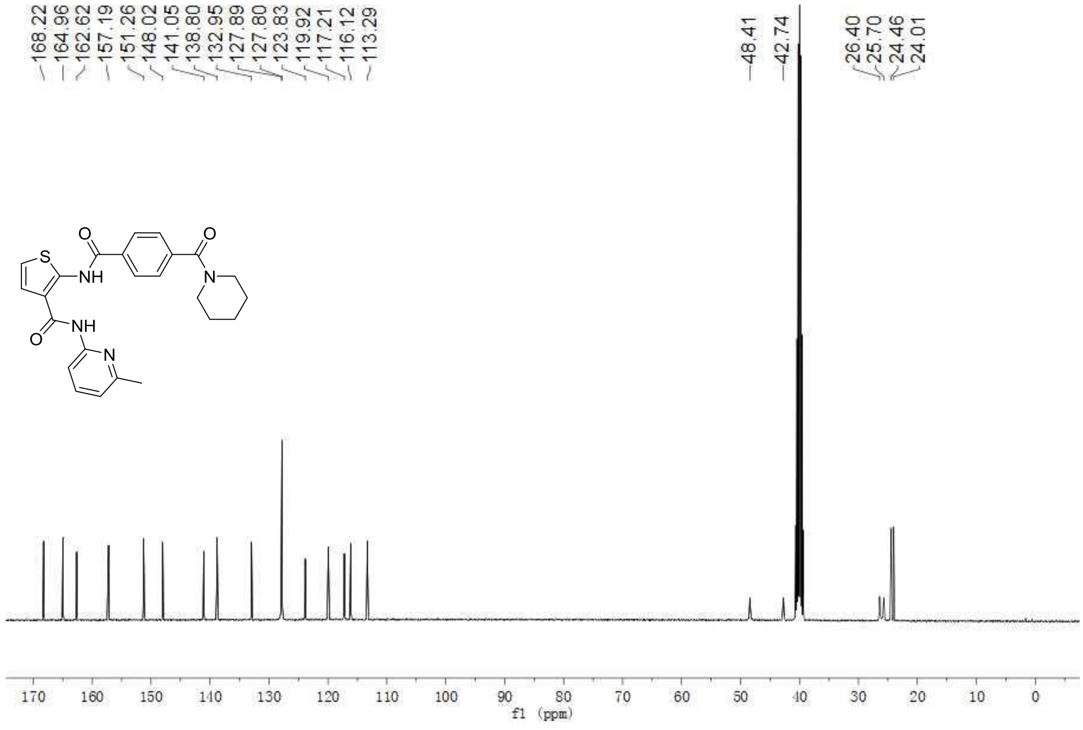

Figure S84. ^{13}C NMR spectrum of **25n**.

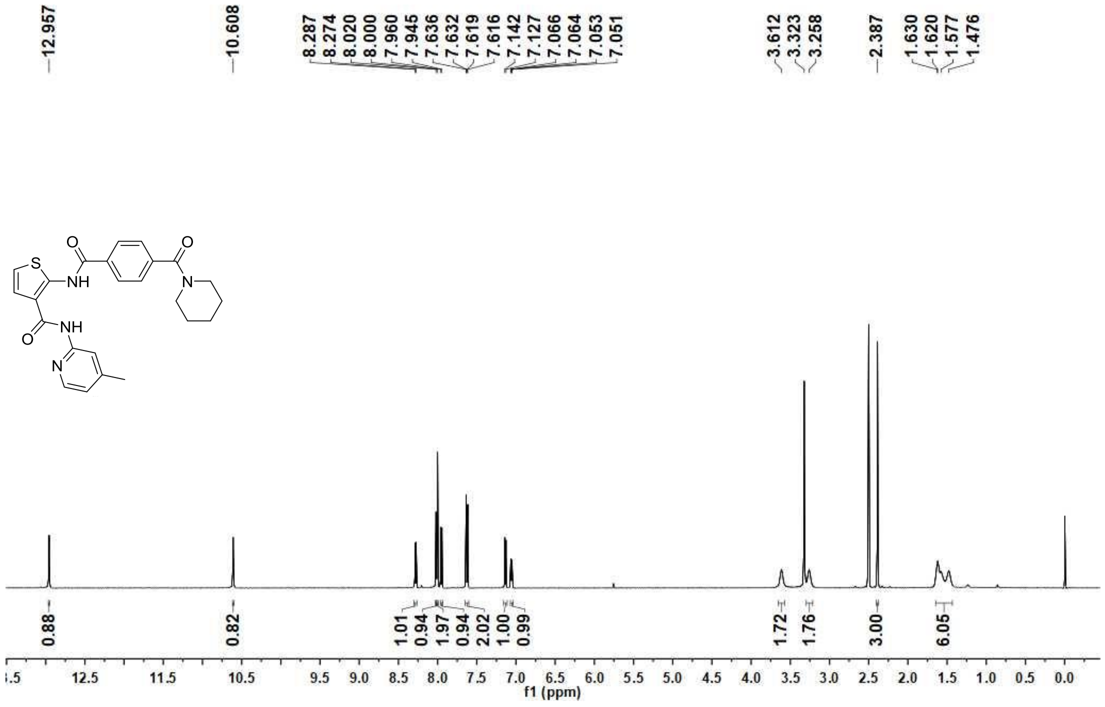

Figure S85. ^{1}H NMR spectrum of **25o**.

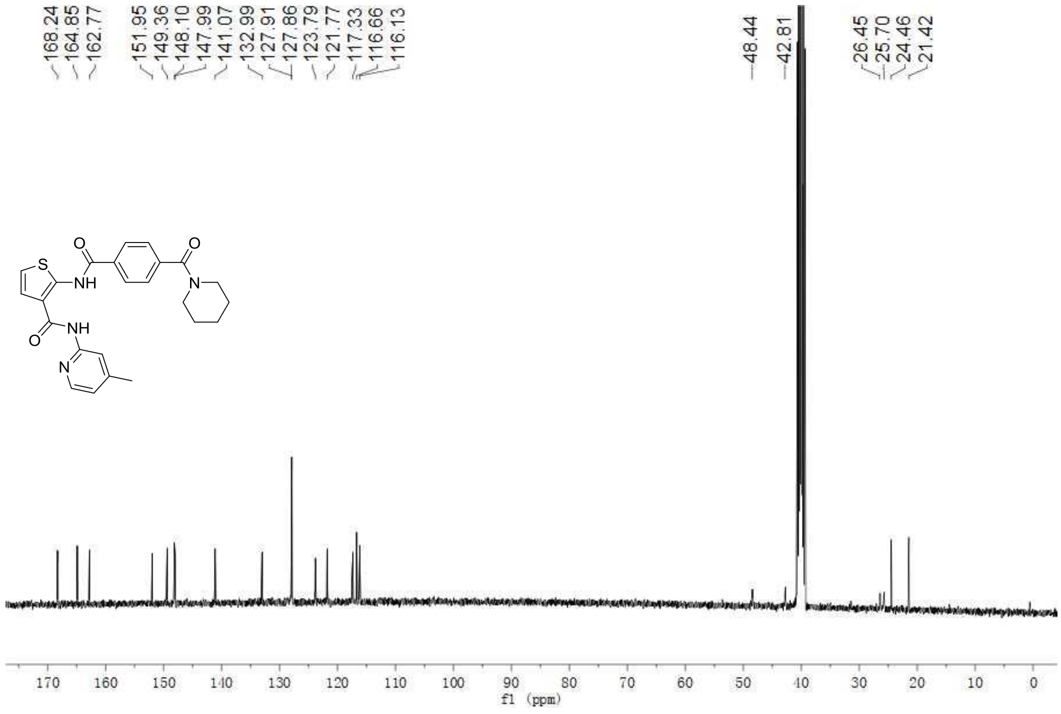

Figure S86. ^{13}C NMR spectrum of **25o**.

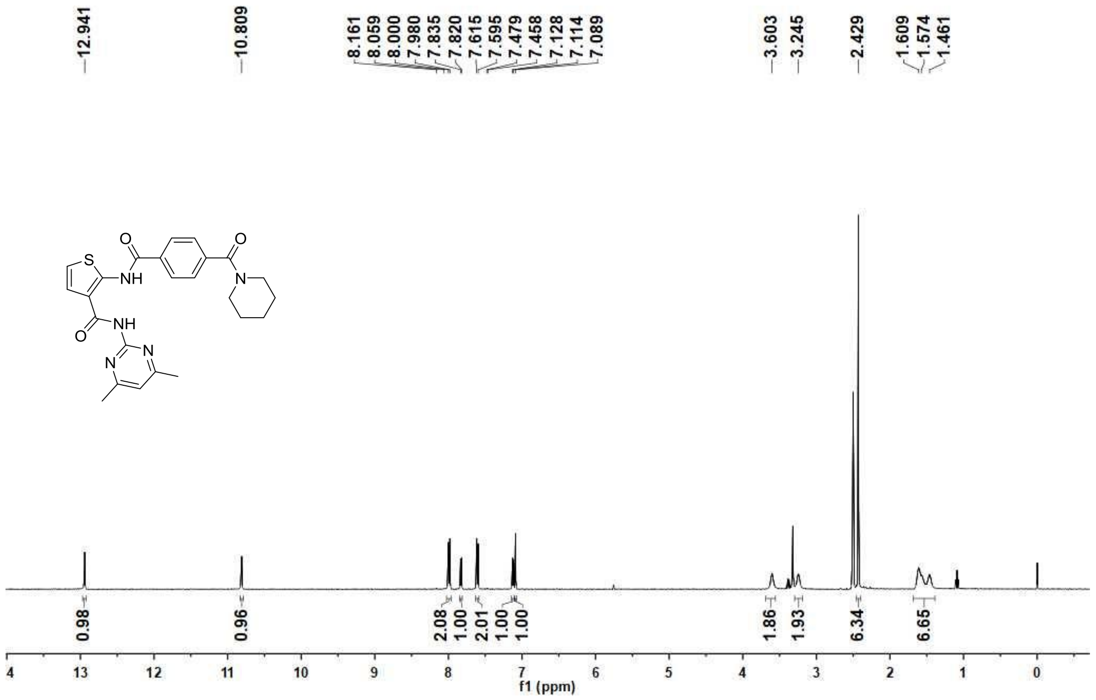

Figure S87. ^{1}H NMR spectrum of **25p**.

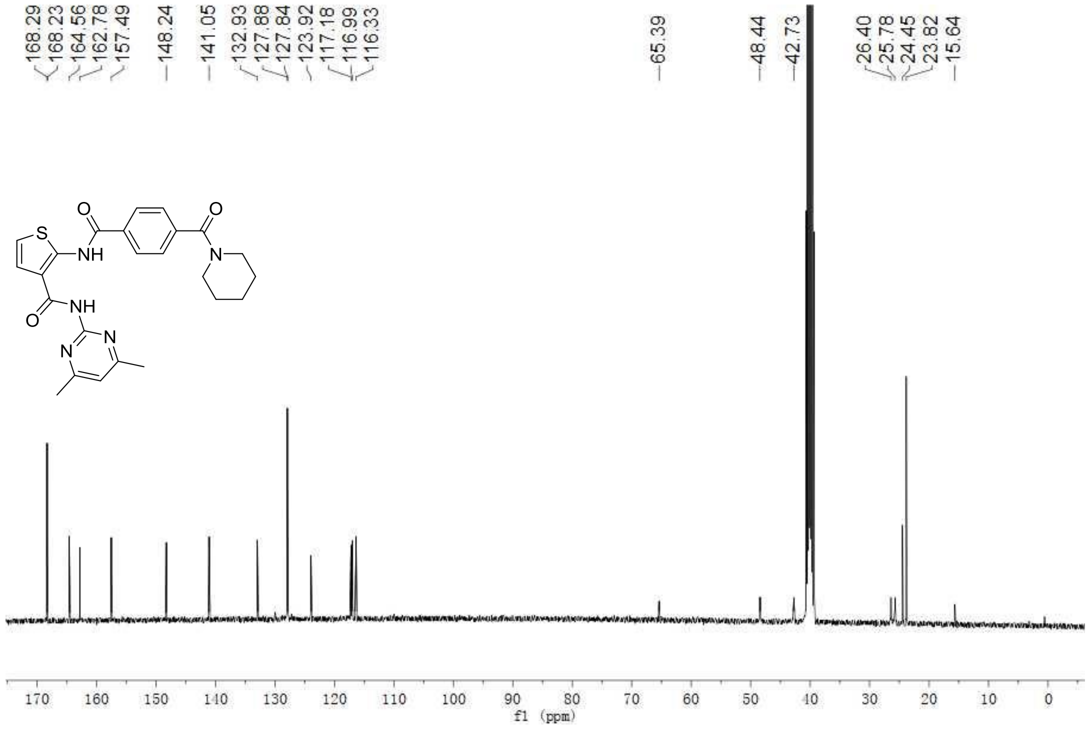

Figure S88. ^{13}C NMR spectrum of **25p**.

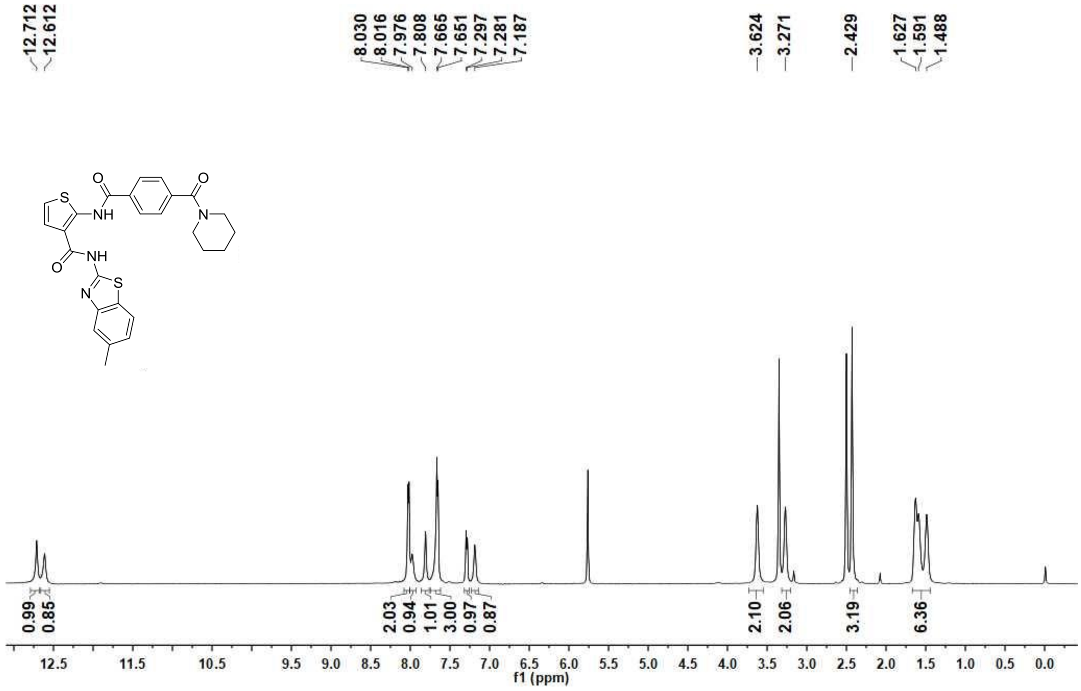

Figure S89. ^{1}H NMR spectrum of **25q**.

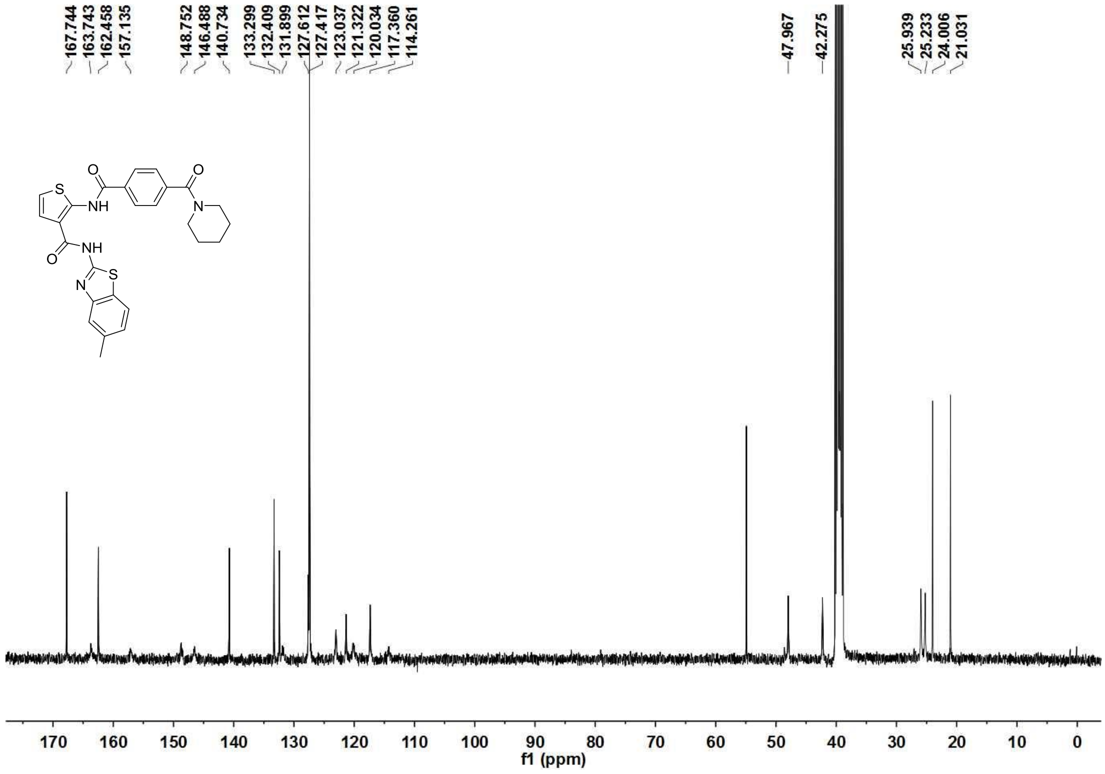

Figure S90. ^{13}C NMR spectrum of **25q**.

Molecular docking

Molecular docking calculations were conducted using CDOCKER protocol in Discovery Studio 2018. Firstly, the protein structure (4KW5) was firstly prepared by adding the hydrogen atoms, inserting the missing loop regions, removing the water molecules, adding missing atoms and deleting alternate conformations (disorder) using the Prepare Protein protocol. The docking protocol reproduced the binding modes of TCA1 with an RMSD of less than 1 Å from the crystallographic pose. Secondly, compound **23j** was minimized by Full Minimization tool using CHARMm forcefield. The radius of binding site was set as 12 Å. Then, compound **23j** was docked to the binding site. The pose cluster radius was set as 0.5 Å. This procedure consisted in a simulated annealing refinement (2000 steps at 700 K, 5000 steps at 300 K) followed by a final full force field minimization using CHARMm. The top 10 poses were retained with the favorable binding mode, and one of them has been selected with highest -CDOCKER_ENERGY.
